# Trends and widening inequities in cardiovascular–kidney–metabolic involvement in cardiovascular mortality: A national spatiotemporal analysis, 2014–2023

**DOI:** 10.1016/j.clinme.2026.100607

**Published:** 2026-06-13

**Authors:** Kaide Xia, Bingpeng Gao, Junwen Wang

**Affiliations:** a Guiyang Maternal and Child Health Care Hospital, Guiyang Children’s Hospital, Guiyang, Guizhou, China; b Department of Urology, Zhejiang Provincial People's Hospital Bijie Hospital, Bijie, Guizhou, China; c Department of Psychosomatic Medicine, The Second People’s Hospital of Guiyang, Guiyang, Guizhou, China

**Keywords:** Cardiovascular disease, Cardiovascular–kidney–metabolic, Mortality, Social vulnerability, Spatial analysis, United States

## Abstract

**Background:**

Cardiovascular–kidney–metabolic (CKM) conditions increasingly contribute to cardiovascular disease (CVD) mortality, but temporal trends in social inequities and geographic priorities remain unclear. We quantified trends, disparities and county-level priority areas for CKM-involved CVD mortality in the USA.

**Methods:**

Using 2014–2023 US vital statistics, we estimated age-standardised CKM-involved CVD mortality. Negative binomial regression estimated Social Vulnerability Index (SVI) quintile-specific relative risks (RRs) and SVI × time interactions. Inequity was measured by the slope (SII) and relative (RII) index of inequality. Bayesian spatiotemporal smoothing identified 2022–2023 county-level RRs and worsening probability (P[increasing]). High-priority areas were defined by current risk (RR ≥ 1.43) and P[increasing] ≥0.80.

**Results:**

National crude mortality rose from 36.37 per 100,000 (2014–2015) to 47.90 (2022–2023). Disparities significantly widened: the SVI Q5 vs Q1 RR increased from 1.23 (95% CI, 1.18–1.28) to 1.34 (1.29–1.40) (interaction p = 0.0197). Both absolute and relative gradients strengthened (SII: 10.48–21.12; RII: 1.30–1.45). County-level RRs (2022–2023) ranged from 0.24 to 4.87 with regional clustering. Overall, 356 counties (11.4%) were classified as high-risk and worsening. We identified 85 Tier 1 counties and estimated 1,721 national excess deaths.

**Conclusions:**

CKM-involved CVD mortality is increasing, and social inequities are widening. Geographically clustered counties face both high current risk and a high likelihood of worsening. These Tier 1 counties represent critical, actionable targets for place-based CKM prevention and treatment strategies.

## Highlights

• County-level CKM-involved cardiovascular mortality rose in the USA, 2014–2023.
• Geographic inequities widened, with increasing clustering of high-burden counties.
• Higher social vulnerability was linked to greater CKM involvement in CVD deaths.
• Spatiotemporal hot spots identify priority counties for cardio–renal prevention.
• Findings support targeted policies to reduce CKM-related cardiovascular mortality gaps.

## Nomenclature

ATSDR: Agency for Toxic Substances and Disease Registry
BYM2: Besag–York–Mollié 2 (scaled spatial random-effects model)
CDC: Centers for Disease Control and Prevention
CKM: Cardiovascular–kidney–metabolic
CVD: Cardiovascular disease
Gi*: Getis–Ord Gi* statistic
MCD: Multiple Causes of Death
NB: Negative binomial
P(increasing): Posterior probability of an increasing temporal trend
RR: Relative risk
RII: Relative index of inequality
SII: Slope index of inequality
SMR: Standardised mortality ratio
SVI: Social Vulnerability Index
UCD: Underlying cause of death
WONDER: Wide-ranging Online Data for Epidemiologic Research

## Introduction

Cardiovascular disease (CVD) remains the leading cause of death in the USA, though contemporary mortality patterns increasingly reflect the intertwined epidemics of metabolic dysfunction, kidney disease and hypertension.^1^, ^2^, ^3^ In 2023, the American Heart Association formally defined cardiovascular–kidney–metabolic (CKM) syndrome to emphasise that cardiovascular outcomes are inextricably linked to upstream and co-occurring cardiometabolic and renal conditions.^4^ Traditional population surveillance, however, typically relies on a single underlying cause of death.^5^, ^6^ This approach fundamentally underestimates the true burden of CVD occurring within CKM multimorbidity and fails to capture the complex clinical reality facing health systems.^7^, ^8^ Death certificates, which document multiple contributing causes, offer a critical but underutilised opportunity to quantify this ʻCKM component–involved’ CVD mortality.^9^

Social and structural determinants are fundamental drivers of these intersecting risks. Counties with greater social vulnerability – characterised by concentrated poverty, lower educational attainment, limited transportation and crowded housing – often experience a syndemic of diabetes, chronic kidney disease, obesity and uncontrolled hypertension, compounded by reduced access to preventive care.^10^ While socioeconomic gradients in individual cardiometabolic outcomes are well-documented, it remains unclear whether the mortality gap between the most and least vulnerable communities has narrowed, persisted, or widened in recent years, particularly following the COVID-19 pandemic.^11^, ^12^ Furthermore, actionable surveillance at the local level faces substantial methodological challenges: data suppression in sparsely populated areas and spatial dependence often obscure true geographic patterns, hindering the precise identification of high-priority jurisdictions for resource allocation.^13^, ^14^

To address these gaps, we constructed a robust, county-level surveillance framework for CKM component-involved CVD mortality, defined as deaths with CVD as the underlying cause and at least one CKM component listed on the death certificate.^15^ Using the Centers for Disease Control and Prevention (CDC) Wide-ranging ONline Data for Epidemiologic Research (WONDER) multiple-cause mortality data from 2014–2023 aligned with contemporaneous Social Vulnerability Index (SVI) measures, this study aimed to quantify national temporal trends and the persistence of social gradients,^10^ rigorously test whether social disparities have widened over time using relative and absolute inequality metrics, and apply Bayesian spatiotemporal smoothing to identify specific counties characterised by both high current risk and a high probability of worsening trends.^16^ By integrating multiple-cause mortality surveillance with advanced inequality metrics and geospatial prioritisation, this work provides a timely, policy-relevant roadmap for targeting CKM-informed interventions to areas of greatest need.

## Methods

This study is a county-level repeated cross-sectional ecological analysis of US death certificate data. We assessed trends in the burden of CVD mortality involving CKM components from 2014 to 2023, examined disparities by social vulnerability, and identified priority intervention areas characterised by both high risk and a persistently worsening trend. The analysis period was aggregated into 2-year blocks (2014–2015, 2016–2017, 2018–2019, 2020–2021, 2022–2023) to mitigate the suppression of small death counts (0–9) at the county level, enhance estimation stability, and temporally align social vulnerability exposure with mortality outcomes. Because this study used publicly available, de-identified, aggregated data, it was exempt from institutional review board review and informed consent was not required.

Mortality data were sourced from the Multiple Cause of Death (MCD) database of the National Center for Health Statistics, accessed via the CDC WONDER platform. The 1999–2020 MCD file was used for 2014–2017, and the MCD Expanded file was used for 2018–2023, based on platform availability.^17^ Data were extracted with county and 2-year block as the basic units, yielding observed death counts (O) and population counts. County-level population and death counts stratified by age group were also extracted to control for age structure. The SVI was obtained from county-level data released by CDC/Agency for Toxic Substances and Disease Registry (ATSDR).^15^ The primary analysis used the SVI national percentile (0–1) and its quintiles (Q1–Q5, with Q1 representing the lowest vulnerability). SVI data were aligned with mortality blocks by year: 2014–2015 with SVI 2014, 2016–2017 with SVI 2016, 2018–2019 with SVI 2018, 2020–2021 with SVI 2020, and 2022–2023 with SVI 2022.

The primary outcome was ʻCKM component-involved CVD mortality,’ defined as deaths where the underlying cause of death (UCD) was CVD and at least one CKM component was mentioned among the multiple causes of death. UCD was defined using International Classification of Diseases (Tenth Revision) codes I00–I99. CKM components in the primary analysis included diabetes (E10–E14), chronic kidney disease (N18.), obesity (E66.) and hypertension-related diseases (I10–I15). In CDC WONDER, UCD was used as the primary filter, and the aforementioned components were filtered as an OR set using the MCD ʻany mention’ option. The MCD ʻmention’ reflects comorbid or contributing conditions recorded on the death certificate; this study does not make individual-level causal attributions.

To control for differences in age structure between counties, we employed an indirect standardisation framework using the standardised mortality ratio (SMR) to construct expected death counts for use as an offset in models. For each 2-year block, national age-specific mortality rates were first calculated under the same outcome definition. The expected death count (E) for each county was then calculated by summing the products of these national age-specific rates and the corresponding county age-group populations. In regression models, log(E) was used as an offset to estimate the risk relative to the national age structure. For descriptive purposes, an indirectly standardised mortality rate was calculated by multiplying the county SMR by the national crude mortality rate for that time block.

The primary analysis of trends and disparities used county × time block as the observational unit, modelling O with count regression. Accounting for overdispersion, the primary model was a negative binomial regression, with SVI quintile (Q1 as reference) as the main exposure and an interaction term with time to test whether social disparities changed over time. Time was represented by the midpoint of each 2-year block and centred for interpretability. Model outputs estimated the relative risk (RR) and 95% confidence interval (CI) for Q5 relative to Q1 at specific time points. The statistical significance of the ʻSVI quintile × time’ interaction was assessed using likelihood ratio tests.

To simultaneously quantify relative and absolute inequalities, we calculated inequality indices based on the continuous SVI percentile (0–1). Model-predicted rates at SVI = 0 and SVI = 1 were obtained within the regression framework. At each time point, we calculated the relative inequality index (RII, predicted rate at SVI = 1/predicted rate at SVI = 0) and the absolute inequality index (SII, predicted rate at SVI = 1 − predicted rate at SVI = 0, per 100,000 population). Interval estimates for RII and SII were obtained by propagating model parameter uncertainty.

To enhance the stability of county-level estimates and characterise spatial correlation and temporal trends, we constructed a Bayesian space–time model to smooth county-level relative risks. The model specified O as a Poisson count process with log(E) as an offset. A Besag–York–Mollié 2 model (BYM2) spatial structure was incorporated to capture spatial autocorrelation, and a first-order random walk described the smoothly varying overall temporal trend.^18^, ^19^ State-level heterogeneity terms and additional independent error terms were added when necessary. Different model specifications were compared using the Deviance Information Criterion and the Watanabe–Akaike Information Criterion. The model with superior fit and stronger interpretability was selected as the primary space–time smoothing framework.

To identify ʻhigh-risk and worsening’ areas, we calculated the ʻprobability of worsening’ P(increasing) for each county from the Bayesian model, defined as the posterior probability that the county-specific trend parameter was positive. Counties were then classified using a bivariate scheme based on ʻcurrent risk level’ and ʻprobability of worsening’: counties with RR-mean in the latest period at or above the 75th percentile were defined as High RR, and counties with P(increasing) ≥0.80 were defined as High P. This created a four-quadrant classification, with a focus on reporting the number and state distribution of counties in the ʻHigh RR + High P’ quadrant. Furthermore, to generate a priority list for resource allocation, we constructed a composite priority score integrating risk level, probability of worsening, and population size. Based on this score, the highest-priority Tier 1 counties were defined.

To validate spatial clustering patterns and corroborate risk maps, we conducted hotspot detection analysis. Getis–Ord Gi* identified hotspot/coldspot areas, and Local Moran’s I assessed local spatial autocorrelation types.^20^, ^21^ To quantify the public health impact in the latest period, we calculated county-level excess deaths, defined as (RR − 1) × E, where RR is the smoothed county-level relative risk and E is the expected death count. County-level excess deaths were summed at the national and state levels and converted to excess mortality rates per 100,000 population.

Robustness checks included comparing different negative binomial model specifications and assessing consistency in fit and inference; adding region or state fixed effects to test the stability of key RR estimates; addressing potential structural changes related to the pandemic using alternative specifications; repeating key estimates using different imputation strategies for suppressed death counts; and performing model diagnostics through comparison of observed versus fitted death counts and examination of residuals. All analyses were conducted in R (version 4.5.0), with statistical inference reporting corresponding 95% confidence intervals or credible intervals.

## Results

**Fig. 1:**
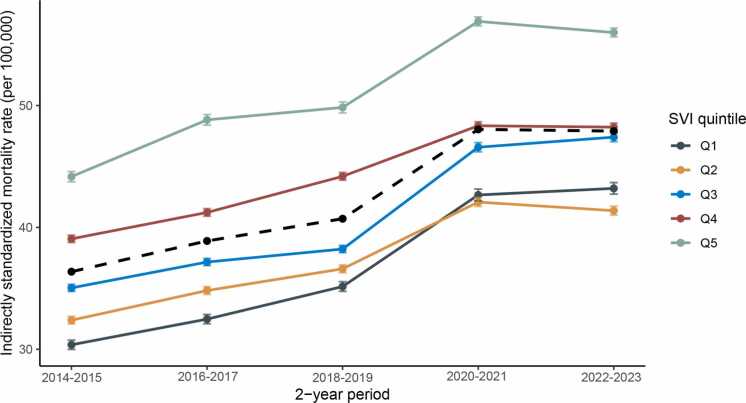
National and SVI-quintile trends in CKM-involved CVD mortality in the United States, 2014–2023. National crude mortality rates (dashed line) and SVI quintile-specific indirectly standardised mortality rates (solid lines; calculated as SMR × national crude rate) are shown across five biennial periods (2014–2015 to 2022–2023; points correspond to period mid-years), with 95% uncertainty intervals. SVI quintiles range from Q1 (least vulnerable) to Q5 (most vulnerable).

National crude CKM-involved CVD mortality increased from 36.37 per 100,000 (2014–2015) to 47.90 (2022–2023), peaking at 48.04 (2020–2021) (Table S1). This trend paralleled improved surveillance completeness, with data suppression declining from 24.9% to 17.9% (Fig. S1). A profound social gradient persisted: standardised rates in the most vulnerable counties (Q5) consistently exceeded the least vulnerable (Q1). Specifically, Q5 rates rose from 44.16 to 55.99, compared with 30.37–43.20 for Q1, underscoring that mortality burden remains heavily concentrated in socially disadvantaged areas (Fig. 1 and Table S2).

**Fig. 2:**
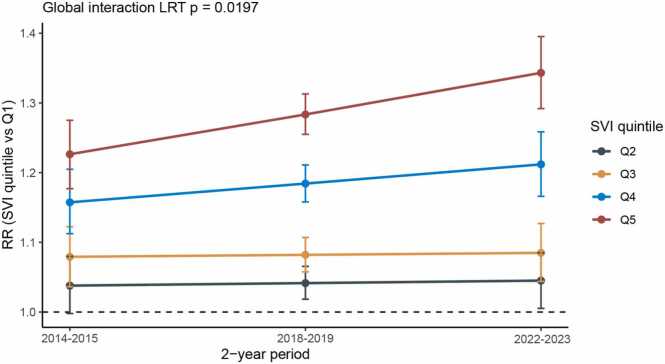
Widening social vulnerability disparities in CKM-involved CVD mortality over time. Negative binomial model-estimated rate ratios (RRs) comparing SVI quintiles Q2–Q5 with Q1 (reference) are shown for 2014–2015, 2018–2019 and 2022–2023 with 95% confidence intervals. The global SVI quintile × time interaction likelihood ratio test is reported to assess temporal widening of relative disparities.

Statistical modelling confirmed that disparities widened over time on both relative and absolute scales. Negative binomial models showed that the relative risk (RR) for Q5 compared with Q1 increased from 1.23 (95% CI, 1.18–1.28) in 2014–2015 to 1.34 (1.29–1.40) in 2022–2023. The significant interaction between SVI quintile and time (p = 0.0197) provided evidence of increasing relative disparities (Table 1 and Fig. 2). This pattern was mirrored on the absolute scale: the slope index of inequality (SII) approximately doubled from 10.48 (95% CI, 8.76–12.22) to 21.12 (18.80–23.55) per 100,000 over the same period. Similarly, the relative RII rose from 1.30 to 1.45, indicating that the mortality burden became increasingly concentrated in high-SVI communities (Table 1).

**Table 1:** Social vulnerability disparities in CKM-involved CVD mortality over time: negative binomial rate ratios and inequality indices.

**Panel A. Relative disparities by SVI quintile**				
**Period**	**RR (Q2 vs Q1)**	**RR (Q3 vs Q1)**	**RR (Q4 vs Q1)**	**RR (Q5 vs Q1)**
2014–2015	1.04 (1.00–1.08)	1.08 (1.04–1.12)	1.16 (1.11–1.20)	1.23 (1.18–1.28)
2018–2019	1.04 (1.02–1.07)	1.08 (1.06–1.11)	1.18 (1.16–1.21)	1.28 (1.25–1.31)
2022–2023	1.05 (1.01–1.09)	1.08 (1.04–1.13)	1.21 (1.17–1.26)	1.34 (1.29–1.40)
**Panel B. Inequality indices from continuous SVI**				
**Period**	**Predicted rate at SVI = 0, per 100,000**	**Predicted rate at SVI = 1, per 100,000**	**RII (SVI = 1 vs 0)**	**SII (SVI = 1–0)**
2014–2015	34.58	45.06	1.30 (1.25–1.36)	10.48 (8.76–12.22)
2018–2019	39.16	53.89	1.38 (1.34–1.41)	14.73 (13.56–15.87)
2022–2023	46.62	67.75	1.45 (1.40–1.52)	21.12 (18.80–23.55)

Global interaction LRT p = 0.0197.

**Fig. 3:**
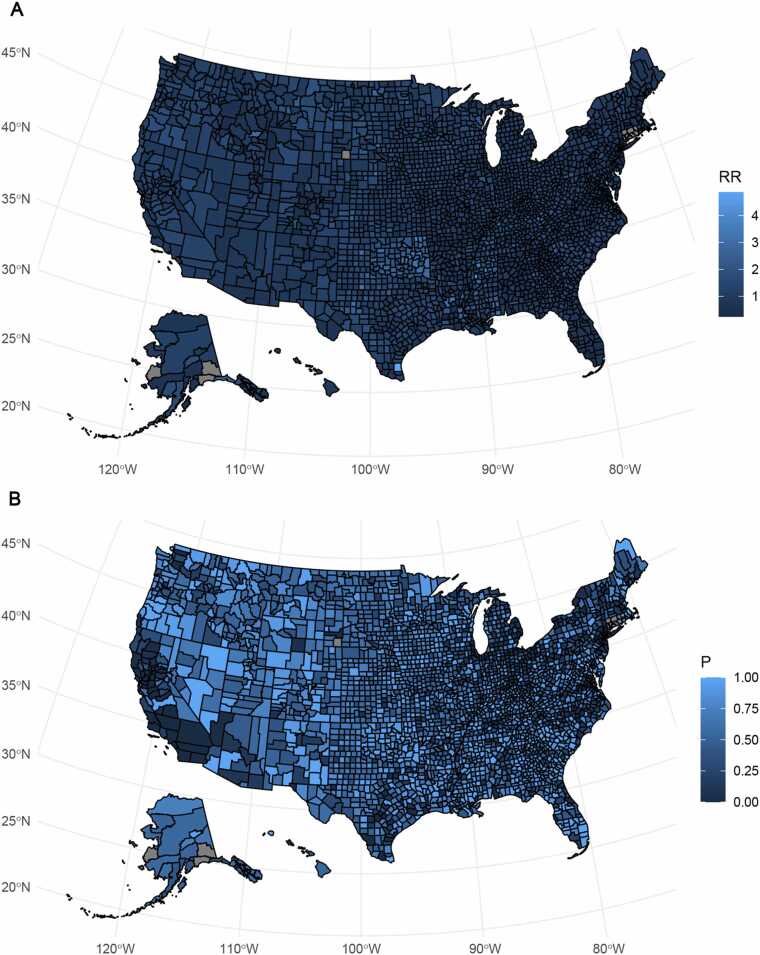
County-level smoothed risk and worsening probability of CKM-involved CVD mortality in the USA, 2022–2023. (A) Smoothed county-level RRs in 2022–2023. (B) Worsening probability, defined as P(increasing).

**Fig. 4:**
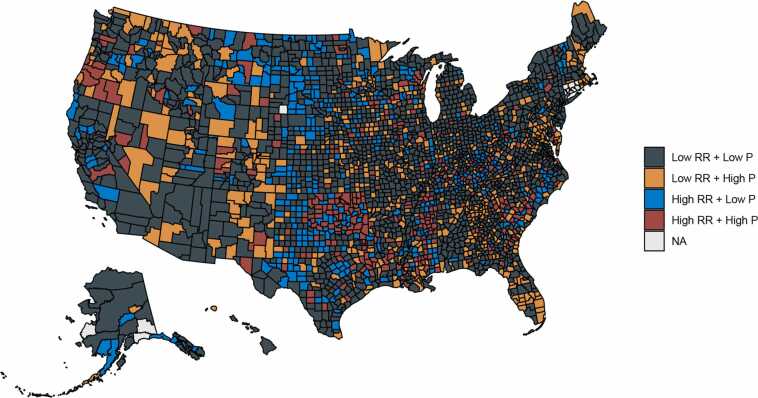
Bivariate county classification by current risk and worsening probability for CKM-involved CVD mortality, 2022–2023. Counties are classified into four groups based on smoothed RR in 2022–2023 (current risk) and P(increasing) (worsening probability): Low RR + Low P, Low RR + High P, High RR + Low P, and High RR + High P (high risk defined as RR ≥1.43 and high probability as P(increasing) ≥0.80). NA indicates counties without available estimates.

Beyond temporal trends, mortality risk in 2022–2023 exhibited marked geographic heterogeneity. Smoothed county-level RRs spanned a wide range (0.24–4.87), with elevated risks clustering in distinct regions rather than appearing as isolated outliers (Fig. 3). To identify areas requiring urgent intervention, we classified counties based on both current risk (RR) and the probability of an increasing trend (P[increasing]) (Fig. 4). We identified 356 counties (11.4%) with concurrent high risk (RR ≥ 1.43) and worsening probability (P ≥ 0.80), predominantly clustered in Oklahoma, Mississippi, Texas, Georgia and Kentucky (Tables S3–S4).

To translate these risk metrics into actionable priorities, we identified 85 ʻTier 1’ counties with the highest composite priority scores. This subset included both large urban centres with a high certainty of increase (eg Tulsa County, OK) and smaller counties with extreme relative risks (Table 2 and Table S5). Tier 1 counties were highly concentrated, with Oklahoma accounting for 35.1% of its counties in this top tier (Table S4). The public health impact of these disparities was substantial: aggregating county-level excess deaths yielded 1,721 excess deaths nationwide in 2022–2023, corresponding to 0.26 excess deaths per 100,000 (observed deaths 316,588 vs expected deaths 314,813 across 3,131 counties). This burden was unevenly distributed, with Oklahoma (62.16 per 100,000) and Mississippi (33.03 per 100,000) experiencing the highest excess mortality rates (Table S6), underscoring the severe human cost of these geographic inequities.

**Table 2:** Top 10 Tier 1 priority counties for CKM-involved CVD mortality in 2022–2023.

County (State)	Population (2022–2023)	P (increasing)	Excess deaths, n	Excess deaths (per 100,000)	Priority score	Smoothed RR in 2022–2023 (95% CI)
Tulsa County, OK	1,359,929	1	848	62	0.974	2.49 (2.37–2.63)
Oklahoma County, OK	1,611,969	1	933	58	0.974	2.46 (2.34–2.58)
Jackson County, MS	291,352	1	287	99	0.97	3.12 (2.85–3.42)
Richmond County, GA	411,758	1	224	54	0.964	2.34 (2.12–2.57)
Anderson County, SC	422,728	1	243	58	0.959	2.17 (1.98–2.37)
Pickens County, SC	268,965	1	164	61	0.957	2.28 (2.05–2.55)
Rogers County, OK	199,308	1	132	66	0.956	2.41 (2.14–2.72)
Le Flore County, OK	98,598	1	100	101	0.952	3.13 (2.71–3.62)
Troup County, GA	140,989	1	90	64	0.951	2.42 (2.10–2.79)
Muskogee County, OK	132,984	1	152	115	0.95	3.49 (3.08–3.95)

Our findings remained robust across a comprehensive set of sensitivity checks. Model diagnostics supported the negative binomial specification and the presence of time-varying disparities (Table S7). Observed-versus-fitted summaries and residual diagnostics did not indicate material lack-of-fit (Table S8 and Figs. S2–S4). Key Q5 vs Q1 estimates were broadly consistent in direction and statistical significance after region adjustment and state fixed effects, and were unchanged under alternative pandemic trend specifications (Tables S9–S10). Bayesian spatiotemporal models incorporating structured random effects substantially improved fit compared with simpler baselines (Table S11), and hotspot detection (Gi* statistics and local Moran’s I) corroborated the spatial patterns observed in the risk mapping (Figs. S5–S6 and Table S12).

## Discussion

In this county-level analysis of US death certificate data from 2014 to 2023, we provide a CKM-informed surveillance view of cardiovascular mortality. Three findings stand out. First, national crude CKM-involved CVD mortality rose from 2014–2015 to a peak in 2020–2021 and remained elevated in 2022–2023, suggesting that the post-2020 increase has not fully receded. Second, the social gradient by SVI persisted across the period and, crucially, disparities widened on both relative and absolute scales – as evidenced by increasing rate ratios, a significant interaction term and rising inequality indices. Third, county-level risk exhibited marked spatial heterogeneity, with clusters of high risk and worsening trends concentrated in specific states. Translating these signals into actionable intelligence, we identified a focused set of ʻTier 1’ counties and quantified benchmark-based excess deaths to guide resource allocation.

A key contribution of this work is the use of multiple-cause mortality to capture the ʻCKM context’ of cardiovascular death. Unlike traditional surveillance that isolates a single underlying cause, our approach acknowledges that diabetes, kidney disease, obesity and hypertension frequently co-occur with fatal cardiovascular events. While a ʻmention’ on the death certificate does not imply strict causal attribution, it represents clinically documented comorbidity proximate to death. Recent multiple-cause-of-death analyses using CDC WONDER similarly leverage co-mentions of cardiometabolic conditions to quantify intersecting mortality burdens, supporting the validity of this surveillance perspective.^22^ The sustained elevation in CKM-involved CVD mortality after 2020 may reflect a convergence of forces – including disruption of chronic disease management, changes in health-seeking behaviour and worsening cardiometabolic risk profiles – operating through well-described pathways linking structural context to chronic disease burden.^23^ This shift in perspective – from ʻHow is CVD mortality changing?’ to ʻHow is CVD mortality changing in the presence of CKM morbidity?’ – aligns directly with the American Heart Association’s recent call for integrated CKM health frameworks.^1^, ^24^

The observed widening of inequality is particularly concerning because it emerged across complementary metrics. The upward drift in relative metrics combined with the rise in absolute burden (SII) suggests that socially vulnerable counties are falling further behind. While prior studies have documented static socioeconomic gradients in CVD outcomes, our findings provide robust statistical evidence that this gap is actively expanding.^25^ Plausible mechanisms include the ʻsyndemic’ nature of CKM conditions in high-SVI areas, where structural determinants intersect with barriers to high-quality primary care.^25^, ^26^ Inequitable access to and uptake of novel pharmacotherapies (eg glucagon-like peptide-1 receptor agonists; sodium–glucose cotransporter 2 inhibitors) may further reinforce these gaps.^27^ These constraints were likely exacerbated during the pandemic, when care disruptions disproportionately affected disadvantaged communities with less reserve capacity.^28^, ^29^

Geography provides an additional layer of interpretability. The concentration of high-risk/worsening counties within specific states (eg Oklahoma, Mississippi) suggests that state-level policy environments (such as Medicaid expansion status), health system capacity and regional risk factor profiles fundamentally shape these outcomes.^30^, ^31^ From a public health perspective, our bivariate classification offers a practical advancement over maps based solely on historical rates. Two counties with similar current burdens may require different strategies if one is stabilising while the other is rapidly deteriorating.^32^, ^33^ The Tier 1 prioritisation framework thus provides a tool for precision public health, guiding the deployment of hypertension control programmes, diabetes prevention services, and integrated CKM care pathways to areas where the ʻneed-trajectory’ profile is most urgent.^32^, ^34^

The benchmark-based excess deaths estimate further translates these abstract risks into a tangible impact metric.^35^ Although ʻexcess’ here denotes a statistical deviation from the reference benchmark rather than direct causality, it highlights the human cost of geographic and social inequities.^36^ The strong heterogeneity in excess mortality across states underscores the importance of tailoring responses to local contexts – particularly in states where the potential return on targeted investment (in terms of avertable deaths) is largest.^37^, ^38^

Several strengths support the credibility of these findings. We improved temporal coherence by aligning SVI exposure to contemporaneous mortality blocks,^39^, ^40^ employed an SMR/offset framework to control for age structure, and implemented Bayesian spatiotemporal smoothing to stabilise estimates in sparsely populated areas.^41^, ^42^ Furthermore, the robustness of our conclusions across multiple sensitivity checks – including alternative pandemic specifications and suppression handling strategies – reinforces the validity of the observed widening disparities.^42^

Limitations should also be considered. First, death certificate coding practices vary by jurisdiction; CKM components may be under-recorded, potentially leading to conservative estimates of CKM involvement. Second, this is an ecological analysis; county-level associations reflect aggregate patterns and should not be interpreted as individual-level causal effects. Third, while SVI captures broad structural vulnerability, it does not isolate specific drivers (eg transportation vs income), which may be actionable targets. Fourth, despite smoothing, small-area estimation relies on modelling assumptions that may influence rankings in the most sparsely populated counties. Finally, disentangling the direct versus indirect effects of the pandemic on post-2020 trends requires complementary clinical data sources. Future work should identify which CKM components drive geographic inequities, and refine targeting via stratification by race/ethnicity and rurality. Linking mortality surveillance to non-fatal outcomes (hospitalisations, prescription fills) and quasi-experimental evaluations of state policies is needed to test interventions for Tier 1 counties.

In summary, CKM component-involved CVD mortality has not only increased nationally in the USA since 2014, but has done so with widening social and geographic divides. By integrating multiple-cause mortality definitions, rigorous inequality metrics and spatiotemporal prioritisation, this study provides an updated, actionable map of where CKM-informed cardiovascular prevention resources are most urgently needed to close the gap.

## CRediT authorship contribution statement

**Kaide Xia:** Conceptualization, Data curation, Formal analysis, Methodology, Visualization, Writing – original draft. **Junwen Wang:** Project administration, Supervision, Validation, Writing – review & editing. **Bingpeng Gao:** Conceptualization, Formal analysis, Methodology, Visualization, Writing – original draft.

## Ethics approval and consent to participate

This study used publicly available, de-identified, aggregated data from the CDC WONDER Multiple Cause of Death database and county-level CDC/ATSDR Social Vulnerability Index data. Institutional review board approval was not required, and informed consent was not applicable.

## Funding

This research received no external funding. No funding source had any role in the study design, data collection, data analysis, data interpretation, writing of the report, or the decision to submit the article for publication.

## Declaration of competing interest

The authors declare that they have no known competing financial interests or personal relationships that could have appeared to influence the work reported in this paper.

## Data Availability

The data for this article are publicly available through the Centers for Disease Control and Prevention Wide-Ranging Online Data for Epidemiologic Research (CDC WONDER) Multiple Cause of Death database and the Agency for Toxic Substances and Disease Registry (ATSDR) Social Vulnerability Index (SVI) data set. All analytic code and derived, nonidentifiable aggregated outputs will be made available from the corresponding author upon reasonable request.

## Footnotes

Appendix A Supplementary data associated with this article can be found in the online version at doi:10.1016/j.clinme.2026.100607.
