# Supplementary material for: Trends and widening inequities in cardiovascular–kidney–metabolic involvement in cardiovascular mortality: A national spatiotemporal analysis, 2014–2023
Source: Clin Med (Lond). 2026 Jun 13;26(5):100607. doi: 10.1016/j.clinme.2026.100607 (PMC13382763; doi:10.1016/j.clinme.2026.100607)
Supplement: Supplementary file 1 — Supplementary material [file mmc1.docx]

**Supplementary Material**

[**Supplementary Tables:** 2](#_Toc219117066)

[Table S1. 2](#_Toc219117067)

[Table S2. 2](#_Toc219117068)

[Table S3. 3](#_Toc219117069)

[Table S4A. 4](#_Toc219117070)

[Table S4B. 7](#_Toc219117071)

[Table S5. 128](#_Toc219117072)

[Table S6. 131](#_Toc219117073)

[Table S7. 133](#_Toc219117074)

[Table S8. 133](#_Toc219117075)

[Table S9. 135](#_Toc219117076)

[Table S10. 135](#_Toc219117077)

[Table S11. 136](#_Toc219117078)

[Table S12. 136](#_Toc219117079)

[**Supplementary Figures:** 160](#_Toc219117080)

[Figure S1. 160](#_Toc219117081)

[Figure S2. 161](#_Toc219117082)

[Figure S3. 161](#_Toc219117083)

[Figure S4. 162](#_Toc219117084)

[Figure S5. 162](#_Toc219117085)

[Figure S6. 163](#_Toc219117086)

# **Supplementary Tables:**

| Table S1. National deaths, population, and crude mortality rates for CKM-involved cardiovascular disease, United States, 2014–2023 | | | |
| --- | --- | --- | --- |
| Period | Deaths | Population | Crude rate (per 100000) |
| 2014-2015 | 232407 | 639061458 | 36.367 |
| 2016-2017 | 252037 | 648129802 | 38.887 |
| 2018-2019 | 266671 | 655026300 | 40.711 |
| 2020-2021 | 315327 | 656331392 | 48.044 |
| 2022-2023 | 316588 | 660898316 | 47.903 |

| Table S2. Observed deaths, expected deaths, and indirectly standardized mortality rates by SVI quintile and biennial period for CKM-involved CVD mortality. | | | | | | | |
| --- | --- | --- | --- | --- | --- | --- | --- |
| Period | SVI quintile | Observed deaths (O) | Fitted deaths (μ) | Expected deaths (E) | Population | Observed SMR (O/E) | Fitted SMR (μ/E) |
| 2014-2015 | Q1 | 24409 | 28950 | 29231 | 77687461 | 0.835 | 0.990 |
| 2014-2015 | Q2 | 42304 | 48834 | 47509 | 126913577 | 0.890 | 1.028 |
| 2014-2015 | Q3 | 56270 | 62427 | 58406 | 155573902 | 0.963 | 1.069 |
| 2014-2015 | Q4 | 66359 | 70820 | 61783 | 176374280 | 1.074 | 1.146 |
| 2014-2015 | Q5 | 43065 | 43072 | 35462 | 102512238 | 1.214 | 1.215 |
| 2016-2017 | Q1 | 27947 | 33544 | 33467 | 83721480 | 0.835 | 1.002 |
| 2016-2017 | Q2 | 46001 | 53536 | 51375 | 128878553 | 0.895 | 1.042 |
| 2016-2017 | Q3 | 62060 | 70347 | 64949 | 161389563 | 0.956 | 1.083 |
| 2016-2017 | Q4 | 66954 | 74110 | 63155 | 167924959 | 1.060 | 1.173 |
| 2016-2017 | Q5 | 49075 | 49152 | 39087 | 106215247 | 1.256 | 1.257 |
| 2018-2019 | Q1 | 30564 | 35910 | 35402 | 83745971 | 0.863 | 1.014 |
| 2018-2019 | Q2 | 50902 | 59814 | 56620 | 136204870 | 0.899 | 1.056 |
| 2018-2019 | Q3 | 62560 | 73132 | 66631 | 157740187 | 0.939 | 1.098 |
| 2018-2019 | Q4 | 75549 | 83615 | 69603 | 177590537 | 1.085 | 1.201 |
| 2018-2019 | Q5 | 47096 | 50081 | 38467 | 99744735 | 1.224 | 1.302 |
| 2020-2021 | Q1 | 29789 | 34439 | 33548 | 65921542 | 0.888 | 1.027 |
| 2020-2021 | Q2 | 52884 | 64667 | 60383 | 123801075 | 0.876 | 1.071 |
| 2020-2021 | Q3 | 57934 | 66463 | 59757 | 120629954 | 0.969 | 1.112 |
| 2020-2021 | Q4 | 83860 | 102501 | 83345 | 170489633 | 1.006 | 1.230 |
| 2020-2021 | Q5 | 90860 | 103402 | 76715 | 175489188 | 1.184 | 1.348 |
| 2022-2023 | Q1 | 31962 | 36820 | 35441 | 70224226 | 0.902 | 1.039 |
| 2022-2023 | Q2 | 49036 | 61638 | 56773 | 115292997 | 0.864 | 1.086 |
| 2022-2023 | Q3 | 63546 | 72376 | 64216 | 131200693 | 0.990 | 1.127 |
| 2022-2023 | Q4 | 81239 | 101594 | 80693 | 168737044 | 1.007 | 1.259 |
| 2022-2023 | Q5 | 90805 | 108414 | 77690 | 175443356 | 1.169 | 1.395 |

| Table S3. Prespecified thresholds and parameters used for bivariate classification (RR_mean and P[increasing)) and prioritization. | |
| --- | --- |
| Parameter | Value |
| RR cut value (top quantile) | 1.770 |
| RR quantile cut | 0.900 |
| P(increasing) threshold | 0.800 |
| Population minimum for prioritization (if used) | 50000 |
| RR bivariate cut (q) | 0.750 |
| RR threshold for bivariate classification (75th percentile of RR_mean in 2022–2023) | 1.432 |

| Table S4A. State distribution of counties classified as “High RR + High P” under the bivariate thresholds (RR ≥ 1.4319; P[increasing] ≥ 0.80). | | | | | | | | |
| --- | --- | --- | --- | --- | --- | --- | --- | --- |
| State FIPS | State | Counties, n | High RR + High P counties, n | Tier 1 counties, n | Population in High RR + High P counties | Population in Tier 1 counties | High RR + High P, % of counties | Tier 1, % of counties |
| 40 | OK | 77 | 50 | 27 | 7039516 | 6473016 | 64.9 | 35.1 |
| 28 | MS | 82 | 33 | 9 | 2221932 | 987800 | 40.2 | 11 |
| 48 | TX | 254 | 33 | 3 | 2526586 | 619420 | 13 | 1.2 |
| 13 | GA | 159 | 28 | 6 | 3569610 | 1149933 | 17.6 | 3.8 |
| 21 | KY | 120 | 26 | 6 | 2008881 | 806220 | 21.7 | 5 |
| 47 | TN | 95 | 17 | 8 | 1530643 | 940689 | 17.9 | 8.4 |
| 45 | SC | 46 | 17 | 7 | 3609298 | 1985207 | 37 | 15.2 |
| 19 | IA | 99 | 16 | 1 | 1326280 | 79835 | 16.2 | 1 |
| 55 | WI | 72 | 13 | 1 | 2130494 | 84072 | 18.1 | 1.4 |
| 22 | LA | 64 | 11 | 6 | 898414 | 715225 | 17.2 | 9.4 |
| 27 | MN | 87 | 11 | 0 | 471072 | 0 | 12.6 | 0 |
| 17 | IL | 102 | 8 | 1 | 393898 | 70837 | 7.8 | 1 |
| 18 | IN | 92 | 7 | 2 | 523675 | 204289 | 7.6 | 2.2 |
| 5 | AR | 75 | 7 | 0 | 660676 | 0 | 9.3 | 0 |
| 54 | WV | 55 | 7 | 0 | 537169 | 0 | 12.7 | 0 |
| 41 | OR | 36 | 6 | 2 | 1508637 | 486843 | 16.7 | 5.6 |
| 37 | NC | 100 | 6 | 1 | 664154 | 233831 | 6 | 1 |
| 31 | NE | 93 | 6 | 0 | 86448 | 0 | 6.5 | 0 |
| 51 | VA | 133 | 6 | 0 | 449856 | 0 | 4.5 | 0 |
| 8 | CO | 64 | 5 | 0 | 560638 | 0 | 7.8 | 0 |
| 24 | MD | 24 | 4 | 1 | 1383865 | 1134346 | 16.7 | 4.2 |
| 16 | ID | 44 | 4 | 0 | 768366 | 0 | 9.1 | 0 |
| 20 | KS | 105 | 3 | 1 | 136900 | 70576 | 2.9 | 1 |
| 53 | WA | 39 | 3 | 1 | 824487 | 224928 | 7.7 | 2.6 |
| 12 | FL | 67 | 3 | 0 | 182139 | 0 | 4.5 | 0 |
| 29 | MO | 115 | 3 | 0 | 682191 | 0 | 2.6 | 0 |
| 30 | MT | 56 | 3 | 0 | 58587 | 0 | 5.4 | 0 |
| 32 | NV | 17 | 3 | 0 | 56864 | 0 | 17.6 | 0 |
| 39 | OH | 88 | 3 | 0 | 157102 | 0 | 3.4 | 0 |
| 42 | PA | 67 | 2 | 1 | 129551 | 68886 | 3 | 1.5 |
| 26 | MI | 83 | 2 | 0 | 226343 | 0 | 2.4 | 0 |
| 36 | NY | 62 | 2 | 0 | 193674 | 0 | 3.2 | 0 |
| 56 | WY | 23 | 2 | 0 | 37563 | 0 | 8.7 | 0 |
| 50 | VT | 14 | 1 | 1 | 101713 | 101713 | 7.1 | 7.1 |
| 2 | AK | 27 | 1 | 0 | 2187 | 0 | 3.7 | 0 |
| 6 | CA | 58 | 1 | 0 | 2032521 | 0 | 1.7 | 0 |
| 10 | DE | 3 | 1 | 0 | 377547 | 0 | 33.3 | 0 |
| 11 | DC | 1 | 1 | 0 | 1349921 | 0 | 100 | 0 |
| 35 | NM | 33 | 1 | 0 | 127453 | 0 | 3 | 0 |
| 1 | AL | 67 | 0 | 0 | 0 | 0 | 0 | 0 |
| 4 | AZ | 15 | 0 | 0 | 0 | 0 | 0 | 0 |
| 15 | HI | 5 | 0 | 0 | 0 | 0 | 0 | 0 |
| 23 | ME | 16 | 0 | 0 | 0 | 0 | 0 | 0 |
| 25 | MA | 14 | 0 | 0 | 0 | 0 | 0 | 0 |
| 33 | NH | 10 | 0 | 0 | 0 | 0 | 0 | 0 |
| 34 | NJ | 21 | 0 | 0 | 0 | 0 | 0 | 0 |
| 38 | ND | 53 | 0 | 0 | 0 | 0 | 0 | 0 |
| 44 | RI | 5 | 0 | 0 | 0 | 0 | 0 | 0 |
| 46 | SD | 65 | 0 | 0 | 0 | 0 | 0 | 0 |
| 49 | UT | 29 | 0 | 0 | 0 | 0 | 0 | 0 |

|  | | | | | | |  |
| --- | --- | --- | --- | --- | --- | --- | --- |
|  | | | | | | |  |
| Table S4B. County-level smoothed RR, P(increasing), bivariate class, and Tier 1 indicator in 2022–2023. | | | | | | |  |
| County FIPS | County (State) | Smoothed RR in 2022–2023 (RR_mean) | P(increasing) | Bivariate class | Tier 1 indicator (Yes/No) | Priority score | Population (2022–2023) |
| 40143 | Tulsa County, OK | 2.495 | 1 | High RR + High P | Yes | 0.974 | 1359929 |
| 40109 | Oklahoma County, OK | 2.458 | 1 | High RR + High P | Yes | 0.974 | 1611969 |
| 28059 | Jackson County, MS | 3.124 | 1 | High RR + High P | Yes | 0.97 | 291352 |
| 13245 | Richmond County, GA | 2.336 | 1 | High RR + High P | Yes | 0.963 | 411758 |
| 45007 | Anderson County, SC | 2.169 | 1 | High RR + High P | Yes | 0.959 | 422728 |
| 45077 | Pickens County, SC | 2.285 | 1 | High RR + High P | Yes | 0.957 | 268965 |
| 40131 | Rogers County, OK | 2.41 | 1 | High RR + High P | Yes | 0.956 | 199308 |
| 40079 | Le Flore County, OK | 3.133 | 1 | High RR + High P | Yes | 0.952 | 98598 |
| 13285 | Troup County, GA | 2.422 | 1 | High RR + High P | Yes | 0.951 | 140989 |
| 40101 | Muskogee County, OK | 3.487 | 1 | High RR + High P | Yes | 0.95 | 132984 |
| 40125 | Pottawatomie County, OK | 2.761 | 1 | High RR + High P | Yes | 0.949 | 147142 |
| 40147 | Washington County, OK | 2.566 | 1 | High RR + High P | Yes | 0.949 | 106944 |
| 28081 | Lee County, MS | 2.522 | 1 | High RR + High P | Yes | 0.948 | 165623 |
| 40027 | Cleveland County, OK | 1.885 | 1 | High RR + High P | Yes | 0.947 | 600677 |
| 45083 | Spartanburg County, SC | 1.868 | 1 | High RR + High P | Yes | 0.946 | 702646 |
| 24510 | Baltimore city, MD | 1.871 | 1 | High RR + High P | Yes | 0.945 | 1134346 |
| 40031 | Comanche County, OK | 2.728 | 0.98 | High RR + High P | Yes | 0.944 | 243799 |
| 40121 | Pittsburg County, OK | 3.18 | 1 | High RR + High P | Yes | 0.941 | 86955 |
| 22069 | Natchitoches Parish, LA | 2.909 | 1 | High RR + High P | Yes | 0.938 | 72939 |
| 40017 | Canadian County, OK | 1.885 | 1 | High RR + High P | Yes | 0.937 | 345083 |
| 40041 | Delaware County, OK | 2.511 | 1 | High RR + High P | Yes | 0.937 | 83183 |
| 45041 | Florence County, SC | 1.849 | 1 | High RR + High P | Yes | 0.936 | 273954 |
| 22105 | Tangipahoa Parish, LA | 1.845 | 1 | High RR + High P | Yes | 0.935 | 274863 |
| 40037 | Creek County, OK | 2.566 | 0.98 | High RR + High P | Yes | 0.935 | 146018 |
| 45085 | Sumter County, SC | 1.879 | 1 | High RR + High P | Yes | 0.935 | 208101 |
| 28067 | Jones County, MS | 1.971 | 1 | High RR + High P | Yes | 0.934 | 132690 |
| 47163 | Sullivan County, TN | 1.811 | 1 | High RR + High P | Yes | 0.933 | 322997 |
| 40115 | Ottawa County, OK | 3.261 | 1 | High RR + High P | Yes | 0.931 | 60616 |
| 21227 | Warren County, KY | 1.804 | 1 | High RR + High P | Yes | 0.931 | 282275 |
| 40013 | Bryan County, OK | 2.23 | 1 | High RR + High P | Yes | 0.931 | 97005 |
| 48441 | Taylor County, TX | 1.833 | 1 | High RR + High P | Yes | 0.931 | 291983 |
| 28117 | Prentiss County, MS | 2.789 | 1 | High RR + High P | Yes | 0.93 | 50026 |
| 40111 | Okmulgee County, OK | 3.051 | 0.99 | High RR + High P | Yes | 0.93 | 74046 |
| 18177 | Wayne County, IN | 2.033 | 1 | High RR + High P | Yes | 0.927 | 132343 |
| 13277 | Tift County, GA | 2.043 | 1 | High RR + High P | Yes | 0.926 | 82889 |
| 40047 | Garfield County, OK | 2.187 | 0.99 | High RR + High P | Yes | 0.925 | 123974 |
| 28145 | Union County, MS | 2.634 | 1 | High RR + High P | Yes | 0.925 | 56416 |
| 22101 | St. Mary Parish, LA | 1.927 | 1 | High RR + High P | Yes | 0.925 | 94874 |
| 21037 | Campbell County, KY | 1.83 | 1 | High RR + High P | Yes | 0.924 | 186974 |
| 45087 | Union County, SC | 2.495 | 1 | High RR + High P | Yes | 0.921 | 53368 |
| 17105 | Livingston County, IL | 2.016 | 1 | High RR + High P | Yes | 0.921 | 70837 |
| 13215 | Muscogee County, GA | 2.055 | 0.96 | High RR + High P | Yes | 0.919 | 404309 |
| 41019 | Douglas County, OR | 1.79 | 1 | High RR + High P | Yes | 0.919 | 224725 |
| 40089 | McCurtain County, OK | 2.904 | 0.98 | High RR + High P | Yes | 0.916 | 61433 |
| 45033 | Dillon County, SC | 2.033 | 1 | High RR + High P | Yes | 0.915 | 55445 |
| 21193 | Perry County, KY | 2.545 | 0.99 | High RR + High P | Yes | 0.914 | 54500 |
| 40119 | Payne County, OK | 1.896 | 0.98 | High RR + High P | Yes | 0.913 | 166053 |
| 41043 | Linn County, OR | 1.792 | 0.99 | High RR + High P | Yes | 0.913 | 262118 |
| 40049 | Garvin County, OK | 2.4 | 0.99 | High RR + High P | Yes | 0.912 | 51636 |
| 28083 | Leflore County, MS | 4.417 | 0.98 | High RR + High P | Yes | 0.911 | 53042 |
| 42059 | Greene County, PA | 1.977 | 0.99 | High RR + High P | Yes | 0.907 | 68886 |
| 21195 | Pike County, KY | 2.016 | 0.97 | High RR + High P | Yes | 0.905 | 112290 |
| 47017 | Carroll County, TN | 2.355 | 0.98 | High RR + High P | Yes | 0.904 | 57278 |
| 18083 | Knox County, IN | 2.322 | 0.97 | High RR + High P | Yes | 0.903 | 71946 |
| 47025 | Claiborne County, TN | 1.96 | 0.99 | High RR + High P | Yes | 0.902 | 65011 |
| 13035 | Butts County, GA | 2.021 | 0.99 | High RR + High P | Yes | 0.9 | 53632 |
| 22115 | Vernon Parish, LA | 2.068 | 0.97 | High RR + High P | Yes | 0.899 | 93120 |
| 40145 | Wagoner County, OK | 2.112 | 0.94 | High RR + High P | Yes | 0.898 | 175977 |
| 40097 | Mayes County, OK | 2.748 | 0.94 | High RR + High P | Yes | 0.896 | 79459 |
| 40071 | Kay County, OK | 2.244 | 0.95 | High RR + High P | Yes | 0.894 | 87379 |
| 47131 | Obion County, TN | 1.963 | 0.98 | High RR + High P | Yes | 0.893 | 60785 |
| 55075 | Marinette County, WI | 2.128 | 0.96 | High RR + High P | Yes | 0.891 | 84072 |
| 22009 | Avoyelles Parish, LA | 2.952 | 0.93 | High RR + High P | Yes | 0.889 | 77123 |
| 28095 | Monroe County, MS | 2.364 | 0.95 | High RR + High P | Yes | 0.889 | 67177 |
| 28137 | Tate County, MS | 3.322 | 0.93 | High RR + High P | Yes | 0.886 | 56520 |
| 37155 | Robeson County, NC | 1.938 | 0.92 | High RR + High P | Yes | 0.883 | 233831 |
| 40137 | Stephens County, OK | 2.635 | 0.91 | High RR + High P | Yes | 0.881 | 87630 |
| 21019 | Boyd County, KY | 2.531 | 0.91 | High RR + High P | Yes | 0.88 | 95885 |
| 40113 | Osage County, OK | 2.155 | 0.93 | High RR + High P | Yes | 0.879 | 91982 |
| 20061 | Geary County, KS | 2.801 | 0.92 | High RR + High P | Yes | 0.879 | 70576 |
| 48497 | Wise County, TX | 1.844 | 0.94 | High RR + High P | Yes | 0.879 | 152994 |
| 21049 | Clark County, KY | 1.816 | 0.97 | High RR + High P | Yes | 0.875 | 74296 |
| 40019 | Carter County, OK | 2.594 | 0.9 | High RR + High P | Yes | 0.875 | 96932 |
| 47003 | Bedford County, TN | 2.432 | 0.9 | High RR + High P | Yes | 0.874 | 105019 |
| 22099 | St. Martin Parish, LA | 2.314 | 0.89 | High RR + High P | Yes | 0.864 | 102306 |
| 47053 | Gibson County, TN | 2.19 | 0.89 | High RR + High P | Yes | 0.864 | 101871 |
| 13293 | Upson County, GA | 2.108 | 0.92 | High RR + High P | Yes | 0.86 | 56356 |
| 28087 | Lowndes County, MS | 2.154 | 0.86 | High RR + High P | Yes | 0.853 | 114954 |
| 48005 | Angelina County, TX | 2.102 | 0.85 | High RR + High P | Yes | 0.851 | 174443 |
| 50011 | Franklin County, VT | 1.931 | 0.89 | High RR + High P | Yes | 0.85 | 101713 |
| 40039 | Custer County, OK | 2.137 | 0.88 | High RR + High P | Yes | 0.84 | 56305 |
| 53015 | Cowlitz County, WA | 1.776 | 0.87 | High RR + High P | Yes | 0.839 | 224928 |
| 47145 | Roane County, TN | 1.908 | 0.84 | High RR + High P | Yes | 0.829 | 111225 |
| 47073 | Hawkins County, TN | 1.869 | 0.84 | High RR + High P | Yes | 0.828 | 116503 |
| 19127 | Marshall County, IA | 2.024 | 0.82 | High RR + High P | Yes | 0.816 | 79835 |
| 13063 | Clayton County, GA | 1.759 | 1 | High RR + High P | No | 0.933 | 596699 |
| 45079 | Richland County, SC | 1.726 | 1 | High RR + High P | No | 0.928 | 846664 |
| 10001 | Kent County, DE | 1.754 | 1 | High RR + High P | No | 0.928 | 377547 |
| 22077 | Pointe Coupee Parish, LA | 3.141 | 1 | High RR + High P | No | 0.927 | 40212 |
| 8077 | Mesa County, CO | 1.759 | 1 | High RR + High P | No | 0.927 | 318212 |
| 40009 | Beckham County, OK | 2.606 | 1 | High RR + High P | No | 0.921 | 44018 |
| 55063 | La Crosse County, WI | 1.759 | 1 | High RR + High P | No | 0.919 | 240738 |
| 28017 | Chickasaw County, MS | 2.798 | 1 | High RR + High P | No | 0.919 | 33729 |
| 55009 | Brown County, WI | 1.678 | 1 | High RR + High P | No | 0.911 | 541485 |
| 41039 | Lane County, OR | 1.609 | 1 | High RR + High P | No | 0.911 | 763362 |
| 19163 | Scott County, IA | 1.644 | 1 | High RR + High P | No | 0.911 | 348021 |
| 28091 | Marion County, MS | 2.03 | 1 | High RR + High P | No | 0.91 | 48390 |
| 19013 | Black Hawk County, IA | 1.756 | 0.99 | High RR + High P | No | 0.905 | 260523 |
| 53005 | Benton County, WA | 1.588 | 1 | High RR + High P | No | 0.902 | 428048 |
| 13115 | Floyd County, GA | 1.633 | 1 | High RR + High P | No | 0.901 | 199550 |
| 19065 | Fayette County, IA | 2.118 | 1 | High RR + High P | No | 0.9 | 38476 |
| 48459 | Upshur County, TX | 1.757 | 1 | High RR + High P | No | 0.899 | 85735 |
| 5085 | Lonoke County, AR | 1.642 | 1 | High RR + High P | No | 0.899 | 151143 |
| 40001 | Adair County, OK | 2.849 | 0.98 | High RR + High P | No | 0.899 | 39143 |
| 45059 | Laurens County, SC | 1.642 | 1 | High RR + High P | No | 0.898 | 136824 |
| 13189 | McDuffie County, GA | 2.422 | 0.98 | High RR + High P | No | 0.896 | 43489 |
| 6019 | Fresno County, CA | 1.528 | 1 | High RR + High P | No | 0.889 | 2032521 |
| 28109 | Pearl River County, MS | 1.613 | 1 | High RR + High P | No | 0.889 | 115161 |
| 28133 | Sunflower County, MS | 1.981 | 0.98 | High RR + High P | No | 0.888 | 49305 |
| 5139 | Union County, AR | 1.714 | 1 | High RR + High P | No | 0.888 | 75210 |
| 28051 | Holmes County, MS | 2.52 | 0.98 | High RR + High P | No | 0.888 | 31888 |
| 28025 | Clay County, MS | 2.435 | 0.97 | High RR + High P | No | 0.887 | 36517 |
| 21059 | Daviess County, KY | 1.56 | 1 | High RR + High P | No | 0.887 | 206647 |
| 5031 | Craighead County, AR | 1.661 | 0.98 | High RR + High P | No | 0.887 | 226856 |
| 28031 | Covington County, MS | 2.57 | 0.97 | High RR + High P | No | 0.885 | 36201 |
| 54011 | Cabell County, WV | 1.559 | 1 | High RR + High P | No | 0.885 | 184567 |
| 40117 | Pawnee County, OK | 3.366 | 0.96 | High RR + High P | No | 0.885 | 31545 |
| 48183 | Gregg County, TX | 1.537 | 1 | High RR + High P | No | 0.884 | 251802 |
| 13275 | Thomas County, GA | 1.73 | 0.99 | High RR + High P | No | 0.883 | 91200 |
| 21183 | Ohio County, KY | 1.921 | 0.98 | High RR + High P | No | 0.883 | 47122 |
| 48457 | Tyler County, TX | 1.79 | 1 | High RR + High P | No | 0.882 | 40793 |
| 55073 | Marathon County, WI | 1.524 | 1 | High RR + High P | No | 0.881 | 276567 |
| 37191 | Wayne County, NC | 1.706 | 0.97 | High RR + High P | No | 0.881 | 236135 |
| 13199 | Meriwether County, GA | 1.893 | 0.99 | High RR + High P | No | 0.881 | 41821 |
| 28139 | Tippah County, MS | 1.798 | 1 | High RR + High P | No | 0.879 | 42725 |
| 47099 | Lawrence County, TN | 1.704 | 0.99 | High RR + High P | No | 0.879 | 91616 |
| 40105 | Nowata County, OK | 3.845 | 0.97 | High RR + High P | No | 0.878 | 18851 |
| 37173 | Swain County, NC | 2.168 | 0.98 | High RR + High P | No | 0.877 | 27852 |
| 21101 | Henderson County, KY | 1.739 | 0.98 | High RR + High P | No | 0.876 | 88151 |
| 5057 | Hempstead County, AR | 1.902 | 0.98 | High RR + High P | No | 0.875 | 38824 |
| 28071 | Lafayette County, MS | 1.695 | 0.98 | High RR + High P | No | 0.874 | 116197 |
| 16027 | Canyon County, ID | 1.543 | 0.99 | High RR + High P | No | 0.874 | 508566 |
| 13305 | Wayne County, GA | 1.767 | 0.98 | High RR + High P | No | 0.872 | 62110 |
| 28141 | Tishomingo County, MS | 2.271 | 0.95 | High RR + High P | No | 0.872 | 37128 |
| 13181 | Lincoln County, GA | 1.919 | 1 | High RR + High P | No | 0.871 | 15738 |
| 13071 | Colquitt County, GA | 1.732 | 0.97 | High RR + High P | No | 0.87 | 91997 |
| 40127 | Pushmataha County, OK | 3.072 | 0.96 | High RR + High P | No | 0.869 | 21509 |
| 17055 | Franklin County, IL | 1.702 | 0.98 | High RR + High P | No | 0.868 | 74353 |
| 13179 | Liberty County, GA | 1.505 | 1 | High RR + High P | No | 0.868 | 137129 |
| 24039 | Somerset County, MD | 1.698 | 0.99 | High RR + High P | No | 0.867 | 49518 |
| 13151 | Henry County, GA | 1.435 | 1 | High RR + High P | No | 0.867 | 503085 |
| 44007 | Providence County, RI | 1.412 | 1 | Low RR + High P | No | 0.866 | 1318039 |
| 48347 | Nacogdoches County, TX | 1.562 | 0.99 | High RR + High P | No | 0.865 | 130369 |
| 55105 | Rock County, WI | 1.46 | 1 | High RR + High P | No | 0.865 | 328119 |
| 13299 | Ware County, GA | 1.754 | 0.96 | High RR + High P | No | 0.861 | 71840 |
| 39073 | Hocking County, OH | 1.731 | 0.98 | High RR + High P | No | 0.861 | 55358 |
| 19155 | Pottawattamie County, IA | 1.477 | 1 | High RR + High P | No | 0.861 | 186244 |
| 27047 | Freeborn County, MN | 1.656 | 0.99 | High RR + High P | No | 0.861 | 61198 |
| 48217 | Hill County, TX | 1.555 | 1 | High RR + High P | No | 0.861 | 75367 |
| 19045 | Clinton County, IA | 1.557 | 0.99 | High RR + High P | No | 0.859 | 92413 |
| 13051 | Chatham County, GA | 1.403 | 1 | Low RR + High P | No | 0.858 | 604680 |
| 45015 | Berkeley County, SC | 1.406 | 1 | Low RR + High P | No | 0.858 | 500169 |
| 45047 | Greenwood County, SC | 1.497 | 1 | High RR + High P | No | 0.858 | 138683 |
| 48073 | Cherokee County, TX | 1.639 | 0.97 | High RR + High P | No | 0.858 | 103649 |
| 30035 | Glacier County, MT | 2.163 | 0.95 | High RR + High P | No | 0.857 | 27259 |
| 19057 | Des Moines County, IA | 1.759 | 0.95 | High RR + High P | No | 0.857 | 76495 |
| 36035 | Fulton County, NY | 1.75 | 0.93 | High RR + High P | No | 0.856 | 104490 |
| 41035 | Klamath County, OR | 1.768 | 0.92 | High RR + High P | No | 0.856 | 140193 |
| 17121 | Marion County, IL | 1.769 | 0.94 | High RR + High P | No | 0.856 | 73578 |
| 24001 | Allegany County, MD | 1.666 | 0.94 | High RR + High P | No | 0.854 | 134539 |
| 35005 | Chaves County, NM | 1.501 | 1 | High RR + High P | No | 0.854 | 127453 |
| 27113 | Pennington County, MN | 1.722 | 1 | High RR + High P | No | 0.853 | 27530 |
| 28013 | Calhoun County, MS | 2.977 | 0.92 | High RR + High P | No | 0.852 | 25531 |
| 13321 | Worth County, GA | 1.572 | 1 | High RR + High P | No | 0.851 | 40704 |
| 40107 | Okfuskee County, OK | 3.452 | 0.92 | High RR + High P | No | 0.85 | 22527 |
| 28065 | Jefferson Davis County, MS | 2.055 | 0.96 | High RR + High P | No | 0.849 | 22048 |
| 13267 | Tattnall County, GA | 1.786 | 0.95 | High RR + High P | No | 0.849 | 48284 |
| 21121 | Knox County, KY | 1.72 | 0.96 | High RR + High P | No | 0.849 | 59567 |
| 31109 | Lancaster County, NE | 1.369 | 1 | Low RR + High P | No | 0.848 | 651150 |
| 48471 | Walker County, TX | 1.432 | 1 | High RR + High P | No | 0.848 | 160946 |
| 55071 | Manitowoc County, WI | 1.438 | 1 | High RR + High P | No | 0.847 | 162529 |
| 22127 | Winn Parish, LA | 3.21 | 0.91 | High RR + High P | No | 0.847 | 26467 |
| 27055 | Houston County, MN | 1.74 | 0.97 | High RR + High P | No | 0.846 | 37309 |
| 51690 | Martinsville city, VA | 2.832 | 0.91 | High RR + High P | No | 0.846 | 27460 |
| 45037 | Edgefield County, SC | 1.595 | 0.98 | High RR + High P | No | 0.845 | 54566 |
| 47183 | Weakley County, TN | 1.532 | 0.99 | High RR + High P | No | 0.845 | 66127 |
| 16083 | Twin Falls County, ID | 1.423 | 1 | Low RR + High P | No | 0.844 | 188977 |
| 55115 | Shawano County, WI | 1.451 | 1 | High RR + High P | No | 0.844 | 82096 |
| 30105 | Valley County, MT | 2.013 | 0.96 | High RR + High P | No | 0.844 | 15016 |
| 55021 | Columbia County, WI | 1.585 | 0.96 | High RR + High P | No | 0.844 | 116319 |
| 16009 | Benewah County, ID | 1.961 | 0.95 | High RR + High P | No | 0.843 | 20660 |
| 13029 | Bryan County, GA | 1.455 | 1 | High RR + High P | No | 0.842 | 97957 |
| 24033 | Prince George's County, MD | 1.358 | 1 | Low RR + High P | No | 0.842 | 1894410 |
| 28085 | Lincoln County, MS | 1.559 | 0.98 | High RR + High P | No | 0.841 | 69466 |
| 20095 | Kingman County, KS | 2.236 | 0.94 | High RR + High P | No | 0.84 | 14240 |
| 55081 | Monroe County, WI | 1.461 | 1 | High RR + High P | No | 0.839 | 92045 |
| 13107 | Emanuel County, GA | 2.046 | 0.9 | High RR + High P | No | 0.839 | 46065 |
| 21237 | Wolfe County, KY | 2.329 | 0.94 | High RR + High P | No | 0.838 | 12698 |
| 45055 | Kershaw County, SC | 1.392 | 1 | Low RR + High P | No | 0.837 | 137755 |
| 48167 | Galveston County, TX | 1.358 | 1 | Low RR + High P | No | 0.837 | 719131 |
| 54077 | Preston County, WV | 1.507 | 0.99 | High RR + High P | No | 0.837 | 68321 |
| 40153 | Woodward County, OK | 1.91 | 0.92 | High RR + High P | No | 0.836 | 39962 |
| 18037 | Dubois County, IN | 1.594 | 0.95 | High RR + High P | No | 0.836 | 87038 |
| 26121 | Muskegon County, MI | 1.377 | 1 | Low RR + High P | No | 0.835 | 352103 |
| 28079 | Leake County, MS | 2.123 | 0.89 | High RR + High P | No | 0.835 | 42461 |
| 47063 | Hamblen County, TN | 1.522 | 0.96 | High RR + High P | No | 0.834 | 131383 |
| 13147 | Hart County, GA | 1.609 | 0.96 | High RR + High P | No | 0.834 | 54442 |
| 40061 | Haskell County, OK | 2.656 | 0.9 | High RR + High P | No | 0.834 | 23499 |
| 39053 | Gallia County, OH | 1.715 | 0.94 | High RR + High P | No | 0.834 | 58016 |
| 13103 | Effingham County, GA | 1.49 | 0.97 | High RR + High P | No | 0.834 | 140575 |
| 48231 | Hunt County, TX | 1.425 | 0.98 | Low RR + High P | No | 0.832 | 221785 |
| 21001 | Adair County, KY | 1.593 | 0.98 | High RR + High P | No | 0.832 | 38361 |
| 28069 | Kemper County, MS | 1.713 | 0.98 | High RR + High P | No | 0.831 | 17254 |
| 48507 | Zavala County, TX | 2.093 | 0.93 | High RR + High P | No | 0.83 | 18696 |
| 13193 | Macon County, GA | 1.657 | 0.98 | High RR + High P | No | 0.83 | 23594 |
| 8085 | Montrose County, CO | 1.576 | 0.95 | High RR + High P | No | 0.83 | 87963 |
| 28157 | Wilkinson County, MS | 1.99 | 0.94 | High RR + High P | No | 0.829 | 16261 |
| 40043 | Dewey County, OK | 2.944 | 0.92 | High RR + High P | No | 0.829 | 8690 |
| 28115 | Pontotoc County, MS | 1.495 | 0.99 | High RR + High P | No | 0.829 | 62902 |
| 40103 | Noble County, OK | 2.336 | 0.9 | High RR + High P | No | 0.829 | 21677 |
| 5113 | Polk County, AR | 1.544 | 0.99 | High RR + High P | No | 0.827 | 38770 |
| 40023 | Choctaw County, OK | 2.301 | 0.88 | High RR + High P | No | 0.827 | 28683 |
| 51155 | Pulaski County, VA | 1.498 | 0.98 | High RR + High P | No | 0.826 | 67338 |
| 28121 | Rankin County, MS | 1.323 | 1 | Low RR + High P | No | 0.825 | 319395 |
| 45075 | Orangeburg County, SC | 1.551 | 0.93 | High RR + High P | No | 0.824 | 165921 |
| 40073 | Kingfisher County, OK | 1.801 | 0.93 | High RR + High P | No | 0.824 | 30709 |
| 30087 | Rosebud County, MT | 1.741 | 0.97 | High RR + High P | No | 0.824 | 16312 |
| 42085 | Mercer County, PA | 1.352 | 1 | Low RR + High P | No | 0.823 | 217640 |
| 40035 | Craig County, OK | 3.144 | 0.85 | High RR + High P | No | 0.823 | 28670 |
| 27083 | Lyon County, MN | 1.533 | 0.98 | High RR + High P | No | 0.822 | 50860 |
| 29083 | Henry County, MO | 1.609 | 0.95 | High RR + High P | No | 0.822 | 44906 |
| 48463 | Uvalde County, TX | 1.675 | 0.93 | High RR + High P | No | 0.822 | 49850 |
| 8101 | Pueblo County, CO | 1.322 | 1 | Low RR + High P | No | 0.821 | 338894 |
| 45089 | Williamsburg County, SC | 1.723 | 0.91 | High RR + High P | No | 0.821 | 60004 |
| 51139 | Page County, VA | 1.626 | 0.94 | High RR + High P | No | 0.82 | 47518 |
| 11001 | District of Columbia, DC | 1.494 | 0.91 | High RR + High P | No | 0.82 | 1349921 |
| 55139 | Winnebago County, WI | 1.327 | 1 | Low RR + High P | No | 0.82 | 342423 |
| 47033 | Crockett County, TN | 1.585 | 0.98 | High RR + High P | No | 0.82 | 27844 |
| 13185 | Lowndes County, GA | 1.318 | 1 | Low RR + High P | No | 0.82 | 240211 |
| 24045 | Wicomico County, MD | 1.355 | 0.99 | Low RR + High P | No | 0.819 | 209286 |
| 24015 | Cecil County, MD | 1.418 | 0.96 | Low RR + High P | No | 0.819 | 210542 |
| 48061 | Cameron County, TX | 1.282 | 1 | Low RR + High P | No | 0.819 | 851621 |
| 40151 | Woods County, OK | 1.807 | 0.94 | High RR + High P | No | 0.819 | 17166 |
| 21017 | Bourbon County, KY | 2.146 | 0.85 | High RR + High P | No | 0.819 | 40243 |
| 54043 | Lincoln County, WV | 1.838 | 0.9 | High RR + High P | No | 0.818 | 39552 |
| 13073 | Columbia County, GA | 1.304 | 1 | Low RR + High P | No | 0.818 | 327633 |
| 21111 | Jefferson County, KY | 1.272 | 1 | Low RR + High P | No | 0.818 | 1545239 |
| 39105 | Meigs County, OH | 1.735 | 0.92 | High RR + High P | No | 0.818 | 43728 |
| 22019 | Calcasieu Parish, LA | 2.104 | 0.76 | High RR + Low P | No | 0.818 | 406049 |
| 41037 | Lake County, OR | 1.745 | 0.96 | High RR + High P | No | 0.817 | 16666 |
| 13001 | Appling County, GA | 1.906 | 0.89 | High RR + High P | No | 0.817 | 36894 |
| 45035 | Dorchester County, SC | 1.399 | 0.96 | Low RR + High P | No | 0.817 | 335883 |
| 1113 | Russell County, AL | 1.366 | 1 | Low RR + High P | No | 0.817 | 117338 |
| 21067 | Fayette County, KY | 1.408 | 0.95 | Low RR + High P | No | 0.817 | 640435 |
| 45045 | Greenville County, SC | 1.272 | 1 | Low RR + High P | No | 0.817 | 1105881 |
| 19063 | Emmet County, IA | 1.709 | 0.96 | High RR + High P | No | 0.817 | 18443 |
| 47155 | Sevier County, TN | 1.962 | 0.79 | High RR + Low P | No | 0.816 | 198221 |
| 18027 | Daviess County, IN | 1.675 | 0.9 | High RR + High P | No | 0.815 | 67152 |
| 31095 | Jefferson County, NE | 1.697 | 0.97 | High RR + High P | No | 0.815 | 14194 |
| 41041 | Lincoln County, OR | 1.458 | 0.96 | High RR + High P | No | 0.814 | 101573 |
| 54033 | Harrison County, WV | 1.545 | 0.92 | High RR + High P | No | 0.814 | 129391 |
| 36025 | Delaware County, NY | 1.458 | 0.96 | High RR + High P | No | 0.814 | 89184 |
| 24037 | St. Mary's County, MD | 1.371 | 0.98 | Low RR + High P | No | 0.814 | 230064 |
| 17081 | Jefferson County, IL | 1.478 | 0.97 | High RR + High P | No | 0.813 | 72739 |
| 21207 | Russell County, KY | 2.296 | 0.84 | High RR + High P | No | 0.813 | 36464 |
| 21093 | Hardin County, KY | 1.51 | 0.92 | High RR + High P | No | 0.813 | 224016 |
| 21025 | Breathitt County, KY | 2.494 | 0.84 | High RR + High P | No | 0.813 | 26311 |
| 26113 | Missaukee County, MI | 1.849 | 0.9 | High RR + High P | No | 0.812 | 30543 |
| 51740 | Portsmouth city, VA | 1.489 | 0.93 | High RR + High P | No | 0.812 | 193747 |
| 44003 | Kent County, RI | 1.312 | 1 | Low RR + High P | No | 0.811 | 342412 |
| 32023 | Nye County, NV | 1.339 | 1 | Low RR + High P | No | 0.811 | 110489 |
| 28077 | Lawrence County, MS | 1.53 | 0.99 | High RR + High P | No | 0.81 | 23502 |
| 45039 | Fairfield County, SC | 1.721 | 0.91 | High RR + High P | No | 0.809 | 40872 |
| 32001 | Churchill County, NV | 1.597 | 0.93 | High RR + High P | No | 0.809 | 51601 |
| 42047 | Elk County, PA | 1.552 | 0.94 | High RR + High P | No | 0.809 | 60665 |
| 20177 | Shawnee County, KS | 1.323 | 0.99 | Low RR + High P | No | 0.809 | 355283 |
| 13015 | Bartow County, GA | 1.365 | 0.97 | Low RR + High P | No | 0.809 | 227856 |
| 28033 | DeSoto County, MS | 1.27 | 1 | Low RR + High P | No | 0.808 | 384391 |
| 54099 | Wayne County, WV | 1.514 | 0.94 | High RR + High P | No | 0.808 | 75666 |
| 28093 | Marshall County, MS | 1.437 | 0.97 | High RR + High P | No | 0.808 | 68028 |
| 40065 | Jackson County, OK | 1.908 | 0.84 | High RR + High P | No | 0.807 | 49328 |
| 10003 | New Castle County, DE | 1.245 | 1 | Low RR + High P | No | 0.807 | 1153846 |
| 20209 | Wyandotte County, KS | 1.303 | 0.99 | Low RR + High P | No | 0.806 | 330907 |
| 47109 | McNairy County, TN | 1.631 | 0.91 | High RR + High P | No | 0.806 | 52156 |
| 16005 | Bannock County, ID | 1.515 | 0.91 | High RR + High P | No | 0.806 | 180025 |
| 22093 | St. James Parish, LA | 1.848 | 0.87 | High RR + High P | No | 0.806 | 38579 |
| 21107 | Hopkins County, KY | 2.696 | 0.76 | High RR + Low P | No | 0.805 | 89855 |
| 45031 | Darlington County, SC | 1.487 | 0.93 | High RR + High P | No | 0.805 | 124852 |
| 37093 | Hoke County, NC | 1.663 | 0.85 | High RR + High P | No | 0.804 | 108137 |
| 55083 | Oconto County, WI | 1.733 | 0.85 | High RR + High P | No | 0.804 | 79471 |
| 51141 | Patrick County, VA | 1.494 | 0.98 | High RR + High P | No | 0.804 | 35110 |
| 8059 | Jefferson County, CO | 1.232 | 1 | Low RR + High P | No | 0.803 | 1152177 |
| 24019 | Dorchester County, MD | 1.598 | 0.9 | High RR + High P | No | 0.803 | 65462 |
| 19193 | Woodbury County, IA | 1.39 | 0.95 | Low RR + High P | No | 0.802 | 211509 |
| 27099 | Mower County, MN | 1.635 | 0.88 | High RR + High P | No | 0.802 | 80108 |
| 37137 | Pamlico County, NC | 1.65 | 0.94 | High RR + High P | No | 0.802 | 24657 |
| 40051 | Grady County, OK | 2.14 | 0.77 | High RR + Low P | No | 0.802 | 114023 |
| 22067 | Morehouse Parish, LA | 1.538 | 0.94 | High RR + High P | No | 0.801 | 48369 |
| 40085 | Love County, OK | 2.248 | 0.84 | High RR + High P | No | 0.801 | 20470 |
| 12133 | Washington County, FL | 1.499 | 0.96 | High RR + High P | No | 0.8 | 51007 |
| 48419 | Shelby County, TX | 1.633 | 0.91 | High RR + High P | No | 0.8 | 48240 |
| 21147 | McCreary County, KY | 1.968 | 0.84 | High RR + High P | No | 0.798 | 33833 |
| 48115 | Dawson County, TX | 1.747 | 0.91 | High RR + High P | No | 0.798 | 24180 |
| 42039 | Crawford County, PA | 1.316 | 0.99 | Low RR + High P | No | 0.797 | 164406 |
| 21079 | Garrard County, KY | 1.822 | 0.86 | High RR + High P | No | 0.797 | 35395 |
| 21167 | Mercer County, KY | 2.034 | 0.81 | High RR + High P | No | 0.796 | 45997 |
| 21095 | Harlan County, KY | 2.383 | 0.78 | High RR + Low P | No | 0.796 | 50976 |
| 5111 | Poinsett County, AR | 1.801 | 0.84 | High RR + High P | No | 0.796 | 44850 |
| 37035 | Catawba County, NC | 1.239 | 1 | Low RR + High P | No | 0.795 | 327812 |
| 29073 | Gasconade County, MO | 1.52 | 0.97 | High RR + High P | No | 0.795 | 29477 |
| 37071 | Gaston County, NC | 1.398 | 0.92 | Low RR + High P | No | 0.795 | 471212 |
| 13187 | Lumpkin County, GA | 1.347 | 1 | Low RR + High P | No | 0.795 | 69964 |
| 18067 | Howard County, IN | 1.315 | 0.99 | Low RR + High P | No | 0.795 | 167377 |
| 40149 | Washita County, OK | 1.607 | 0.94 | High RR + High P | No | 0.794 | 21472 |
| 27011 | Big Stone County, MN | 1.875 | 0.9 | High RR + High P | No | 0.793 | 10254 |
| 48405 | San Augustine County, TX | 1.633 | 0.94 | High RR + High P | No | 0.793 | 15693 |
| 51165 | Rockingham County, VA | 1.302 | 0.99 | Low RR + High P | No | 0.792 | 171982 |
| 55141 | Wood County, WI | 1.272 | 1 | Low RR + High P | No | 0.792 | 147717 |
| 26091 | Lenawee County, MI | 1.646 | 0.81 | High RR + High P | No | 0.791 | 195800 |
| 48237 | Jack County, TX | 1.842 | 0.88 | High RR + High P | No | 0.791 | 17639 |
| 31045 | Dawes County, NE | 1.638 | 0.94 | High RR + High P | No | 0.79 | 16385 |
| 37163 | Sampson County, NC | 1.795 | 0.78 | High RR + Low P | No | 0.789 | 118628 |
| 48149 | Fayette County, TX | 1.505 | 0.93 | High RR + High P | No | 0.789 | 50408 |
| 48251 | Johnson County, TX | 1.485 | 0.86 | High RR + High P | No | 0.789 | 398402 |
| 8043 | Fremont County, CO | 1.596 | 0.85 | High RR + High P | No | 0.789 | 99934 |
| 28035 | Forrest County, MS | 1.581 | 0.84 | High RR + High P | No | 0.788 | 156184 |
| 48343 | Morris County, TX | 1.848 | 0.85 | High RR + High P | No | 0.788 | 24118 |
| 28147 | Walthall County, MS | 2.235 | 0.81 | High RR + High P | No | 0.787 | 27687 |
| 37197 | Yadkin County, NC | 1.37 | 0.97 | Low RR + High P | No | 0.786 | 75281 |
| 13097 | Douglas County, GA | 1.215 | 1 | Low RR + High P | No | 0.786 | 296613 |
| 48353 | Nolan County, TX | 2.196 | 0.8 | High RR + High P | No | 0.786 | 28766 |
| 37131 | Northampton County, NC | 1.557 | 0.92 | High RR + High P | No | 0.785 | 33542 |
| 19015 | Boone County, IA | 1.329 | 1 | Low RR + High P | No | 0.784 | 53169 |
| 55077 | Marquette County, WI | 1.466 | 0.96 | High RR + High P | No | 0.784 | 31615 |
| 12121 | Suwannee County, FL | 1.531 | 0.88 | High RR + High P | No | 0.784 | 91535 |
| 40141 | Tillman County, OK | 2.251 | 0.83 | High RR + High P | No | 0.784 | 13840 |
| 40081 | Lincoln County, OK | 2.391 | 0.74 | High RR + Low P | No | 0.783 | 68696 |
| 21029 | Bullitt County, KY | 1.288 | 0.98 | Low RR + High P | No | 0.783 | 168637 |
| 21081 | Grant County, KY | 2.097 | 0.77 | High RR + Low P | No | 0.783 | 51093 |
| 24005 | Baltimore County, MD | 1.179 | 1 | Low RR + High P | No | 0.782 | 1690689 |
| 47089 | Jefferson County, TN | 1.271 | 1 | Low RR + High P | No | 0.782 | 114498 |
| 35015 | Eddy County, NM | 1.244 | 1 | Low RR + High P | No | 0.782 | 120478 |
| 13313 | Whitfield County, GA | 1.215 | 1 | Low RR + High P | No | 0.782 | 206782 |
| 21233 | Webster County, KY | 1.781 | 0.85 | High RR + High P | No | 0.782 | 25465 |
| 32510 | Carson City, NV | 1.289 | 0.99 | Low RR + High P | No | 0.781 | 116101 |
| 45011 | Barnwell County, SC | 1.591 | 0.89 | High RR + High P | No | 0.781 | 40918 |
| 13119 | Franklin County, GA | 1.482 | 0.93 | High RR + High P | No | 0.781 | 48928 |
| 27123 | Ramsey County, MN | 1.188 | 1 | Low RR + High P | No | 0.781 | 1073378 |
| 22015 | Bossier Parish, LA | 1.862 | 0.73 | High RR + Low P | No | 0.779 | 258948 |
| 20037 | Crawford County, KS | 1.334 | 0.98 | Low RR + High P | No | 0.779 | 77909 |
| 53041 | Lewis County, WA | 1.548 | 0.83 | High RR + High P | No | 0.779 | 171511 |
| 17073 | Henry County, IL | 1.324 | 0.97 | Low RR + High P | No | 0.779 | 97044 |
| 41011 | Coos County, OR | 1.386 | 0.93 | Low RR + High P | No | 0.778 | 129135 |
| 12059 | Holmes County, FL | 1.521 | 0.92 | High RR + High P | No | 0.778 | 39597 |
| 20161 | Riley County, KS | 1.257 | 0.99 | Low RR + High P | No | 0.778 | 142965 |
| 42051 | Fayette County, PA | 2.15 | 0.7 | High RR + Low P | No | 0.778 | 249283 |
| 48483 | Wheeler County, TX | 1.641 | 0.93 | High RR + High P | No | 0.778 | 9591 |
| 27119 | Polk County, MN | 1.726 | 0.81 | High RR + High P | No | 0.778 | 61137 |
| 19145 | Page County, IA | 1.722 | 0.86 | High RR + High P | No | 0.778 | 30190 |
| 48227 | Howard County, TX | 1.859 | 0.78 | High RR + Low P | No | 0.777 | 61286 |
| 21051 | Clay County, KY | 3.167 | 0.74 | High RR + Low P | No | 0.777 | 39522 |
| 47071 | Hardin County, TN | 1.433 | 0.94 | High RR + High P | No | 0.777 | 54294 |
| 28129 | Smith County, MS | 1.671 | 0.88 | High RR + High P | No | 0.776 | 28181 |
| 55029 | Door County, WI | 1.546 | 0.88 | High RR + High P | No | 0.776 | 61113 |
| 56021 | Laramie County, WY | 1.382 | 0.91 | Low RR + High P | No | 0.776 | 201765 |
| 40069 | Johnston County, OK | 2.153 | 0.8 | High RR + High P | No | 0.775 | 20562 |
| 30111 | Yellowstone County, MT | 1.296 | 0.95 | Low RR + High P | No | 0.775 | 340713 |
| 16055 | Kootenai County, ID | 1.216 | 0.99 | Low RR + High P | No | 0.775 | 368300 |
| 48449 | Titus County, TX | 1.436 | 0.92 | High RR + High P | No | 0.775 | 62594 |
| 16039 | Elmore County, ID | 1.599 | 0.84 | High RR + High P | No | 0.774 | 59115 |
| 40011 | Blaine County, OK | 1.796 | 0.85 | High RR + High P | No | 0.774 | 16984 |
| 36057 | Montgomery County, NY | 1.309 | 0.97 | Low RR + High P | No | 0.773 | 98701 |
| 40021 | Cherokee County, OK | 2.457 | 0.7 | High RR + Low P | No | 0.771 | 96259 |
| 48289 | Leon County, TX | 1.582 | 0.89 | High RR + High P | No | 0.77 | 32758 |
| 26161 | Washtenaw County, MI | 1.179 | 1 | Low RR + High P | No | 0.77 | 732040 |
| 21159 | Martin County, KY | 1.832 | 0.82 | High RR + High P | No | 0.77 | 22024 |
| 54071 | Pendleton County, WV | 1.579 | 0.93 | High RR + High P | No | 0.769 | 12096 |
| 54025 | Greenbrier County, WV | 1.378 | 0.94 | Low RR + High P | No | 0.768 | 64524 |
| 55103 | Richland County, WI | 1.498 | 0.92 | High RR + High P | No | 0.768 | 34325 |
| 21209 | Scott County, KY | 1.294 | 0.97 | Low RR + High P | No | 0.768 | 119245 |
| 21021 | Boyle County, KY | 1.512 | 0.88 | High RR + High P | No | 0.768 | 61839 |
| 48311 | McMullen County, TX | 1.946 | 0.85 | High RR + High P | No | 0.768 | 1137 |
| 21071 | Floyd County, KY | 1.692 | 0.8 | High RR + Low P | No | 0.767 | 69341 |
| 13047 | Catoosa County, GA | 1.269 | 0.97 | Low RR + High P | No | 0.767 | 137746 |
| 45049 | Hampton County, SC | 2.141 | 0.76 | High RR + Low P | No | 0.766 | 36258 |
| 10005 | Sussex County, DE | 1.161 | 1 | Low RR + High P | No | 0.766 | 519956 |
| 47103 | Lincoln County, TN | 1.347 | 0.95 | Low RR + High P | No | 0.765 | 72175 |
| 28113 | Pike County, MS | 1.43 | 0.9 | Low RR + High P | No | 0.765 | 79081 |
| 19027 | Carroll County, IA | 1.508 | 0.9 | High RR + High P | No | 0.765 | 41063 |
| 19031 | Cedar County, IA | 1.535 | 0.89 | High RR + High P | No | 0.765 | 36655 |
| 18165 | Vermillion County, IN | 2.241 | 0.76 | High RR + Low P | No | 0.765 | 30850 |
| 45005 | Allendale County, SC | 2.069 | 0.8 | High RR + High P | No | 0.765 | 14787 |
| 27147 | Steele County, MN | 1.445 | 0.89 | High RR + High P | No | 0.764 | 74850 |
| 29077 | Greene County, MO | 1.44 | 0.82 | High RR + High P | No | 0.764 | 607808 |
| 19019 | Buchanan County, IA | 1.591 | 0.85 | High RR + High P | No | 0.764 | 41380 |
| 48401 | Rusk County, TX | 1.437 | 0.88 | High RR + High P | No | 0.763 | 105603 |
| 42089 | Monroe County, PA | 1.162 | 1 | Low RR + High P | No | 0.763 | 333504 |
| 21199 | Pulaski County, KY | 1.52 | 0.82 | High RR + High P | No | 0.762 | 131958 |
| 51770 | Roanoke city, VA | 1.264 | 0.96 | Low RR + High P | No | 0.762 | 194828 |
| 8041 | El Paso County, CO | 1.193 | 0.98 | Low RR + High P | No | 0.762 | 1484811 |
| 13255 | Spalding County, GA | 1.183 | 1 | Low RR + High P | No | 0.762 | 138954 |
| 13013 | Barrow County, GA | 1.205 | 0.99 | Low RR + High P | No | 0.761 | 182037 |
| 18113 | Noble County, IN | 1.437 | 0.88 | High RR + High P | No | 0.761 | 94664 |
| 47045 | Dyer County, TN | 1.613 | 0.8 | High RR + High P | No | 0.761 | 72938 |
| 42087 | Mifflin County, PA | 1.418 | 0.89 | Low RR + High P | No | 0.76 | 91906 |
| 42055 | Franklin County, PA | 1.322 | 0.91 | Low RR + High P | No | 0.76 | 314805 |
| 48461 | Upton County, TX | 2.153 | 0.81 | High RR + High P | No | 0.76 | 6255 |
| 28105 | Oktibbeha County, MS | 1.477 | 0.84 | High RR + High P | No | 0.76 | 102587 |
| 48239 | Jackson County, TX | 1.438 | 0.94 | High RR + High P | No | 0.76 | 30404 |
| 28111 | Perry County, MS | 2.316 | 0.76 | High RR + Low P | No | 0.76 | 22713 |
| 19175 | Union County, IA | 1.875 | 0.79 | High RR + Low P | No | 0.759 | 23809 |
| 56013 | Fremont County, WY | 1.632 | 0.79 | High RR + Low P | No | 0.759 | 79381 |
| 8001 | Adams County, CO | 1.157 | 1 | Low RR + High P | No | 0.759 | 1060874 |
| 21083 | Graves County, KY | 1.384 | 0.92 | Low RR + High P | No | 0.758 | 72954 |
| 48455 | Trinity County, TX | 1.432 | 0.94 | High RR + High P | No | 0.758 | 28221 |
| 56003 | Big Horn County, WY | 1.434 | 0.94 | High RR + High P | No | 0.757 | 23897 |
| 51185 | Tazewell County, VA | 1.453 | 0.87 | High RR + High P | No | 0.757 | 78683 |
| 19023 | Butler County, IA | 2.22 | 0.75 | High RR + Low P | No | 0.756 | 28418 |
| 21137 | Lincoln County, KY | 2.494 | 0.7 | High RR + Low P | No | 0.756 | 49195 |
| 48189 | Hale County, TX | 1.439 | 0.89 | High RR + High P | No | 0.756 | 63727 |
| 18053 | Grant County, IN | 1.404 | 0.87 | Low RR + High P | No | 0.755 | 132193 |
| 24017 | Charles County, MD | 1.214 | 0.97 | Low RR + High P | No | 0.755 | 342084 |
| 31111 | Lincoln County, NE | 1.282 | 0.98 | Low RR + High P | No | 0.755 | 66979 |
| 13197 | Marion County, GA | 1.574 | 0.9 | High RR + High P | No | 0.754 | 14876 |
| 35025 | Lea County, NM | 1.224 | 0.98 | Low RR + High P | No | 0.754 | 144444 |
| 36015 | Chemung County, NY | 1.563 | 0.78 | High RR + Low P | No | 0.754 | 162966 |
| 27003 | Anoka County, MN | 1.159 | 0.99 | Low RR + High P | No | 0.754 | 741549 |
| 55097 | Portage County, WI | 1.234 | 0.97 | Low RR + High P | No | 0.753 | 141658 |
| 13157 | Jackson County, GA | 1.186 | 0.99 | Low RR + High P | No | 0.753 | 172624 |
| 22049 | Jackson Parish, LA | 1.722 | 0.81 | High RR + High P | No | 0.753 | 29562 |
| 40135 | Sequoyah County, OK | 2.265 | 0.68 | High RR + Low P | No | 0.753 | 80092 |
| 13283 | Treutlen County, GA | 1.679 | 0.86 | High RR + High P | No | 0.753 | 12700 |
| 48473 | Waller County, TX | 1.28 | 0.95 | Low RR + High P | No | 0.753 | 125405 |
| 48261 | Kenedy County, TX | 4.867 | 0.77 | High RR + Low P | No | 0.752 | 697 |
| 54053 | Mason County, WV | 1.34 | 0.95 | Low RR + High P | No | 0.752 | 49676 |
| 51700 | Newport News city, VA | 1.347 | 0.88 | Low RR + High P | No | 0.752 | 367098 |
| 19153 | Polk County, IA | 1.119 | 1 | Low RR + High P | No | 0.752 | 1006439 |
| 42075 | Lebanon County, PA | 1.152 | 1 | Low RR + High P | No | 0.751 | 288154 |
| 28161 | Yalobusha County, MS | 1.628 | 0.84 | High RR + High P | No | 0.751 | 24799 |
| 49039 | Sanpete County, UT | 1.324 | 0.95 | Low RR + High P | No | 0.751 | 59968 |
| 28055 | Issaquena County, MS | 2.147 | 0.8 | High RR + Low P | No | 0.751 | 2518 |
| 12029 | Dixie County, FL | 1.343 | 0.97 | Low RR + High P | No | 0.751 | 34585 |
| 18035 | Delaware County, IN | 1.525 | 0.78 | High RR + Low P | No | 0.75 | 224484 |
| 48293 | Limestone County, TX | 1.723 | 0.78 | High RR + Low P | No | 0.749 | 44414 |
| 53073 | Whatcom County, WA | 1.199 | 0.96 | Low RR + High P | No | 0.749 | 462517 |
| 31113 | Logan County, NE | 2.269 | 0.79 | High RR + Low P | No | 0.749 | 1340 |
| 40029 | Coal County, OK | 1.937 | 0.8 | High RR + Low P | No | 0.749 | 10557 |
| 54087 | Roane County, WV | 1.639 | 0.82 | High RR + High P | No | 0.749 | 27576 |
| 54081 | Raleigh County, WV | 1.345 | 0.89 | Low RR + High P | No | 0.748 | 145157 |
| 21013 | Bell County, KY | 2.804 | 0.68 | High RR + Low P | No | 0.747 | 46928 |
| 31145 | Red Willow County, NE | 1.452 | 0.92 | High RR + High P | No | 0.747 | 20985 |
| 54039 | Kanawha County, WV | 1.332 | 0.88 | Low RR + High P | No | 0.747 | 350669 |
| 48423 | Smith County, TX | 1.118 | 1 | Low RR + High P | No | 0.746 | 487204 |
| 5091 | Miller County, AR | 1.503 | 0.82 | High RR + High P | No | 0.746 | 85023 |
| 31053 | Dodge County, NE | 1.388 | 0.89 | Low RR + High P | No | 0.746 | 74232 |
| 18181 | White County, IN | 1.453 | 0.88 | High RR + High P | No | 0.746 | 49577 |
| 30063 | Missoula County, MT | 1.152 | 1 | Low RR + High P | No | 0.746 | 242860 |
| 2230 | Skagway Municipality, AK | 1.736 | 0.85 | High RR + High P | No | 0.746 | 2187 |
| 31059 | Fillmore County, NE | 1.575 | 0.89 | High RR + High P | No | 0.745 | 11089 |
| 26059 | Hillsdale County, MI | 2.101 | 0.67 | High RR + Low P | No | 0.745 | 91322 |
| 32003 | Clark County, NV | 1.096 | 1 | Low RR + High P | No | 0.745 | 4659108 |
| 48001 | Anderson County, TX | 1.238 | 0.96 | Low RR + High P | No | 0.745 | 114963 |
| 37009 | Ashe County, NC | 1.349 | 0.93 | Low RR + High P | No | 0.745 | 54125 |
| 28099 | Neshoba County, MS | 1.579 | 0.8 | High RR + Low P | No | 0.744 | 57593 |
| 12125 | Union County, FL | 2.107 | 0.73 | High RR + Low P | No | 0.744 | 31046 |
| 42067 | Juniata County, PA | 1.398 | 0.91 | Low RR + High P | No | 0.744 | 46524 |
| 27025 | Chisago County, MN | 1.264 | 0.95 | Low RR + High P | No | 0.744 | 116433 |
| 20059 | Franklin County, KS | 1.57 | 0.81 | High RR + High P | No | 0.743 | 52084 |
| 24021 | Frederick County, MD | 1.19 | 0.96 | Low RR + High P | No | 0.743 | 580931 |
| 20139 | Osage County, KS | 1.404 | 0.92 | Low RR + High P | No | 0.743 | 31529 |
| 27059 | Isanti County, MN | 1.239 | 0.97 | Low RR + High P | No | 0.742 | 85893 |
| 23003 | Aroostook County, ME | 1.171 | 0.99 | Low RR + High P | No | 0.742 | 134673 |
| 47115 | Marion County, TN | 1.552 | 0.8 | High RR + High P | No | 0.741 | 58432 |
| 55131 | Washington County, WI | 1.132 | 1 | Low RR + High P | No | 0.741 | 275924 |
| 28163 | Yazoo County, MS | 1.362 | 0.92 | Low RR + High P | No | 0.74 | 51689 |
| 47069 | Hardeman County, TN | 1.747 | 0.75 | High RR + Low P | No | 0.74 | 51078 |
| 26129 | Ogemaw County, MI | 1.271 | 0.98 | Low RR + High P | No | 0.74 | 41909 |
| 40045 | Ellis County, OK | 1.578 | 0.89 | High RR + High P | No | 0.74 | 7331 |
| 21181 | Nicholas County, KY | 1.624 | 0.84 | High RR + High P | No | 0.74 | 15472 |
| 21219 | Todd County, KY | 1.625 | 0.82 | High RR + High P | No | 0.739 | 24888 |
| 48103 | Crane County, TX | 2.086 | 0.77 | High RR + Low P | No | 0.738 | 9125 |
| 55031 | Douglas County, WI | 1.277 | 0.94 | Low RR + High P | No | 0.738 | 88490 |
| 27093 | Meeker County, MN | 1.531 | 0.82 | High RR + High P | No | 0.738 | 47016 |
| 27069 | Kittson County, MN | 1.674 | 0.84 | High RR + High P | No | 0.738 | 8146 |
| 18025 | Crawford County, IN | 1.445 | 0.91 | High RR + High P | No | 0.737 | 20955 |
| 8071 | Las Animas County, CO | 1.34 | 0.95 | Low RR + High P | No | 0.737 | 28659 |
| 31127 | Nemaha County, NE | 1.68 | 0.82 | High RR + High P | No | 0.737 | 14112 |
| 39071 | Highland County, OH | 1.232 | 0.96 | Low RR + High P | No | 0.737 | 87040 |
| 22095 | St. John the Baptist Parish, LA | 1.256 | 0.96 | Low RR + High P | No | 0.736 | 79547 |
| 24025 | Harford County, MD | 1.112 | 1 | Low RR + High P | No | 0.736 | 528459 |
| 18021 | Clay County, IN | 1.388 | 0.89 | Low RR + High P | No | 0.736 | 52870 |
| 56005 | Campbell County, WY | 1.427 | 0.82 | Low RR + High P | No | 0.736 | 94557 |
| 48185 | Grimes County, TX | 1.355 | 0.9 | Low RR + High P | No | 0.736 | 63554 |
| 21225 | Union County, KY | 1.477 | 0.88 | High RR + High P | No | 0.736 | 26210 |
| 40087 | McClain County, OK | 1.914 | 0.67 | High RR + Low P | No | 0.736 | 92482 |
| 21125 | Laurel County, KY | 1.528 | 0.77 | High RR + Low P | No | 0.736 | 126175 |
| 32011 | Eureka County, NV | 1.597 | 0.88 | High RR + High P | No | 0.735 | 3780 |
| 40099 | Murray County, OK | 2.135 | 0.72 | High RR + Low P | No | 0.735 | 27435 |
| 53047 | Okanogan County, WA | 1.273 | 0.94 | Low RR + High P | No | 0.734 | 87172 |
| 28107 | Panola County, MS | 3.426 | 0.63 | High RR + Low P | No | 0.734 | 65261 |
| 19017 | Bremer County, IA | 1.368 | 0.9 | Low RR + High P | No | 0.734 | 50561 |
| 28029 | Copiah County, MS | 1.588 | 0.78 | High RR + Low P | No | 0.733 | 55376 |
| 48375 | Potter County, TX | 1.319 | 0.87 | Low RR + High P | No | 0.733 | 229558 |
| 27131 | Rice County, MN | 1.237 | 0.94 | Low RR + High P | No | 0.733 | 135471 |
| 48023 | Baylor County, TX | 1.597 | 0.86 | High RR + High P | No | 0.733 | 6925 |
| 39019 | Carroll County, OH | 1.819 | 0.71 | High RR + Low P | No | 0.733 | 53444 |
| 48257 | Kaufman County, TX | 1.368 | 0.82 | Low RR + High P | No | 0.733 | 358301 |
| 40005 | Atoka County, OK | 1.59 | 0.81 | High RR + High P | No | 0.732 | 28852 |
| 18019 | Clark County, IN | 1.132 | 0.99 | Low RR + High P | No | 0.732 | 249689 |
| 31091 | Hooker County, NE | 1.958 | 0.78 | High RR + Low P | No | 0.731 | 1361 |
| 19039 | Clarke County, IA | 1.499 | 0.88 | High RR + High P | No | 0.731 | 19231 |
| 47023 | Chester County, TN | 1.574 | 0.8 | High RR + High P | No | 0.731 | 35164 |
| 51089 | Henry County, VA | 1.269 | 0.93 | Low RR + High P | No | 0.731 | 99497 |
| 45001 | Abbeville County, SC | 1.176 | 1 | Low RR + High P | No | 0.731 | 48772 |
| 48277 | Lamar County, TX | 1.474 | 0.79 | High RR + Low P | No | 0.73 | 101642 |
| 30069 | Petroleum County, MT | 2.429 | 0.75 | High RR + Low P | No | 0.73 | 1075 |
| 27045 | Fillmore County, MN | 1.769 | 0.73 | High RR + Low P | No | 0.73 | 42976 |
| 19135 | Monroe County, IA | 1.877 | 0.76 | High RR + Low P | No | 0.73 | 15034 |
| 27021 | Cass County, MN | 1.246 | 0.96 | Low RR + High P | No | 0.729 | 62754 |
| 40055 | Greer County, OK | 1.674 | 0.82 | High RR + High P | No | 0.729 | 11012 |
| 29095 | Jackson County, MO | 1.179 | 0.93 | Low RR + High P | No | 0.729 | 1434778 |
| 56045 | Weston County, WY | 1.502 | 0.89 | High RR + High P | No | 0.729 | 13666 |
| 53045 | Mason County, WA | 1.191 | 0.96 | Low RR + High P | No | 0.728 | 136560 |
| 18005 | Bartholomew County, IN | 1.113 | 1 | Low RR + High P | No | 0.728 | 167397 |
| 27137 | St. Louis County, MN | 1.217 | 0.92 | Low RR + High P | No | 0.727 | 400072 |
| 28005 | Amite County, MS | 2.29 | 0.7 | High RR + Low P | No | 0.727 | 25001 |
| 27145 | Stearns County, MN | 1.119 | 0.99 | Low RR + High P | No | 0.727 | 321219 |
| 13129 | Gordon County, GA | 1.245 | 0.93 | Low RR + High P | No | 0.727 | 118612 |
| 31119 | Madison County, NE | 1.526 | 0.78 | High RR + Low P | No | 0.726 | 71041 |
| 21141 | Logan County, KY | 1.298 | 0.93 | Low RR + High P | No | 0.726 | 56139 |
| 48291 | Liberty County, TX | 1.553 | 0.72 | High RR + Low P | No | 0.726 | 210734 |
| 27067 | Kandiyohi County, MN | 1.21 | 0.96 | Low RR + High P | No | 0.726 | 87584 |
| 51730 | Petersburg city, VA | 1.221 | 0.96 | Low RR + High P | No | 0.726 | 66771 |
| 21045 | Casey County, KY | 1.693 | 0.76 | High RR + Low P | No | 0.726 | 31829 |
| 8053 | Hinsdale County, CO | 1.959 | 0.77 | High RR + Low P | No | 0.726 | 1536 |
| 55079 | Milwaukee County, WI | 1.644 | 0.65 | High RR + Low P | No | 0.725 | 1834283 |
| 42063 | Indiana County, PA | 1.148 | 0.98 | Low RR + High P | No | 0.725 | 166158 |
| 40123 | Pontotoc County, OK | 2.26 | 0.63 | High RR + Low P | No | 0.725 | 76428 |
| 51025 | Brunswick County, VA | 1.237 | 0.99 | Low RR + High P | No | 0.724 | 31580 |
| 45021 | Cherokee County, SC | 1.15 | 0.99 | Low RR + High P | No | 0.724 | 112835 |
| 17009 | Brown County, IL | 1.572 | 0.84 | High RR + High P | No | 0.724 | 12610 |
| 8075 | Logan County, CO | 1.473 | 0.82 | High RR + High P | No | 0.724 | 41411 |
| 27061 | Itasca County, MN | 1.56 | 0.75 | High RR + Low P | No | 0.723 | 90559 |
| 53021 | Franklin County, WA | 1.176 | 0.96 | Low RR + High P | No | 0.723 | 197616 |
| 47055 | Giles County, TN | 1.503 | 0.79 | High RR + Low P | No | 0.723 | 61261 |
| 46063 | Harding County, SD | 1.81 | 0.78 | High RR + Low P | No | 0.723 | 2663 |
| 49007 | Carbon County, UT | 1.557 | 0.78 | High RR + Low P | No | 0.723 | 41149 |
| 27167 | Wilkin County, MN | 1.539 | 0.85 | High RR + High P | No | 0.722 | 12664 |
| 19077 | Guthrie County, IA | 1.505 | 0.84 | High RR + High P | No | 0.722 | 21403 |
| 45063 | Lexington County, SC | 1.063 | 1 | Low RR + High P | No | 0.722 | 614633 |
| 55123 | Vernon County, WI | 1.367 | 0.86 | Low RR + High P | No | 0.721 | 62233 |
| 48451 | Tom Green County, TX | 1.163 | 0.96 | Low RR + High P | No | 0.721 | 237970 |
| 37123 | Montgomery County, NC | 1.652 | 0.73 | High RR + Low P | No | 0.721 | 51926 |
| 37083 | Halifax County, NC | 1.76 | 0.66 | High RR + Low P | No | 0.719 | 95009 |
| 31067 | Gage County, NE | 1.395 | 0.86 | Low RR + High P | No | 0.719 | 43198 |
| 20133 | Neosho County, KS | 1.272 | 0.96 | Low RR + High P | No | 0.718 | 31003 |
| 55055 | Jefferson County, WI | 1.281 | 0.87 | Low RR + High P | No | 0.718 | 171468 |
| 48203 | Harrison County, TX | 1.381 | 0.8 | Low RR + High P | No | 0.718 | 140933 |
| 46003 | Aurora County, SD | 1.874 | 0.76 | High RR + Low P | No | 0.717 | 5544 |
| 40095 | Marshall County, OK | 2.161 | 0.67 | High RR + Low P | No | 0.717 | 31891 |
| 19001 | Adair County, IA | 1.553 | 0.83 | High RR + High P | No | 0.716 | 14846 |
| 35057 | Torrance County, NM | 1.275 | 0.96 | Low RR + High P | No | 0.716 | 31016 |
| 12075 | Levy County, FL | 1.287 | 0.89 | Low RR + High P | No | 0.716 | 91798 |
| 21191 | Pendleton County, KY | 1.326 | 0.92 | Low RR + High P | No | 0.716 | 29513 |
| 31153 | Sarpy County, NE | 1.182 | 0.92 | Low RR + High P | No | 0.715 | 396313 |
| 17163 | St. Clair County, IL | 1.085 | 0.99 | Low RR + High P | No | 0.715 | 503283 |
| 48087 | Collingsworth County, TX | 1.583 | 0.83 | High RR + High P | No | 0.715 | 5148 |
| 22065 | Madison Parish, LA | 1.92 | 0.71 | High RR + Low P | No | 0.715 | 18726 |
| 17125 | Mason County, IL | 1.42 | 0.87 | Low RR + High P | No | 0.715 | 25222 |
| 48219 | Hockley County, TX | 1.394 | 0.85 | Low RR + High P | No | 0.714 | 42738 |
| 19169 | Story County, IA | 1.109 | 0.99 | Low RR + High P | No | 0.714 | 196823 |
| 37125 | Moore County, NC | 1.138 | 0.97 | Low RR + High P | No | 0.714 | 212389 |
| 47129 | Morgan County, TN | 1.332 | 0.9 | Low RR + High P | No | 0.714 | 42780 |
| 46137 | Ziebach County, SD | 1.828 | 0.76 | High RR + Low P | No | 0.714 | 4716 |
| 20155 | Reno County, KS | 1.172 | 0.96 | Low RR + High P | No | 0.714 | 123049 |
| 56037 | Sweetwater County, WY | 1.202 | 0.95 | Low RR + High P | No | 0.713 | 82623 |
| 39119 | Muskingum County, OH | 1.099 | 0.99 | Low RR + High P | No | 0.713 | 172419 |
| 42065 | Jefferson County, PA | 1.197 | 0.95 | Low RR + High P | No | 0.713 | 87343 |
| 48233 | Hutchinson County, TX | 1.599 | 0.75 | High RR + Low P | No | 0.712 | 40255 |
| 19071 | Fremont County, IA | 2.085 | 0.71 | High RR + Low P | No | 0.712 | 12933 |
| 22057 | Lafourche Parish, LA | 1.06 | 1 | Low RR + High P | No | 0.711 | 190807 |
| 19133 | Monona County, IA | 1.74 | 0.75 | High RR + Low P | No | 0.711 | 16999 |
| 31103 | Keya Paha County, NE | 1.82 | 0.76 | High RR + Low P | No | 0.711 | 1610 |
| 22117 | Washington Parish, LA | 1.104 | 1 | Low RR + High P | No | 0.711 | 89856 |
| 31131 | Otoe County, NE | 1.295 | 0.93 | Low RR + High P | No | 0.71 | 32521 |
| 13059 | Clarke County, GA | 1.063 | 1 | Low RR + High P | No | 0.71 | 259659 |
| 27043 | Faribault County, MN | 1.528 | 0.8 | High RR + Low P | No | 0.71 | 27803 |
| 18045 | Fountain County, IN | 1.223 | 0.97 | Low RR + High P | No | 0.71 | 33351 |
| 32009 | Esmeralda County, NV | 1.665 | 0.8 | High RR + High P | No | 0.71 | 1483 |
| 27039 | Dodge County, MN | 1.86 | 0.67 | High RR + Low P | No | 0.71 | 42080 |
| 37165 | Scotland County, NC | 1.51 | 0.76 | High RR + Low P | No | 0.709 | 68726 |
| 17187 | Warren County, IL | 1.447 | 0.82 | High RR + High P | No | 0.709 | 32545 |
| 48003 | Andrews County, TX | 1.751 | 0.7 | High RR + Low P | No | 0.709 | 36988 |
| 48351 | Newton County, TX | 1.409 | 0.86 | Low RR + High P | No | 0.709 | 24080 |
| 19191 | Winneshiek County, IA | 1.357 | 0.87 | Low RR + High P | No | 0.708 | 39717 |
| 46067 | Hutchinson County, SD | 1.672 | 0.77 | High RR + Low P | No | 0.708 | 14745 |
| 21003 | Allen County, KY | 1.414 | 0.82 | Low RR + High P | No | 0.707 | 43071 |
| 33019 | Sullivan County, NH | 1.201 | 0.93 | Low RR + High P | No | 0.707 | 87887 |
| 42007 | Beaver County, PA | 1.065 | 0.99 | Low RR + High P | No | 0.707 | 331368 |
| 55045 | Green County, WI | 1.298 | 0.88 | Low RR + High P | No | 0.707 | 73836 |
| 17039 | De Witt County, IL | 1.464 | 0.81 | High RR + High P | No | 0.706 | 30730 |
| 48275 | Knox County, TX | 1.951 | 0.72 | High RR + Low P | No | 0.706 | 6586 |
| 16001 | Ada County, ID | 1.01 | 1 | Low RR + High P | No | 0.706 | 1043677 |
| 48173 | Glasscock County, TX | 2.962 | 0.69 | High RR + Low P | No | 0.706 | 2280 |
| 48137 | Edwards County, TX | 1.795 | 0.76 | High RR + Low P | No | 0.706 | 2821 |
| 27095 | Mille Lacs County, MN | 1.648 | 0.7 | High RR + Low P | No | 0.706 | 54722 |
| 51187 | Warren County, VA | 1.297 | 0.86 | Low RR + High P | No | 0.706 | 83247 |
| 21123 | Larue County, KY | 1.562 | 0.77 | High RR + Low P | No | 0.706 | 30453 |
| 46099 | Minnehaha County, SD | 1.412 | 0.74 | Low RR + Low P | No | 0.706 | 410775 |
| 47171 | Unicoi County, TN | 1.392 | 0.84 | Low RR + High P | No | 0.706 | 35347 |
| 37001 | Alamance County, NC | 1.101 | 0.97 | Low RR + High P | No | 0.706 | 355783 |
| 12049 | Hardee County, FL | 1.281 | 0.91 | Low RR + High P | No | 0.705 | 51411 |
| 48295 | Lipscomb County, TX | 1.655 | 0.78 | High RR + Low P | No | 0.705 | 5752 |
| 45061 | Lee County, SC | 1.317 | 0.91 | Low RR + High P | No | 0.705 | 32128 |
| 19131 | Mitchell County, IA | 1.495 | 0.82 | High RR + High P | No | 0.705 | 21062 |
| 8103 | Rio Blanco County, CO | 1.477 | 0.84 | High RR + High P | No | 0.704 | 13118 |
| 48307 | McCulloch County, TX | 1.323 | 0.94 | Low RR + High P | No | 0.704 | 14920 |
| 48309 | McLennan County, TX | 1.784 | 0.59 | High RR + Low P | No | 0.704 | 534745 |
| 48387 | Red River County, TX | 1.501 | 0.81 | High RR + High P | No | 0.704 | 23283 |
| 35009 | Curry County, NM | 1.106 | 0.99 | Low RR + High P | No | 0.704 | 94652 |
| 40093 | Major County, OK | 1.848 | 0.71 | High RR + Low P | No | 0.703 | 15117 |
| 13145 | Harris County, GA | 1.221 | 0.92 | Low RR + High P | No | 0.703 | 72911 |
| 55061 | Kewaunee County, WI | 1.206 | 0.96 | Low RR + High P | No | 0.703 | 41343 |
| 54047 | McDowell County, WV | 1.742 | 0.7 | High RR + Low P | No | 0.703 | 35245 |
| 40067 | Jefferson County, OK | 2.581 | 0.67 | High RR + Low P | No | 0.703 | 10755 |
| 12101 | Pasco County, FL | 1 | 1 | Low RR + High P | No | 0.702 | 1242137 |
| 27103 | Nicollet County, MN | 1.186 | 0.95 | Low RR + High P | No | 0.701 | 68585 |
| 13139 | Hall County, GA | 1.011 | 1 | Low RR + High P | No | 0.701 | 429888 |
| 21015 | Boone County, KY | 1.416 | 0.73 | Low RR + Low P | No | 0.701 | 279541 |
| 30003 | Big Horn County, MT | 1.744 | 0.71 | High RR + Low P | No | 0.7 | 25577 |
| 51520 | Bristol city, VA | 1.397 | 0.83 | Low RR + High P | No | 0.7 | 33747 |
| 5127 | Scott County, AR | 1.281 | 0.94 | Low RR + High P | No | 0.7 | 19647 |
| 48059 | Callahan County, TX | 1.29 | 0.92 | Low RR + High P | No | 0.7 | 28621 |
| 19151 | Pocahontas County, IA | 1.687 | 0.75 | High RR + Low P | No | 0.699 | 14049 |
| 51660 | Harrisonburg city, VA | 1.384 | 0.78 | Low RR + Low P | No | 0.699 | 102232 |
| 1125 | Tuscaloosa County, AL | 1.006 | 1 | Low RR + High P | No | 0.699 | 474063 |
| 18055 | Greene County, IN | 1.227 | 0.92 | Low RR + High P | No | 0.699 | 62267 |
| 6003 | Alpine County, CA | 2.059 | 0.71 | High RR + Low P | No | 0.698 | 2331 |
| 47039 | Decatur County, TN | 1.726 | 0.72 | High RR + Low P | No | 0.698 | 23205 |
| 53063 | Spokane County, WA | 1.029 | 0.99 | Low RR + High P | No | 0.698 | 1101195 |
| 30037 | Golden Valley County, MT | 2.351 | 0.69 | High RR + Low P | No | 0.698 | 1661 |
| 29071 | Franklin County, MO | 1.197 | 0.89 | Low RR + High P | No | 0.698 | 212197 |
| 51171 | Shenandoah County, VA | 1.146 | 0.96 | Low RR + High P | No | 0.698 | 90164 |
| 40075 | Kiowa County, OK | 2.583 | 0.64 | High RR + Low P | No | 0.698 | 16878 |
| 51800 | Suffolk city, VA | 1.318 | 0.8 | Low RR + Low P | No | 0.697 | 199085 |
| 54083 | Randolph County, WV | 1.29 | 0.88 | Low RR + High P | No | 0.696 | 54902 |
| 29085 | Hickory County, MO | 1.376 | 0.88 | Low RR + High P | No | 0.696 | 17293 |
| 36047 | Kings County, NY | 1.028 | 0.98 | Low RR + High P | No | 0.696 | 5150756 |
| 31097 | Johnson County, NE | 2.232 | 0.67 | High RR + Low P | No | 0.696 | 10443 |
| 22073 | Ouachita Parish, LA | 1.045 | 0.99 | Low RR + High P | No | 0.696 | 315272 |
| 17005 | Bond County, IL | 1.676 | 0.71 | High RR + Low P | No | 0.695 | 32986 |
| 39067 | Harrison County, OH | 1.477 | 0.79 | High RR + Low P | No | 0.695 | 28510 |
| 13039 | Camden County, GA | 1.089 | 0.99 | Low RR + High P | No | 0.695 | 115089 |
| 48235 | Irion County, TX | 2.399 | 0.68 | High RR + Low P | No | 0.695 | 3083 |
| 48117 | Deaf Smith County, TX | 1.317 | 0.88 | Low RR + High P | No | 0.695 | 36746 |
| 48155 | Foard County, TX | 2.534 | 0.67 | High RR + Low P | No | 0.694 | 2142 |
| 48179 | Gray County, TX | 1.565 | 0.73 | High RR + Low P | No | 0.694 | 41896 |
| 19149 | Plymouth County, IA | 1.205 | 0.93 | Low RR + High P | No | 0.694 | 51444 |
| 55125 | Vilas County, WI | 1.602 | 0.7 | High RR + Low P | No | 0.694 | 47627 |
| 47095 | Lake County, TN | 1.916 | 0.69 | High RR + Low P | No | 0.694 | 12847 |
| 30109 | Wibaux County, MT | 2.986 | 0.66 | High RR + Low P | No | 0.693 | 1837 |
| 27157 | Wabasha County, MN | 1.719 | 0.68 | High RR + Low P | No | 0.693 | 43335 |
| 41049 | Morrow County, OR | 1.326 | 0.89 | Low RR + High P | No | 0.693 | 24594 |
| 17017 | Cass County, IL | 1.712 | 0.71 | High RR + Low P | No | 0.693 | 25234 |
| 21099 | Hart County, KY | 1.493 | 0.76 | High RR + Low P | No | 0.693 | 39318 |
| 22097 | St. Landry Parish, LA | 2.378 | 0.55 | High RR + Low P | No | 0.692 | 163143 |
| 20203 | Wichita County, KS | 1.634 | 0.77 | High RR + Low P | No | 0.691 | 4160 |
| 55137 | Waushara County, WI | 1.232 | 0.91 | Low RR + High P | No | 0.691 | 49861 |
| 49057 | Weber County, UT | 1.101 | 0.94 | Low RR + High P | No | 0.691 | 541386 |
| 17053 | Ford County, IL | 1.443 | 0.8 | High RR + High P | No | 0.69 | 26506 |
| 20071 | Greeley County, KS | 2.53 | 0.66 | High RR + Low P | No | 0.69 | 2413 |
| 21115 | Johnson County, KY | 1.336 | 0.83 | Low RR + High P | No | 0.69 | 44351 |
| 18097 | Marion County, IN | 1.299 | 0.76 | Low RR + Low P | No | 0.689 | 1937683 |
| 19005 | Allamakee County, IA | 1.624 | 0.72 | High RR + Low P | No | 0.689 | 28092 |
| 47031 | Coffee County, TN | 1.38 | 0.76 | Low RR + Low P | No | 0.688 | 120412 |
| 37117 | Martin County, NC | 1.78 | 0.64 | High RR + Low P | No | 0.688 | 42925 |
| 48045 | Briscoe County, TX | 1.568 | 0.79 | High RR + Low P | No | 0.688 | 2884 |
| 5073 | Lafayette County, AR | 1.7 | 0.73 | High RR + Low P | No | 0.688 | 12224 |
| 4027 | Yuma County, AZ | 1.006 | 1 | Low RR + High P | No | 0.688 | 422095 |
| 48499 | Wood County, TX | 1.333 | 0.79 | Low RR + Low P | No | 0.687 | 94851 |
| 47009 | Blount County, TN | 1.07 | 0.96 | Low RR + High P | No | 0.687 | 281266 |
| 1045 | Dale County, AL | 1.047 | 1 | Low RR + High P | No | 0.687 | 99366 |
| 48229 | Hudspeth County, TX | 1.5 | 0.81 | High RR + High P | No | 0.687 | 6867 |
| 36085 | Richmond County, NY | 0.994 | 0.99 | Low RR + High P | No | 0.686 | 982045 |
| 51710 | Norfolk city, VA | 1.189 | 0.85 | Low RR + High P | No | 0.686 | 463488 |
| 8063 | Kit Carson County, CO | 1.333 | 0.9 | Low RR + High P | No | 0.686 | 13977 |
| 5071 | Johnson County, AR | 1.11 | 0.99 | Low RR + High P | No | 0.685 | 52099 |
| 12053 | Hernando County, FL | 0.977 | 1 | Low RR + High P | No | 0.685 | 419742 |
| 18101 | Martin County, IN | 1.504 | 0.78 | High RR + Low P | No | 0.685 | 19724 |
| 2068 | Denali Borough, AK | 1.795 | 0.72 | High RR + Low P | No | 0.685 | 3159 |
| 53027 | Grays Harbor County, WA | 1.305 | 0.79 | Low RR + Low P | No | 0.684 | 154331 |
| 24003 | Anne Arundel County, MD | 0.97 | 1 | Low RR + High P | No | 0.684 | 1187929 |
| 27109 | Olmsted County, MN | 1.432 | 0.69 | Low RR + Low P | No | 0.683 | 328820 |
| 21085 | Grayson County, KY | 1.284 | 0.84 | Low RR + High P | No | 0.682 | 53468 |
| 41029 | Jackson County, OR | 1.11 | 0.92 | Low RR + High P | No | 0.681 | 442437 |
| 5081 | Little River County, AR | 1.504 | 0.76 | High RR + Low P | No | 0.681 | 23670 |
| 31115 | Loup County, NE | 3.227 | 0.63 | High RR + Low P | No | 0.681 | 1193 |
| 48111 | Dallam County, TX | 1.738 | 0.7 | High RR + Low P | No | 0.681 | 14472 |
| 27161 | Waseca County, MN | 1.394 | 0.79 | Low RR + Low P | No | 0.681 | 37895 |
| 48331 | Milam County, TX | 1.529 | 0.71 | High RR + Low P | No | 0.68 | 51573 |
| 51580 | Covington city, VA | 1.667 | 0.72 | High RR + Low P | No | 0.679 | 11178 |
| 27171 | Wright County, MN | 1.057 | 0.96 | Low RR + High P | No | 0.679 | 299268 |
| 53007 | Chelan County, WA | 1.133 | 0.92 | Low RR + High P | No | 0.679 | 160035 |
| 13137 | Habersham County, GA | 1.06 | 0.98 | Low RR + High P | No | 0.679 | 96222 |
| 47147 | Robertson County, TN | 1.394 | 0.72 | Low RR + Low P | No | 0.679 | 152174 |
| 18081 | Johnson County, IN | 1.172 | 0.86 | Low RR + High P | No | 0.678 | 333505 |
| 13161 | Jeff Davis County, GA | 1.522 | 0.74 | High RR + Low P | No | 0.678 | 29797 |
| 1017 | Chambers County, AL | 1.13 | 0.96 | Low RR + High P | No | 0.678 | 68243 |
| 48315 | Marion County, TX | 1.425 | 0.8 | Low RR + High P | No | 0.678 | 19121 |
| 8031 | Denver County, CO | 1.365 | 0.7 | Low RR + Low P | No | 0.678 | 1430030 |
| 51161 | Roanoke County, VA | 1.043 | 0.98 | Low RR + High P | No | 0.678 | 193782 |
| 21009 | Barren County, KY | 1.494 | 0.69 | High RR + Low P | No | 0.678 | 89880 |
| 55117 | Sheboygan County, WI | 1.26 | 0.8 | Low RR + Low P | No | 0.677 | 235413 |
| 47079 | Henry County, TN | 1.355 | 0.78 | Low RR + Low P | No | 0.677 | 64936 |
| 40015 | Caddo County, OK | 2.148 | 0.58 | High RR + Low P | No | 0.677 | 52451 |
| 46017 | Buffalo County, SD | 3.668 | 0.62 | High RR + Low P | No | 0.676 | 3738 |
| 45067 | Marion County, SC | 1.592 | 0.66 | High RR + Low P | No | 0.676 | 56979 |
| 16011 | Bingham County, ID | 1.436 | 0.71 | High RR + Low P | No | 0.676 | 100368 |
| 41013 | Crook County, OR | 1.207 | 0.9 | Low RR + High P | No | 0.676 | 53329 |
| 28023 | Clarke County, MS | 1.858 | 0.62 | High RR + Low P | No | 0.676 | 30431 |
| 32031 | Washoe County, NV | 1.129 | 0.88 | Low RR + High P | No | 0.675 | 995002 |
| 45081 | Saluda County, SC | 1.8 | 0.62 | High RR + Low P | No | 0.675 | 38107 |
| 28047 | Harrison County, MS | 1.863 | 0.54 | High RR + Low P | No | 0.675 | 421055 |
| 45027 | Clarendon County, SC | 1.569 | 0.67 | High RR + Low P | No | 0.675 | 61931 |
| 31069 | Garden County, NE | 1.745 | 0.71 | High RR + Low P | No | 0.675 | 3629 |
| 22051 | Jefferson Parish, LA | 1.054 | 0.94 | Low RR + High P | No | 0.675 | 847807 |
| 30017 | Custer County, MT | 1.355 | 0.83 | Low RR + High P | No | 0.674 | 23985 |
| 47065 | Hamilton County, TN | 1.084 | 0.92 | Low RR + High P | No | 0.674 | 754466 |
| 48365 | Panola County, TX | 1.76 | 0.62 | High RR + Low P | No | 0.674 | 45580 |
| 48469 | Victoria County, TX | 1.141 | 0.9 | Low RR + High P | No | 0.674 | 182738 |
| 19029 | Cass County, IA | 1.88 | 0.62 | High RR + Low P | No | 0.674 | 26267 |
| 48071 | Chambers County, TX | 1.195 | 0.86 | Low RR + High P | No | 0.674 | 105185 |
| 48101 | Cottle County, TX | 2.587 | 0.63 | High RR + Low P | No | 0.673 | 2611 |
| 13045 | Carroll County, GA | 1.1 | 0.92 | Low RR + High P | No | 0.673 | 251628 |
| 5093 | Mississippi County, AR | 1.31 | 0.79 | Low RR + Low P | No | 0.673 | 77526 |
| 13017 | Ben Hill County, GA | 1.293 | 0.85 | Low RR + High P | No | 0.673 | 34185 |
| 48389 | Reeves County, TX | 1.49 | 0.76 | High RR + Low P | No | 0.673 | 23462 |
| 55095 | Polk County, WI | 1.632 | 0.62 | High RR + Low P | No | 0.673 | 91308 |
| 2013 | Aleutians East Borough, AK | 1.423 | 0.82 | Low RR + High P | No | 0.673 | 6891 |
| 48055 | Caldwell County, TX | 1.34 | 0.76 | Low RR + Low P | No | 0.672 | 97708 |
| 27091 | Martin County, MN | 1.68 | 0.65 | High RR + Low P | No | 0.672 | 39313 |
| 31169 | Thayer County, NE | 1.449 | 0.8 | High RR + High P | No | 0.672 | 9683 |
| 45009 | Bamberg County, SC | 1.681 | 0.67 | High RR + Low P | No | 0.671 | 25924 |
| 41033 | Josephine County, OR | 1.25 | 0.8 | Low RR + Low P | No | 0.671 | 175621 |
| 31041 | Custer County, NE | 1.553 | 0.73 | High RR + Low P | No | 0.671 | 21085 |
| 53019 | Ferry County, WA | 1.263 | 0.92 | Low RR + High P | No | 0.671 | 14965 |
| 26069 | Iosco County, MI | 1.179 | 0.92 | Low RR + High P | No | 0.67 | 50846 |
| 37089 | Henderson County, NC | 0.984 | 0.99 | Low RR + High P | No | 0.67 | 237528 |
| 8009 | Baca County, CO | 1.402 | 0.83 | Low RR + High P | No | 0.67 | 6779 |
| 18135 | Randolph County, IN | 1.415 | 0.74 | Low RR + Low P | No | 0.669 | 48566 |
| 30049 | Lewis and Clark County, MT | 1.145 | 0.89 | Low RR + High P | No | 0.669 | 148795 |
| 54015 | Clay County, WV | 1.289 | 0.89 | Low RR + High P | No | 0.668 | 15623 |
| 54073 | Pleasants County, WV | 1.626 | 0.71 | High RR + Low P | No | 0.668 | 14998 |
| 39167 | Washington County, OH | 1.106 | 0.93 | Low RR + High P | No | 0.668 | 117445 |
| 13209 | Montgomery County, GA | 1.305 | 0.87 | Low RR + High P | No | 0.667 | 17404 |
| 12033 | Escambia County, FL | 1.007 | 0.96 | Low RR + High P | No | 0.667 | 650788 |
| 39009 | Athens County, OH | 1.224 | 0.82 | Low RR + High P | No | 0.667 | 123587 |
| 23001 | Androscoggin County, ME | 1.042 | 0.96 | Low RR + High P | No | 0.667 | 226990 |
| 31183 | Wheeler County, NE | 1.518 | 0.78 | High RR + Low P | No | 0.667 | 1557 |
| 27075 | Lake County, MN | 1.374 | 0.8 | Low RR + High P | No | 0.667 | 21767 |
| 46057 | Hamlin County, SD | 1.395 | 0.81 | Low RR + High P | No | 0.667 | 12845 |
| 41053 | Polk County, OR | 1.207 | 0.82 | Low RR + High P | No | 0.667 | 179398 |
| 8029 | Delta County, CO | 1.163 | 0.91 | Low RR + High P | No | 0.667 | 63306 |
| 32007 | Elko County, NV | 1.003 | 1 | Low RR + High P | No | 0.666 | 108272 |
| 48083 | Coleman County, TX | 1.274 | 0.9 | Low RR + High P | No | 0.666 | 15664 |
| 21169 | Metcalfe County, KY | 1.548 | 0.72 | High RR + Low P | No | 0.666 | 20890 |
| 47093 | Knox County, TN | 1.306 | 0.72 | Low RR + Low P | No | 0.666 | 996049 |
| 31057 | Dundy County, NE | 1.527 | 0.77 | High RR + Low P | No | 0.665 | 3156 |
| 19075 | Grundy County, IA | 1.335 | 0.82 | Low RR + High P | No | 0.665 | 24739 |
| 20045 | Douglas County, KS | 0.963 | 1 | Low RR + High P | No | 0.664 | 240560 |
| 30029 | Flathead County, MT | 0.951 | 1 | Low RR + High P | No | 0.664 | 225564 |
| 39127 | Perry County, OH | 1.831 | 0.57 | High RR + Low P | No | 0.664 | 71022 |
| 13031 | Bulloch County, GA | 1.023 | 0.97 | Low RR + High P | No | 0.663 | 167365 |
| 39109 | Miami County, OH | 1.34 | 0.72 | Low RR + Low P | No | 0.663 | 221089 |
| 27165 | Watonwan County, MN | 1.958 | 0.6 | High RR + Low P | No | 0.662 | 22183 |
| 54069 | Ohio County, WV | 1.08 | 0.95 | Low RR + High P | No | 0.661 | 82657 |
| 48393 | Roberts County, TX | 3.034 | 0.6 | High RR + Low P | No | 0.661 | 1652 |
| 27049 | Goodhue County, MN | 1.901 | 0.55 | High RR + Low P | No | 0.661 | 96076 |
| 38079 | Rolette County, ND | 1.624 | 0.67 | High RR + Low P | No | 0.661 | 23595 |
| 8005 | Arapahoe County, CO | 0.97 | 0.97 | Low RR + High P | No | 0.661 | 1312330 |
| 18111 | Newton County, IN | 1.322 | 0.82 | Low RR + High P | No | 0.661 | 27799 |
| 40133 | Seminole County, OK | 2.901 | 0.54 | High RR + Low P | No | 0.661 | 47029 |
| 45071 | Newberry County, SC | 1.291 | 0.78 | Low RR + Low P | No | 0.66 | 77140 |
| 25005 | Bristol County, MA | 0.912 | 1 | Low RR + High P | No | 0.66 | 1161585 |
| 12111 | St. Lucie County, FL | 0.92 | 1 | Low RR + High P | No | 0.66 | 732472 |
| 46111 | Sanborn County, SD | 1.801 | 0.66 | High RR + Low P | No | 0.66 | 4802 |
| 46081 | Lawrence County, SD | 1.105 | 0.95 | Low RR + High P | No | 0.66 | 55293 |
| 46095 | Mellette County, SD | 2.271 | 0.61 | High RR + Low P | No | 0.659 | 3724 |
| 27023 | Chippewa County, MN | 1.161 | 0.95 | Low RR + High P | No | 0.659 | 24441 |
| 19055 | Delaware County, IA | 1.591 | 0.66 | High RR + Low P | No | 0.659 | 35207 |
| 13295 | Walker County, GA | 1.218 | 0.8 | Low RR + High P | No | 0.659 | 138454 |
| 13055 | Chattooga County, GA | 1.409 | 0.72 | Low RR + Low P | No | 0.659 | 50184 |
| 40139 | Texas County, OK | 1.459 | 0.71 | High RR + Low P | No | 0.658 | 40876 |
| 49031 | Piute County, UT | 1.504 | 0.77 | High RR + Low P | No | 0.658 | 3043 |
| 35061 | Valencia County, NM | 1.011 | 0.97 | Low RR + High P | No | 0.658 | 157342 |
| 22025 | Catahoula Parish, LA | 1.351 | 0.82 | Low RR + High P | No | 0.658 | 17018 |
| 30011 | Carter County, MT | 1.664 | 0.7 | High RR + Low P | No | 0.657 | 2821 |
| 27127 | Redwood County, MN | 1.6 | 0.66 | High RR + Low P | No | 0.657 | 30646 |
| 37157 | Rockingham County, NC | 1.121 | 0.88 | Low RR + High P | No | 0.657 | 184484 |
| 38085 | Sioux County, ND | 1.93 | 0.63 | High RR + Low P | No | 0.657 | 7329 |
| 19165 | Shelby County, IA | 1.469 | 0.73 | High RR + Low P | No | 0.656 | 23517 |
| 40091 | McIntosh County, OK | 2.4 | 0.55 | High RR + Low P | No | 0.656 | 39006 |
| 50027 | Windsor County, VT | 1.441 | 0.66 | High RR + Low P | No | 0.656 | 116251 |
| 40063 | Hughes County, OK | 2.316 | 0.57 | High RR + Low P | No | 0.655 | 26870 |
| 28089 | Madison County, MS | 1.119 | 0.88 | Low RR + High P | No | 0.655 | 223782 |
| 21139 | Livingston County, KY | 1.288 | 0.85 | Low RR + High P | No | 0.655 | 17860 |
| 27013 | Blue Earth County, MN | 1.14 | 0.87 | Low RR + High P | No | 0.655 | 139609 |
| 1081 | Lee County, AL | 1.008 | 0.95 | Low RR + High P | No | 0.655 | 363913 |
| 30019 | Daniels County, MT | 1.777 | 0.66 | High RR + Low P | No | 0.655 | 3278 |
| 17101 | Lawrence County, IL | 1.749 | 0.61 | High RR + Low P | No | 0.655 | 29704 |
| 17019 | Champaign County, IL | 1.05 | 0.92 | Low RR + High P | No | 0.655 | 411813 |
| 42093 | Montour County, PA | 1.084 | 0.98 | Low RR + High P | No | 0.655 | 35914 |
| 48021 | Bastrop County, TX | 1.323 | 0.71 | Low RR + Low P | No | 0.654 | 217173 |
| 35013 | Dona Ana County, NM | 0.948 | 0.99 | Low RR + High P | No | 0.654 | 448740 |
| 19141 | O'Brien County, IA | 1.096 | 0.98 | Low RR + High P | No | 0.652 | 28097 |
| 27027 | Clay County, MN | 1.12 | 0.89 | Low RR + High P | No | 0.652 | 132118 |
| 54097 | Upshur County, WV | 1.237 | 0.83 | Low RR + High P | No | 0.652 | 47234 |
| 53053 | Pierce County, WA | 1.263 | 0.72 | Low RR + Low P | No | 0.652 | 1855175 |
| 50019 | Orleans County, VT | 2.029 | 0.55 | High RR + Low P | No | 0.651 | 55078 |
| 18031 | Decatur County, IN | 1.153 | 0.9 | Low RR + High P | No | 0.651 | 52853 |
| 5003 | Ashley County, AR | 1.104 | 0.96 | Low RR + High P | No | 0.651 | 36610 |
| 26101 | Manistee County, MI | 1.133 | 0.92 | Low RR + High P | No | 0.651 | 50927 |
| 41021 | Gilliam County, OR | 1.534 | 0.74 | High RR + Low P | No | 0.65 | 4034 |
| 17075 | Iroquois County, IL | 1.419 | 0.7 | Low RR + Low P | No | 0.649 | 52629 |
| 48077 | Clay County, TX | 1.616 | 0.65 | High RR + Low P | No | 0.648 | 21237 |
| 20199 | Wallace County, KS | 2.143 | 0.6 | High RR + Low P | No | 0.648 | 3002 |
| 35001 | Bernalillo County, NM | 0.888 | 1 | Low RR + High P | No | 0.648 | 1344543 |
| 29159 | Pettis County, MO | 1.039 | 0.96 | Low RR + High P | No | 0.648 | 86927 |
| 21221 | Trigg County, KY | 1.405 | 0.74 | Low RR + Low P | No | 0.647 | 28717 |
| 26127 | Oceana County, MI | 1.086 | 0.94 | Low RR + High P | No | 0.647 | 53901 |
| 51119 | Middlesex County, VA | 1.093 | 0.98 | Low RR + High P | No | 0.647 | 21876 |
| 31149 | Rock County, NE | 1.825 | 0.64 | High RR + Low P | No | 0.646 | 2512 |
| 51137 | Orange County, VA | 0.963 | 1 | Low RR + High P | No | 0.646 | 76629 |
| 48363 | Palo Pinto County, TX | 2.22 | 0.52 | High RR + Low P | No | 0.646 | 59069 |
| 48255 | Karnes County, TX | 1.67 | 0.62 | High RR + Low P | No | 0.645 | 29963 |
| 5095 | Monroe County, AR | 1.3 | 0.84 | Low RR + High P | No | 0.645 | 13080 |
| 31015 | Boyd County, NE | 2.197 | 0.6 | High RR + Low P | No | 0.645 | 3455 |
| 17169 | Schuyler County, IL | 1.29 | 0.84 | Low RR + High P | No | 0.644 | 13486 |
| 27017 | Carlton County, MN | 1.368 | 0.7 | Low RR + Low P | No | 0.644 | 73369 |
| 48367 | Parker County, TX | 1.214 | 0.76 | Low RR + Low P | No | 0.644 | 339432 |
| 37079 | Greene County, NC | 1.806 | 0.57 | High RR + Low P | No | 0.644 | 40897 |
| 29027 | Callaway County, MO | 1.068 | 0.92 | Low RR + High P | No | 0.644 | 89454 |
| 19143 | Osceola County, IA | 1.408 | 0.76 | Low RR + Low P | No | 0.644 | 12050 |
| 39027 | Clinton County, OH | 1.205 | 0.8 | Low RR + High P | No | 0.643 | 83828 |
| 26115 | Monroe County, MI | 0.989 | 0.95 | Low RR + High P | No | 0.643 | 310280 |
| 24013 | Carroll County, MD | 1.056 | 0.89 | Low RR + High P | No | 0.643 | 352111 |
| 20005 | Atchison County, KS | 1.289 | 0.8 | Low RR + Low P | No | 0.643 | 32069 |
| 47113 | Madison County, TN | 1.488 | 0.6 | High RR + Low P | No | 0.643 | 198365 |
| 18105 | Monroe County, IN | 1 | 0.94 | Low RR + High P | No | 0.643 | 278963 |
| 35017 | Grant County, NM | 1.018 | 0.98 | Low RR + High P | No | 0.643 | 55165 |
| 36017 | Chenango County, NY | 1.561 | 0.59 | High RR + Low P | No | 0.643 | 92251 |
| 20083 | Hodgeman County, KS | 1.808 | 0.63 | High RR + Low P | No | 0.642 | 3352 |
| 48435 | Sutton County, TX | 1.493 | 0.74 | High RR + Low P | No | 0.642 | 6453 |
| 47027 | Clay County, TN | 1.632 | 0.65 | High RR + Low P | No | 0.642 | 15370 |
| 48429 | Stephens County, TX | 1.62 | 0.65 | High RR + Low P | No | 0.642 | 18720 |
| 28143 | Tunica County, MS | 2.082 | 0.57 | High RR + Low P | No | 0.642 | 18641 |
| 19095 | Iowa County, IA | 1.331 | 0.77 | Low RR + Low P | No | 0.641 | 32878 |
| 31029 | Chase County, NE | 1.405 | 0.77 | Low RR + Low P | No | 0.641 | 7478 |
| 40057 | Harmon County, OK | 1.824 | 0.62 | High RR + Low P | No | 0.641 | 4808 |
| 1097 | Mobile County, AL | 1.02 | 0.9 | Low RR + High P | No | 0.641 | 823038 |
| 28039 | George County, MS | 1.055 | 0.96 | Low RR + High P | No | 0.641 | 50818 |
| 19043 | Clayton County, IA | 1.293 | 0.79 | Low RR + Low P | No | 0.641 | 34026 |
| 13125 | Glascock County, GA | 1.722 | 0.65 | High RR + Low P | No | 0.641 | 5882 |
| 12001 | Alachua County, FL | 0.906 | 0.99 | Low RR + High P | No | 0.64 | 570035 |
| 18167 | Vigo County, IN | 1.321 | 0.69 | Low RR + Low P | No | 0.64 | 212235 |
| 56001 | Albany County, WY | 1.005 | 0.97 | Low RR + High P | No | 0.64 | 76435 |
| 38095 | Towner County, ND | 1.644 | 0.67 | High RR + Low P | No | 0.64 | 4090 |
| 20103 | Leavenworth County, KS | 1.034 | 0.92 | Low RR + High P | No | 0.639 | 166440 |
| 48431 | Sterling County, TX | 1.845 | 0.62 | High RR + Low P | No | 0.639 | 2823 |
| 51081 | Greensville County, VA | 1.649 | 0.62 | High RR + Low P | No | 0.639 | 22337 |
| 2105 | Hoonah-Angoon Census Area, AK | 1.861 | 0.61 | High RR + Low P | No | 0.638 | 4541 |
| 15007 | Kauai County, HI | 0.968 | 0.97 | Low RR + High P | No | 0.637 | 147634 |
| 38091 | Steele County, ND | 1.384 | 0.78 | Low RR + Low P | No | 0.636 | 3575 |
| 20181 | Sherman County, KS | 1.356 | 0.78 | Low RR + Low P | No | 0.636 | 11720 |
| 17149 | Pike County, IL | 1.07 | 0.96 | Low RR + High P | No | 0.635 | 28841 |
| 51550 | Chesapeake city, VA | 1.009 | 0.91 | Low RR + High P | No | 0.635 | 506345 |
| 26027 | Cass County, MI | 1.151 | 0.83 | Low RR + High P | No | 0.635 | 103119 |
| 48079 | Cochran County, TX | 1.997 | 0.59 | High RR + Low P | No | 0.635 | 5031 |
| 39157 | Tuscarawas County, OH | 1.327 | 0.67 | Low RR + Low P | No | 0.634 | 183845 |
| 26145 | Saginaw County, MI | 1.351 | 0.64 | Low RR + Low P | No | 0.634 | 376155 |
| 48433 | Stonewall County, TX | 1.807 | 0.62 | High RR + Low P | No | 0.634 | 2436 |
| 4013 | Maricopa County, AZ | 0.85 | 1 | Low RR + High P | No | 0.634 | 9141704 |
| 29037 | Cass County, MO | 0.978 | 0.94 | Low RR + High P | No | 0.633 | 222089 |
| 48421 | Sherman County, TX | 1.866 | 0.6 | High RR + Low P | No | 0.633 | 5456 |
| 46085 | Lyman County, SD | 1.559 | 0.69 | High RR + Low P | No | 0.633 | 7410 |
| 42071 | Lancaster County, PA | 0.992 | 0.91 | Low RR + High P | No | 0.633 | 1115249 |
| 42001 | Adams County, PA | 1.012 | 0.92 | Low RR + High P | No | 0.633 | 212707 |
| 46049 | Faulk County, SD | 1.621 | 0.66 | High RR + Low P | No | 0.632 | 4273 |
| 48097 | Cooke County, TX | 1.353 | 0.68 | Low RR + Low P | No | 0.632 | 86830 |
| 38027 | Eddy County, ND | 1.443 | 0.74 | High RR + Low P | No | 0.632 | 4570 |
| 38075 | Renville County, ND | 1.706 | 0.64 | High RR + Low P | No | 0.632 | 4529 |
| 12085 | Martin County, FL | 0.871 | 1 | Low RR + High P | No | 0.631 | 325302 |
| 13257 | Stephens County, GA | 1.232 | 0.78 | Low RR + Low P | No | 0.631 | 53974 |
| 39175 | Wyandot County, OH | 1.317 | 0.74 | Low RR + Low P | No | 0.631 | 43009 |
| 45017 | Calhoun County, SC | 1.881 | 0.56 | High RR + Low P | No | 0.631 | 28383 |
| 13191 | McIntosh County, GA | 1.063 | 0.97 | Low RR + High P | No | 0.631 | 22684 |
| 48133 | Eastland County, TX | 1.241 | 0.8 | Low RR + High P | No | 0.63 | 35930 |
| 28037 | Franklin County, MS | 1.289 | 0.81 | Low RR + High P | No | 0.63 | 15221 |
| 20007 | Barber County, KS | 1.335 | 0.79 | Low RR + Low P | No | 0.63 | 8195 |
| 48481 | Wharton County, TX | 1.319 | 0.7 | Low RR + Low P | No | 0.63 | 83479 |
| 17179 | Tazewell County, IL | 1.313 | 0.66 | Low RR + Low P | No | 0.629 | 259151 |
| 48125 | Dickens County, TX | 1.896 | 0.6 | High RR + Low P | No | 0.629 | 3447 |
| 17083 | Jersey County, IL | 1.104 | 0.91 | Low RR + High P | No | 0.629 | 42282 |
| 19103 | Johnson County, IA | 1.359 | 0.63 | Low RR + Low P | No | 0.629 | 314443 |
| 13247 | Rockdale County, GA | 0.956 | 0.96 | Low RR + High P | No | 0.628 | 190932 |
| 45073 | Oconee County, SC | 1.393 | 0.62 | Low RR + Low P | No | 0.628 | 161381 |
| 2090 | Fairbanks North Star Borough, AK | 0.942 | 0.97 | Low RR + High P | No | 0.628 | 190042 |
| 47159 | Smith County, TN | 1.473 | 0.64 | High RR + Low P | No | 0.628 | 41008 |
| 31025 | Cass County, NE | 1.265 | 0.76 | Low RR + Low P | No | 0.627 | 54598 |
| 41009 | Columbia County, OR | 1.283 | 0.71 | Low RR + Low P | No | 0.626 | 107487 |
| 27019 | Carver County, MN | 0.996 | 0.92 | Low RR + High P | No | 0.626 | 221177 |
| 12013 | Calhoun County, FL | 1.295 | 0.77 | Low RR + Low P | No | 0.626 | 26935 |
| 48383 | Reagan County, TX | 2.486 | 0.56 | High RR + Low P | No | 0.626 | 6262 |
| 51173 | Smyth County, VA | 1.301 | 0.72 | Low RR + Low P | No | 0.625 | 58624 |
| 27041 | Douglas County, MN | 1.209 | 0.77 | Low RR + Low P | No | 0.625 | 79637 |
| 29111 | Lewis County, MO | 1.233 | 0.82 | Low RR + High P | No | 0.625 | 19704 |
| 27163 | Washington County, MN | 0.89 | 0.99 | Low RR + High P | No | 0.625 | 555105 |
| 46125 | Turner County, SD | 1.205 | 0.85 | Low RR + High P | No | 0.625 | 17915 |
| 55057 | Juneau County, WI | 1.598 | 0.58 | High RR + Low P | No | 0.625 | 53369 |
| 48165 | Gaines County, TX | 1.173 | 0.83 | Low RR + High P | No | 0.625 | 44663 |
| 49045 | Tooele County, UT | 0.982 | 0.93 | Low RR + High P | No | 0.623 | 162006 |
| 46021 | Campbell County, SD | 1.861 | 0.59 | High RR + Low P | No | 0.623 | 2695 |
| 13223 | Paulding County, GA | 1.035 | 0.87 | Low RR + High P | No | 0.623 | 361917 |
| 48269 | King County, TX | 1.481 | 0.71 | High RR + Low P | No | 0.623 | 451 |
| 47057 | Grainger County, TN | 1.269 | 0.75 | Low RR + Low P | No | 0.622 | 48866 |
| 27107 | Norman County, MN | 1.577 | 0.64 | High RR + Low P | No | 0.622 | 12700 |
| 19053 | Decatur County, IA | 1.309 | 0.78 | Low RR + Low P | No | 0.622 | 15360 |
| 51001 | Accomack County, VA | 1.159 | 0.82 | Low RR + High P | No | 0.621 | 66462 |
| 24043 | Washington County, MD | 1.599 | 0.52 | High RR + Low P | No | 0.62 | 311070 |
| 53077 | Yakima County, WA | 1.389 | 0.59 | Low RR + Low P | No | 0.62 | 513662 |
| 49009 | Daggett County, UT | 2.043 | 0.57 | High RR + Low P | No | 0.62 | 2007 |
| 39065 | Hardin County, OH | 1.411 | 0.64 | Low RR + Low P | No | 0.62 | 60759 |
| 4001 | Apache County, AZ | 1.214 | 0.73 | Low RR + Low P | No | 0.62 | 130569 |
| 19025 | Calhoun County, IA | 1.374 | 0.72 | Low RR + Low P | No | 0.62 | 19488 |
| 47157 | Shelby County, TN | 1.293 | 0.63 | Low RR + Low P | No | 0.62 | 1826399 |
| 20111 | Lyon County, KS | 1.093 | 0.88 | Low RR + High P | No | 0.62 | 64203 |
| 18075 | Jay County, IN | 1.203 | 0.8 | Low RR + High P | No | 0.62 | 40150 |
| 8017 | Cheyenne County, CO | 1.913 | 0.58 | High RR + Low P | No | 0.619 | 3458 |
| 17063 | Grundy County, IL | 1.186 | 0.76 | Low RR + Low P | No | 0.619 | 106727 |
| 54105 | Wirt County, WV | 1.362 | 0.76 | Low RR + Low P | No | 0.619 | 10092 |
| 16019 | Bonneville County, ID | 1.008 | 0.89 | Low RR + High P | No | 0.619 | 260909 |
| 5141 | Van Buren County, AR | 0.989 | 0.99 | Low RR + High P | No | 0.619 | 32245 |
| 31063 | Frontier County, NE | 1.272 | 0.82 | Low RR + High P | No | 0.618 | 5196 |
| 19185 | Wayne County, IA | 1.692 | 0.6 | High RR + Low P | No | 0.618 | 13047 |
| 44009 | Washington County, RI | 1.037 | 0.86 | Low RR + High P | No | 0.618 | 260238 |
| 50009 | Essex County, VT | 1.445 | 0.7 | High RR + Low P | No | 0.618 | 12007 |
| 51037 | Charlotte County, VA | 1.242 | 0.8 | Low RR + High P | No | 0.618 | 22736 |
| 28043 | Grenada County, MS | 1.427 | 0.64 | Low RR + Low P | No | 0.617 | 42199 |
| 48013 | Atascosa County, TX | 1.611 | 0.54 | High RR + Low P | No | 0.617 | 102520 |
| 31121 | Merrick County, NE | 1.221 | 0.83 | Low RR + High P | No | 0.617 | 15470 |
| 37119 | Mecklenburg County, NC | 0.808 | 1 | Low RR + High P | No | 0.617 | 2307776 |
| 47143 | Rhea County, TN | 1.316 | 0.69 | Low RR + Low P | No | 0.616 | 67613 |
| 13281 | Towns County, GA | 1.091 | 0.92 | Low RR + High P | No | 0.616 | 25996 |
| 20165 | Rush County, KS | 1.328 | 0.77 | Low RR + Low P | No | 0.615 | 5771 |
| 20047 | Edwards County, KS | 1.768 | 0.59 | High RR + Low P | No | 0.615 | 5493 |
| 31107 | Knox County, NE | 1.162 | 0.88 | Low RR + High P | No | 0.615 | 16605 |
| 42107 | Schuylkill County, PA | 2.14 | 0.37 | High RR + Low P | No | 0.614 | 286974 |
| 54013 | Calhoun County, WV | 1.316 | 0.77 | Low RR + Low P | No | 0.614 | 12028 |
| 13077 | Coweta County, GA | 1.174 | 0.73 | Low RR + Low P | No | 0.614 | 308771 |
| 51670 | Hopewell city, VA | 1.061 | 0.91 | Low RR + High P | No | 0.614 | 45542 |
| 54107 | Wood County, WV | 1.315 | 0.64 | Low RR + Low P | No | 0.614 | 166440 |
| 38047 | Logan County, ND | 1.707 | 0.61 | High RR + Low P | No | 0.614 | 3707 |
| 55035 | Eau Claire County, WI | 1.185 | 0.73 | Low RR + Low P | No | 0.614 | 214689 |
| 26153 | Schoolcraft County, MI | 1.201 | 0.84 | Low RR + High P | No | 0.613 | 16323 |
| 21005 | Anderson County, KY | 1.821 | 0.52 | High RR + Low P | No | 0.613 | 48886 |
| 31085 | Hayes County, NE | 1.835 | 0.58 | High RR + Low P | No | 0.613 | 1710 |
| 30051 | Liberty County, MT | 1.677 | 0.61 | High RR + Low P | No | 0.613 | 3948 |
| 2070 | Dillingham Census Area, AK | 1.71 | 0.59 | High RR + Low P | No | 0.612 | 9331 |
| 48049 | Brown County, TX | 1.369 | 0.63 | Low RR + Low P | No | 0.612 | 77152 |
| 41003 | Benton County, OR | 0.975 | 0.92 | Low RR + High P | No | 0.612 | 195374 |
| 28061 | Jasper County, MS | 1.524 | 0.61 | High RR + Low P | No | 0.612 | 32090 |
| 22043 | Grant Parish, LA | 1.088 | 0.89 | Low RR + High P | No | 0.611 | 43951 |
| 5033 | Crawford County, AR | 0.941 | 0.96 | Low RR + High P | No | 0.611 | 122932 |
| 20029 | Cloud County, KS | 1.174 | 0.85 | Low RR + High P | No | 0.611 | 17788 |
| 22031 | De Soto Parish, LA | 1.22 | 0.76 | Low RR + Low P | No | 0.61 | 53970 |
| 37073 | Gates County, NC | 1.324 | 0.74 | Low RR + Low P | No | 0.61 | 20713 |
| 13083 | Dade County, GA | 1.155 | 0.84 | Low RR + High P | No | 0.61 | 32255 |
| 46119 | Sully County, SD | 1.908 | 0.57 | High RR + Low P | No | 0.61 | 2976 |
| 8081 | Moffat County, CO | 1.111 | 0.9 | Low RR + High P | No | 0.61 | 26532 |
| 37031 | Carteret County, NC | 1.076 | 0.82 | Low RR + High P | No | 0.61 | 138941 |
| 36117 | Wayne County, NY | 0.851 | 1 | Low RR + High P | No | 0.609 | 181787 |
| 18153 | Sullivan County, IN | 1.026 | 0.94 | Low RR + High P | No | 0.609 | 41428 |
| 8039 | Elbert County, CO | 0.998 | 0.94 | Low RR + High P | No | 0.609 | 56648 |
| 31001 | Adams County, NE | 1.114 | 0.83 | Low RR + High P | No | 0.609 | 61846 |
| 6035 | Lassen County, CA | 1.174 | 0.78 | Low RR + Low P | No | 0.609 | 58881 |
| 48503 | Young County, TX | 1.458 | 0.62 | High RR + Low P | No | 0.608 | 36136 |
| 31171 | Thomas County, NE | 1.634 | 0.62 | High RR + Low P | No | 0.608 | 1342 |
| 13149 | Heard County, GA | 1.316 | 0.73 | Low RR + Low P | No | 0.608 | 23750 |
| 26133 | Osceola County, MI | 1.178 | 0.79 | Low RR + Low P | No | 0.608 | 46609 |
| 46097 | Miner County, SD | 1.635 | 0.61 | High RR + Low P | No | 0.607 | 4565 |
| 37069 | Franklin County, NC | 1.089 | 0.81 | Low RR + High P | No | 0.607 | 151401 |
| 37171 | Surry County, NC | 1.277 | 0.66 | Low RR + Low P | No | 0.607 | 142944 |
| 20085 | Jackson County, KS | 1.255 | 0.77 | Low RR + Low P | No | 0.607 | 26660 |
| 39003 | Allen County, OH | 1.34 | 0.61 | Low RR + Low P | No | 0.607 | 201935 |
| 47067 | Hancock County, TN | 1.207 | 0.82 | Low RR + High P | No | 0.607 | 13802 |
| 13297 | Walton County, GA | 1.108 | 0.78 | Low RR + Low P | No | 0.606 | 209766 |
| 19173 | Taylor County, IA | 1.307 | 0.76 | Low RR + Low P | No | 0.606 | 11799 |
| 28101 | Newton County, MS | 0.948 | 0.99 | Low RR + High P | No | 0.606 | 42042 |
| 19107 | Keokuk County, IA | 1.11 | 0.9 | Low RR + High P | No | 0.606 | 19797 |
| 27097 | Morrison County, MN | 1.485 | 0.58 | High RR + Low P | No | 0.606 | 68487 |
| 37103 | Jones County, NC | 1.77 | 0.56 | High RR + Low P | No | 0.606 | 18620 |
| 55133 | Waukesha County, WI | 0.904 | 0.94 | Low RR + High P | No | 0.605 | 822864 |
| 17113 | McLean County, IL | 1.027 | 0.84 | Low RR + High P | No | 0.605 | 341359 |
| 51720 | Norton city, VA | 1.388 | 0.72 | Low RR + Low P | No | 0.605 | 7081 |
| 51195 | Wise County, VA | 1.06 | 0.87 | Low RR + High P | No | 0.605 | 70501 |
| 21047 | Christian County, KY | 1.178 | 0.73 | Low RR + Low P | No | 0.605 | 144318 |
| 37141 | Pender County, NC | 0.987 | 0.9 | Low RR + High P | No | 0.604 | 134219 |
| 19033 | Cerro Gordo County, IA | 1.106 | 0.81 | Low RR + High P | No | 0.604 | 84832 |
| 51073 | Gloucester County, VA | 1.165 | 0.77 | Low RR + Low P | No | 0.604 | 79607 |
| 49049 | Utah County, UT | 0.906 | 0.93 | Low RR + High P | No | 0.604 | 1421866 |
| 8057 | Jackson County, CO | 1.526 | 0.65 | High RR + Low P | No | 0.604 | 2623 |
| 1047 | Dallas County, AL | 1.185 | 0.76 | Low RR + Low P | No | 0.603 | 72894 |
| 50005 | Caledonia County, VT | 1.614 | 0.54 | High RR + Low P | No | 0.603 | 61197 |
| 48095 | Concho County, TX | 1.44 | 0.68 | High RR + Low P | No | 0.603 | 6664 |
| 41069 | Wheeler County, OR | 1.436 | 0.7 | High RR + Low P | No | 0.603 | 2871 |
| 21065 | Estill County, KY | 1.144 | 0.84 | Low RR + High P | No | 0.603 | 27960 |
| 24031 | Montgomery County, MD | 0.761 | 1 | Low RR + High P | No | 0.602 | 2111541 |
| 48065 | Carson County, TX | 1.247 | 0.79 | Low RR + Low P | No | 0.602 | 11694 |
| 21007 | Ballard County, KY | 1.287 | 0.76 | Low RR + Low P | No | 0.602 | 15224 |
| 41071 | Yamhill County, OR | 1.424 | 0.56 | Low RR + Low P | No | 0.601 | 217006 |
| 21073 | Franklin County, KY | 1.171 | 0.75 | Low RR + Low P | No | 0.601 | 103192 |
| 55049 | Iowa County, WI | 1.363 | 0.65 | Low RR + Low P | No | 0.601 | 47914 |
| 31019 | Buffalo County, NE | 1.124 | 0.79 | Low RR + Low P | No | 0.601 | 101229 |
| 16025 | Camas County, ID | 1.443 | 0.69 | High RR + Low P | No | 0.601 | 2401 |
| 27169 | Winona County, MN | 1.302 | 0.65 | Low RR + Low P | No | 0.601 | 99190 |
| 20093 | Kearny County, KS | 1.478 | 0.65 | High RR + Low P | No | 0.601 | 7688 |
| 5079 | Lincoln County, AR | 1.278 | 0.74 | Low RR + Low P | No | 0.6 | 25808 |
| 20153 | Rawlins County, KS | 1.413 | 0.7 | Low RR + Low P | No | 0.6 | 4986 |
| 21053 | Clinton County, KY | 1.176 | 0.83 | Low RR + High P | No | 0.6 | 18287 |
| 24009 | Calvert County, MD | 1.155 | 0.74 | Low RR + Low P | No | 0.6 | 189287 |
| 48467 | Van Zandt County, TX | 0.951 | 0.93 | Low RR + High P | No | 0.6 | 126888 |
| 48491 | Williamson County, TX | 0.904 | 0.92 | Low RR + High P | No | 0.599 | 1369464 |
| 45023 | Chester County, SC | 1.227 | 0.72 | Low RR + Low P | No | 0.599 | 64219 |
| 8069 | Larimer County, CO | 0.809 | 0.99 | Low RR + High P | No | 0.599 | 738348 |
| 33001 | Belknap County, NH | 0.962 | 0.92 | Low RR + High P | No | 0.599 | 129732 |
| 8099 | Prowers County, CO | 1.321 | 0.71 | Low RR + Low P | No | 0.599 | 23624 |
| 46061 | Hanson County, SD | 1.779 | 0.56 | High RR + Low P | No | 0.599 | 6950 |
| 37021 | Buncombe County, NC | 0.931 | 0.91 | Low RR + High P | No | 0.598 | 549691 |
| 21033 | Caldwell County, KY | 1.409 | 0.65 | Low RR + Low P | No | 0.598 | 25159 |
| 31047 | Dawson County, NE | 1.154 | 0.79 | Low RR + Low P | No | 0.598 | 47990 |
| 47111 | Macon County, TN | 2.029 | 0.44 | High RR + Low P | No | 0.598 | 53007 |
| 48475 | Ward County, TX | 1.539 | 0.59 | High RR + Low P | No | 0.598 | 21914 |
| 18125 | Pike County, IN | 1.644 | 0.56 | High RR + Low P | No | 0.598 | 24249 |
| 12007 | Bradford County, FL | 1.182 | 0.76 | Low RR + Low P | No | 0.598 | 55154 |
| 46059 | Hand County, SD | 1.617 | 0.6 | High RR + Low P | No | 0.597 | 6219 |
| 21035 | Calloway County, KY | 1.197 | 0.73 | Low RR + Low P | No | 0.597 | 76373 |
| 51840 | Winchester city, VA | 1.04 | 0.89 | Low RR + High P | No | 0.597 | 55478 |
| 13319 | Wilkinson County, GA | 1.162 | 0.84 | Low RR + High P | No | 0.597 | 17402 |
| 48263 | Kent County, TX | 3.296 | 0.5 | High RR + Low P | No | 0.597 | 1478 |
| 13263 | Talbot County, GA | 1.364 | 0.71 | Low RR + Low P | No | 0.597 | 11475 |
| 13279 | Toombs County, GA | 1.527 | 0.56 | High RR + Low P | No | 0.596 | 53853 |
| 21175 | Morgan County, KY | 1.386 | 0.65 | Low RR + Low P | No | 0.596 | 28429 |
| 19089 | Howard County, IA | 1.471 | 0.62 | High RR + Low P | No | 0.596 | 18885 |
| 13233 | Polk County, GA | 0.963 | 0.92 | Low RR + High P | No | 0.595 | 87892 |
| 18065 | Henry County, IN | 1.235 | 0.69 | Low RR + Low P | No | 0.595 | 97744 |
| 19113 | Linn County, IA | 1.012 | 0.82 | Low RR + High P | No | 0.595 | 457912 |
| 12055 | Highlands County, FL | 0.807 | 1 | Low RR + High P | No | 0.595 | 213263 |
| 16079 | Shoshone County, ID | 1.056 | 0.91 | Low RR + High P | No | 0.594 | 27961 |
| 51650 | Hampton city, VA | 1.325 | 0.59 | Low RR + Low P | No | 0.594 | 274956 |
| 41017 | Deschutes County, OR | 1.002 | 0.83 | Low RR + High P | No | 0.594 | 414996 |
| 55047 | Green Lake County, WI | 1.57 | 0.56 | High RR + Low P | No | 0.594 | 38552 |
| 13261 | Sumter County, GA | 1.073 | 0.84 | Low RR + High P | No | 0.594 | 57754 |
| 27053 | Hennepin County, MN | 1.016 | 0.8 | Low RR + Low P | No | 0.594 | 2515779 |
| 20025 | Clark County, KS | 1.74 | 0.57 | High RR + Low P | No | 0.593 | 3786 |
| 17061 | Greene County, IL | 1.322 | 0.7 | Low RR + Low P | No | 0.593 | 23183 |
| 21187 | Owen County, KY | 1.412 | 0.64 | Low RR + Low P | No | 0.593 | 22607 |
| 48447 | Throckmorton County, TX | 1.942 | 0.54 | High RR + Low P | No | 0.593 | 3065 |
| 12023 | Columbia County, FL | 1.071 | 0.8 | Low RR + Low P | No | 0.592 | 144933 |
| 17181 | Union County, IL | 0.99 | 0.94 | Low RR + High P | No | 0.592 | 33439 |
| 1089 | Madison County, AL | 0.755 | 1 | Low RR + High P | No | 0.592 | 816205 |
| 51820 | Waynesboro city, VA | 1.201 | 0.74 | Low RR + Low P | No | 0.592 | 45981 |
| 16087 | Washington County, ID | 1.063 | 0.91 | Low RR + High P | No | 0.592 | 22572 |
| 47139 | Polk County, TN | 1.28 | 0.7 | Low RR + Low P | No | 0.591 | 35811 |
| 40053 | Grant County, OK | 1.433 | 0.66 | High RR + Low P | No | 0.591 | 8205 |
| 38037 | Grant County, ND | 1.46 | 0.65 | High RR + Low P | No | 0.591 | 4453 |
| 12093 | Okeechobee County, FL | 0.987 | 0.9 | Low RR + High P | No | 0.591 | 81800 |
| 2275 | Wrangell City and Borough, AK | 1.702 | 0.57 | High RR + Low P | No | 0.59 | 4135 |
| 29055 | Crawford County, MO | 1.203 | 0.74 | Low RR + Low P | No | 0.59 | 45378 |
| 17033 | Crawford County, IL | 1.519 | 0.57 | High RR + Low P | No | 0.59 | 36795 |
| 48395 | Robertson County, TX | 1.887 | 0.48 | High RR + Low P | No | 0.59 | 34397 |
| 19139 | Muscatine County, IA | 1.166 | 0.74 | Low RR + Low P | No | 0.59 | 84613 |
| 48329 | Midland County, TX | 0.97 | 0.85 | Low RR + High P | No | 0.59 | 349127 |
| 42069 | Lackawanna County, PA | 0.871 | 0.95 | Low RR + High P | No | 0.59 | 431873 |
| 27077 | Lake of the Woods County, MN | 1.603 | 0.59 | High RR + Low P | No | 0.59 | 7664 |
| 8115 | Sedgwick County, CO | 1.391 | 0.69 | Low RR + Low P | No | 0.589 | 4585 |
| 37061 | Duplin County, NC | 1.639 | 0.48 | High RR + Low P | No | 0.589 | 98454 |
| 5047 | Franklin County, AR | 1.101 | 0.84 | Low RR + High P | No | 0.589 | 34738 |
| 55005 | Barron County, WI | 1.37 | 0.59 | Low RR + Low P | No | 0.589 | 93548 |
| 41055 | Sherman County, OR | 1.752 | 0.56 | High RR + Low P | No | 0.589 | 3898 |
| 47077 | Henderson County, TN | 1.825 | 0.45 | High RR + Low P | No | 0.589 | 55977 |
| 48067 | Cass County, TX | 1.379 | 0.6 | Low RR + Low P | No | 0.589 | 57197 |
| 13307 | Webster County, GA | 1.267 | 0.77 | Low RR + Low P | No | 0.588 | 4674 |
| 56031 | Platte County, WY | 1.112 | 0.87 | Low RR + High P | No | 0.588 | 17198 |
| 36013 | Chautauqua County, NY | 1.275 | 0.61 | Low RR + Low P | No | 0.588 | 250235 |
| 12069 | Lake County, FL | 0.729 | 1 | Low RR + High P | No | 0.587 | 834552 |
| 16051 | Jefferson County, ID | 1.009 | 0.88 | Low RR + High P | No | 0.587 | 67534 |
| 55135 | Waupaca County, WI | 1.933 | 0.37 | High RR + Low P | No | 0.587 | 102898 |
| 12095 | Orange County, FL | 0.846 | 0.94 | Low RR + High P | No | 0.587 | 2923177 |
| 48317 | Martin County, TX | 1.473 | 0.62 | High RR + Low P | No | 0.587 | 10444 |
| 31071 | Garfield County, NE | 1.556 | 0.6 | High RR + Low P | No | 0.587 | 3550 |
| 46121 | Todd County, SD | 1.591 | 0.57 | High RR + Low P | No | 0.587 | 18432 |
| 46035 | Davison County, SD | 1.33 | 0.65 | Low RR + Low P | No | 0.587 | 39829 |
| 27125 | Red Lake County, MN | 1.43 | 0.65 | Low RR + Low P | No | 0.586 | 7792 |
| 17071 | Henderson County, IL | 1.414 | 0.65 | Low RR + Low P | No | 0.586 | 12256 |
| 37113 | Macon County, NC | 0.981 | 0.9 | Low RR + High P | No | 0.586 | 76381 |
| 31087 | Hitchcock County, NE | 1.396 | 0.68 | Low RR + Low P | No | 0.585 | 5166 |
| 42105 | Potter County, PA | 1.043 | 0.89 | Low RR + High P | No | 0.584 | 32205 |
| 8003 | Alamosa County, CO | 1.193 | 0.75 | Low RR + Low P | No | 0.584 | 33242 |
| 38063 | Nelson County, ND | 1.583 | 0.59 | High RR + Low P | No | 0.583 | 5990 |
| 46041 | Dewey County, SD | 1.639 | 0.56 | High RR + Low P | No | 0.583 | 10350 |
| 12063 | Jackson County, FL | 1.136 | 0.75 | Low RR + Low P | No | 0.583 | 96877 |
| 30075 | Powder River County, MT | 1.493 | 0.62 | High RR + Low P | No | 0.582 | 3475 |
| 48265 | Kerr County, TX | 1.014 | 0.83 | Low RR + High P | No | 0.581 | 107699 |
| 31159 | Seward County, NE | 1.126 | 0.8 | Low RR + High P | No | 0.581 | 35296 |
| 31143 | Polk County, NE | 1.24 | 0.76 | Low RR + Low P | No | 0.581 | 10468 |
| 28125 | Sharkey County, MS | 1.577 | 0.59 | High RR + Low P | No | 0.581 | 6798 |
| 12047 | Hamilton County, FL | 1.269 | 0.71 | Low RR + Low P | No | 0.58 | 26665 |
| 48169 | Garza County, TX | 1.339 | 0.7 | Low RR + Low P | No | 0.58 | 9015 |
| 16007 | Bear Lake County, ID | 1.141 | 0.83 | Low RR + High P | No | 0.58 | 13496 |
| 23017 | Oxford County, ME | 0.832 | 0.98 | Low RR + High P | No | 0.58 | 119363 |
| 45019 | Charleston County, SC | 1.197 | 0.63 | Low RR + Low P | No | 0.58 | 843717 |
| 20003 | Anderson County, KS | 1.284 | 0.72 | Low RR + Low P | No | 0.58 | 15636 |
| 54045 | Logan County, WV | 1.77 | 0.44 | High RR + Low P | No | 0.58 | 62033 |
| 38013 | Burke County, ND | 1.764 | 0.55 | High RR + Low P | No | 0.58 | 4266 |
| 48425 | Somervell County, TX | 1.126 | 0.83 | Low RR + High P | No | 0.579 | 19685 |
| 26119 | Montmorency County, MI | 1.37 | 0.65 | Low RR + Low P | No | 0.579 | 19235 |
| 31055 | Douglas County, NE | 1.138 | 0.69 | Low RR + Low P | No | 0.579 | 1175646 |
| 30025 | Fallon County, MT | 1.521 | 0.6 | High RR + Low P | No | 0.579 | 6044 |
| 29015 | Benton County, MO | 1.009 | 0.89 | Low RR + High P | No | 0.578 | 40785 |
| 31049 | Deuel County, NE | 1.74 | 0.55 | High RR + Low P | No | 0.578 | 3772 |
| 49021 | Iron County, UT | 0.875 | 0.95 | Low RR + High P | No | 0.578 | 126719 |
| 45003 | Aiken County, SC | 0.946 | 0.86 | Low RR + High P | No | 0.578 | 351358 |
| 20081 | Haskell County, KS | 1.522 | 0.6 | High RR + Low P | No | 0.577 | 7225 |
| 38041 | Hettinger County, ND | 1.521 | 0.6 | High RR + Low P | No | 0.577 | 4826 |
| 48011 | Armstrong County, TX | 1.982 | 0.51 | High RR + Low P | No | 0.577 | 3689 |
| 48413 | Schleicher County, TX | 1.813 | 0.53 | High RR + Low P | No | 0.577 | 4750 |
| 19181 | Warren County, IA | 0.859 | 0.97 | Low RR + High P | No | 0.577 | 109508 |
| 21205 | Rowan County, KY | 1.734 | 0.46 | High RR + Low P | No | 0.576 | 48781 |
| 48211 | Hemphill County, TX | 1.552 | 0.59 | High RR + Low P | No | 0.576 | 6414 |
| 20017 | Chase County, KS | 1.491 | 0.61 | High RR + Low P | No | 0.576 | 5147 |
| 46031 | Corson County, SD | 1.884 | 0.51 | High RR + Low P | No | 0.576 | 7599 |
| 31007 | Banner County, NE | 1.281 | 0.75 | Low RR + Low P | No | 0.576 | 1331 |
| 39083 | Knox County, OH | 1.157 | 0.7 | Low RR + Low P | No | 0.575 | 126516 |
| 26123 | Newaygo County, MI | 1.251 | 0.63 | Low RR + Low P | No | 0.575 | 101836 |
| 31073 | Gosper County, NE | 1.53 | 0.6 | High RR + Low P | No | 0.575 | 3682 |
| 13227 | Pickens County, GA | 0.832 | 0.99 | Low RR + High P | No | 0.575 | 70794 |
| 20187 | Stanton County, KS | 1.75 | 0.54 | High RR + Low P | No | 0.575 | 3868 |
| 48063 | Camp County, TX | 1.208 | 0.74 | Low RR + Low P | No | 0.575 | 25721 |
| 47123 | Monroe County, TN | 1.206 | 0.66 | Low RR + Low P | No | 0.575 | 96274 |
| 28049 | Hinds County, MS | 2.089 | 0.2 | High RR + Low P | No | 0.574 | 432425 |
| 40059 | Harper County, OK | 1.345 | 0.69 | Low RR + Low P | No | 0.574 | 6355 |
| 21201 | Robertson County, KY | 1.789 | 0.53 | High RR + Low P | No | 0.574 | 4560 |
| 29187 | St. Francois County, MO | 0.838 | 0.97 | Low RR + High P | No | 0.574 | 133964 |
| 20035 | Cowley County, KS | 0.981 | 0.88 | Low RR + High P | No | 0.573 | 68580 |
| 12015 | Charlotte County, FL | 0.691 | 1 | Low RR + High P | No | 0.573 | 408716 |
| 1061 | Geneva County, AL | 1.225 | 0.68 | Low RR + Low P | No | 0.572 | 53748 |
| 21135 | Lewis County, KY | 1.83 | 0.47 | High RR + Low P | No | 0.572 | 25910 |
| 38077 | Richland County, ND | 1.303 | 0.65 | Low RR + Low P | No | 0.572 | 33132 |
| 19021 | Buena Vista County, IA | 1.187 | 0.72 | Low RR + Low P | No | 0.572 | 41211 |
| 19189 | Winnebago County, IA | 1.094 | 0.84 | Low RR + High P | No | 0.572 | 21210 |
| 41025 | Harney County, OR | 1.074 | 0.87 | Low RR + High P | No | 0.571 | 14972 |
| 28155 | Webster County, MS | 2.337 | 0.41 | High RR + Low P | No | 0.57 | 19932 |
| 22103 | St. Tammany Parish, LA | 0.672 | 1 | Low RR + High P | No | 0.57 | 548820 |
| 21229 | Washington County, KY | 1.192 | 0.75 | Low RR + Low P | No | 0.57 | 24327 |
| 20023 | Cheyenne County, KS | 1.321 | 0.7 | Low RR + Low P | No | 0.57 | 5254 |
| 35039 | Rio Arriba County, NM | 0.911 | 0.93 | Low RR + High P | No | 0.57 | 79867 |
| 48373 | Polk County, TX | 1.459 | 0.52 | High RR + Low P | No | 0.569 | 107442 |
| 5133 | Sevier County, AR | 1.067 | 0.83 | Low RR + High P | No | 0.569 | 31370 |
| 6023 | Humboldt County, CA | 1.82 | 0.28 | High RR + Low P | No | 0.569 | 268929 |
| 41007 | Clatsop County, OR | 1.135 | 0.73 | Low RR + Low P | No | 0.569 | 82754 |
| 45013 | Beaufort County, SC | 0.676 | 1 | Low RR + High P | No | 0.569 | 395126 |
| 48057 | Calhoun County, TX | 1.182 | 0.72 | Low RR + Low P | No | 0.569 | 39378 |
| 51191 | Washington County, VA | 0.867 | 0.95 | Low RR + High P | No | 0.569 | 107905 |
| 51750 | Radford city, VA | 1.122 | 0.78 | Low RR + Low P | No | 0.568 | 33690 |
| 39059 | Guernsey County, OH | 1.268 | 0.62 | Low RR + Low P | No | 0.568 | 76149 |
| 26021 | Berrien County, MI | 1.145 | 0.67 | Low RR + Low P | No | 0.568 | 305096 |
| 42119 | Union County, PA | 1.153 | 0.71 | Low RR + Low P | No | 0.567 | 84732 |
| 8061 | Kiowa County, CO | 1.721 | 0.54 | High RR + Low P | No | 0.567 | 2818 |
| 51775 | Salem city, VA | 1.317 | 0.61 | Low RR + Low P | No | 0.567 | 51300 |
| 33013 | Merrimack County, NH | 0.974 | 0.81 | Low RR + High P | No | 0.567 | 313562 |
| 54009 | Brooke County, WV | 0.999 | 0.88 | Low RR + High P | No | 0.567 | 43067 |
| 48303 | Lubbock County, TX | 1.375 | 0.51 | Low RR + Low P | No | 0.567 | 638701 |
| 28041 | Greene County, MS | 2.661 | 0.36 | High RR + Low P | No | 0.567 | 27167 |
| 46037 | Day County, SD | 1.258 | 0.72 | Low RR + Low P | No | 0.567 | 10926 |
| 13085 | Dawson County, GA | 1.049 | 0.8 | Low RR + High P | No | 0.567 | 61921 |
| 56027 | Niobrara County, WY | 1.348 | 0.67 | Low RR + Low P | No | 0.566 | 4702 |
| 40083 | Logan County, OK | 1.784 | 0.35 | High RR + Low P | No | 0.566 | 104953 |
| 55027 | Dodge County, WI | 1.589 | 0.42 | High RR + Low P | No | 0.566 | 176605 |
| 31151 | Saline County, NE | 1.366 | 0.61 | Low RR + Low P | No | 0.566 | 29103 |
| 46043 | Douglas County, SD | 1.331 | 0.68 | Low RR + Low P | No | 0.566 | 5625 |
| 31147 | Richardson County, NE | 1.135 | 0.81 | Low RR + High P | No | 0.566 | 15419 |
| 47185 | White County, TN | 1.067 | 0.79 | Low RR + Low P | No | 0.566 | 56692 |
| 27005 | Becker County, MN | 1.143 | 0.72 | Low RR + Low P | No | 0.566 | 70646 |
| 1009 | Blount County, AL | 0.818 | 0.97 | Low RR + High P | No | 0.565 | 119332 |
| 54057 | Mineral County, WV | 1.382 | 0.57 | Low RR + Low P | No | 0.565 | 53769 |
| 21131 | Leslie County, KY | 2.703 | 0.37 | High RR + Low P | No | 0.565 | 19913 |
| 20049 | Elk County, KS | 1.442 | 0.61 | High RR + Low P | No | 0.565 | 4915 |
| 24023 | Garrett County, MD | 1.675 | 0.44 | High RR + Low P | No | 0.564 | 57011 |
| 46047 | Fall River County, SD | 1.317 | 0.67 | Low RR + Low P | No | 0.564 | 14790 |
| 13091 | Dodge County, GA | 0.987 | 0.89 | Low RR + High P | No | 0.564 | 39649 |
| 46055 | Haakon County, SD | 1.546 | 0.58 | High RR + Low P | No | 0.564 | 3685 |
| 13231 | Pike County, GA | 1.172 | 0.72 | Low RR + Low P | No | 0.564 | 40416 |
| 38023 | Divide County, ND | 1.307 | 0.7 | Low RR + Low P | No | 0.564 | 4304 |
| 53023 | Garfield County, WA | 1.464 | 0.6 | High RR + Low P | No | 0.564 | 4720 |
| 48253 | Jones County, TX | 1.303 | 0.62 | Low RR + Low P | No | 0.564 | 40652 |
| 49035 | Salt Lake County, UT | 0.851 | 0.9 | Low RR + High P | No | 0.564 | 2372382 |
| 46007 | Bennett County, SD | 1.727 | 0.52 | High RR + Low P | No | 0.564 | 6638 |
| 38087 | Slope County, ND | 1.469 | 0.6 | High RR + Low P | No | 0.564 | 1356 |
| 19123 | Mahaska County, IA | 1.132 | 0.76 | Low RR + Low P | No | 0.563 | 43845 |
| 48249 | Jim Wells County, TX | 1.356 | 0.57 | Low RR + Low P | No | 0.563 | 77391 |
| 51810 | Virginia Beach city, VA | 0.772 | 0.96 | Low RR + High P | No | 0.563 | 908718 |
| 2060 | Bristol Bay Borough, AK | 1.668 | 0.55 | High RR + Low P | No | 0.563 | 1714 |
| 5045 | Faulkner County, AR | 1.164 | 0.65 | Low RR + Low P | No | 0.563 | 257516 |
| 47117 | Marshall County, TN | 1.396 | 0.55 | Low RR + Low P | No | 0.563 | 72818 |
| 48145 | Falls County, TX | 1.752 | 0.44 | High RR + Low P | No | 0.562 | 34242 |
| 18137 | Ripley County, IN | 1.382 | 0.56 | Low RR + Low P | No | 0.562 | 58257 |
| 27087 | Mahnomen County, MN | 2.468 | 0.42 | High RR + Low P | No | 0.562 | 10619 |
| 31077 | Greeley County, NE | 1.465 | 0.6 | High RR + Low P | No | 0.562 | 4454 |
| 48051 | Burleson County, TX | 1.159 | 0.74 | Low RR + Low P | No | 0.562 | 38165 |
| 17001 | Adams County, IL | 1.08 | 0.74 | Low RR + Low P | No | 0.561 | 128997 |
| 13057 | Cherokee County, GA | 0.664 | 1 | Low RR + High P | No | 0.561 | 567796 |
| 28019 | Choctaw County, MS | 1.134 | 0.8 | Low RR + Low P | No | 0.561 | 16183 |
| 18121 | Parke County, IN | 1.18 | 0.72 | Low RR + Low P | No | 0.561 | 32851 |
| 19161 | Sac County, IA | 1.582 | 0.53 | High RR + Low P | No | 0.56 | 19375 |
| 41065 | Wasco County, OR | 1.614 | 0.46 | High RR + Low P | No | 0.56 | 52888 |
| 19073 | Greene County, IA | 1.294 | 0.66 | Low RR + Low P | No | 0.56 | 17294 |
| 46065 | Hughes County, SD | 1.142 | 0.75 | Low RR + Low P | No | 0.559 | 35305 |
| 40033 | Cotton County, OK | 2.19 | 0.42 | High RR + Low P | No | 0.559 | 10876 |
| 13153 | Houston County, GA | 0.857 | 0.91 | Low RR + High P | No | 0.559 | 341749 |
| 21163 | Meade County, KY | 1.529 | 0.49 | High RR + Low P | No | 0.559 | 60141 |
| 53069 | Wahkiakum County, WA | 1.336 | 0.65 | Low RR + Low P | No | 0.559 | 9453 |
| 48487 | Wilbarger County, TX | 1.448 | 0.56 | High RR + Low P | No | 0.559 | 25057 |
| 31021 | Burt County, NE | 1.231 | 0.72 | Low RR + Low P | No | 0.558 | 13497 |
| 13205 | Mitchell County, GA | 1.099 | 0.77 | Low RR + Low P | No | 0.558 | 42306 |
| 20065 | Graham County, KS | 1.337 | 0.66 | Low RR + Low P | No | 0.557 | 4773 |
| 12041 | Gilchrist County, FL | 1.138 | 0.75 | Low RR + Low P | No | 0.557 | 38589 |
| 54035 | Jackson County, WV | 1.392 | 0.56 | Low RR + Low P | No | 0.557 | 55260 |
| 18059 | Hancock County, IN | 0.92 | 0.86 | Low RR + High P | No | 0.557 | 169283 |
| 17161 | Rock Island County, IL | 0.857 | 0.91 | Low RR + High P | No | 0.557 | 282795 |
| 46071 | Jackson County, SD | 1.562 | 0.56 | High RR + Low P | No | 0.557 | 5608 |
| 51069 | Frederick County, VA | 0.929 | 0.85 | Low RR + High P | No | 0.557 | 190802 |
| 20195 | Trego County, KS | 1.577 | 0.56 | High RR + Low P | No | 0.556 | 5477 |
| 12086 | Miami-Dade County, FL | 0.701 | 0.97 | Low RR + High P | No | 0.556 | 5359923 |
| 13135 | Gwinnett County, GA | 0.56 | 1 | Low RR + High P | No | 0.556 | 1959699 |
| 37065 | Edgecombe County, NC | 2.1 | 0.21 | High RR + Low P | No | 0.555 | 97221 |
| 54055 | Mercer County, WV | 1.244 | 0.6 | Low RR + Low P | No | 0.555 | 116669 |
| 25023 | Plymouth County, MA | 0.717 | 0.97 | Low RR + High P | No | 0.555 | 1068556 |
| 41057 | Tillamook County, OR | 0.938 | 0.9 | Low RR + High P | No | 0.555 | 54938 |
| 19007 | Appanoose County, IA | 1.828 | 0.42 | High RR + Low P | No | 0.554 | 24260 |
| 12099 | Palm Beach County, FL | 0.54 | 1 | Low RR + High P | No | 0.554 | 3053668 |
| 51143 | Pittsylvania County, VA | 0.919 | 0.87 | Low RR + High P | No | 0.553 | 119395 |
| 20117 | Marshall County, KS | 0.982 | 0.9 | Low RR + High P | No | 0.553 | 19925 |
| 39087 | Lawrence County, OH | 1.585 | 0.41 | High RR + Low P | No | 0.552 | 112690 |
| 55119 | Taylor County, WI | 1.187 | 0.69 | Low RR + Low P | No | 0.552 | 39999 |
| 8045 | Garfield County, CO | 0.921 | 0.86 | Low RR + High P | No | 0.551 | 124963 |
| 6069 | San Benito County, CA | 1.005 | 0.77 | Low RR + Low P | No | 0.551 | 135818 |
| 42091 | Montgomery County, PA | 0.662 | 0.98 | Low RR + High P | No | 0.551 | 1733786 |
| 17095 | Knox County, IL | 1.523 | 0.45 | High RR + Low P | No | 0.551 | 97031 |
| 38083 | Sheridan County, ND | 1.535 | 0.56 | High RR + Low P | No | 0.551 | 2552 |
| 38005 | Benson County, ND | 2.702 | 0.36 | High RR + Low P | No | 0.551 | 11517 |
| 48243 | Jeff Davis County, TX | 1.22 | 0.73 | Low RR + Low P | No | 0.551 | 3762 |
| 38025 | Dunn County, ND | 1.352 | 0.63 | Low RR + Low P | No | 0.551 | 8013 |
| 42015 | Bradford County, PA | 1.152 | 0.66 | Low RR + Low P | No | 0.55 | 119549 |
| 13053 | Chattahoochee County, GA | 1.427 | 0.57 | Low RR + Low P | No | 0.55 | 17407 |
| 51197 | Wythe County, VA | 1.46 | 0.51 | High RR + Low P | No | 0.55 | 56219 |
| 5099 | Nevada County, AR | 1.14 | 0.77 | Low RR + Low P | No | 0.55 | 16299 |
| 35043 | Sandoval County, NM | 0.751 | 0.96 | Low RR + High P | No | 0.55 | 309533 |
| 31129 | Nuckolls County, NE | 1.604 | 0.53 | High RR + Low P | No | 0.549 | 8164 |
| 38043 | Kidder County, ND | 1.497 | 0.57 | High RR + Low P | No | 0.549 | 4723 |
| 53009 | Clallam County, WA | 1.032 | 0.75 | Low RR + Low P | No | 0.548 | 155300 |
| 26135 | Oscoda County, MI | 1.304 | 0.64 | Low RR + Low P | No | 0.548 | 16936 |
| 27009 | Benton County, MN | 1.448 | 0.49 | High RR + Low P | No | 0.548 | 83109 |
| 13113 | Fayette County, GA | 0.666 | 0.99 | Low RR + High P | No | 0.548 | 245332 |
| 13111 | Fannin County, GA | 0.884 | 0.94 | Low RR + High P | No | 0.548 | 51688 |
| 16021 | Boundary County, ID | 1.026 | 0.84 | Low RR + High P | No | 0.548 | 26829 |
| 22125 | West Feliciana Parish, LA | 0.978 | 0.88 | Low RR + High P | No | 0.548 | 30767 |
| 54065 | Morgan County, WV | 1.125 | 0.75 | Low RR + Low P | No | 0.548 | 35084 |
| 39007 | Ashtabula County, OH | 1.393 | 0.49 | Low RR + Low P | No | 0.547 | 193805 |
| 13287 | Turner County, GA | 1.892 | 0.41 | High RR + Low P | No | 0.547 | 17745 |
| 30005 | Blaine County, MT | 1.525 | 0.54 | High RR + Low P | No | 0.547 | 13871 |
| 53067 | Thurston County, WA | 1.113 | 0.64 | Low RR + Low P | No | 0.547 | 597642 |
| 33009 | Grafton County, NH | 0.958 | 0.8 | Low RR + High P | No | 0.547 | 186277 |
| 21235 | Whitley County, KY | 1.651 | 0.38 | High RR + Low P | No | 0.547 | 73733 |
| 18173 | Warrick County, IN | 0.94 | 0.83 | Low RR + High P | No | 0.546 | 131098 |
| 12115 | Sarasota County, FL | 0.518 | 1 | Low RR + High P | No | 0.546 | 931565 |
| 27129 | Renville County, MN | 1.372 | 0.57 | Low RR + Low P | No | 0.546 | 28821 |
| 21043 | Carter County, KY | 2.056 | 0.26 | High RR + Low P | No | 0.546 | 52709 |
| 38059 | Morton County, ND | 1.046 | 0.76 | Low RR + Low P | No | 0.546 | 67600 |
| 16033 | Clark County, ID | 2.964 | 0.37 | High RR + Low P | No | 0.546 | 1611 |
| 30079 | Prairie County, MT | 1.897 | 0.45 | High RR + Low P | No | 0.546 | 2220 |
| 31083 | Harlan County, NE | 1.069 | 0.84 | Low RR + High P | No | 0.546 | 6077 |
| 53029 | Island County, WA | 1.039 | 0.73 | Low RR + Low P | No | 0.545 | 172949 |
| 37175 | Transylvania County, NC | 0.795 | 0.98 | Low RR + High P | No | 0.545 | 66922 |
| 1069 | Houston County, AL | 0.959 | 0.79 | Low RR + Low P | No | 0.545 | 216509 |
| 16045 | Gem County, ID | 1.115 | 0.73 | Low RR + Low P | No | 0.544 | 41525 |
| 13311 | White County, GA | 0.867 | 0.93 | Low RR + High P | No | 0.544 | 57830 |
| 48301 | Loving County, TX | 1.25 | 0.71 | Low RR + Low P | No | 0.544 | 87 |
| 20097 | Kiowa County, KS | 1.584 | 0.53 | High RR + Low P | No | 0.544 | 4779 |
| 47075 | Haywood County, TN | 1.921 | 0.33 | High RR + Low P | No | 0.544 | 34846 |
| 48361 | Orange County, TX | 1.429 | 0.46 | Low RR + Low P | No | 0.544 | 170770 |
| 50003 | Bennington County, VT | 1.577 | 0.42 | High RR + Low P | No | 0.544 | 74526 |
| 37193 | Wilkes County, NC | 1.157 | 0.64 | Low RR + Low P | No | 0.544 | 131804 |
| 22005 | Ascension Parish, LA | 1.135 | 0.64 | Low RR + Low P | No | 0.543 | 262117 |
| 28053 | Humphreys County, MS | 1.898 | 0.41 | High RR + Low P | No | 0.542 | 14570 |
| 41063 | Wallowa County, OR | 1.336 | 0.61 | Low RR + Low P | No | 0.542 | 15324 |
| 30107 | Wheatland County, MT | 1.804 | 0.46 | High RR + Low P | No | 0.542 | 4095 |
| 31173 | Thurston County, NE | 1.436 | 0.57 | High RR + Low P | No | 0.542 | 13112 |
| 21027 | Breckinridge County, KY | 1.288 | 0.6 | Low RR + Low P | No | 0.542 | 42085 |
| 48417 | Shackelford County, TX | 1.417 | 0.59 | Low RR + Low P | No | 0.542 | 6441 |
| 28015 | Carroll County, MS | 1.679 | 0.46 | High RR + Low P | No | 0.541 | 19271 |
| 13099 | Early County, GA | 0.952 | 0.91 | Low RR + High P | No | 0.541 | 21123 |
| 48105 | Crockett County, TX | 1.735 | 0.48 | High RR + Low P | No | 0.541 | 5818 |
| 48313 | Madison County, TX | 1.8 | 0.39 | High RR + Low P | No | 0.541 | 27383 |
| 18063 | Hendricks County, IN | 0.873 | 0.85 | Low RR + High P | No | 0.541 | 369506 |
| 21217 | Taylor County, KY | 1.528 | 0.46 | High RR + Low P | No | 0.541 | 52839 |
| 13271 | Telfair County, GA | 0.903 | 0.95 | Low RR + High P | No | 0.541 | 23298 |
| 18163 | Vanderburgh County, IN | 1.221 | 0.56 | Low RR + Low P | No | 0.54 | 359373 |
| 1053 | Escambia County, AL | 1.87 | 0.25 | High RR + Low P | No | 0.54 | 73194 |
| 37111 | McDowell County, NC | 1.195 | 0.61 | Low RR + Low P | No | 0.54 | 89648 |
| 13163 | Jefferson County, GA | 2.002 | 0.31 | High RR + Low P | No | 0.54 | 30477 |
| 40077 | Latimer County, OK | 1.899 | 0.38 | High RR + Low P | No | 0.54 | 19082 |
| 38015 | Burleigh County, ND | 0.821 | 0.91 | Low RR + High P | No | 0.54 | 199408 |
| 20013 | Brown County, KS | 1.165 | 0.72 | Low RR + Low P | No | 0.54 | 18623 |
| 27139 | Scott County, MN | 1.126 | 0.63 | Low RR + Low P | No | 0.539 | 310237 |
| 47049 | Fentress County, TN | 0.942 | 0.89 | Low RR + High P | No | 0.539 | 39031 |
| 39163 | Vinton County, OH | 1.549 | 0.5 | High RR + Low P | No | 0.539 | 25051 |
| 27089 | Marshall County, MN | 1.243 | 0.66 | Low RR + Low P | No | 0.538 | 17699 |
| 51640 | Galax city, VA | 1.048 | 0.83 | Low RR + High P | No | 0.538 | 13419 |
| 51059 | Fairfax County, VA | 0.378 | 1 | Low RR + High P | No | 0.538 | 2281187 |
| 20063 | Gove County, KS | 1.341 | 0.62 | Low RR + Low P | No | 0.538 | 5477 |
| 2282 | Yakutat Borough, AK | 2.812 | 0.35 | High RR + Low P | No | 0.537 | 1372 |
| 21091 | Hancock County, KY | 1.108 | 0.77 | Low RR + Low P | No | 0.537 | 17947 |
| 39115 | Morgan County, OH | 1.321 | 0.59 | Low RR + Low P | No | 0.537 | 27315 |
| 20175 | Seward County, KS | 0.992 | 0.82 | Low RR + High P | No | 0.536 | 42412 |
| 31009 | Blaine County, NE | 1.306 | 0.65 | Low RR + Low P | No | 0.536 | 888 |
| 20159 | Rice County, KS | 1.077 | 0.79 | Low RR + Low P | No | 0.536 | 18630 |
| 21075 | Fulton County, KY | 1.402 | 0.57 | Low RR + Low P | No | 0.536 | 12709 |
| 26117 | Montcalm County, MI | 1.088 | 0.68 | Low RR + Low P | No | 0.536 | 135363 |
| 21087 | Green County, KY | 1.372 | 0.57 | Low RR + Low P | No | 0.535 | 22860 |
| 31081 | Hamilton County, NE | 0.989 | 0.86 | Low RR + High P | No | 0.535 | 18976 |
| 42097 | Northumberland County, PA | 1.347 | 0.5 | Low RR + Low P | No | 0.535 | 180243 |
| 48299 | Llano County, TX | 1.106 | 0.72 | Low RR + Low P | No | 0.535 | 45498 |
| 46105 | Perkins County, SD | 1.321 | 0.63 | Low RR + Low P | No | 0.535 | 5643 |
| 46079 | Lake County, SD | 1.261 | 0.63 | Low RR + Low P | No | 0.535 | 21971 |
| 16057 | Latah County, ID | 0.942 | 0.82 | Low RR + High P | No | 0.534 | 82225 |
| 46129 | Walworth County, SD | 1.423 | 0.56 | Low RR + Low P | No | 0.534 | 10555 |
| 13177 | Lee County, GA | 0.826 | 0.94 | Low RR + High P | No | 0.534 | 67550 |
| 13273 | Terrell County, GA | 1.001 | 0.85 | Low RR + High P | No | 0.534 | 17510 |
| 5083 | Logan County, AR | 1.116 | 0.71 | Low RR + Low P | No | 0.534 | 42658 |
| 36007 | Broome County, NY | 1.097 | 0.64 | Low RR + Low P | No | 0.534 | 393335 |
| 37121 | Mitchell County, NC | 1.197 | 0.66 | Low RR + Low P | No | 0.533 | 30047 |
| 54061 | Monongalia County, WV | 0.958 | 0.77 | Low RR + Low P | No | 0.533 | 214696 |
| 19061 | Dubuque County, IA | 1.333 | 0.5 | Low RR + Low P | No | 0.533 | 197602 |
| 44001 | Bristol County, RI | 0.949 | 0.8 | Low RR + High P | No | 0.533 | 100562 |
| 39133 | Portage County, OH | 0.935 | 0.78 | Low RR + Low P | No | 0.532 | 324407 |
| 48485 | Wichita County, TX | 1.832 | 0.1 | High RR + Low P | No | 0.532 | 260400 |
| 29139 | Montgomery County, MO | 1.326 | 0.59 | Low RR + Low P | No | 0.532 | 22938 |
| 13213 | Murray County, GA | 1.084 | 0.7 | Low RR + Low P | No | 0.532 | 81445 |
| 5037 | Cross County, AR | 1.004 | 0.81 | Low RR + High P | No | 0.532 | 33010 |
| 18123 | Perry County, IN | 0.932 | 0.88 | Low RR + High P | No | 0.532 | 38416 |
| 12119 | Sumter County, FL | 0.532 | 1 | Low RR + High P | No | 0.532 | 296332 |
| 19081 | Hancock County, IA | 1.201 | 0.67 | Low RR + Low P | No | 0.531 | 21266 |
| 48191 | Hall County, TX | 1.363 | 0.6 | Low RR + Low P | No | 0.531 | 5636 |
| 1107 | Pickens County, AL | 0.868 | 0.93 | Low RR + High P | No | 0.531 | 37437 |
| 55019 | Clark County, WI | 0.901 | 0.87 | Low RR + High P | No | 0.531 | 69506 |
| 27141 | Sherburne County, MN | 1.045 | 0.69 | Low RR + Low P | No | 0.531 | 202746 |
| 21161 | Mason County, KY | 2.028 | 0.26 | High RR + Low P | No | 0.531 | 33744 |
| 30041 | Hill County, MT | 1.361 | 0.56 | Low RR + Low P | No | 0.53 | 32394 |
| 17117 | Macoupin County, IL | 1.323 | 0.53 | Low RR + Low P | No | 0.53 | 88141 |
| 49003 | Box Elder County, UT | 1.054 | 0.7 | Low RR + Low P | No | 0.53 | 124207 |
| 12011 | Broward County, FL | 0.523 | 0.98 | Low RR + High P | No | 0.53 | 3909420 |
| 55107 | Rusk County, WI | 0.938 | 0.89 | Low RR + High P | No | 0.53 | 28242 |
| 27115 | Pine County, MN | 1.447 | 0.47 | High RR + Low P | No | 0.53 | 59730 |
| 20201 | Washington County, KS | 1.547 | 0.51 | High RR + Low P | No | 0.53 | 10997 |
| 16035 | Clearwater County, ID | 0.999 | 0.84 | Low RR + High P | No | 0.53 | 18249 |
| 13143 | Haralson County, GA | 1.207 | 0.6 | Low RR + Low P | No | 0.529 | 63393 |
| 54059 | Mingo County, WV | 2.27 | 0.16 | High RR + Low P | No | 0.529 | 44468 |
| 32019 | Lyon County, NV | 1.107 | 0.65 | Low RR + Low P | No | 0.529 | 124211 |
| 49013 | Duchesne County, UT | 1.125 | 0.7 | Low RR + Low P | No | 0.529 | 40619 |
| 47041 | DeKalb County, TN | 1.186 | 0.64 | Low RR + Low P | No | 0.529 | 42227 |
| 37011 | Avery County, NC | 1.057 | 0.76 | Low RR + Low P | No | 0.528 | 35102 |
| 36049 | Lewis County, NY | 1.178 | 0.63 | Low RR + Low P | No | 0.528 | 53143 |
| 29221 | Washington County, MO | 0.986 | 0.8 | Low RR + High P | No | 0.528 | 46926 |
| 29227 | Worth County, MO | 1.274 | 0.65 | Low RR + Low P | No | 0.528 | 3847 |
| 31133 | Pawnee County, NE | 1.283 | 0.64 | Low RR + Low P | No | 0.528 | 5043 |
| 53017 | Douglas County, WA | 1.145 | 0.63 | Low RR + Low P | No | 0.527 | 89021 |
| 19197 | Wright County, IA | 1.365 | 0.56 | Low RR + Low P | No | 0.526 | 25351 |
| 20019 | Chautauqua County, KS | 1.185 | 0.71 | Low RR + Low P | No | 0.526 | 6734 |
| 48081 | Coke County, TX | 1.229 | 0.67 | Low RR + Low P | No | 0.526 | 6674 |
| 47015 | Cannon County, TN | 1.738 | 0.36 | High RR + Low P | No | 0.526 | 29870 |
| 23005 | Cumberland County, ME | 0.564 | 0.98 | Low RR + High P | No | 0.526 | 619067 |
| 37027 | Caldwell County, NC | 1.329 | 0.5 | Low RR + Low P | No | 0.526 | 160921 |
| 31163 | Sherman County, NE | 1.234 | 0.67 | Low RR + Low P | No | 0.526 | 5989 |
| 13043 | Candler County, GA | 1.096 | 0.75 | Low RR + Low P | No | 0.526 | 22021 |
| 41031 | Jefferson County, OR | 1.272 | 0.57 | Low RR + Low P | No | 0.526 | 50778 |
| 8089 | Otero County, CO | 1.994 | 0.24 | High RR + Low P | No | 0.526 | 36447 |
| 1043 | Cullman County, AL | 0.638 | 0.98 | Low RR + High P | No | 0.525 | 182679 |
| 18119 | Owen County, IN | 1.096 | 0.71 | Low RR + Low P | No | 0.525 | 43019 |
| 50021 | Rutland County, VT | 1.789 | 0.16 | High RR + Low P | No | 0.525 | 120624 |
| 41027 | Hood River County, OR | 1.17 | 0.64 | Low RR + Low P | No | 0.525 | 47780 |
| 16053 | Jerome County, ID | 1.172 | 0.63 | Low RR + Low P | No | 0.525 | 50723 |
| 54005 | Boone County, WV | 1.734 | 0.31 | High RR + Low P | No | 0.525 | 41483 |
| 27117 | Pipestone County, MN | 1.055 | 0.78 | Low RR + Low P | No | 0.525 | 18572 |
| 21173 | Montgomery County, KY | 1.64 | 0.33 | High RR + Low P | No | 0.524 | 56884 |
| 30077 | Powell County, MT | 1.14 | 0.73 | Low RR + Low P | No | 0.524 | 14211 |
| 5041 | Desha County, AR | 1.215 | 0.64 | Low RR + Low P | No | 0.524 | 21237 |
| 47081 | Hickman County, TN | 0.887 | 0.89 | Low RR + High P | No | 0.524 | 51307 |
| 1095 | Marshall County, AL | 0.661 | 0.97 | Low RR + High P | No | 0.524 | 200184 |
| 45029 | Colleton County, SC | 1.772 | 0.2 | High RR + Low P | No | 0.524 | 77454 |
| 12071 | Lee County, FL | 0.453 | 0.99 | Low RR + High P | No | 0.523 | 1656964 |
| 29019 | Boone County, MO | 0.863 | 0.82 | Low RR + High P | No | 0.523 | 377184 |
| 19041 | Clay County, IA | 1.134 | 0.7 | Low RR + Low P | No | 0.523 | 33018 |
| 35059 | Union County, NM | 1.003 | 0.85 | Low RR + High P | No | 0.523 | 7946 |
| 37023 | Burke County, NC | 1.408 | 0.42 | Low RR + Low P | No | 0.523 | 176310 |
| 51111 | Lunenburg County, VA | 0.919 | 0.9 | Low RR + High P | No | 0.523 | 24040 |
| 21213 | Simpson County, KY | 1.657 | 0.35 | High RR + Low P | No | 0.522 | 40143 |
| 6033 | Lake County, CA | 1.537 | 0.32 | High RR + Low P | No | 0.522 | 136050 |
| 39001 | Adams County, OH | 1.783 | 0.25 | High RR + Low P | No | 0.522 | 54980 |
| 12067 | Lafayette County, FL | 1.209 | 0.65 | Low RR + Low P | No | 0.522 | 15855 |
| 22007 | Assumption Parish, LA | 1.758 | 0.29 | High RR + Low P | No | 0.522 | 40623 |
| 51183 | Sussex County, VA | 1.039 | 0.78 | Low RR + Low P | No | 0.522 | 21397 |
| 54041 | Lewis County, WV | 1.73 | 0.34 | High RR + Low P | No | 0.521 | 33174 |
| 21197 | Powell County, KY | 2.007 | 0.25 | High RR + Low P | No | 0.521 | 26013 |
| 30103 | Treasure County, MT | 1.621 | 0.48 | High RR + Low P | No | 0.52 | 1526 |
| 13243 | Randolph County, GA | 1.14 | 0.73 | Low RR + Low P | No | 0.52 | 12204 |
| 55087 | Outagamie County, WI | 1.15 | 0.58 | Low RR + Low P | No | 0.52 | 385503 |
| 42127 | Wayne County, PA | 0.948 | 0.78 | Low RR + Low P | No | 0.52 | 102346 |
| 28073 | Lamar County, MS | 0.902 | 0.81 | Low RR + High P | No | 0.52 | 131963 |
| 54093 | Tucker County, WV | 1.536 | 0.49 | High RR + Low P | No | 0.52 | 13224 |
| 26093 | Livingston County, MI | 0.818 | 0.85 | Low RR + High P | No | 0.52 | 392891 |
| 54001 | Barbour County, WV | 1.638 | 0.38 | High RR + Low P | No | 0.52 | 30769 |
| 29011 | Barton County, MO | 0.932 | 0.88 | Low RR + High P | No | 0.52 | 23426 |
| 1057 | Fayette County, AL | 1.066 | 0.74 | Low RR + Low P | No | 0.519 | 32049 |
| 8033 | Dolores County, CO | 1.61 | 0.48 | High RR + Low P | No | 0.519 | 4973 |
| 46083 | Lincoln County, SD | 0.889 | 0.82 | Low RR + High P | No | 0.519 | 144262 |
| 53039 | Klickitat County, WA | 1.029 | 0.75 | Low RR + Low P | No | 0.519 | 46892 |
| 8035 | Douglas County, CO | 0.673 | 0.93 | Low RR + High P | No | 0.518 | 759843 |
| 21023 | Bracken County, KY | 1.21 | 0.64 | Low RR + Low P | No | 0.518 | 16861 |
| 25013 | Hampden County, MA | 0.859 | 0.8 | Low RR + High P | No | 0.517 | 921049 |
| 37043 | Clay County, NC | 0.891 | 0.92 | Low RR + High P | No | 0.517 | 23479 |
| 48037 | Bowie County, TX | 1.745 | 0.1 | High RR + Low P | No | 0.517 | 183399 |
| 51177 | Spotsylvania County, VA | 0.516 | 0.98 | Low RR + High P | No | 0.516 | 296416 |
| 48147 | Fannin County, TX | 1.027 | 0.72 | Low RR + Low P | No | 0.516 | 74646 |
| 21103 | Henry County, KY | 1.145 | 0.67 | Low RR + Low P | No | 0.516 | 31733 |
| 22041 | Franklin Parish, LA | 2.746 | 0.07 | High RR + Low P | No | 0.516 | 38643 |
| 45069 | Marlboro County, SC | 0.939 | 0.82 | Low RR + High P | No | 0.516 | 51710 |
| 47181 | Wayne County, TN | 1.182 | 0.63 | Low RR + Low P | No | 0.515 | 32332 |
| 50013 | Grand Isle County, VT | 1.168 | 0.69 | Low RR + Low P | No | 0.515 | 14957 |
| 17157 | Randolph County, IL | 0.964 | 0.78 | Low RR + Low P | No | 0.515 | 59841 |
| 38017 | Cass County, ND | 0.997 | 0.69 | Low RR + Low P | No | 0.515 | 389075 |
| 56029 | Park County, WY | 0.826 | 0.91 | Low RR + High P | No | 0.515 | 61240 |
| 51107 | Loudoun County, VA | 0.394 | 0.98 | Low RR + High P | No | 0.515 | 869139 |
| 41047 | Marion County, OR | 1.269 | 0.49 | Low RR + Low P | No | 0.515 | 693460 |
| 27073 | Lac qui Parle County, MN | 1.152 | 0.7 | Low RR + Low P | No | 0.514 | 13286 |
| 42117 | Tioga County, PA | 0.871 | 0.85 | Low RR + High P | No | 0.514 | 81807 |
| 13239 | Quitman County, GA | 1.391 | 0.56 | Low RR + Low P | No | 0.514 | 4539 |
| 28135 | Tallahatchie County, MS | 1.041 | 0.76 | Low RR + Low P | No | 0.514 | 23904 |
| 38093 | Stutsman County, ND | 1.24 | 0.58 | Low RR + Low P | No | 0.514 | 42868 |
| 48287 | Lee County, TX | 1.381 | 0.51 | Low RR + Low P | No | 0.514 | 36211 |
| 51179 | Stafford County, VA | 0.529 | 0.98 | Low RR + High P | No | 0.513 | 328597 |
| 24047 | Worcester County, MD | 0.892 | 0.82 | Low RR + High P | No | 0.513 | 108105 |
| 23029 | Washington County, ME | 1.09 | 0.67 | Low RR + Low P | No | 0.513 | 63082 |
| 40129 | Roger Mills County, OK | 1.318 | 0.59 | Low RR + Low P | No | 0.513 | 6632 |
| 12051 | Hendry County, FL | 0.95 | 0.77 | Low RR + Low P | No | 0.513 | 84724 |
| 20189 | Stevens County, KS | 1.098 | 0.75 | Low RR + Low P | No | 0.513 | 10243 |
| 29189 | St. Louis County, MO | 0.803 | 0.83 | Low RR + High P | No | 0.513 | 1977850 |
| 27159 | Wadena County, MN | 1.629 | 0.37 | High RR + Low P | No | 0.512 | 28477 |
| 13123 | Gilmer County, GA | 0.773 | 0.93 | Low RR + High P | No | 0.512 | 65263 |
| 48403 | Sabine County, TX | 0.994 | 0.81 | Low RR + High P | No | 0.512 | 20147 |
| 46027 | Clay County, SD | 1.457 | 0.47 | High RR + Low P | No | 0.512 | 30732 |
| 53001 | Adams County, WA | 1.598 | 0.35 | High RR + Low P | No | 0.512 | 41718 |
| 17077 | Jackson County, IL | 0.957 | 0.76 | Low RR + Low P | No | 0.511 | 104618 |
| 37091 | Hertford County, NC | 1.755 | 0.26 | High RR + Low P | No | 0.511 | 39087 |
| 12019 | Clay County, FL | 0.819 | 0.83 | Low RR + High P | No | 0.511 | 459073 |
| 27135 | Roseau County, MN | 1.604 | 0.37 | High RR + Low P | No | 0.511 | 30586 |
| 29093 | Iron County, MO | 1.13 | 0.7 | Low RR + Low P | No | 0.511 | 18853 |
| 37153 | Richmond County, NC | 1.448 | 0.39 | High RR + Low P | No | 0.511 | 84897 |
| 21143 | Lyon County, KY | 1.153 | 0.68 | Low RR + Low P | No | 0.51 | 18286 |
| 2164 | Lake and Peninsula Borough, AK | 1.734 | 0.42 | High RR + Low P | No | 0.51 | 2708 |
| 48391 | Refugio County, TX | 1.058 | 0.77 | Low RR + Low P | No | 0.51 | 13304 |
| 54017 | Doddridge County, WV | 1.099 | 0.73 | Low RR + Low P | No | 0.51 | 15392 |
| 21041 | Carroll County, KY | 1.652 | 0.37 | High RR + Low P | No | 0.51 | 21926 |
| 51121 | Montgomery County, VA | 0.802 | 0.86 | Low RR + High P | No | 0.51 | 197492 |
| 55041 | Forest County, WI | 1.555 | 0.44 | High RR + Low P | No | 0.509 | 18657 |
| 26079 | Kalkaska County, MI | 1.354 | 0.52 | Low RR + Low P | No | 0.509 | 36734 |
| 48009 | Archer County, TX | 1.377 | 0.54 | Low RR + Low P | No | 0.509 | 17878 |
| 12027 | DeSoto County, FL | 0.874 | 0.84 | Low RR + High P | No | 0.509 | 71299 |
| 42025 | Carbon County, PA | 1.105 | 0.61 | Low RR + Low P | No | 0.509 | 130951 |
| 49037 | San Juan County, UT | 1.031 | 0.76 | Low RR + Low P | No | 0.509 | 28764 |
| 49053 | Washington County, UT | 0.706 | 0.9 | Low RR + High P | No | 0.509 | 400182 |
| 5005 | Baxter County, AR | 0.642 | 0.97 | Low RR + High P | No | 0.509 | 85360 |
| 48349 | Navarro County, TX | 1.373 | 0.43 | Low RR + Low P | No | 0.508 | 110322 |
| 29013 | Bates County, MO | 0.907 | 0.86 | Low RR + High P | No | 0.508 | 32412 |
| 29009 | Barry County, MO | 0.843 | 0.88 | Low RR + High P | No | 0.507 | 70209 |
| 47097 | Lauderdale County, TN | 1.276 | 0.55 | Low RR + Low P | No | 0.507 | 49351 |
| 38071 | Ramsey County, ND | 2.167 | 0.16 | High RR + Low P | No | 0.506 | 22979 |
| 55037 | Florence County, WI | 1.472 | 0.5 | High RR + Low P | No | 0.506 | 9388 |
| 46053 | Gregory County, SD | 1.194 | 0.66 | Low RR + Low P | No | 0.506 | 8017 |
| 47125 | Montgomery County, TN | 1.567 | 0.15 | High RR + Low P | No | 0.506 | 474771 |
| 40007 | Beaver County, OK | 1.216 | 0.63 | Low RR + Low P | No | 0.506 | 10037 |
| 22001 | Acadia Parish, LA | 0.892 | 0.8 | Low RR + High P | No | 0.506 | 113214 |
| 29211 | Sullivan County, MO | 1.648 | 0.4 | High RR + Low P | No | 0.505 | 11613 |
| 39047 | Fayette County, OH | 1.472 | 0.39 | High RR + Low P | No | 0.505 | 57649 |
| 37105 | Lee County, NC | 1.269 | 0.5 | Low RR + Low P | No | 0.505 | 132602 |
| 19099 | Jasper County, IA | 0.801 | 0.9 | Low RR + High P | No | 0.505 | 75868 |
| 13007 | Baker County, GA | 1.306 | 0.59 | Low RR + Low P | No | 0.504 | 5535 |
| 21089 | Greenup County, KY | 1.827 | 0.08 | High RR + Low P | No | 0.504 | 70596 |
| 38039 | Griggs County, ND | 1.353 | 0.57 | Low RR + Low P | No | 0.504 | 4497 |
| 54079 | Putnam County, WV | 1.068 | 0.64 | Low RR + Low P | No | 0.503 | 114025 |
| 12113 | Santa Rosa County, FL | 0.86 | 0.79 | Low RR + Low P | No | 0.503 | 401361 |
| 13009 | Baldwin County, GA | 1.414 | 0.4 | Low RR + Low P | No | 0.503 | 87029 |
| 45025 | Chesterfield County, SC | 1.108 | 0.62 | Low RR + Low P | No | 0.503 | 87772 |
| 17041 | Douglas County, IL | 0.79 | 0.94 | Low RR + High P | No | 0.503 | 39296 |
| 48163 | Frio County, TX | 1.9 | 0.15 | High RR + Low P | No | 0.502 | 35703 |
| 38065 | Oliver County, ND | 1.265 | 0.61 | Low RR + Low P | No | 0.502 | 3741 |
| 22061 | Lincoln Parish, LA | 0.708 | 0.93 | Low RR + High P | No | 0.502 | 95942 |
| 27085 | McLeod County, MN | 1.598 | 0.25 | High RR + Low P | No | 0.502 | 73519 |
| 31185 | York County, NE | 1.386 | 0.5 | Low RR + Low P | No | 0.502 | 28668 |
| 39005 | Ashland County, OH | 1.555 | 0.25 | High RR + Low P | No | 0.502 | 104214 |
| 18093 | Lawrence County, IN | 1.517 | 0.3 | High RR + Low P | No | 0.502 | 90315 |
| 13127 | Glynn County, GA | 0.599 | 0.95 | Low RR + High P | No | 0.502 | 171317 |
| 27151 | Swift County, MN | 1.564 | 0.41 | High RR + Low P | No | 0.502 | 19490 |
| 21133 | Letcher County, KY | 1.542 | 0.36 | High RR + Low P | No | 0.502 | 41250 |
| 46069 | Hyde County, SD | 1.732 | 0.39 | High RR + Low P | No | 0.502 | 2385 |
| 6099 | Stanislaus County, CA | 1.724 | 0 | High RR + Low P | No | 0.501 | 1102656 |
| 48157 | Fort Bend County, TX | 0.866 | 0.76 | Low RR + Low P | No | 0.501 | 1805697 |
| 51620 | Franklin city, VA | 1.652 | 0.36 | High RR + Low P | No | 0.501 | 16613 |
| 20099 | Labette County, KS | 0.861 | 0.88 | Low RR + High P | No | 0.501 | 39430 |
| 49055 | Wayne County, UT | 1.45 | 0.51 | High RR + Low P | No | 0.5 | 5248 |
| 46089 | McPherson County, SD | 1.488 | 0.5 | High RR + Low P | No | 0.5 | 4730 |
| 19111 | Lee County, IA | 1.61 | 0.24 | High RR + Low P | No | 0.5 | 65394 |
| 48181 | Grayson County, TX | 1.257 | 0.48 | Low RR + Low P | No | 0.5 | 290105 |
| 5061 | Howard County, AR | 1.09 | 0.7 | Low RR + Low P | No | 0.499 | 25155 |
| 2016 | Aleutians West Census Area, AK | 1.211 | 0.62 | Low RR + Low P | No | 0.499 | 10250 |
| 12131 | Walton County, FL | 0.639 | 0.94 | Low RR + High P | No | 0.499 | 169758 |
| 19183 | Washington County, IA | 1.084 | 0.66 | Low RR + Low P | No | 0.498 | 45102 |
| 18041 | Fayette County, IN | 1.538 | 0.34 | High RR + Low P | No | 0.498 | 46751 |
| 17025 | Clay County, IL | 0.917 | 0.84 | Low RR + High P | No | 0.498 | 26076 |
| 47013 | Campbell County, TN | 1.601 | 0.2 | High RR + Low P | No | 0.498 | 79787 |
| 28153 | Wayne County, MS | 2.286 | 0.03 | High RR + Low P | No | 0.498 | 39286 |
| 48177 | Gonzales County, TX | 1.524 | 0.37 | High RR + Low P | No | 0.497 | 39746 |
| 21119 | Knott County, KY | 1.825 | 0.19 | High RR + Low P | No | 0.497 | 27506 |
| 51077 | Grayson County, VA | 0.838 | 0.91 | Low RR + High P | No | 0.497 | 30610 |
| 55051 | Iron County, WI | 1.185 | 0.64 | Low RR + Low P | No | 0.497 | 12427 |
| 8021 | Conejos County, CO | 1.068 | 0.73 | Low RR + Low P | No | 0.497 | 15108 |
| 46107 | Potter County, SD | 1.419 | 0.52 | Low RR + Low P | No | 0.497 | 4859 |
| 13027 | Brooks County, GA | 0.864 | 0.88 | Low RR + High P | No | 0.497 | 32529 |
| 18009 | Blackford County, IN | 1.491 | 0.43 | High RR + Low P | No | 0.497 | 23812 |
| 48225 | Houston County, TX | 1.162 | 0.6 | Low RR + Low P | No | 0.497 | 44009 |
| 40025 | Cimarron County, OK | 1.415 | 0.52 | Low RR + Low P | No | 0.496 | 4424 |
| 29053 | Cooper County, MO | 1.039 | 0.72 | Low RR + Low P | No | 0.496 | 33710 |
| 27155 | Traverse County, MN | 1.224 | 0.62 | Low RR + Low P | No | 0.496 | 6378 |
| 28057 | Itawamba County, MS | 1.185 | 0.58 | Low RR + Low P | No | 0.495 | 48064 |
| 31011 | Boone County, NE | 1.197 | 0.62 | Low RR + Low P | No | 0.495 | 10676 |
| 19087 | Henry County, IA | 1.416 | 0.43 | Low RR + Low P | No | 0.495 | 39800 |
| 22023 | Cameron Parish, LA | 1.166 | 0.66 | Low RR + Low P | No | 0.494 | 9664 |
| 51045 | Craig County, VA | 1.107 | 0.71 | Low RR + Low P | No | 0.494 | 9701 |
| 20109 | Logan County, KS | 1.288 | 0.58 | Low RR + Low P | No | 0.494 | 5368 |
| 24027 | Howard County, MD | 0.7 | 0.87 | Low RR + High P | No | 0.494 | 671367 |
| 20129 | Morton County, KS | 1.421 | 0.51 | Low RR + Low P | No | 0.494 | 5181 |
| 41045 | Malheur County, OR | 1.288 | 0.5 | Low RR + Low P | No | 0.494 | 63902 |
| 27033 | Cottonwood County, MN | 1.672 | 0.3 | High RR + Low P | No | 0.494 | 22701 |
| 17109 | McDonough County, IL | 1.27 | 0.52 | Low RR + Low P | No | 0.493 | 53753 |
| 37147 | Pitt County, NC | 1.598 | 0.05 | High RR + Low P | No | 0.493 | 348729 |
| 2240 | Southeast Fairbanks Census Area, AK | 1.146 | 0.66 | Low RR + Low P | No | 0.493 | 14093 |
| 25009 | Essex County, MA | 0.68 | 0.86 | Low RR + High P | No | 0.492 | 1616588 |
| 5001 | Arkansas County, AR | 0.756 | 0.94 | Low RR + High P | No | 0.492 | 32805 |
| 39015 | Brown County, OH | 1.211 | 0.53 | Low RR + Low P | No | 0.492 | 87477 |
| 12039 | Gadsden County, FL | 1.436 | 0.34 | High RR + Low P | No | 0.492 | 87226 |
| 26039 | Crawford County, MI | 1.193 | 0.59 | Low RR + Low P | No | 0.491 | 26972 |
| 18061 | Harrison County, IN | 1.165 | 0.57 | Low RR + Low P | No | 0.491 | 79796 |
| 5105 | Perry County, AR | 0.9 | 0.85 | Low RR + High P | No | 0.491 | 20257 |
| 51091 | Highland County, VA | 1.061 | 0.75 | Low RR + Low P | No | 0.49 | 4635 |
| 12045 | Gulf County, FL | 1.154 | 0.61 | Low RR + Low P | No | 0.49 | 30992 |
| 13061 | Clay County, GA | 1.086 | 0.73 | Low RR + Low P | No | 0.49 | 5706 |
| 20151 | Pratt County, KS | 0.966 | 0.8 | Low RR + Low P | No | 0.49 | 18155 |
| 21057 | Cumberland County, KY | 1.635 | 0.36 | High RR + Low P | No | 0.49 | 11969 |
| 31005 | Arthur County, NE | 1.171 | 0.66 | Low RR + Low P | No | 0.49 | 837 |
| 29167 | Polk County, MO | 0.895 | 0.79 | Low RR + Low P | No | 0.49 | 65439 |
| 48127 | Dimmit County, TX | 1.391 | 0.5 | Low RR + Low P | No | 0.489 | 16617 |
| 39033 | Crawford County, OH | 1.574 | 0.19 | High RR + Low P | No | 0.489 | 83038 |
| 36101 | Steuben County, NY | 1.267 | 0.46 | Low RR + Low P | No | 0.489 | 184667 |
| 48333 | Mills County, TX | 1.061 | 0.74 | Low RR + Low P | No | 0.489 | 9046 |
| 8013 | Boulder County, CO | 0.751 | 0.82 | Low RR + High P | No | 0.489 | 654170 |
| 16063 | Lincoln County, ID | 1.267 | 0.58 | Low RR + Low P | No | 0.488 | 10835 |
| 6009 | Calaveras County, CA | 1.001 | 0.67 | Low RR + Low P | No | 0.488 | 93121 |
| 48221 | Hood County, TX | 1.012 | 0.65 | Low RR + Low P | No | 0.488 | 134168 |
| 26053 | Gogebic County, MI | 1.328 | 0.51 | Low RR + Low P | No | 0.488 | 28601 |
| 19069 | Franklin County, IA | 1.036 | 0.73 | Low RR + Low P | No | 0.488 | 19821 |
| 18103 | Miami County, IN | 0.995 | 0.7 | Low RR + Low P | No | 0.487 | 70948 |
| 19101 | Jefferson County, IA | 0.904 | 0.82 | Low RR + High P | No | 0.487 | 31108 |
| 36077 | Otsego County, NY | 1.133 | 0.57 | Low RR + Low P | No | 0.487 | 120535 |
| 37133 | Onslow County, NC | 1.151 | 0.53 | Low RR + Low P | No | 0.487 | 422707 |
| 41059 | Umatilla County, OR | 1.443 | 0.26 | High RR + Low P | No | 0.486 | 160314 |
| 31125 | Nance County, NE | 1.275 | 0.57 | Low RR + Low P | No | 0.486 | 6591 |
| 36073 | Orleans County, NY | 1.401 | 0.37 | Low RR + Low P | No | 0.486 | 78287 |
| 46117 | Stanley County, SD | 1.078 | 0.72 | Low RR + Low P | No | 0.485 | 6060 |
| 1109 | Pike County, AL | 0.64 | 0.95 | Low RR + High P | No | 0.484 | 66145 |
| 37075 | Graham County, NC | 0.945 | 0.81 | Low RR + High P | No | 0.484 | 16084 |
| 16075 | Payette County, ID | 1.461 | 0.33 | High RR + Low P | No | 0.484 | 54208 |
| 37047 | Columbus County, NC | 1.554 | 0.16 | High RR + Low P | No | 0.484 | 100196 |
| 55093 | Pierce County, WI | 1.256 | 0.49 | Low RR + Low P | No | 0.484 | 85578 |
| 48327 | Menard County, TX | 1.269 | 0.58 | Low RR + Low P | No | 0.484 | 3937 |
| 28097 | Montgomery County, MS | 1.536 | 0.37 | High RR + Low P | No | 0.484 | 19163 |
| 17203 | Woodford County, IL | 1.111 | 0.59 | Low RR + Low P | No | 0.483 | 76462 |
| 55067 | Langlade County, WI | 1.441 | 0.38 | High RR + Low P | No | 0.483 | 38906 |
| 30093 | Silver Bow County, MT | 0.962 | 0.72 | Low RR + Low P | No | 0.483 | 72307 |
| 48345 | Motley County, TX | 1.802 | 0.28 | High RR + Low P | No | 0.482 | 2054 |
| 55001 | Adams County, WI | 1.6 | 0.22 | High RR + Low P | No | 0.482 | 42701 |
| 35037 | Quay County, NM | 0.96 | 0.79 | Low RR + Low P | No | 0.482 | 17039 |
| 37039 | Cherokee County, NC | 1.006 | 0.68 | Low RR + Low P | No | 0.482 | 59418 |
| 19079 | Hamilton County, IA | 1.049 | 0.69 | Low RR + Low P | No | 0.481 | 29532 |
| 13235 | Pulaski County, GA | 1.081 | 0.68 | Low RR + Low P | No | 0.481 | 20058 |
| 48443 | Terrell County, TX | 1.754 | 0.3 | High RR + Low P | No | 0.481 | 1375 |
| 17037 | DeKalb County, IL | 0.762 | 0.83 | Low RR + High P | No | 0.481 | 200511 |
| 31141 | Platte County, NE | 0.923 | 0.75 | Low RR + Low P | No | 0.481 | 68964 |
| 55078 | Menominee County, WI | 1.503 | 0.43 | High RR + Low P | No | 0.481 | 8432 |
| 51013 | Arlington County, VA | 0.383 | 0.94 | Low RR + High P | No | 0.48 | 467840 |
| 30027 | Fergus County, MT | 0.921 | 0.81 | Low RR + High P | No | 0.48 | 23444 |
| 39081 | Jefferson County, OH | 1.388 | 0.31 | Low RR + Low P | No | 0.48 | 128299 |
| 39077 | Huron County, OH | 1.415 | 0.29 | Low RR + Low P | No | 0.48 | 116418 |
| 48099 | Coryell County, TX | 1.65 | 0.02 | High RR + Low P | No | 0.48 | 169083 |
| 46025 | Clark County, SD | 1.191 | 0.61 | Low RR + Low P | No | 0.479 | 7871 |
| 22121 | West Baton Rouge Parish, LA | 0.787 | 0.87 | Low RR + High P | No | 0.479 | 56300 |
| 47137 | Pickett County, TN | 1.099 | 0.69 | Low RR + Low P | No | 0.479 | 10231 |
| 54027 | Hampshire County, WV | 0.996 | 0.7 | Low RR + Low P | No | 0.479 | 47106 |
| 48207 | Haskell County, TX | 1.193 | 0.6 | Low RR + Low P | No | 0.479 | 10761 |
| 49041 | Sevier County, UT | 1.016 | 0.69 | Low RR + Low P | No | 0.478 | 44387 |
| 51600 | Fairfax city, VA | 0.812 | 0.86 | Low RR + High P | No | 0.478 | 49896 |
| 37107 | Lenoir County, NC | 1.691 | 0.02 | High RR + Low P | No | 0.478 | 109394 |
| 48033 | Borden County, TX | 1.563 | 0.41 | High RR + Low P | No | 0.478 | 1152 |
| 6029 | Kern County, CA | 1.576 | 0 | High RR + Low P | No | 0.478 | 1830571 |
| 2290 | Yukon-Koyukuk Census Area, AK | 1.259 | 0.57 | Low RR + Low P | No | 0.477 | 10298 |
| 20171 | Scott County, KS | 1.065 | 0.71 | Low RR + Low P | No | 0.477 | 9929 |
| 20135 | Ness County, KS | 1.263 | 0.57 | Low RR + Low P | No | 0.477 | 5275 |
| 55109 | St. Croix County, WI | 0.929 | 0.7 | Low RR + Low P | No | 0.477 | 192730 |
| 51035 | Carroll County, VA | 0.898 | 0.77 | Low RR + Low P | No | 0.477 | 58357 |
| 16061 | Lewis County, ID | 1.106 | 0.68 | Low RR + Low P | No | 0.476 | 7464 |
| 42057 | Fulton County, PA | 1.126 | 0.62 | Low RR + Low P | No | 0.476 | 29022 |
| 30033 | Garfield County, MT | 1.564 | 0.4 | High RR + Low P | No | 0.476 | 2429 |
| 37003 | Alexander County, NC | 0.858 | 0.79 | Low RR + Low P | No | 0.476 | 72789 |
| 20033 | Comanche County, KS | 1.468 | 0.45 | High RR + Low P | No | 0.476 | 3345 |
| 4011 | Greenlee County, AZ | 1.041 | 0.7 | Low RR + Low P | No | 0.476 | 18696 |
| 48495 | Winkler County, TX | 1.397 | 0.47 | Low RR + Low P | No | 0.476 | 14714 |
| 48113 | Dallas County, TX | 1.468 | 0.06 | High RR + Low P | No | 0.476 | 5208351 |
| 38007 | Billings County, ND | 1.599 | 0.37 | High RR + Low P | No | 0.475 | 2059 |
| 34011 | Cumberland County, NJ | 1.2 | 0.47 | Low RR + Low P | No | 0.475 | 303673 |
| 41067 | Washington County, OR | 1.034 | 0.58 | Low RR + Low P | No | 0.475 | 1198951 |
| 38011 | Bowman County, ND | 1.111 | 0.68 | Low RR + Low P | No | 0.475 | 5766 |
| 50015 | Lamoille County, VT | 1.35 | 0.42 | Low RR + Low P | No | 0.475 | 52163 |
| 29021 | Buchanan County, MO | 0.964 | 0.66 | Low RR + Low P | No | 0.475 | 165847 |
| 17173 | Shelby County, IL | 0.989 | 0.7 | Low RR + Low P | No | 0.475 | 41278 |
| 36019 | Clinton County, NY | 1.191 | 0.5 | Low RR + Low P | No | 0.474 | 156152 |
| 20179 | Sheridan County, KS | 1.205 | 0.6 | Low RR + Low P | No | 0.474 | 4862 |
| 48027 | Bell County, TX | 1.44 | 0.12 | High RR + Low P | No | 0.474 | 780647 |
| 2188 | Northwest Arctic Borough, AK | 1.305 | 0.53 | Low RR + Low P | No | 0.474 | 14814 |
| 54085 | Ritchie County, WV | 1.216 | 0.57 | Low RR + Low P | No | 0.474 | 16397 |
| 26083 | Keweenaw County, MI | 1.415 | 0.48 | Low RR + Low P | No | 0.473 | 4327 |
| 13109 | Evans County, GA | 0.97 | 0.75 | Low RR + Low P | No | 0.473 | 21413 |
| 20077 | Harper County, KS | 1.006 | 0.75 | Low RR + Low P | No | 0.473 | 10835 |
| 22063 | Livingston Parish, LA | 0.304 | 0.94 | Low RR + High P | No | 0.473 | 298201 |
| 51680 | Lynchburg city, VA | 1.198 | 0.49 | Low RR + Low P | No | 0.473 | 158763 |
| 19125 | Marion County, IA | 1.357 | 0.39 | Low RR + Low P | No | 0.473 | 67400 |
| 21011 | Bath County, KY | 1.343 | 0.48 | Low RR + Low P | No | 0.473 | 25803 |
| 17137 | Morgan County, IL | 1.465 | 0.27 | High RR + Low P | No | 0.473 | 64438 |
| 13011 | Banks County, GA | 0.872 | 0.81 | Low RR + High P | No | 0.473 | 39131 |
| 48437 | Swisher County, TX | 1.263 | 0.55 | Low RR + Low P | No | 0.472 | 13914 |
| 55033 | Dunn County, WI | 1.058 | 0.6 | Low RR + Low P | No | 0.472 | 91416 |
| 17191 | Wayne County, IL | 1.458 | 0.35 | High RR + Low P | No | 0.472 | 31604 |
| 55101 | Racine County, WI | 1.282 | 0.36 | Low RR + Low P | No | 0.472 | 392574 |
| 48409 | San Patricio County, TX | 1.445 | 0.19 | High RR + Low P | No | 0.472 | 140747 |
| 16023 | Butte County, ID | 1.057 | 0.72 | Low RR + Low P | No | 0.472 | 5456 |
| 13005 | Bacon County, GA | 0.878 | 0.83 | Low RR + High P | No | 0.471 | 22297 |
| 13033 | Burke County, GA | 1.517 | 0.25 | High RR + Low P | No | 0.471 | 48872 |
| 19167 | Sioux County, IA | 0.96 | 0.69 | Low RR + Low P | No | 0.47 | 72373 |
| 17045 | Edgar County, IL | 1.575 | 0.23 | High RR + Low P | No | 0.47 | 32719 |
| 13217 | Newton County, GA | 0.837 | 0.76 | Low RR + Low P | No | 0.47 | 237887 |
| 47029 | Cocke County, TN | 1.447 | 0.25 | High RR + Low P | No | 0.47 | 74253 |
| 5067 | Jackson County, AR | 1.153 | 0.58 | Low RR + Low P | No | 0.47 | 33438 |
| 37129 | New Hanover County, NC | 0.576 | 0.88 | Low RR + High P | No | 0.47 | 474636 |
| 55099 | Price County, WI | 0.977 | 0.72 | Low RR + Low P | No | 0.47 | 28261 |
| 1071 | Jackson County, AL | 0.629 | 0.9 | Low RR + High P | No | 0.47 | 106390 |
| 31099 | Kearney County, NE | 1.364 | 0.48 | Low RR + Low P | No | 0.47 | 13476 |
| 30053 | Lincoln County, MT | 0.673 | 0.93 | Low RR + High P | No | 0.469 | 43405 |
| 48205 | Hartley County, TX | 1.17 | 0.6 | Low RR + Low P | No | 0.469 | 10373 |
| 51149 | Prince George County, VA | 0.591 | 0.92 | Low RR + High P | No | 0.469 | 85892 |
| 48131 | Duval County, TX | 1.187 | 0.57 | Low RR + Low P | No | 0.469 | 19310 |
| 29107 | Lafayette County, MO | 1.107 | 0.58 | Low RR + Low P | No | 0.468 | 66163 |
| 46005 | Beadle County, SD | 1.01 | 0.68 | Low RR + Low P | No | 0.468 | 39079 |
| 39121 | Noble County, OH | 0.822 | 0.86 | Low RR + High P | No | 0.467 | 28636 |
| 51570 | Colonial Heights city, VA | 0.733 | 0.91 | Low RR + High P | No | 0.467 | 36671 |
| 27051 | Grant County, MN | 0.986 | 0.75 | Low RR + Low P | No | 0.467 | 12260 |
| 6007 | Butte County, CA | 1.527 | 0.02 | High RR + Low P | No | 0.467 | 414541 |
| 23031 | York County, ME | 0.643 | 0.84 | Low RR + High P | No | 0.466 | 435536 |
| 30059 | Meagher County, MT | 1.333 | 0.52 | Low RR + Low P | No | 0.466 | 4097 |
| 21109 | Jackson County, KY | 0.875 | 0.82 | Low RR + High P | No | 0.466 | 26093 |
| 19003 | Adams County, IA | 1.151 | 0.62 | Low RR + Low P | No | 0.466 | 7145 |
| 38031 | Foster County, ND | 1.188 | 0.59 | Low RR + Low P | No | 0.466 | 6689 |
| 13089 | DeKalb County, GA | 0.745 | 0.78 | Low RR + Low P | No | 0.466 | 1524771 |
| 19083 | Hardin County, IA | 1.367 | 0.42 | Low RR + Low P | No | 0.466 | 33077 |
| 48385 | Real County, TX | 1.214 | 0.58 | Low RR + Low P | No | 0.465 | 5686 |
| 41023 | Grant County, OR | 1.404 | 0.44 | Low RR + Low P | No | 0.465 | 14440 |
| 39079 | Jackson County, OH | 1.256 | 0.47 | Low RR + Low P | No | 0.465 | 65217 |
| 16069 | Nez Perce County, ID | 1.268 | 0.44 | Low RR + Low P | No | 0.465 | 85896 |
| 18183 | Whitley County, IN | 0.896 | 0.74 | Low RR + Low P | No | 0.465 | 69319 |
| 5119 | Pulaski County, AR | 1.411 | 0.12 | Low RR + Low P | No | 0.465 | 799396 |
| 49047 | Uintah County, UT | 0.858 | 0.77 | Low RR + Low P | No | 0.464 | 74851 |
| 1031 | Coffee County, AL | 0.547 | 0.92 | Low RR + High P | No | 0.464 | 110483 |
| 48493 | Wilson County, TX | 0.928 | 0.7 | Low RR + Low P | No | 0.464 | 106937 |
| 47001 | Anderson County, TN | 1.27 | 0.4 | Low RR + Low P | No | 0.464 | 159163 |
| 18131 | Pulaski County, IN | 1.38 | 0.42 | Low RR + Low P | No | 0.463 | 24834 |
| 19105 | Jones County, IA | 1.372 | 0.38 | Low RR + Low P | No | 0.463 | 41849 |
| 37177 | Tyrrell County, NC | 1.195 | 0.59 | Low RR + Low P | No | 0.463 | 6934 |
| 51109 | Louisa County, VA | 0.644 | 0.9 | Low RR + High P | No | 0.463 | 81067 |
| 12107 | Putnam County, FL | 1.063 | 0.57 | Low RR + Low P | No | 0.463 | 150691 |
| 48399 | Runnels County, TX | 1.024 | 0.69 | Low RR + Low P | No | 0.463 | 19752 |
| 29099 | Jefferson County, MO | 1.019 | 0.57 | Low RR + Low P | No | 0.462 | 460450 |
| 17069 | Hardin County, IL | 1.494 | 0.39 | High RR + Low P | No | 0.462 | 7141 |
| 29061 | Daviess County, MO | 1.138 | 0.6 | Low RR + Low P | No | 0.462 | 17008 |
| 51113 | Madison County, VA | 0.811 | 0.86 | Low RR + High P | No | 0.462 | 28136 |
| 19049 | Dallas County, IA | 0.667 | 0.84 | Low RR + High P | No | 0.462 | 219238 |
| 13169 | Jones County, GA | 0.852 | 0.78 | Low RR + Low P | No | 0.462 | 57481 |
| 30101 | Toole County, MT | 1.082 | 0.66 | Low RR + Low P | No | 0.461 | 10210 |
| 30091 | Sheridan County, MT | 1.181 | 0.59 | Low RR + Low P | No | 0.461 | 7053 |
| 36005 | Bronx County, NY | 1.501 | 0 | High RR + Low P | No | 0.461 | 2738284 |
| 19035 | Cherokee County, IA | 1.057 | 0.65 | Low RR + Low P | No | 0.461 | 23095 |
| 20125 | Montgomery County, KS | 1.073 | 0.59 | Low RR + Low P | No | 0.461 | 61421 |
| 20105 | Lincoln County, KS | 1.244 | 0.56 | Low RR + Low P | No | 0.46 | 5831 |
| 54103 | Wetzel County, WV | 1.315 | 0.47 | Low RR + Low P | No | 0.46 | 27884 |
| 38009 | Bottineau County, ND | 1.144 | 0.61 | Low RR + Low P | No | 0.46 | 12733 |
| 6021 | Glenn County, CA | 1.552 | 0.12 | High RR + Low P | No | 0.459 | 56489 |
| 51071 | Giles County, VA | 1.434 | 0.32 | High RR + Low P | No | 0.459 | 32889 |
| 6091 | Sierra County, CA | 1.157 | 0.6 | Low RR + Low P | No | 0.459 | 6422 |
| 13095 | Dougherty County, GA | 0.733 | 0.81 | Low RR + High P | No | 0.459 | 165540 |
| 48153 | Floyd County, TX | 1.174 | 0.59 | Low RR + Low P | No | 0.459 | 10329 |
| 26139 | Ottawa County, MI | 0.932 | 0.63 | Low RR + Low P | No | 0.459 | 604290 |
| 55053 | Jackson County, WI | 1.202 | 0.52 | Low RR + Low P | No | 0.459 | 41748 |
| 48285 | Lavaca County, TX | 1.307 | 0.43 | Low RR + Low P | No | 0.458 | 41224 |
| 20207 | Woodson County, KS | 1.258 | 0.55 | Low RR + Low P | No | 0.458 | 6232 |
| 29043 | Christian County, MO | 0.821 | 0.75 | Low RR + Low P | No | 0.457 | 187544 |
| 26089 | Leelanau County, MI | 0.842 | 0.8 | Low RR + Low P | No | 0.457 | 45902 |
| 38061 | Mountrail County, ND | 0.95 | 0.75 | Low RR + Low P | No | 0.457 | 18624 |
| 13117 | Forsyth County, GA | 0.434 | 0.89 | Low RR + High P | No | 0.457 | 540199 |
| 26095 | Luce County, MI | 1.38 | 0.44 | Low RR + Low P | No | 0.457 | 12709 |
| 55085 | Oneida County, WI | 1.474 | 0.16 | High RR + Low P | No | 0.457 | 76419 |
| 19137 | Montgomery County, IA | 1.361 | 0.43 | Low RR + Low P | No | 0.457 | 20349 |
| 28075 | Lauderdale County, MS | 0.264 | 0.93 | Low RR + High P | No | 0.456 | 141669 |
| 20053 | Ellsworth County, KS | 0.842 | 0.86 | Low RR + High P | No | 0.456 | 12740 |
| 51083 | Halifax County, VA | 0.905 | 0.72 | Low RR + Low P | No | 0.456 | 67052 |
| 37149 | Polk County, NC | 0.861 | 0.78 | Low RR + Low P | No | 0.456 | 40072 |
| 47161 | Stewart County, TN | 1.482 | 0.28 | High RR + Low P | No | 0.455 | 28216 |
| 22085 | Sabine Parish, LA | 0.712 | 0.88 | Low RR + High P | No | 0.455 | 43880 |
| 21145 | McCracken County, KY | 1.361 | 0.25 | Low RR + Low P | No | 0.455 | 134794 |
| 21231 | Wayne County, KY | 1.253 | 0.48 | Low RR + Low P | No | 0.454 | 39214 |
| 48489 | Willacy County, TX | 1.373 | 0.36 | Low RR + Low P | No | 0.454 | 40291 |
| 5145 | White County, AR | 1.53 | 0.02 | High RR + Low P | No | 0.454 | 156068 |
| 53025 | Grant County, WA | 1.377 | 0.18 | Low RR + Low P | No | 0.454 | 204258 |
| 30009 | Carbon County, MT | 0.807 | 0.86 | Low RR + High P | No | 0.454 | 22655 |
| 31033 | Cheyenne County, NE | 1.112 | 0.6 | Low RR + Low P | No | 0.454 | 19067 |
| 26023 | Branch County, MI | 1.223 | 0.44 | Low RR + Low P | No | 0.453 | 89717 |
| 6047 | Merced County, CA | 1.527 | 0 | High RR + Low P | No | 0.453 | 582130 |
| 39141 | Ross County, OH | 1.329 | 0.28 | Low RR + Low P | No | 0.453 | 153009 |
| 31017 | Brown County, NE | 1.468 | 0.38 | High RR + Low P | No | 0.453 | 5736 |
| 48121 | Denton County, TX | 0.706 | 0.77 | Low RR + Low P | No | 0.453 | 1985463 |
| 21105 | Hickman County, KY | 1.149 | 0.6 | Low RR + Low P | No | 0.452 | 8881 |
| 48015 | Austin County, TX | 1.004 | 0.62 | Low RR + Low P | No | 0.452 | 62747 |
| 18175 | Washington County, IN | 1.431 | 0.23 | Low RR + Low P | No | 0.452 | 56376 |
| 39045 | Fairfield County, OH | 1.352 | 0.18 | Low RR + Low P | No | 0.452 | 328316 |
| 51590 | Danville city, VA | 1.263 | 0.41 | Low RR + Low P | No | 0.452 | 83854 |
| 19093 | Ida County, IA | 0.922 | 0.78 | Low RR + Low P | No | 0.452 | 13738 |
| 20079 | Harvey County, KS | 0.947 | 0.67 | Low RR + Low P | No | 0.451 | 67175 |
| 18001 | Adams County, IN | 1.119 | 0.55 | Low RR + Low P | No | 0.451 | 72439 |
| 48089 | Colorado County, TX | 1.398 | 0.3 | Low RR + Low P | No | 0.451 | 41949 |
| 29225 | Webster County, MO | 0.775 | 0.8 | Low RR + Low P | No | 0.451 | 81838 |
| 17127 | Massac County, IL | 1.297 | 0.46 | Low RR + Low P | No | 0.451 | 27488 |
| 13229 | Pierce County, GA | 1.092 | 0.59 | Low RR + Low P | No | 0.451 | 40615 |
| 2195 | Petersburg Borough/Census Area, AK | 1.17 | 0.59 | Low RR + Low P | No | 0.45 | 6797 |
| 54021 | Gilmer County, WV | 1.354 | 0.44 | Low RR + Low P | No | 0.45 | 14584 |
| 46093 | Meade County, SD | 0.966 | 0.65 | Low RR + Low P | No | 0.45 | 61656 |
| 6039 | Madera County, CA | 1.418 | 0.06 | Low RR + Low P | No | 0.45 | 323187 |
| 29025 | Caldwell County, MO | 0.852 | 0.82 | Low RR + High P | No | 0.45 | 17894 |
| 1087 | Macon County, AL | 0.784 | 0.83 | Low RR + High P | No | 0.449 | 36902 |
| 18023 | Clinton County, IN | 1.161 | 0.52 | Low RR + Low P | No | 0.449 | 65499 |
| 38049 | McHenry County, ND | 1.159 | 0.59 | Low RR + Low P | No | 0.449 | 10326 |
| 29215 | Texas County, MO | 0.988 | 0.64 | Low RR + Low P | No | 0.448 | 50923 |
| 5063 | Independence County, AR | 0.495 | 0.92 | Low RR + High P | No | 0.448 | 76574 |
| 42049 | Erie County, PA | 0.856 | 0.69 | Low RR + Low P | No | 0.448 | 536411 |
| 21031 | Butler County, KY | 0.939 | 0.73 | Low RR + Low P | No | 0.448 | 24740 |
| 54029 | Hancock County, WV | 0.865 | 0.75 | Low RR + Low P | No | 0.448 | 56399 |
| 30085 | Roosevelt County, MT | 1.463 | 0.3 | High RR + Low P | No | 0.447 | 20856 |
| 30043 | Jefferson County, MT | 0.84 | 0.81 | Low RR + High P | No | 0.447 | 25879 |
| 17183 | Vermilion County, IL | 1.434 | 0.08 | High RR + Low P | No | 0.447 | 143755 |
| 17135 | Montgomery County, IL | 0.932 | 0.69 | Low RR + Low P | No | 0.447 | 55564 |
| 37143 | Perquimans County, NC | 1.228 | 0.5 | Low RR + Low P | No | 0.447 | 26598 |
| 12081 | Manatee County, FL | 0.501 | 0.84 | Low RR + High P | No | 0.447 | 870264 |
| 53065 | Stevens County, WA | 1.194 | 0.45 | Low RR + Low P | No | 0.447 | 97120 |
| 46109 | Roberts County, SD | 0.887 | 0.78 | Low RR + Low P | No | 0.447 | 20345 |
| 54063 | Monroe County, WV | 0.99 | 0.68 | Low RR + Low P | No | 0.446 | 24704 |
| 6015 | Del Norte County, CA | 1.484 | 0.14 | High RR + Low P | No | 0.446 | 53701 |
| 22113 | Vermilion Parish, LA | 0.52 | 0.89 | Low RR + High P | No | 0.446 | 113945 |
| 20089 | Jewell County, KS | 1.147 | 0.6 | Low RR + Low P | No | 0.446 | 5732 |
| 17003 | Alexander County, IL | 1.177 | 0.57 | Low RR + Low P | No | 0.446 | 9536 |
| 39097 | Madison County, OH | 1.049 | 0.57 | Low RR + Low P | No | 0.445 | 88183 |
| 27001 | Aitkin County, MN | 0.955 | 0.7 | Low RR + Low P | No | 0.445 | 32244 |
| 48427 | Starr County, TX | 0.813 | 0.74 | Low RR + Low P | No | 0.445 | 131587 |
| 39063 | Hancock County, OH | 1.216 | 0.4 | Low RR + Low P | No | 0.445 | 149478 |
| 48135 | Ector County, TX | 1.518 | 0 | High RR + Low P | No | 0.445 | 325551 |
| 49033 | Rich County, UT | 1.411 | 0.41 | Low RR + Low P | No | 0.445 | 5319 |
| 26007 | Alpena County, MI | 1.436 | 0.18 | High RR + Low P | No | 0.444 | 57737 |
| 18117 | Orange County, IN | 1.402 | 0.28 | Low RR + Low P | No | 0.444 | 39239 |
| 54109 | Wyoming County, WV | 1.29 | 0.42 | Low RR + Low P | No | 0.444 | 40849 |
| 20075 | Hamilton County, KS | 1.092 | 0.63 | Low RR + Low P | No | 0.444 | 4889 |
| 37049 | Craven County, NC | 1.288 | 0.28 | Low RR + Low P | No | 0.443 | 203478 |
| 48359 | Oldham County, TX | 1.553 | 0.28 | High RR + Low P | No | 0.443 | 3544 |
| 18099 | Marshall County, IN | 0.979 | 0.6 | Low RR + Low P | No | 0.443 | 92730 |
| 19085 | Harrison County, IA | 0.931 | 0.72 | Low RR + Low P | No | 0.442 | 29350 |
| 51630 | Fredericksburg city, VA | 0.705 | 0.84 | Low RR + High P | No | 0.442 | 57576 |
| 22087 | St. Bernard Parish, LA | 1.129 | 0.52 | Low RR + Low P | No | 0.442 | 88882 |
| 18139 | Rush County, IN | 1.374 | 0.32 | Low RR + Low P | No | 0.442 | 33519 |
| 8025 | Crowley County, CO | 1.186 | 0.56 | Low RR + Low P | No | 0.442 | 11263 |
| 29117 | Livingston County, MO | 0.935 | 0.72 | Low RR + Low P | No | 0.442 | 28892 |
| 13019 | Berrien County, GA | 1.151 | 0.54 | Low RR + Low P | No | 0.442 | 36785 |
| 48075 | Childress County, TX | 0.981 | 0.7 | Low RR + Low P | No | 0.442 | 13629 |
| 48109 | Culberson County, TX | 1.212 | 0.55 | Low RR + Low P | No | 0.441 | 4359 |
| 51115 | Mathews County, VA | 0.829 | 0.82 | Low RR + High P | No | 0.441 | 16964 |
| 1123 | Tallapoosa County, AL | 0.797 | 0.76 | Low RR + Low P | No | 0.44 | 81590 |
| 6089 | Shasta County, CA | 1.41 | 0.03 | Low RR + Low P | No | 0.44 | 361303 |
| 21077 | Gallatin County, KY | 1.512 | 0.24 | High RR + Low P | No | 0.44 | 17565 |
| 51169 | Scott County, VA | 1.211 | 0.47 | Low RR + Low P | No | 0.44 | 42913 |
| 5137 | Stone County, AR | 0.783 | 0.84 | Low RR + High P | No | 0.44 | 25256 |
| 37181 | Vance County, NC | 1.136 | 0.51 | Low RR + Low P | No | 0.439 | 84455 |
| 20027 | Clay County, KS | 0.892 | 0.77 | Low RR + Low P | No | 0.439 | 16048 |
| 48195 | Hansford County, TX | 1.174 | 0.56 | Low RR + Low P | No | 0.439 | 10182 |
| 37187 | Washington County, NC | 1.554 | 0.15 | High RR + Low P | No | 0.438 | 21489 |
| 48273 | Kleberg County, TX | 1.403 | 0.19 | Low RR + Low P | No | 0.438 | 60445 |
| 22089 | St. Charles Parish, LA | 0.478 | 0.89 | Low RR + High P | No | 0.438 | 101678 |
| 13165 | Jenkins County, GA | 0.91 | 0.75 | Low RR + Low P | No | 0.438 | 17282 |
| 48411 | San Saba County, TX | 1.149 | 0.57 | Low RR + Low P | No | 0.438 | 11725 |
| 42027 | Centre County, PA | 1.093 | 0.5 | Low RR + Low P | No | 0.438 | 315328 |
| 54003 | Berkeley County, WV | 1.348 | 0.14 | Low RR + Low P | No | 0.437 | 261819 |
| 4017 | Navajo County, AZ | 1.017 | 0.56 | Low RR + Low P | No | 0.437 | 217975 |
| 55003 | Ashland County, WI | 1.274 | 0.43 | Low RR + Low P | No | 0.437 | 32119 |
| 37169 | Stokes County, NC | 0.999 | 0.59 | Low RR + Low P | No | 0.437 | 90668 |
| 30045 | Judith Basin County, MT | 1.416 | 0.38 | Low RR + Low P | No | 0.437 | 4172 |
| 24011 | Caroline County, MD | 1.371 | 0.22 | Low RR + Low P | No | 0.437 | 67020 |
| 5017 | Chicot County, AR | 0.965 | 0.69 | Low RR + Low P | No | 0.436 | 19372 |
| 27173 | Yellow Medicine County, MN | 0.846 | 0.8 | Low RR + Low P | No | 0.436 | 18951 |
| 17201 | Winnebago County, IL | 1.036 | 0.52 | Low RR + Low P | No | 0.436 | 562464 |
| 19129 | Mills County, IA | 0.826 | 0.79 | Low RR + Low P | No | 0.436 | 29168 |
| 48355 | Nueces County, TX | 1.396 | 0.02 | Low RR + Low P | No | 0.436 | 703879 |
| 16043 | Fremont County, ID | 0.851 | 0.78 | Low RR + Low P | No | 0.436 | 28213 |
| 22037 | East Feliciana Parish, LA | 1.113 | 0.55 | Low RR + Low P | No | 0.436 | 38437 |
| 48477 | Washington County, TX | 0.958 | 0.62 | Low RR + Low P | No | 0.435 | 73375 |
| 6011 | Colusa County, CA | 1.3 | 0.37 | Low RR + Low P | No | 0.435 | 43946 |
| 21155 | Marion County, KY | 1.306 | 0.37 | Low RR + Low P | No | 0.435 | 39582 |
| 20119 | Meade County, KS | 1.2 | 0.54 | Low RR + Low P | No | 0.435 | 7824 |
| 54091 | Taylor County, WV | 1.242 | 0.45 | Low RR + Low P | No | 0.435 | 32798 |
| 42023 | Cameron County, PA | 1.037 | 0.65 | Low RR + Low P | No | 0.435 | 8786 |
| 32013 | Humboldt County, NV | 0.946 | 0.69 | Low RR + Low P | No | 0.435 | 34432 |
| 6071 | San Bernardino County, CA | 1.384 | 0.01 | Low RR + Low P | No | 0.435 | 4390519 |
| 13259 | Stewart County, GA | 1.1 | 0.6 | Low RR + Low P | No | 0.434 | 9278 |
| 20101 | Lane County, KS | 1.414 | 0.38 | Low RR + Low P | No | 0.434 | 3073 |
| 1075 | Lamar County, AL | 0.861 | 0.76 | Low RR + Low P | No | 0.434 | 27367 |
| 37159 | Rowan County, NC | 1.044 | 0.52 | Low RR + Low P | No | 0.434 | 301334 |
| 21129 | Lee County, KY | 1.445 | 0.29 | High RR + Low P | No | 0.434 | 14566 |
| 48129 | Donley County, TX | 1.218 | 0.53 | Low RR + Low P | No | 0.434 | 6503 |
| 18011 | Boone County, IN | 0.961 | 0.59 | Low RR + Low P | No | 0.433 | 150429 |
| 13219 | Oconee County, GA | 0.552 | 0.86 | Low RR + High P | No | 0.433 | 87806 |
| 1093 | Marion County, AL | 0.517 | 0.9 | Low RR + High P | No | 0.433 | 58426 |
| 48151 | Fisher County, TX | 1.14 | 0.58 | Low RR + Low P | No | 0.433 | 7238 |
| 13167 | Johnson County, GA | 1.174 | 0.54 | Low RR + Low P | No | 0.433 | 18493 |
| 29207 | Stoddard County, MO | 0.703 | 0.82 | Low RR + High P | No | 0.433 | 56787 |
| 48465 | Val Verde County, TX | 1.22 | 0.38 | Low RR + Low P | No | 0.433 | 95337 |
| 12091 | Okaloosa County, FL | 0.767 | 0.72 | Low RR + Low P | No | 0.433 | 434928 |
| 20205 | Wilson County, KS | 1.371 | 0.36 | Low RR + Low P | No | 0.433 | 16936 |
| 47091 | Johnson County, TN | 0.959 | 0.66 | Low RR + Low P | No | 0.433 | 36421 |
| 36113 | Warren County, NY | 1.144 | 0.46 | Low RR + Low P | No | 0.432 | 130807 |
| 37017 | Bladen County, NC | 1.454 | 0.08 | High RR + Low P | No | 0.432 | 58875 |
| 37051 | Cumberland County, NC | 1.405 | 0 | Low RR + Low P | No | 0.432 | 674671 |
| 21179 | Nelson County, KY | 1.116 | 0.5 | Low RR + Low P | No | 0.432 | 95089 |
| 31061 | Franklin County, NE | 1.404 | 0.37 | Low RR + Low P | No | 0.431 | 5675 |
| 26047 | Emmet County, MI | 1.163 | 0.47 | Low RR + Low P | No | 0.431 | 68338 |
| 42077 | Lehigh County, PA | 0.969 | 0.56 | Low RR + Low P | No | 0.431 | 754028 |
| 38081 | Sargent County, ND | 1.165 | 0.56 | Low RR + Low P | No | 0.43 | 7580 |
| 8023 | Costilla County, CO | 1.177 | 0.55 | Low RR + Low P | No | 0.43 | 7243 |
| 6085 | Santa Clara County, CA | 0.867 | 0.62 | Low RR + Low P | No | 0.43 | 3755927 |
| 5023 | Cleburne County, AR | 1.052 | 0.57 | Low RR + Low P | No | 0.43 | 50698 |
| 47107 | McMinn County, TN | 1.133 | 0.48 | Low RR + Low P | No | 0.43 | 110373 |
| 49011 | Davis County, UT | 0.744 | 0.72 | Low RR + Low P | No | 0.43 | 743094 |
| 53055 | San Juan County, WA | 0.489 | 0.92 | Low RR + High P | No | 0.429 | 37238 |
| 29219 | Warren County, MO | 0.681 | 0.81 | Low RR + High P | No | 0.429 | 75096 |
| 37013 | Beaufort County, NC | 1.368 | 0.15 | Low RR + Low P | No | 0.429 | 88881 |
| 17087 | Johnson County, IL | 0.784 | 0.81 | Low RR + High P | No | 0.428 | 26717 |
| 26105 | Mason County, MI | 1.075 | 0.55 | Low RR + Low P | No | 0.428 | 58477 |
| 39023 | Clark County, OH | 1.337 | 0.1 | Low RR + Low P | No | 0.428 | 269342 |
| 34001 | Atlantic County, NJ | 0.808 | 0.68 | Low RR + Low P | No | 0.428 | 550595 |
| 48107 | Crosby County, TX | 1.378 | 0.37 | Low RR + Low P | No | 0.428 | 9909 |
| 22107 | Tensas Parish, LA | 1.215 | 0.52 | Low RR + Low P | No | 0.428 | 7578 |
| 48241 | Jasper County, TX | 1.404 | 0.13 | Low RR + Low P | No | 0.428 | 65189 |
| 49005 | Cache County, UT | 0.828 | 0.68 | Low RR + Low P | No | 0.427 | 282538 |
| 2198 | Prince of Wales-Hyder Census Area, AK | 1.075 | 0.6 | Low RR + Low P | No | 0.427 | 11346 |
| 35055 | Taos County, NM | 0.593 | 0.84 | Low RR + High P | No | 0.427 | 68995 |
| 48213 | Henderson County, TX | 1.305 | 0.18 | Low RR + Low P | No | 0.427 | 170648 |
| 17129 | Menard County, IL | 1.019 | 0.61 | Low RR + Low P | No | 0.426 | 24026 |
| 21117 | Kenton County, KY | 1.434 | 0 | High RR + Low P | No | 0.426 | 341601 |
| 5055 | Greene County, AR | 1.325 | 0.2 | Low RR + Low P | No | 0.425 | 93151 |
| 27143 | Sibley County, MN | 1.202 | 0.47 | Low RR + Low P | No | 0.425 | 30012 |
| 31181 | Webster County, NE | 1.383 | 0.36 | Low RR + Low P | No | 0.425 | 6690 |
| 13211 | Morgan County, GA | 0.693 | 0.82 | Low RR + High P | No | 0.424 | 42484 |
| 32005 | Douglas County, NV | 0.68 | 0.78 | Low RR + Low P | No | 0.424 | 99207 |
| 49027 | Millard County, UT | 1.19 | 0.49 | Low RR + Low P | No | 0.424 | 26780 |
| 51043 | Clarke County, VA | 0.814 | 0.77 | Low RR + Low P | No | 0.424 | 30797 |
| 37127 | Nash County, NC | 1.228 | 0.29 | Low RR + Low P | No | 0.424 | 192371 |
| 12123 | Taylor County, FL | 0.932 | 0.66 | Low RR + Low P | No | 0.423 | 42895 |
| 47141 | Putnam County, TN | 1.023 | 0.53 | Low RR + Low P | No | 0.423 | 166314 |
| 22109 | Terrebonne Parish, LA | 1.141 | 0.42 | Low RR + Low P | No | 0.423 | 208248 |
| 19067 | Floyd County, IA | 1.019 | 0.6 | Low RR + Low P | No | 0.423 | 30674 |
| 21185 | Oldham County, KY | 0.701 | 0.76 | Low RR + Low P | No | 0.423 | 139627 |
| 39029 | Columbiana County, OH | 1.264 | 0.22 | Low RR + Low P | No | 0.422 | 200751 |
| 23007 | Franklin County, ME | 0.826 | 0.72 | Low RR + Low P | No | 0.422 | 61423 |
| 31023 | Butler County, NE | 1.415 | 0.27 | Low RR + Low P | No | 0.422 | 16902 |
| 54019 | Fayette County, WV | 1.203 | 0.39 | Low RR + Low P | No | 0.422 | 78460 |
| 5051 | Garland County, AR | 0.89 | 0.62 | Low RR + Low P | No | 0.422 | 199766 |
| 5121 | Randolph County, AR | 0.736 | 0.81 | Low RR + High P | No | 0.422 | 37759 |
| 13207 | Monroe County, GA | 0.89 | 0.67 | Low RR + Low P | No | 0.421 | 60094 |
| 35019 | Guadalupe County, NM | 1.098 | 0.59 | Low RR + Low P | No | 0.421 | 8608 |
| 47083 | Houston County, TN | 1.294 | 0.42 | Low RR + Low P | No | 0.42 | 16640 |
| 56025 | Natrona County, WY | 1.356 | 0.06 | Low RR + Low P | No | 0.42 | 159464 |
| 31027 | Cedar County, NE | 1.231 | 0.47 | Low RR + Low P | No | 0.42 | 16628 |
| 5135 | Sharp County, AR | 0.71 | 0.82 | Low RR + High P | No | 0.42 | 35804 |
| 55129 | Washburn County, WI | 1.011 | 0.59 | Low RR + Low P | No | 0.42 | 33801 |
| 26109 | Menominee County, MI | 1.204 | 0.42 | Low RR + Low P | No | 0.419 | 46170 |
| 27015 | Brown County, MN | 1.307 | 0.28 | Low RR + Low P | No | 0.419 | 51390 |
| 21127 | Lawrence County, KY | 1.361 | 0.26 | Low RR + Low P | No | 0.419 | 32097 |
| 48337 | Montague County, TX | 1.303 | 0.31 | Low RR + Low P | No | 0.419 | 42677 |
| 39135 | Preble County, OH | 1.255 | 0.3 | Low RR + Low P | No | 0.419 | 81130 |
| 48245 | Jefferson County, TX | 1.335 | 0.03 | Low RR + Low P | No | 0.419 | 503097 |
| 48053 | Burnet County, TX | 1.177 | 0.39 | Low RR + Low P | No | 0.419 | 106558 |
| 26073 | Isabella County, MI | 0.984 | 0.56 | Low RR + Low P | No | 0.418 | 128484 |
| 38051 | McIntosh County, ND | 1.129 | 0.57 | Low RR + Low P | No | 0.418 | 4983 |
| 8121 | Washington County, CO | 1.01 | 0.64 | Low RR + Low P | No | 0.418 | 9702 |
| 5123 | St. Francis County, AR | 1.033 | 0.57 | Low RR + Low P | No | 0.418 | 44503 |
| 45065 | McCormick County, SC | 1.062 | 0.58 | Low RR + Low P | No | 0.418 | 19682 |
| 36003 | Allegany County, NY | 0.857 | 0.67 | Low RR + Low P | No | 0.418 | 93377 |
| 8093 | Park County, CO | 0.675 | 0.83 | Low RR + High P | No | 0.417 | 36038 |
| 13021 | Bibb County, GA | 0.616 | 0.77 | Low RR + Low P | No | 0.417 | 312638 |
| 13253 | Seminole County, GA | 0.771 | 0.81 | Low RR + High P | No | 0.417 | 18236 |
| 18051 | Gibson County, IN | 1.37 | 0.13 | Low RR + Low P | No | 0.416 | 65906 |
| 21165 | Menifee County, KY | 1.248 | 0.46 | Low RR + Low P | No | 0.416 | 12522 |
| 48283 | La Salle County, TX | 1.201 | 0.5 | Low RR + Low P | No | 0.416 | 13093 |
| 47035 | Cumberland County, TN | 0.7 | 0.76 | Low RR + Low P | No | 0.416 | 128242 |
| 20173 | Sedgwick County, KS | 0.972 | 0.53 | Low RR + Low P | No | 0.416 | 1053837 |
| 42073 | Lawrence County, PA | 1.155 | 0.39 | Low RR + Low P | No | 0.416 | 169284 |
| 18109 | Morgan County, IN | 1.186 | 0.34 | Low RR + Low P | No | 0.415 | 145487 |
| 38055 | McLean County, ND | 0.911 | 0.7 | Low RR + Low P | No | 0.415 | 19690 |
| 19059 | Dickinson County, IA | 0.705 | 0.81 | Low RR + High P | No | 0.415 | 36104 |
| 18151 | Steuben County, IN | 0.993 | 0.57 | Low RR + Low P | No | 0.413 | 69689 |
| 48029 | Bexar County, TX | 1.357 | 0 | Low RR + Low P | No | 0.413 | 4147870 |
| 28027 | Coahoma County, MS | 0.912 | 0.66 | Low RR + Low P | No | 0.413 | 40312 |
| 51065 | Fluvanna County, VA | 0.592 | 0.83 | Low RR + High P | No | 0.413 | 56579 |
| 29510 | St. Louis city, MO | 1.145 | 0.35 | Low RR + Low P | No | 0.413 | 567947 |
| 31165 | Sioux County, NE | 1.149 | 0.56 | Low RR + Low P | No | 0.413 | 2281 |
| 17015 | Carroll County, IL | 1.156 | 0.49 | Low RR + Low P | No | 0.413 | 31105 |
| 31105 | Kimball County, NE | 1.125 | 0.56 | Low RR + Low P | No | 0.412 | 6625 |
| 48041 | Brazos County, TX | 1.316 | 0.04 | Low RR + Low P | No | 0.412 | 486634 |
| 27081 | Lincoln County, MN | 1.366 | 0.32 | Low RR + Low P | No | 0.412 | 11097 |
| 56023 | Lincoln County, WY | 0.753 | 0.77 | Low RR + Low P | No | 0.412 | 41551 |
| 37195 | Wilson County, NC | 1.338 | 0.05 | Low RR + Low P | No | 0.411 | 157276 |
| 35031 | McKinley County, NM | 1.115 | 0.43 | Low RR + Low P | No | 0.411 | 138728 |
| 26097 | Mackinac County, MI | 1.218 | 0.44 | Low RR + Low P | No | 0.411 | 21813 |
| 47087 | Jackson County, TN | 1.389 | 0.21 | Low RR + Low P | No | 0.41 | 24378 |
| 1127 | Walker County, AL | 0.561 | 0.8 | Low RR + Low P | No | 0.41 | 129166 |
| 28151 | Washington County, MS | 0.788 | 0.71 | Low RR + Low P | No | 0.41 | 84470 |
| 17119 | Madison County, IL | 0.913 | 0.57 | Low RR + Low P | No | 0.41 | 526324 |
| 23025 | Somerset County, ME | 1.153 | 0.41 | Low RR + Low P | No | 0.41 | 102420 |
| 6103 | Tehama County, CA | 1.331 | 0.07 | Low RR + Low P | No | 0.41 | 130195 |
| 51167 | Russell County, VA | 0.91 | 0.64 | Low RR + Low P | No | 0.409 | 50914 |
| 50007 | Chittenden County, VT | 1.155 | 0.33 | Low RR + Low P | No | 0.409 | 338838 |
| 18179 | Wells County, IN | 1.12 | 0.48 | Low RR + Low P | No | 0.409 | 56853 |
| 51595 | Emporia city, VA | 0.939 | 0.69 | Low RR + Low P | No | 0.408 | 10996 |
| 50017 | Orange County, VT | 1.417 | 0.03 | Low RR + Low P | No | 0.408 | 59823 |
| 48119 | Delta County, TX | 1.397 | 0.26 | Low RR + Low P | No | 0.408 | 10933 |
| 1013 | Butler County, AL | 1.112 | 0.51 | Low RR + Low P | No | 0.408 | 37050 |
| 1049 | DeKalb County, AL | 0.555 | 0.78 | Low RR + Low P | No | 0.407 | 144651 |
| 33007 | Coos County, NH | 1.248 | 0.29 | Low RR + Low P | No | 0.407 | 62848 |
| 39129 | Pickaway County, OH | 1.279 | 0.16 | Low RR + Low P | No | 0.407 | 121093 |
| 31175 | Valley County, NE | 1.025 | 0.61 | Low RR + Low P | No | 0.407 | 8070 |
| 27101 | Murray County, MN | 0.864 | 0.74 | Low RR + Low P | No | 0.406 | 16089 |
| 17103 | Lee County, IL | 1.207 | 0.34 | Low RR + Low P | No | 0.406 | 67592 |
| 32017 | Lincoln County, NV | 1.016 | 0.61 | Low RR + Low P | No | 0.406 | 8892 |
| 51063 | Floyd County, VA | 0.757 | 0.77 | Low RR + Low P | No | 0.406 | 31302 |
| 27065 | Kanabec County, MN | 1.14 | 0.49 | Low RR + Low P | No | 0.406 | 33051 |
| 13195 | Madison County, GA | 0.801 | 0.71 | Low RR + Low P | No | 0.405 | 63641 |
| 47173 | Union County, TN | 1.363 | 0.15 | Low RR + Low P | No | 0.405 | 41214 |
| 16049 | Idaho County, ID | 0.583 | 0.84 | Low RR + High P | No | 0.405 | 35527 |
| 30055 | McCone County, MT | 1.351 | 0.35 | Low RR + Low P | No | 0.405 | 3387 |
| 48161 | Freestone County, TX | 1.127 | 0.49 | Low RR + Low P | No | 0.405 | 40473 |
| 55111 | Sauk County, WI | 1.035 | 0.5 | Low RR + Low P | No | 0.405 | 131690 |
| 39095 | Lucas County, OH | 1.346 | 0 | Low RR + Low P | No | 0.404 | 852203 |
| 31079 | Hall County, NE | 1.275 | 0.16 | Low RR + Low P | No | 0.404 | 124293 |
| 51017 | Bath County, VA | 1.019 | 0.61 | Low RR + Low P | No | 0.404 | 8112 |
| 53043 | Lincoln County, WA | 1.099 | 0.54 | Low RR + Low P | No | 0.404 | 23351 |
| 1035 | Conecuh County, AL | 0.872 | 0.71 | Low RR + Low P | No | 0.404 | 22398 |
| 47059 | Greene County, TN | 1.171 | 0.32 | Low RR + Low P | No | 0.404 | 143869 |
| 48039 | Brazoria County, TX | 1.037 | 0.45 | Low RR + Low P | No | 0.403 | 787172 |
| 37099 | Jackson County, NC | 1.042 | 0.51 | Low RR + Low P | No | 0.403 | 88496 |
| 18169 | Wabash County, IN | 0.972 | 0.57 | Low RR + Low P | No | 0.403 | 61512 |
| 53075 | Whitman County, WA | 1.102 | 0.44 | Low RR + Low P | No | 0.402 | 95591 |
| 6107 | Tulare County, CA | 1.336 | 0 | Low RR + Low P | No | 0.402 | 957352 |
| 54075 | Pocahontas County, WV | 0.771 | 0.79 | Low RR + Low P | No | 0.402 | 15562 |
| 1119 | Sumter County, AL | 0.789 | 0.76 | Low RR + Low P | No | 0.402 | 23616 |
| 48017 | Bailey County, TX | 1.002 | 0.61 | Low RR + Low P | No | 0.402 | 13475 |
| 18155 | Switzerland County, IN | 0.895 | 0.69 | Low RR + Low P | No | 0.401 | 20028 |
| 12129 | Wakulla County, FL | 1.311 | 0.15 | Low RR + Low P | No | 0.401 | 71618 |
| 26081 | Kent County, MI | 1.261 | 0.04 | Low RR + Low P | No | 0.401 | 1320274 |
| 26045 | Eaton County, MI | 0.877 | 0.6 | Low RR + Low P | No | 0.401 | 217674 |
| 39057 | Greene County, OH | 1.073 | 0.42 | Low RR + Low P | No | 0.401 | 338718 |
| 50025 | Windham County, VT | 1.348 | 0.04 | Low RR + Low P | No | 0.401 | 91865 |
| 28009 | Benton County, MS | 1.186 | 0.47 | Low RR + Low P | No | 0.4 | 14994 |
| 22055 | Lafayette Parish, LA | 0.565 | 0.74 | Low RR + Low P | No | 0.4 | 497464 |
| 48247 | Jim Hogg County, TX | 1.221 | 0.45 | Low RR + Low P | No | 0.4 | 9494 |
| 26165 | Wexford County, MI | 1.139 | 0.42 | Low RR + Low P | No | 0.4 | 68140 |
| 20087 | Jefferson County, KS | 1.162 | 0.44 | Low RR + Low P | No | 0.4 | 36681 |
| 38103 | Wells County, ND | 0.949 | 0.67 | Low RR + Low P | No | 0.4 | 7798 |
| 47101 | Lewis County, TN | 0.835 | 0.72 | Low RR + Low P | No | 0.4 | 25978 |
| 26107 | Mecosta County, MI | 1.144 | 0.41 | Low RR + Low P | No | 0.4 | 81881 |
| 48297 | Live Oak County, TX | 0.881 | 0.7 | Low RR + Low P | No | 0.4 | 23125 |
| 32015 | Lander County, NV | 1.114 | 0.54 | Low RR + Low P | No | 0.399 | 11530 |
| 20115 | Marion County, KS | 0.787 | 0.76 | Low RR + Low P | No | 0.399 | 23473 |
| 29051 | Cole County, MO | 0.766 | 0.68 | Low RR + Low P | No | 0.399 | 154234 |
| 37041 | Chowan County, NC | 1.361 | 0.18 | Low RR + Low P | No | 0.399 | 27789 |
| 37045 | Cleveland County, NC | 1.261 | 0.12 | Low RR + Low P | No | 0.398 | 202031 |
| 51790 | Staunton city, VA | 1.077 | 0.5 | Low RR + Low P | No | 0.398 | 51855 |
| 30081 | Ravalli County, MT | 0.589 | 0.77 | Low RR + Low P | No | 0.398 | 94915 |
| 42109 | Snyder County, PA | 0.797 | 0.69 | Low RR + Low P | No | 0.398 | 79223 |
| 27111 | Otter Tail County, MN | 1.193 | 0.28 | Low RR + Low P | No | 0.398 | 121170 |
| 38029 | Emmons County, ND | 1.066 | 0.57 | Low RR + Low P | No | 0.398 | 6478 |
| 29129 | Mercer County, MO | 1.13 | 0.54 | Low RR + Low P | No | 0.398 | 6935 |
| 39137 | Putnam County, OH | 1.113 | 0.44 | Low RR + Low P | No | 0.398 | 68529 |
| 27121 | Pope County, MN | 1.29 | 0.31 | Low RR + Low P | No | 0.398 | 22820 |
| 41061 | Union County, OR | 0.977 | 0.57 | Low RR + Low P | No | 0.397 | 52109 |
| 17171 | Scott County, IL | 1.17 | 0.5 | Low RR + Low P | No | 0.397 | 9481 |
| 22039 | Evangeline Parish, LA | 0.636 | 0.78 | Low RR + Low P | No | 0.397 | 63787 |
| 56041 | Uinta County, WY | 1.141 | 0.45 | Low RR + Low P | No | 0.397 | 41472 |
| 1025 | Clarke County, AL | 0.743 | 0.75 | Low RR + Low P | No | 0.397 | 44911 |
| 53059 | Skamania County, WA | 0.832 | 0.72 | Low RR + Low P | No | 0.397 | 25095 |
| 19091 | Humboldt County, IA | 1.117 | 0.52 | Low RR + Low P | No | 0.396 | 19021 |
| 41005 | Clackamas County, OR | 1.1 | 0.35 | Low RR + Low P | No | 0.396 | 846291 |
| 12043 | Glades County, FL | 0.667 | 0.81 | Low RR + High P | No | 0.396 | 25249 |
| 48479 | Webb County, TX | 1.33 | 0 | Low RR + Low P | No | 0.396 | 536767 |
| 18143 | Scott County, IN | 0.88 | 0.64 | Low RR + Low P | No | 0.396 | 49157 |
| 29007 | Audrain County, MO | 1.262 | 0.26 | Low RR + Low P | No | 0.396 | 48876 |
| 48141 | El Paso County, TX | 1.274 | 0.01 | Low RR + Low P | No | 0.396 | 1737119 |
| 26035 | Clare County, MI | 1.206 | 0.3 | Low RR + Low P | No | 0.395 | 62623 |
| 47005 | Benton County, TN | 1.155 | 0.45 | Low RR + Low P | No | 0.395 | 32097 |
| 38021 | Dickey County, ND | 0.958 | 0.64 | Low RR + Low P | No | 0.395 | 9828 |
| 42043 | Dauphin County, PA | 1.097 | 0.36 | Low RR + Low P | No | 0.395 | 577921 |
| 19171 | Tama County, IA | 1.27 | 0.29 | Low RR + Low P | No | 0.395 | 33776 |
| 29165 | Platte County, MO | 0.825 | 0.62 | Low RR + Low P | No | 0.395 | 222510 |
| 19037 | Chickasaw County, IA | 0.813 | 0.74 | Low RR + Low P | No | 0.395 | 23386 |
| 48341 | Moore County, TX | 1.172 | 0.41 | Low RR + Low P | No | 0.395 | 42284 |
| 6115 | Yuba County, CA | 1.338 | 0 | Low RR + Low P | No | 0.394 | 170075 |
| 56007 | Carbon County, WY | 0.782 | 0.75 | Low RR + Low P | No | 0.394 | 28872 |
| 2185 | North Slope Borough, AK | 0.808 | 0.74 | Low RR + Low P | No | 0.394 | 21414 |
| 39131 | Pike County, OH | 0.968 | 0.57 | Low RR + Low P | No | 0.394 | 54013 |
| 35033 | Mora County, NM | 0.948 | 0.66 | Low RR + Low P | No | 0.394 | 8270 |
| 46101 | Moody County, SD | 1.166 | 0.48 | Low RR + Low P | No | 0.394 | 12832 |
| 13101 | Echols County, GA | 1.019 | 0.59 | Low RR + Low P | No | 0.393 | 7395 |
| 46127 | Union County, SD | 1.054 | 0.53 | Low RR + Low P | No | 0.393 | 34246 |
| 48143 | Erath County, TX | 1.16 | 0.35 | Low RR + Low P | No | 0.393 | 88032 |
| 17145 | Perry County, IL | 0.752 | 0.74 | Low RR + Low P | No | 0.393 | 41015 |
| 23027 | Waldo County, ME | 0.97 | 0.55 | Low RR + Low P | No | 0.393 | 80875 |
| 32029 | Storey County, NV | 0.807 | 0.77 | Low RR + Low P | No | 0.393 | 8342 |
| 20185 | Stafford County, KS | 1.052 | 0.57 | Low RR + Low P | No | 0.393 | 7880 |
| 29201 | Scott County, MO | 0.816 | 0.66 | Low RR + Low P | No | 0.392 | 75757 |
| 17067 | Hancock County, IL | 0.888 | 0.65 | Low RR + Low P | No | 0.392 | 34440 |
| 48199 | Hardin County, TX | 1.231 | 0.17 | Low RR + Low P | No | 0.391 | 116101 |
| 26067 | Ionia County, MI | 0.968 | 0.53 | Low RR + Low P | No | 0.391 | 133098 |
| 33003 | Carroll County, NH | 0.787 | 0.66 | Low RR + Low P | No | 0.391 | 104602 |
| 55023 | Crawford County, WI | 0.896 | 0.64 | Low RR + Low P | No | 0.391 | 31959 |
| 8037 | Eagle County, CO | 0.397 | 0.8 | Low RR + Low P | No | 0.391 | 109599 |
| 34033 | Salem County, NJ | 0.862 | 0.6 | Low RR + Low P | No | 0.391 | 130495 |
| 21153 | Magoffin County, KY | 1.162 | 0.45 | Low RR + Low P | No | 0.391 | 22576 |
| 35007 | Colfax County, NM | 1.044 | 0.55 | Low RR + Low P | No | 0.391 | 24527 |
| 29213 | Taney County, MO | 0.492 | 0.78 | Low RR + Low P | No | 0.391 | 113524 |
| 1105 | Perry County, AL | 0.772 | 0.77 | Low RR + Low P | No | 0.391 | 15642 |
| 51029 | Buckingham County, VA | 0.761 | 0.74 | Low RR + Low P | No | 0.39 | 33966 |
| 13241 | Rabun County, GA | 0.563 | 0.82 | Low RR + High P | No | 0.39 | 34709 |
| 38019 | Cavalier County, ND | 0.972 | 0.62 | Low RR + Low P | No | 0.39 | 7204 |
| 39161 | Van Wert County, OH | 1.221 | 0.27 | Low RR + Low P | No | 0.39 | 57508 |
| 6095 | Solano County, CA | 1.256 | 0.02 | Low RR + Low P | No | 0.39 | 898103 |
| 48357 | Ochiltree County, TX | 0.951 | 0.62 | Low RR + Low P | No | 0.39 | 19396 |
| 41001 | Baker County, OR | 1.221 | 0.33 | Low RR + Low P | No | 0.39 | 33849 |
| 38097 | Traill County, ND | 0.816 | 0.74 | Low RR + Low P | No | 0.39 | 15863 |
| 5103 | Ouachita County, AR | 0.566 | 0.8 | Low RR + High P | No | 0.389 | 43787 |
| 21171 | Monroe County, KY | 1.279 | 0.3 | Low RR + Low P | No | 0.389 | 22659 |
| 4009 | Graham County, AZ | 1.043 | 0.49 | Low RR + Low P | No | 0.389 | 78347 |
| 30083 | Richland County, MT | 1.18 | 0.42 | Low RR + Low P | No | 0.389 | 22403 |
| 46039 | Deuel County, SD | 1.074 | 0.56 | Low RR + Low P | No | 0.389 | 8715 |
| 37015 | Bertie County, NC | 1.333 | 0.15 | Low RR + Low P | No | 0.389 | 33990 |
| 8055 | Huerfano County, CO | 0.951 | 0.63 | Low RR + Low P | No | 0.388 | 14152 |
| 35027 | Lincoln County, NM | 0.488 | 0.83 | Low RR + High P | No | 0.388 | 40372 |
| 16065 | Madison County, ID | 1.078 | 0.42 | Low RR + Low P | No | 0.388 | 109505 |
| 36109 | Tompkins County, NY | 0.683 | 0.69 | Low RR + Low P | No | 0.388 | 207857 |
| 15001 | Hawaii County, HI | 0.9 | 0.55 | Low RR + Low P | No | 0.388 | 413963 |
| 26051 | Gladwin County, MI | 0.868 | 0.63 | Low RR + Low P | No | 0.387 | 51474 |
| 40003 | Alfalfa County, OK | 1.261 | 0.37 | Low RR + Low P | No | 0.387 | 11362 |
| 28159 | Winston County, MS | 0.965 | 0.58 | Low RR + Low P | No | 0.387 | 34920 |
| 28131 | Stone County, MS | 0.97 | 0.57 | Low RR + Low P | No | 0.387 | 37397 |
| 29069 | Dunklin County, MO | 1.276 | 0.17 | Low RR + Low P | No | 0.387 | 54443 |
| 26043 | Dickinson County, MI | 1.182 | 0.34 | Low RR + Low P | No | 0.386 | 51925 |
| 20055 | Finney County, KS | 0.712 | 0.71 | Low RR + Low P | No | 0.386 | 75105 |
| 12057 | Hillsborough County, FL | 0.962 | 0.48 | Low RR + Low P | No | 0.386 | 3049037 |
| 18159 | Tipton County, IN | 1.041 | 0.53 | Low RR + Low P | No | 0.386 | 30581 |
| 51103 | Lancaster County, VA | 0.641 | 0.81 | Low RR + High P | No | 0.386 | 21665 |
| 18133 | Putnam County, IN | 1.155 | 0.35 | Low RR + Low P | No | 0.385 | 74861 |
| 48305 | Lynn County, TX | 1.042 | 0.56 | Low RR + Low P | No | 0.385 | 11499 |
| 21189 | Owsley County, KY | 1.218 | 0.42 | Low RR + Low P | No | 0.385 | 7959 |
| 50001 | Addison County, VT | 1.282 | 0.11 | Low RR + Low P | No | 0.385 | 75275 |
| 20141 | Osborne County, KS | 1.053 | 0.56 | Low RR + Low P | No | 0.385 | 6908 |
| 53049 | Pacific County, WA | 1.182 | 0.34 | Low RR + Low P | No | 0.385 | 48295 |
| 48501 | Yoakum County, TX | 1.033 | 0.56 | Low RR + Low P | No | 0.383 | 14952 |
| 16031 | Cassia County, ID | 1.007 | 0.53 | Low RR + Low P | No | 0.383 | 51340 |
| 6027 | Inyo County, CA | 0.842 | 0.66 | Low RR + Low P | No | 0.383 | 37269 |
| 39055 | Geauga County, OH | 0.664 | 0.7 | Low RR + Low P | No | 0.382 | 190898 |
| 17091 | Kankakee County, IL | 1.163 | 0.22 | Low RR + Low P | No | 0.382 | 211938 |
| 36107 | Tioga County, NY | 1.146 | 0.32 | Low RR + Low P | No | 0.382 | 95481 |
| 39125 | Paulding County, OH | 1.339 | 0.09 | Low RR + Low P | No | 0.382 | 37468 |
| 27105 | Nobles County, MN | 0.98 | 0.55 | Low RR + Low P | No | 0.382 | 43685 |
| 6077 | San Joaquin County, CA | 1.274 | 0 | Low RR + Low P | No | 0.382 | 1595258 |
| 53035 | Kitsap County, WA | 0.925 | 0.52 | Low RR + Low P | No | 0.382 | 555471 |
| 19177 | Van Buren County, IA | 0.918 | 0.64 | Low RR + Low P | No | 0.381 | 14518 |
| 35029 | Luna County, NM | 0.88 | 0.61 | Low RR + Low P | No | 0.381 | 51009 |
| 19157 | Poweshiek County, IA | 0.892 | 0.62 | Low RR + Low P | No | 0.381 | 36971 |
| 54007 | Braxton County, WV | 1.177 | 0.41 | Low RR + Low P | No | 0.381 | 24332 |
| 24029 | Kent County, MD | 1.179 | 0.36 | Low RR + Low P | No | 0.381 | 38598 |
| 46087 | McCook County, SD | 0.978 | 0.6 | Low RR + Low P | No | 0.381 | 11577 |
| 30001 | Beaverhead County, MT | 0.87 | 0.67 | Low RR + Low P | No | 0.381 | 19632 |
| 51193 | Westmoreland County, VA | 0.766 | 0.71 | Low RR + Low P | No | 0.381 | 37778 |
| 13049 | Charlton County, GA | 0.729 | 0.76 | Low RR + Low P | No | 0.38 | 25722 |
| 38069 | Pierce County, ND | 1.035 | 0.57 | Low RR + Low P | No | 0.38 | 7833 |
| 48505 | Zapata County, TX | 1.138 | 0.44 | Low RR + Low P | No | 0.38 | 27574 |
| 39147 | Seneca County, OH | 1.093 | 0.39 | Low RR + Low P | No | 0.38 | 109061 |
| 54049 | Marion County, WV | 1.098 | 0.38 | Low RR + Low P | No | 0.379 | 111591 |
| 39173 | Wood County, OH | 1.226 | 0.04 | Low RR + Low P | No | 0.379 | 264292 |
| 5069 | Jefferson County, AR | 1.138 | 0.3 | Low RR + Low P | No | 0.379 | 127956 |
| 53011 | Clark County, WA | 1.173 | 0.11 | Low RR + Low P | No | 0.379 | 1037887 |
| 22111 | Union Parish, LA | 0.694 | 0.74 | Low RR + Low P | No | 0.379 | 41379 |
| 28119 | Quitman County, MS | 0.967 | 0.6 | Low RR + Low P | No | 0.378 | 11220 |
| 49001 | Beaver County, UT | 0.856 | 0.7 | Low RR + Low P | No | 0.378 | 14550 |
| 18007 | Benton County, IN | 1.247 | 0.32 | Low RR + Low P | No | 0.378 | 17401 |
| 51163 | Rockbridge County, VA | 0.684 | 0.74 | Low RR + Low P | No | 0.378 | 44926 |
| 16067 | Minidoka County, ID | 1.004 | 0.53 | Low RR + Low P | No | 0.378 | 44688 |
| 13037 | Calhoun County, GA | 0.994 | 0.58 | Low RR + Low P | No | 0.378 | 10931 |
| 21203 | Rockcastle County, KY | 0.941 | 0.59 | Low RR + Low P | No | 0.378 | 32415 |
| 48397 | Rockwall County, TX | 0.942 | 0.52 | Low RR + Low P | No | 0.377 | 254649 |
| 38105 | Williams County, ND | 0.889 | 0.59 | Low RR + Low P | No | 0.377 | 77079 |
| 29173 | Ralls County, MO | 0.727 | 0.76 | Low RR + Low P | No | 0.377 | 20899 |
| 48445 | Terry County, TX | 0.86 | 0.66 | Low RR + Low P | No | 0.377 | 22991 |
| 31093 | Howard County, NE | 0.952 | 0.61 | Low RR + Low P | No | 0.377 | 13036 |
| 51683 | Manassas city, VA | 0.744 | 0.67 | Low RR + Low P | No | 0.377 | 85369 |
| 29135 | Moniteau County, MO | 0.671 | 0.77 | Low RR + Low P | No | 0.377 | 30516 |
| 12037 | Franklin County, FL | 0.775 | 0.72 | Low RR + Low P | No | 0.377 | 25086 |
| 39091 | Logan County, OH | 1.288 | 0.03 | Low RR + Low P | No | 0.377 | 92119 |
| 39099 | Mahoning County, OH | 0.993 | 0.44 | Low RR + Low P | No | 0.376 | 451518 |
| 53031 | Jefferson County, WA | 0.787 | 0.66 | Low RR + Low P | No | 0.376 | 67284 |
| 2180 | Nome Census Area, AK | 0.999 | 0.56 | Low RR + Low P | No | 0.376 | 19554 |
| 54031 | Hardy County, WV | 1.129 | 0.44 | Low RR + Low P | No | 0.376 | 28419 |
| 5147 | Woodruff County, AR | 1.123 | 0.49 | Low RR + Low P | No | 0.375 | 12021 |
| 21113 | Jessamine County, KY | 1.191 | 0.17 | Low RR + Low P | No | 0.375 | 109253 |
| 6067 | Sacramento County, CA | 1.252 | 0 | Low RR + Low P | No | 0.375 | 3168668 |
| 18147 | Spencer County, IN | 0.953 | 0.56 | Low RR + Low P | No | 0.375 | 39759 |
| 6065 | Riverside County, CA | 1.247 | 0 | Low RR + Low P | No | 0.374 | 4966683 |
| 46045 | Edmunds County, SD | 1.083 | 0.53 | Low RR + Low P | No | 0.374 | 8128 |
| 48139 | Ellis County, TX | 1.074 | 0.31 | Low RR + Low P | No | 0.374 | 435152 |
| 21039 | Carlisle County, KY | 1.063 | 0.54 | Low RR + Low P | No | 0.374 | 9423 |
| 29229 | Wright County, MO | 1.252 | 0.2 | Low RR + Low P | No | 0.374 | 38546 |
| 8047 | Gilpin County, CO | 0.985 | 0.58 | Low RR + Low P | No | 0.374 | 11812 |
| 48035 | Bosque County, TX | 1.256 | 0.2 | Low RR + Low P | No | 0.373 | 37703 |
| 47167 | Tipton County, TN | 1.085 | 0.37 | Low RR + Low P | No | 0.373 | 123665 |
| 39149 | Shelby County, OH | 1.158 | 0.26 | Low RR + Low P | No | 0.373 | 95449 |
| 6113 | Yolo County, CA | 1.246 | 0 | Low RR + Low P | No | 0.373 | 442562 |
| 47135 | Perry County, TN | 1.399 | 0.04 | Low RR + Low P | No | 0.373 | 17596 |
| 48281 | Lampasas County, TX | 1.194 | 0.27 | Low RR + Low P | No | 0.373 | 46052 |
| 38033 | Golden Valley County, ND | 1.205 | 0.42 | Low RR + Low P | No | 0.373 | 3489 |
| 55015 | Calumet County, WI | 1.254 | 0.05 | Low RR + Low P | No | 0.372 | 105911 |
| 42103 | Pike County, PA | 0.766 | 0.63 | Low RR + Low P | No | 0.372 | 121823 |
| 45057 | Lancaster County, SC | 1.149 | 0.21 | Low RR + Low P | No | 0.372 | 212939 |
| 21069 | Fleming County, KY | 1.304 | 0.14 | Low RR + Low P | No | 0.372 | 30720 |
| 22003 | Allen Parish, LA | 0.528 | 0.78 | Low RR + Low P | No | 0.371 | 44399 |
| 51175 | Southampton County, VA | 0.847 | 0.64 | Low RR + Low P | No | 0.371 | 35874 |
| 6031 | Kings County, CA | 1.222 | 0.02 | Low RR + Low P | No | 0.37 | 305669 |
| 42011 | Berks County, PA | 0.953 | 0.46 | Low RR + Low P | No | 0.37 | 864394 |
| 35006 | Cibola County, NM | 1.16 | 0.31 | Low RR + Low P | No | 0.37 | 53651 |
| 46135 | Yankton County, SD | 1.164 | 0.31 | Low RR + Low P | No | 0.37 | 46929 |
| 17059 | Gallatin County, IL | 0.941 | 0.61 | Low RR + Low P | No | 0.369 | 9493 |
| 51093 | Isle of Wight County, VA | 1.177 | 0.22 | Low RR + Low P | No | 0.369 | 80846 |
| 5065 | Izard County, AR | 0.655 | 0.76 | Low RR + Low P | No | 0.369 | 28198 |
| 48197 | Hardeman County, TX | 1.199 | 0.4 | Low RR + Low P | No | 0.369 | 7015 |
| 41051 | Multnomah County, OR | 1.1 | 0.22 | Low RR + Low P | No | 0.369 | 1584261 |
| 53013 | Columbia County, WA | 1.187 | 0.41 | Low RR + Low P | No | 0.368 | 8079 |
| 22123 | West Carroll Parish, LA | 0.839 | 0.68 | Low RR + Low P | No | 0.368 | 18804 |
| 48159 | Franklin County, TX | 1.065 | 0.5 | Low RR + Low P | No | 0.368 | 21400 |
| 51053 | Dinwiddie County, VA | 0.579 | 0.75 | Low RR + Low P | No | 0.368 | 56442 |
| 54067 | Nicholas County, WV | 1.224 | 0.18 | Low RR + Low P | No | 0.368 | 48478 |
| 47121 | Meigs County, TN | 0.936 | 0.59 | Low RR + Low P | No | 0.368 | 26949 |
| 46029 | Codington County, SD | 1.254 | 0.11 | Low RR + Low P | No | 0.368 | 57707 |
| 28003 | Alcorn County, MS | 0.704 | 0.69 | Low RR + Low P | No | 0.368 | 68282 |
| 15009 | Maui County, HI | 1.012 | 0.4 | Low RR + Low P | No | 0.368 | 328542 |
| 20147 | Phillips County, KS | 0.897 | 0.65 | Low RR + Low P | No | 0.367 | 9544 |
| 45043 | Georgetown County, SC | 1.149 | 0.23 | Low RR + Low P | No | 0.367 | 130400 |
| 22083 | Richland Parish, LA | 0.524 | 0.78 | Low RR + Low P | No | 0.367 | 39496 |
| 4012 | La Paz County, AZ | 1.144 | 0.39 | Low RR + Low P | No | 0.367 | 33245 |
| 13289 | Twiggs County, GA | 0.835 | 0.69 | Low RR + Low P | No | 0.367 | 15367 |
| 30071 | Phillips County, MT | 1.026 | 0.56 | Low RR + Low P | No | 0.367 | 8477 |
| 49015 | Emery County, UT | 1.045 | 0.52 | Low RR + Low P | No | 0.367 | 20237 |
| 54101 | Webster County, WV | 1.255 | 0.28 | Low RR + Low P | No | 0.366 | 16186 |
| 13065 | Clinch County, GA | 0.987 | 0.57 | Low RR + Low P | No | 0.366 | 13425 |
| 47047 | Fayette County, TN | 0.937 | 0.53 | Low RR + Low P | No | 0.366 | 87513 |
| 51153 | Prince William County, VA | 0.499 | 0.69 | Low RR + Low P | No | 0.366 | 976652 |
| 29217 | Vernon County, MO | 1.054 | 0.47 | Low RR + Low P | No | 0.365 | 39309 |
| 35023 | Hidalgo County, NM | 1.068 | 0.52 | Low RR + Low P | No | 0.364 | 7976 |
| 26087 | Lapeer County, MI | 0.883 | 0.55 | Low RR + Low P | No | 0.363 | 177609 |
| 39021 | Champaign County, OH | 1.18 | 0.18 | Low RR + Low P | No | 0.363 | 77588 |
| 39039 | Defiance County, OH | 1.23 | 0.07 | Low RR + Low P | No | 0.362 | 76443 |
| 31043 | Dakota County, NE | 1.076 | 0.42 | Low RR + Low P | No | 0.362 | 42384 |
| 20191 | Sumner County, KS | 0.489 | 0.77 | Low RR + Low P | No | 0.362 | 44693 |
| 26001 | Alcona County, MI | 1.349 | 0.04 | Low RR + Low P | No | 0.361 | 20886 |
| 13087 | Decatur County, GA | 0.928 | 0.55 | Low RR + Low P | No | 0.361 | 58106 |
| 46075 | Jones County, SD | 1.07 | 0.52 | Low RR + Low P | No | 0.361 | 1730 |
| 13251 | Screven County, GA | 1.245 | 0.19 | Low RR + Low P | No | 0.36 | 28144 |
| 37033 | Caswell County, NC | 0.815 | 0.63 | Low RR + Low P | No | 0.36 | 45238 |
| 50023 | Washington County, VT | 1.24 | 0.02 | Low RR + Low P | No | 0.359 | 120257 |
| 8011 | Bent County, CO | 0.953 | 0.59 | Low RR + Low P | No | 0.359 | 11120 |
| 48171 | Gillespie County, TX | 0.996 | 0.49 | Low RR + Low P | No | 0.359 | 55234 |
| 51057 | Essex County, VA | 0.745 | 0.71 | Low RR + Low P | No | 0.358 | 21228 |
| 29063 | DeKalb County, MO | 0.732 | 0.72 | Low RR + Low P | No | 0.358 | 21209 |
| 37167 | Stanly County, NC | 1.178 | 0.11 | Low RR + Low P | No | 0.358 | 129930 |
| 5013 | Calhoun County, AR | 0.947 | 0.59 | Low RR + Low P | No | 0.358 | 9328 |
| 56033 | Sheridan County, WY | 0.654 | 0.7 | Low RR + Low P | No | 0.358 | 64554 |
| 27153 | Todd County, MN | 0.911 | 0.56 | Low RR + Low P | No | 0.358 | 51200 |
| 51079 | Greene County, VA | 0.586 | 0.75 | Low RR + Low P | No | 0.358 | 42316 |
| 21239 | Woodford County, KY | 1.212 | 0.14 | Low RR + Low P | No | 0.357 | 54321 |
| 31051 | Dixon County, NE | 0.93 | 0.6 | Low RR + Low P | No | 0.357 | 11007 |
| 51181 | Surry County, VA | 1.262 | 0.24 | Low RR + Low P | No | 0.357 | 13120 |
| 42035 | Clinton County, PA | 1.166 | 0.19 | Low RR + Low P | No | 0.357 | 75383 |
| 30089 | Sanders County, MT | 0.92 | 0.58 | Low RR + Low P | No | 0.357 | 27068 |
| 54023 | Grant County, WV | 1.218 | 0.25 | Low RR + Low P | No | 0.357 | 21859 |
| 37095 | Hyde County, NC | 1.168 | 0.4 | Low RR + Low P | No | 0.357 | 9263 |
| 47175 | Van Buren County, TN | 0.89 | 0.62 | Low RR + Low P | No | 0.357 | 12923 |
| 46009 | Bon Homme County, SD | 1.054 | 0.51 | Low RR + Low P | No | 0.357 | 14129 |
| 48069 | Castro County, TX | 0.875 | 0.63 | Low RR + Low P | No | 0.356 | 14514 |
| 36123 | Yates County, NY | 1.009 | 0.48 | Low RR + Low P | No | 0.355 | 48974 |
| 39171 | Williams County, OH | 1.205 | 0.1 | Low RR + Low P | No | 0.355 | 73239 |
| 47153 | Sequatchie County, TN | 1.091 | 0.42 | Low RR + Low P | No | 0.355 | 34079 |
| 51099 | King George County, VA | 0.439 | 0.76 | Low RR + Low P | No | 0.354 | 56486 |
| 54089 | Summers County, WV | 1.049 | 0.48 | Low RR + Low P | No | 0.353 | 23283 |
| 48271 | Kinney County, TX | 1.12 | 0.46 | Low RR + Low P | No | 0.353 | 6279 |
| 26137 | Otsego County, MI | 0.955 | 0.52 | Low RR + Low P | No | 0.353 | 51308 |
| 26151 | Sanilac County, MI | 1.228 | 0.03 | Low RR + Low P | No | 0.353 | 80850 |
| 1083 | Limestone County, AL | 1.138 | 0.14 | Low RR + Low P | No | 0.352 | 225522 |
| 48321 | Matagorda County, TX | 0.952 | 0.5 | Low RR + Low P | No | 0.352 | 72481 |
| 48093 | Comanche County, TX | 0.652 | 0.74 | Low RR + Low P | No | 0.352 | 27971 |
| 29197 | Schuyler County, MO | 1.099 | 0.47 | Low RR + Low P | No | 0.352 | 8053 |
| 42037 | Columbia County, PA | 0.991 | 0.42 | Low RR + Low P | No | 0.352 | 130728 |
| 36009 | Cattaraugus County, NY | 1.134 | 0.17 | Low RR + Low P | No | 0.352 | 151650 |
| 19179 | Wapello County, IA | 1.228 | 0.04 | Low RR + Low P | No | 0.352 | 70247 |
| 46091 | Marshall County, SD | 1.039 | 0.52 | Low RR + Low P | No | 0.351 | 8753 |
| 29017 | Bollinger County, MO | 0.641 | 0.75 | Low RR + Low P | No | 0.351 | 21075 |
| 1023 | Choctaw County, AL | 1.065 | 0.45 | Low RR + Low P | No | 0.351 | 24683 |
| 18087 | Lagrange County, IN | 0.972 | 0.47 | Low RR + Low P | No | 0.351 | 81761 |
| 1059 | Franklin County, AL | 0.655 | 0.69 | Low RR + Low P | No | 0.35 | 63712 |
| 39143 | Sandusky County, OH | 1.167 | 0.11 | Low RR + Low P | No | 0.35 | 117276 |
| 8079 | Mineral County, CO | 0.915 | 0.62 | Low RR + Low P | No | 0.35 | 1875 |
| 38035 | Grand Forks County, ND | 0.95 | 0.46 | Low RR + Low P | No | 0.35 | 145129 |
| 6055 | Napa County, CA | 1.187 | 0.01 | Low RR + Low P | No | 0.35 | 267586 |
| 19047 | Crawford County, IA | 0.766 | 0.66 | Low RR + Low P | No | 0.35 | 32142 |
| 31155 | Saunders County, NE | 1.195 | 0.16 | Low RR + Low P | No | 0.35 | 46640 |
| 17051 | Fayette County, IL | 0.707 | 0.68 | Low RR + Low P | No | 0.349 | 42434 |
| 16029 | Caribou County, ID | 1.012 | 0.52 | Low RR + Low P | No | 0.349 | 14419 |
| 55013 | Burnett County, WI | 0.98 | 0.51 | Low RR + Low P | No | 0.349 | 34080 |
| 42081 | Lycoming County, PA | 1.152 | 0.07 | Low RR + Low P | No | 0.349 | 225758 |
| 17099 | LaSalle County, IL | 1.173 | 0.04 | Low RR + Low P | No | 0.349 | 216490 |
| 31003 | Antelope County, NE | 0.976 | 0.55 | Low RR + Low P | No | 0.349 | 12607 |
| 31035 | Clay County, NE | 0.988 | 0.55 | Low RR + Low P | No | 0.348 | 12165 |
| 18033 | De Kalb County, IN | 1.061 | 0.34 | Low RR + Low P | No | 0.348 | 88011 |
| 30013 | Cascade County, MT | 1.033 | 0.34 | Low RR + Low P | No | 0.348 | 169901 |
| 17023 | Clark County, IL | 0.945 | 0.55 | Low RR + Low P | No | 0.348 | 30248 |
| 8015 | Chaffee County, CO | 0.531 | 0.75 | Low RR + Low P | No | 0.348 | 40882 |
| 21055 | Crittenden County, KY | 1.242 | 0.19 | Low RR + Low P | No | 0.348 | 17965 |
| 51067 | Franklin County, VA | 1.085 | 0.28 | Low RR + Low P | No | 0.348 | 110628 |
| 1039 | Covington County, AL | 1.02 | 0.41 | Low RR + Low P | No | 0.348 | 75555 |
| 47127 | Moore County, TN | 1.014 | 0.52 | Low RR + Low P | No | 0.348 | 13464 |
| 13025 | Brantley County, GA | 0.653 | 0.71 | Low RR + Low P | No | 0.347 | 36611 |
| 21157 | Marshall County, KY | 1 | 0.44 | Low RR + Low P | No | 0.347 | 63534 |
| 20039 | Decatur County, KS | 1.009 | 0.54 | Low RR + Low P | No | 0.347 | 5401 |
| 13315 | Wilcox County, GA | 0.81 | 0.66 | Low RR + Low P | No | 0.347 | 17530 |
| 51027 | Buchanan County, VA | 1.222 | 0.12 | Low RR + Low P | No | 0.347 | 38470 |
| 47011 | Bradley County, TN | 0.885 | 0.51 | Low RR + Low P | No | 0.347 | 222118 |
| 13291 | Union County, GA | 0.371 | 0.76 | Low RR + Low P | No | 0.347 | 53514 |
| 37139 | Pasquotank County, NC | 1.163 | 0.14 | Low RR + Low P | No | 0.346 | 82612 |
| 20009 | Barton County, KS | 0.915 | 0.54 | Low RR + Low P | No | 0.346 | 49957 |
| 53061 | Snohomish County, WA | 1.001 | 0.3 | Low RR + Low P | No | 0.346 | 1685213 |
| 16059 | Lemhi County, ID | 0.683 | 0.73 | Low RR + Low P | No | 0.346 | 16725 |
| 38101 | Ward County, ND | 0.895 | 0.51 | Low RR + Low P | No | 0.346 | 137120 |
| 48335 | Mitchell County, TX | 1.151 | 0.36 | Low RR + Low P | No | 0.346 | 18013 |
| 47169 | Trousdale County, TN | 1.162 | 0.3 | Low RR + Low P | No | 0.345 | 24348 |
| 29209 | Stone County, MO | 0.579 | 0.7 | Low RR + Low P | No | 0.345 | 64792 |
| 39011 | Auglaize County, OH | 1.142 | 0.17 | Low RR + Low P | No | 0.345 | 91996 |
| 26011 | Arenac County, MI | 1.127 | 0.34 | Low RR + Low P | No | 0.345 | 30268 |
| 39107 | Mercer County, OH | 1.23 | 0.01 | Low RR + Low P | No | 0.344 | 84863 |
| 5021 | Clay County, AR | 0.892 | 0.58 | Low RR + Low P | No | 0.344 | 28472 |
| 30067 | Park County, MT | 0.534 | 0.75 | Low RR + Low P | No | 0.344 | 35656 |
| 19011 | Benton County, IA | 0.975 | 0.48 | Low RR + Low P | No | 0.343 | 51538 |
| 30073 | Pondera County, MT | 0.841 | 0.64 | Low RR + Low P | No | 0.343 | 12217 |
| 5097 | Montgomery County, AR | 0.654 | 0.74 | Low RR + Low P | No | 0.343 | 17163 |
| 53051 | Pend Oreille County, WA | 0.93 | 0.55 | Low RR + Low P | No | 0.343 | 28499 |
| 26009 | Antrim County, MI | 0.971 | 0.48 | Low RR + Low P | No | 0.343 | 48674 |
| 26057 | Gratiot County, MI | 1.162 | 0.12 | Low RR + Low P | No | 0.343 | 82472 |
| 30097 | Sweet Grass County, MT | 1.024 | 0.52 | Low RR + Low P | No | 0.343 | 7490 |
| 31135 | Perkins County, NE | 0.851 | 0.65 | Low RR + Low P | No | 0.342 | 5622 |
| 8125 | Yuma County, CO | 0.657 | 0.73 | Low RR + Low P | No | 0.342 | 19757 |
| 55121 | Trempealeau County, WI | 1.053 | 0.36 | Low RR + Low P | No | 0.342 | 61790 |
| 51760 | Richmond city, VA | 0.695 | 0.59 | Low RR + Low P | No | 0.342 | 457614 |
| 46077 | Kingsbury County, SD | 0.905 | 0.59 | Low RR + Low P | No | 0.341 | 10555 |
| 18039 | Elkhart County, IN | 1.113 | 0.09 | Low RR + Low P | No | 0.341 | 413282 |
| 17143 | Peoria County, IL | 1.134 | 0.06 | Low RR + Low P | No | 0.341 | 355513 |
| 26085 | Lake County, MI | 1.191 | 0.21 | Low RR + Low P | No | 0.341 | 25385 |
| 42121 | Venango County, PA | 1.066 | 0.28 | Low RR + Low P | No | 0.341 | 99165 |
| 38053 | McKenzie County, ND | 0.819 | 0.62 | Low RR + Low P | No | 0.34 | 28141 |
| 22011 | Beauregard Parish, LA | 0.378 | 0.72 | Low RR + Low P | No | 0.34 | 73451 |
| 29199 | Scotland County, MO | 0.968 | 0.55 | Low RR + Low P | No | 0.34 | 9323 |
| 37077 | Granville County, NC | 0.986 | 0.4 | Low RR + Low P | No | 0.34 | 123894 |
| 5027 | Columbia County, AR | 0.928 | 0.53 | Low RR + Low P | No | 0.339 | 44436 |
| 48279 | Lamb County, TX | 1.199 | 0.18 | Low RR + Low P | No | 0.339 | 25497 |
| 5015 | Carroll County, AR | 0.434 | 0.73 | Low RR + Low P | No | 0.339 | 57531 |
| 28007 | Attala County, MS | 0.828 | 0.6 | Low RR + Low P | No | 0.339 | 34889 |
| 18095 | Madison County, IN | 1.004 | 0.32 | Low RR + Low P | No | 0.338 | 263887 |
| 51105 | Lee County, VA | 0.929 | 0.52 | Low RR + Low P | No | 0.338 | 43592 |
| 48319 | Mason County, TX | 0.96 | 0.56 | Low RR + Low P | No | 0.338 | 7909 |
| 47177 | Warren County, TN | 1.033 | 0.35 | Low RR + Low P | No | 0.338 | 84639 |
| 12003 | Baker County, FL | 0.83 | 0.58 | Low RR + Low P | No | 0.338 | 56154 |
| 31157 | Scotts Bluff County, NE | 0.951 | 0.47 | Low RR + Low P | No | 0.337 | 71468 |
| 53003 | Asotin County, WA | 1.143 | 0.23 | Low RR + Low P | No | 0.337 | 45085 |
| 6101 | Sutter County, CA | 1.175 | 0.01 | Low RR + Low P | No | 0.337 | 196517 |
| 5107 | Phillips County, AR | 1.175 | 0.21 | Low RR + Low P | No | 0.337 | 30311 |
| 48439 | Tarrant County, TX | 1.156 | 0 | Low RR + Low P | No | 0.336 | 4338593 |
| 37005 | Alleghany County, NC | 0.761 | 0.66 | Low RR + Low P | No | 0.335 | 22535 |
| 17057 | Fulton County, IL | 1.032 | 0.37 | Low RR + Low P | No | 0.335 | 65476 |
| 55059 | Kenosha County, WI | 1.1 | 0.11 | Low RR + Low P | No | 0.335 | 335125 |
| 38045 | LaMoure County, ND | 0.904 | 0.59 | Low RR + Low P | No | 0.335 | 8172 |
| 54037 | Jefferson County, WV | 0.861 | 0.52 | Low RR + Low P | No | 0.335 | 118788 |
| 22045 | Iberia Parish, LA | 1.117 | 0.13 | Low RR + Low P | No | 0.335 | 135979 |
| 16071 | Oneida County, ID | 1.03 | 0.49 | Low RR + Low P | No | 0.334 | 9693 |
| 13069 | Coffee County, GA | 0.647 | 0.64 | Low RR + Low P | No | 0.334 | 86564 |
| 38057 | Mercer County, ND | 0.795 | 0.65 | Low RR + Low P | No | 0.334 | 16636 |
| 39043 | Erie County, OH | 1.071 | 0.21 | Low RR + Low P | No | 0.334 | 148549 |
| 44005 | Newport County, RI | 0.652 | 0.61 | Low RR + Low P | No | 0.334 | 168553 |
| 26025 | Calhoun County, MI | 1.131 | 0.04 | Low RR + Low P | No | 0.334 | 266790 |
| 30039 | Granite County, MT | 0.959 | 0.55 | Low RR + Low P | No | 0.334 | 7092 |
| 27079 | Le Sueur County, MN | 1.118 | 0.22 | Low RR + Low P | No | 0.334 | 58379 |
| 36043 | Herkimer County, NY | 1.03 | 0.31 | Low RR + Low P | No | 0.333 | 119068 |
| 13303 | Washington County, GA | 0.996 | 0.45 | Low RR + Low P | No | 0.333 | 39585 |
| 13265 | Taliaferro County, GA | 1.189 | 0.3 | Low RR + Low P | No | 0.333 | 3217 |
| 51015 | Augusta County, VA | 0.86 | 0.52 | Low RR + Low P | No | 0.333 | 156186 |
| 18069 | Huntington County, IN | 1.159 | 0.1 | Low RR + Low P | No | 0.333 | 73581 |
| 22119 | Webster Parish, LA | 0.681 | 0.63 | Low RR + Low P | No | 0.333 | 70765 |
| 5029 | Conway County, AR | 1.002 | 0.44 | Low RR + Low P | No | 0.333 | 42098 |
| 46123 | Tripp County, SD | 0.861 | 0.61 | Low RR + Low P | No | 0.333 | 11200 |
| 12097 | Osceola County, FL | 0.919 | 0.41 | Low RR + Low P | No | 0.333 | 860327 |
| 20041 | Dickinson County, KS | 1.034 | 0.42 | Low RR + Low P | No | 0.333 | 36800 |
| 38001 | Adams County, ND | 1.05 | 0.48 | Low RR + Low P | No | 0.333 | 4296 |
| 29171 | Putnam County, MO | 0.942 | 0.56 | Low RR + Low P | No | 0.333 | 9300 |
| 42115 | Susquehanna County, PA | 0.868 | 0.53 | Low RR + Low P | No | 0.332 | 76248 |
| 31075 | Grant County, NE | 0.827 | 0.65 | Low RR + Low P | No | 0.332 | 1149 |
| 5019 | Clark County, AR | 0.9 | 0.54 | Low RR + Low P | No | 0.332 | 42550 |
| 16073 | Owyhee County, ID | 1.147 | 0.28 | Low RR + Low P | No | 0.332 | 25367 |
| 51131 | Northampton County, VA | 0.904 | 0.56 | Low RR + Low P | No | 0.332 | 23999 |
| 26017 | Bay County, MI | 0.954 | 0.39 | Low RR + Low P | No | 0.331 | 205138 |
| 39113 | Montgomery County, OH | 1.127 | 0.01 | Low RR + Low P | No | 0.331 | 1067270 |
| 27037 | Dakota County, MN | 0.99 | 0.28 | Low RR + Low P | No | 0.331 | 890712 |
| 4023 | Santa Cruz County, AZ | 0.625 | 0.63 | Low RR + Low P | No | 0.33 | 97838 |
| 13309 | Wheeler County, GA | 0.837 | 0.62 | Low RR + Low P | No | 0.33 | 14391 |
| 36045 | Jefferson County, NY | 1.137 | 0.03 | Low RR + Low P | No | 0.33 | 230248 |
| 17027 | Clinton County, IL | 0.945 | 0.46 | Low RR + Low P | No | 0.33 | 73722 |
| 35051 | Sierra County, NM | 0.773 | 0.63 | Low RR + Low P | No | 0.329 | 22962 |
| 31139 | Pierce County, NE | 0.893 | 0.58 | Low RR + Low P | No | 0.329 | 14616 |
| 36093 | Schenectady County, NY | 0.903 | 0.44 | Low RR + Low P | No | 0.329 | 319596 |
| 56011 | Crook County, WY | 0.969 | 0.52 | Low RR + Low P | No | 0.329 | 15011 |
| 39037 | Darke County, OH | 1.147 | 0.05 | Low RR + Low P | No | 0.328 | 102941 |
| 49043 | Summit County, UT | 0.421 | 0.69 | Low RR + Low P | No | 0.328 | 85804 |
| 31065 | Furnas County, NE | 0.933 | 0.56 | Low RR + Low P | No | 0.328 | 9126 |
| 17011 | Bureau County, IL | 0.537 | 0.68 | Low RR + Low P | No | 0.328 | 65556 |
| 13249 | Schley County, GA | 1.095 | 0.42 | Low RR + Low P | No | 0.328 | 9023 |
| 42017 | Bucks County, PA | 0.593 | 0.59 | Low RR + Low P | No | 0.328 | 1291541 |
| 56017 | Hot Springs County, WY | 1.004 | 0.5 | Low RR + Low P | No | 0.328 | 9284 |
| 29005 | Atchison County, MO | 0.855 | 0.61 | Low RR + Low P | No | 0.328 | 10268 |
| 37007 | Anson County, NC | 1.044 | 0.36 | Low RR + Low P | No | 0.327 | 43855 |
| 21211 | Shelby County, KY | 0.701 | 0.6 | Low RR + Low P | No | 0.327 | 98357 |
| 37057 | Davidson County, NC | 1.048 | 0.17 | Low RR + Low P | No | 0.327 | 347184 |
| 20123 | Mitchell County, KS | 0.8 | 0.64 | Low RR + Low P | No | 0.327 | 11449 |
| 29039 | Cedar County, MO | 0.782 | 0.61 | Low RR + Low P | No | 0.327 | 29273 |
| 17189 | Washington County, IL | 1.039 | 0.42 | Low RR + Low P | No | 0.326 | 27159 |
| 48323 | Maverick County, TX | 0.902 | 0.47 | Low RR + Low P | No | 0.326 | 115390 |
| 17049 | Effingham County, IL | 0.966 | 0.42 | Low RR + Low P | No | 0.326 | 68631 |
| 51023 | Botetourt County, VA | 0.945 | 0.46 | Low RR + Low P | No | 0.326 | 68225 |
| 26041 | Delta County, MI | 0.973 | 0.41 | Low RR + Low P | No | 0.325 | 73571 |
| 36099 | Seneca County, NY | 0.822 | 0.56 | Low RR + Low P | No | 0.325 | 64935 |
| 20069 | Gray County, KS | 0.884 | 0.58 | Low RR + Low P | No | 0.325 | 11454 |
| 51009 | Amherst County, VA | 1.024 | 0.35 | Low RR + Low P | No | 0.325 | 62868 |
| 37087 | Haywood County, NC | 1.147 | 0.03 | Low RR + Low P | No | 0.324 | 125584 |
| 20183 | Smith County, KS | 0.948 | 0.55 | Low RR + Low P | No | 0.324 | 7162 |
| 31177 | Washington County, NE | 1.023 | 0.39 | Low RR + Low P | No | 0.324 | 42319 |
| 17047 | Edwards County, IL | 0.826 | 0.62 | Low RR + Low P | No | 0.323 | 12026 |
| 48407 | San Jacinto County, TX | 0.992 | 0.4 | Low RR + Low P | No | 0.323 | 57276 |
| 6111 | Ventura County, CA | 0.994 | 0.22 | Low RR + Low P | No | 0.323 | 1662461 |
| 31117 | McPherson County, NE | 0.84 | 0.63 | Low RR + Low P | No | 0.323 | 762 |
| 29103 | Knox County, MO | 0.955 | 0.54 | Low RR + Low P | No | 0.323 | 7500 |
| 36115 | Washington County, NY | 1.113 | 0.08 | Low RR + Low P | No | 0.322 | 120857 |
| 13301 | Warren County, GA | 1.003 | 0.49 | Low RR + Low P | No | 0.322 | 10263 |
| 48123 | DeWitt County, TX | 1.216 | 0.02 | Low RR + Low P | No | 0.321 | 39683 |
| 12079 | Madison County, FL | 1.087 | 0.29 | Low RR + Low P | No | 0.321 | 36749 |
| 20163 | Rooks County, KS | 0.895 | 0.57 | Low RR + Low P | No | 0.321 | 9588 |
| 34015 | Gloucester County, NJ | 0.733 | 0.54 | Low RR + Low P | No | 0.321 | 615190 |
| 26005 | Allegan County, MI | 0.885 | 0.45 | Low RR + Low P | No | 0.321 | 243229 |
| 21151 | Madison County, KY | 0.917 | 0.42 | Low RR + Low P | No | 0.321 | 191886 |
| 6045 | Mendocino County, CA | 1.101 | 0.06 | Low RR + Low P | No | 0.321 | 179115 |
| 31123 | Morrill County, NE | 1.013 | 0.48 | Low RR + Low P | No | 0.32 | 9026 |
| 13131 | Grady County, GA | 0.946 | 0.46 | Low RR + Low P | No | 0.32 | 52060 |
| 8087 | Morgan County, CO | 1.008 | 0.36 | Low RR + Low P | No | 0.32 | 58790 |
| 20137 | Norton County, KS | 0.971 | 0.51 | Low RR + Low P | No | 0.319 | 10655 |
| 47021 | Cheatham County, TN | 1.04 | 0.27 | Low RR + Low P | No | 0.319 | 84018 |
| 48019 | Bandera County, TX | 0.711 | 0.61 | Low RR + Low P | No | 0.319 | 44757 |
| 23019 | Penobscot County, ME | 0.895 | 0.42 | Low RR + Low P | No | 0.319 | 310040 |
| 55011 | Buffalo County, WI | 1.061 | 0.36 | Low RR + Low P | No | 0.319 | 26809 |
| 56009 | Converse County, WY | 0.813 | 0.59 | Low RR + Low P | No | 0.319 | 27574 |
| 46013 | Brown County, SD | 1.045 | 0.26 | Low RR + Low P | No | 0.318 | 75634 |
| 49025 | Kane County, UT | 0.865 | 0.57 | Low RR + Low P | No | 0.318 | 16628 |
| 48047 | Brooks County, TX | 0.94 | 0.53 | Low RR + Low P | No | 0.318 | 13750 |
| 13155 | Irwin County, GA | 1.114 | 0.3 | Low RR + Low P | No | 0.318 | 18234 |
| 31089 | Holt County, NE | 0.852 | 0.58 | Low RR + Low P | No | 0.318 | 20106 |
| 18043 | Floyd County, IN | 1.105 | 0.05 | Low RR + Low P | No | 0.318 | 161430 |
| 30065 | Musselshell County, MT | 0.914 | 0.56 | Low RR + Low P | No | 0.318 | 10477 |
| 8113 | San Miguel County, CO | 0.809 | 0.61 | Low RR + Low P | No | 0.318 | 15876 |
| 20073 | Greenwood County, KS | 0.798 | 0.62 | Low RR + Low P | No | 0.317 | 11778 |
| 36033 | Franklin County, NY | 1.099 | 0.12 | Low RR + Low P | No | 0.317 | 92895 |
| 36075 | Oswego County, NY | 1.017 | 0.2 | Low RR + Low P | No | 0.317 | 236401 |
| 32033 | White Pine County, NV | 0.666 | 0.69 | Low RR + Low P | No | 0.317 | 17268 |
| 8067 | La Plata County, CO | 0.732 | 0.57 | Low RR + Low P | No | 0.316 | 112960 |
| 26103 | Marquette County, MI | 0.948 | 0.38 | Low RR + Low P | No | 0.316 | 133547 |
| 29123 | Madison County, MO | 0.933 | 0.51 | Low RR + Low P | No | 0.316 | 25455 |
| 13105 | Elbert County, GA | 1.096 | 0.25 | Low RR + Low P | No | 0.316 | 39886 |
| 17065 | Hamilton County, IL | 0.851 | 0.58 | Low RR + Low P | No | 0.316 | 15892 |
| 1129 | Washington County, AL | 0.512 | 0.71 | Low RR + Low P | No | 0.315 | 30116 |
| 18161 | Union County, IN | 0.921 | 0.54 | Low RR + Low P | No | 0.315 | 13940 |
| 18157 | Tippecanoe County, IN | 0.947 | 0.32 | Low RR + Low P | No | 0.315 | 377029 |
| 17013 | Calhoun County, IL | 0.908 | 0.56 | Low RR + Low P | No | 0.315 | 8670 |
| 12105 | Polk County, FL | 0.869 | 0.42 | Low RR + Low P | No | 0.315 | 1606712 |
| 47151 | Scott County, TN | 1.018 | 0.36 | Low RR + Low P | No | 0.315 | 44170 |
| 38099 | Walsh County, ND | 0.939 | 0.51 | Low RR + Low P | No | 0.314 | 20765 |
| 21223 | Trimble County, KY | 1.018 | 0.43 | Low RR + Low P | No | 0.314 | 17134 |
| 30099 | Teton County, MT | 0.918 | 0.54 | Low RR + Low P | No | 0.314 | 12776 |
| 19119 | Lyon County, IA | 0.937 | 0.51 | Low RR + Low P | No | 0.314 | 24501 |
| 21061 | Edmonson County, KY | 1.079 | 0.31 | Low RR + Low P | No | 0.314 | 24750 |
| 17159 | Richland County, IL | 0.941 | 0.49 | Low RR + Low P | No | 0.314 | 31043 |
| 39117 | Morrow County, OH | 1.105 | 0.13 | Low RR + Low P | No | 0.314 | 70931 |
| 42101 | Philadelphia County, PA | 1.059 | 0.03 | Low RR + Low P | No | 0.313 | 3117378 |
| 4005 | Coconino County, AZ | 0.855 | 0.46 | Low RR + Low P | No | 0.313 | 288695 |
| 38073 | Ransom County, ND | 0.946 | 0.52 | Low RR + Low P | No | 0.312 | 11250 |
| 17021 | Christian County, IL | 0.876 | 0.5 | Low RR + Low P | No | 0.312 | 66618 |
| 39049 | Franklin County, OH | 1.058 | 0.02 | Low RR + Low P | No | 0.312 | 2647793 |
| 6083 | Santa Barbara County, CA | 1.065 | 0.02 | Low RR + Low P | No | 0.311 | 885172 |
| 51011 | Appomattox County, VA | 1.071 | 0.28 | Low RR + Low P | No | 0.311 | 33567 |
| 48025 | Bee County, TX | 1.099 | 0.14 | Low RR + Low P | No | 0.311 | 61842 |
| 29183 | St. Charles County, MO | 0.61 | 0.56 | Low RR + Low P | No | 0.31 | 830457 |
| 26075 | Jackson County, MI | 1.061 | 0.06 | Low RR + Low P | No | 0.31 | 319182 |
| 20143 | Ottawa County, KS | 0.939 | 0.52 | Low RR + Low P | No | 0.31 | 11624 |
| 46073 | Jerauld County, SD | 1.095 | 0.38 | Low RR + Low P | No | 0.31 | 3322 |
| 55113 | Sawyer County, WI | 1.185 | 0.03 | Low RR + Low P | No | 0.31 | 37073 |
| 20197 | Wabaunsee County, KS | 0.902 | 0.55 | Low RR + Low P | No | 0.31 | 14066 |
| 8073 | Lincoln County, CO | 1.026 | 0.43 | Low RR + Low P | No | 0.31 | 10990 |
| 13221 | Oglethorpe County, GA | 0.742 | 0.6 | Low RR + Low P | No | 0.31 | 31285 |
| 13003 | Atkinson County, GA | 0.641 | 0.69 | Low RR + Low P | No | 0.309 | 16453 |
| 25015 | Hampshire County, MA | 0.589 | 0.58 | Low RR + Low P | No | 0.309 | 325020 |
| 49017 | Garfield County, UT | 0.996 | 0.47 | Low RR + Low P | No | 0.309 | 10572 |
| 30023 | Deer Lodge County, MT | 0.76 | 0.61 | Low RR + Low P | No | 0.309 | 19217 |
| 48267 | Kimble County, TX | 0.895 | 0.56 | Low RR + Low P | No | 0.309 | 8862 |
| 27071 | Koochiching County, MN | 0.994 | 0.42 | Low RR + Low P | No | 0.308 | 23599 |
| 55065 | Lafayette County, WI | 0.632 | 0.65 | Low RR + Low P | No | 0.308 | 33851 |
| 6043 | Mariposa County, CA | 1.058 | 0.3 | Low RR + Low P | No | 0.308 | 33972 |
| 37059 | Davie County, NC | 0.962 | 0.36 | Low RR + Low P | No | 0.308 | 88714 |
| 29079 | Grundy County, MO | 0.779 | 0.6 | Low RR + Low P | No | 0.308 | 19659 |
| 26033 | Chippewa County, MI | 1.027 | 0.26 | Low RR + Low P | No | 0.307 | 72471 |
| 37019 | Brunswick County, NC | 0.761 | 0.52 | Low RR + Low P | No | 0.307 | 312872 |
| 16077 | Power County, ID | 0.889 | 0.55 | Low RR + Low P | No | 0.307 | 16389 |
| 47149 | Rutherford County, TN | 0.917 | 0.33 | Low RR + Low P | No | 0.307 | 727783 |
| 8111 | San Juan County, CO | 0.724 | 0.67 | Low RR + Low P | No | 0.306 | 1608 |
| 17151 | Pope County, IL | 0.925 | 0.54 | Low RR + Low P | No | 0.306 | 7453 |
| 48007 | Aransas County, TX | 1.048 | 0.26 | Low RR + Low P | No | 0.306 | 50337 |
| 39069 | Henry County, OH | 1.138 | 0.05 | Low RR + Low P | No | 0.306 | 55046 |
| 16017 | Bonner County, ID | 0.974 | 0.3 | Low RR + Low P | No | 0.305 | 103954 |
| 29087 | Holt County, MO | 0.891 | 0.56 | Low RR + Low P | No | 0.305 | 8502 |
| 42133 | York County, PA | 0.84 | 0.43 | Low RR + Low P | No | 0.305 | 925689 |
| 18171 | Warren County, IN | 0.898 | 0.53 | Low RR + Low P | No | 0.305 | 17006 |
| 48379 | Rains County, TX | 0.955 | 0.46 | Low RR + Low P | No | 0.305 | 25822 |
| 39035 | Cuyahoga County, OH | 1.089 | 0 | Low RR + Low P | No | 0.305 | 2470082 |
| 20067 | Grant County, KS | 0.983 | 0.46 | Low RR + Low P | No | 0.305 | 14397 |
| 22021 | Caldwell Parish, LA | 0.729 | 0.62 | Low RR + Low P | No | 0.305 | 18882 |
| 17085 | Jo Daviess County, IL | 0.972 | 0.39 | Low RR + Low P | No | 0.305 | 43601 |
| 13201 | Miller County, GA | 0.819 | 0.59 | Low RR + Low P | No | 0.304 | 11521 |
| 36089 | St. Lawrence County, NY | 1.071 | 0.04 | Low RR + Low P | No | 0.304 | 214029 |
| 5035 | Crittenden County, AR | 1.015 | 0.22 | Low RR + Low P | No | 0.304 | 94190 |
| 19051 | Davis County, IA | 0.905 | 0.52 | Low RR + Low P | No | 0.304 | 18316 |
| 39031 | Coshocton County, OH | 0.867 | 0.48 | Low RR + Low P | No | 0.303 | 73484 |
| 29125 | Maries County, MO | 0.847 | 0.56 | Low RR + Low P | No | 0.303 | 16862 |
| 29157 | Perry County, MO | 0.735 | 0.59 | Low RR + Low P | No | 0.302 | 37828 |
| 39111 | Monroe County, OH | 0.969 | 0.43 | Low RR + Low P | No | 0.302 | 26368 |
| 25007 | Dukes County, MA | 0.413 | 0.68 | Low RR + Low P | No | 0.302 | 41781 |
| 51101 | King William County, VA | 0.681 | 0.61 | Low RR + Low P | No | 0.302 | 37559 |
| 18029 | Dearborn County, IN | 1.036 | 0.17 | Low RR + Low P | No | 0.302 | 102115 |
| 26163 | Wayne County, MI | 1.075 | 0 | Low RR + Low P | No | 0.301 | 3510111 |
| 51085 | Hanover County, VA | 0.419 | 0.6 | Low RR + Low P | No | 0.301 | 227008 |
| 26063 | Huron County, MI | 1.125 | 0.04 | Low RR + Low P | No | 0.301 | 62072 |
| 39093 | Lorain County, OH | 1.003 | 0.11 | Low RR + Low P | No | 0.301 | 633941 |
| 16037 | Custer County, ID | 0.919 | 0.53 | Low RR + Low P | No | 0.3 | 9041 |
| 1005 | Barbour County, AL | 0.729 | 0.58 | Low RR + Low P | No | 0.3 | 49285 |
| 18141 | St. Joseph County, IN | 1.065 | 0.01 | Low RR + Low P | No | 0.3 | 545130 |
| 31167 | Stanton County, NE | 0.908 | 0.53 | Low RR + Low P | No | 0.3 | 11621 |
| 6109 | Tuolumne County, CA | 0.969 | 0.28 | Low RR + Low P | No | 0.3 | 108791 |
| 48201 | Harris County, TX | 1.069 | 0 | Low RR + Low P | No | 0.299 | 9616462 |
| 55127 | Walworth County, WI | 0.954 | 0.28 | Low RR + Low P | No | 0.299 | 211486 |
| 29023 | Butler County, MO | 0.424 | 0.63 | Low RR + Low P | No | 0.299 | 84114 |
| 48187 | Guadalupe County, TX | 1.072 | 0 | Low RR + Low P | No | 0.299 | 371156 |
| 36031 | Essex County, NY | 0.959 | 0.34 | Low RR + Low P | No | 0.298 | 73538 |
| 48193 | Hamilton County, TX | 0.645 | 0.66 | Low RR + Low P | No | 0.298 | 16932 |
| 17123 | Marshall County, IL | 1.037 | 0.33 | Low RR + Low P | No | 0.298 | 23354 |
| 17175 | Stark County, IL | 0.869 | 0.56 | Low RR + Low P | No | 0.297 | 10531 |
| 5101 | Newton County, AR | 0.547 | 0.7 | Low RR + Low P | No | 0.297 | 14172 |
| 37199 | Yancey County, NC | 0.962 | 0.4 | Low RR + Low P | No | 0.297 | 37706 |
| 42031 | Clarion County, PA | 0.918 | 0.41 | Low RR + Low P | No | 0.296 | 74197 |
| 35053 | Socorro County, NM | 0.965 | 0.41 | Low RR + Low P | No | 0.296 | 32074 |
| 6093 | Siskiyou County, CA | 0.949 | 0.34 | Low RR + Low P | No | 0.296 | 86691 |
| 17153 | Pulaski County, IL | 0.929 | 0.51 | Low RR + Low P | No | 0.295 | 9861 |
| 29075 | Gentry County, MO | 0.756 | 0.61 | Low RR + Low P | No | 0.295 | 12561 |
| 37151 | Randolph County, NC | 1.033 | 0.05 | Low RR + Low P | No | 0.295 | 293654 |
| 8123 | Weld County, CO | 0.869 | 0.36 | Low RR + Low P | No | 0.295 | 709708 |
| 48381 | Randall County, TX | 0.952 | 0.25 | Low RR + Low P | No | 0.295 | 294499 |
| 17007 | Boone County, IL | 0.813 | 0.49 | Low RR + Low P | No | 0.295 | 106270 |
| 39101 | Marion County, OH | 1.076 | 0.02 | Low RR + Low P | No | 0.295 | 129460 |
| 5039 | Dallas County, AR | 0.955 | 0.48 | Low RR + Low P | No | 0.295 | 12381 |
| 19097 | Jackson County, IA | 0.932 | 0.44 | Low RR + Low P | No | 0.295 | 38688 |
| 36041 | Hamilton County, NY | 0.772 | 0.6 | Low RR + Low P | No | 0.295 | 10203 |
| 30061 | Mineral County, MT | 0.909 | 0.52 | Low RR + Low P | No | 0.294 | 10110 |
| 27007 | Beltrami County, MN | 0.973 | 0.27 | Low RR + Low P | No | 0.294 | 93229 |
| 35011 | DeBaca County, NM | 1.066 | 0.36 | Low RR + Low P | No | 0.294 | 3349 |
| 26029 | Charlevoix County, MI | 0.751 | 0.56 | Low RR + Low P | No | 0.294 | 52332 |
| 48369 | Parmer County, TX | 0.911 | 0.5 | Low RR + Low P | No | 0.294 | 19266 |
| 45053 | Jasper County, SC | 1.068 | 0.11 | Low RR + Low P | No | 0.294 | 65530 |
| 22035 | East Carroll Parish, LA | 0.928 | 0.5 | Low RR + Low P | No | 0.293 | 13768 |
| 29045 | Clark County, MO | 0.759 | 0.6 | Low RR + Low P | No | 0.293 | 13356 |
| 27029 | Clearwater County, MN | 0.777 | 0.59 | Low RR + Low P | No | 0.293 | 17282 |
| 20149 | Pottawatomie County, KS | 0.991 | 0.3 | Low RR + Low P | No | 0.293 | 52674 |
| 51051 | Dickenson County, VA | 0.827 | 0.55 | Low RR + Low P | No | 0.293 | 27373 |
| 55039 | Fond du Lac County, WI | 1.03 | 0.06 | Low RR + Low P | No | 0.293 | 207760 |
| 2100 | Haines Borough, AK | 0.886 | 0.55 | Low RR + Low P | No | 0.293 | 4140 |
| 27035 | Crow Wing County, MN | 0.962 | 0.25 | Low RR + Low P | No | 0.293 | 136188 |
| 6073 | San Diego County, CA | 1.055 | 0 | Low RR + Low P | No | 0.293 | 6547149 |
| 35021 | Harding County, NM | 0.628 | 0.69 | Low RR + Low P | No | 0.293 | 1245 |
| 46011 | Brookings County, SD | 1.026 | 0.18 | Low RR + Low P | No | 0.292 | 71471 |
| 51135 | Nottoway County, VA | 1.052 | 0.25 | Low RR + Low P | No | 0.292 | 31121 |
| 19009 | Audubon County, IA | 0.769 | 0.6 | Low RR + Low P | No | 0.291 | 11109 |
| 47043 | Dickson County, TN | 1.097 | 0 | Low RR + Low P | No | 0.291 | 112456 |
| 37161 | Rutherford County, NC | 0.979 | 0.2 | Low RR + Low P | No | 0.29 | 130452 |
| 20167 | Russell County, KS | 0.674 | 0.64 | Low RR + Low P | No | 0.29 | 13456 |
| 30015 | Chouteau County, MT | 0.918 | 0.51 | Low RR + Low P | No | 0.29 | 11755 |
| 6061 | Placer County, CA | 1.039 | 0 | Low RR + Low P | No | 0.29 | 841635 |
| 27031 | Cook County, MN | 0.909 | 0.51 | Low RR + Low P | No | 0.29 | 11360 |
| 46051 | Grant County, SD | 0.797 | 0.58 | Low RR + Low P | No | 0.29 | 15039 |
| 42083 | Mc Kean County, PA | 0.723 | 0.55 | Low RR + Low P | No | 0.29 | 79338 |
| 42005 | Armstrong County, PA | 0.941 | 0.3 | Low RR + Low P | No | 0.29 | 128778 |
| 37085 | Harnett County, NC | 1.062 | 0 | Low RR + Low P | No | 0.29 | 279955 |
| 55069 | Lincoln County, WI | 1.087 | 0.07 | Low RR + Low P | No | 0.29 | 56760 |
| 48325 | Medina County, TX | 1.045 | 0.06 | Low RR + Low P | No | 0.289 | 108454 |
| 25003 | Berkshire County, MA | 0.676 | 0.53 | Low RR + Low P | No | 0.289 | 254365 |
| 31031 | Cherry County, NE | 0.976 | 0.43 | Low RR + Low P | No | 0.288 | 10977 |
| 29115 | Linn County, MO | 0.949 | 0.43 | Low RR + Low P | No | 0.288 | 23617 |
| 18115 | Ohio County, IN | 0.853 | 0.55 | Low RR + Low P | No | 0.288 | 12101 |
| 5059 | Hot Spring County, AR | 1.035 | 0.16 | Low RR + Low P | No | 0.288 | 66407 |
| 28063 | Jefferson County, MS | 0.877 | 0.53 | Low RR + Low P | No | 0.287 | 14008 |
| 51003 | Albemarle County, VA | 0.497 | 0.57 | Low RR + Low P | No | 0.287 | 230521 |
| 17167 | Sangamon County, IL | 1.042 | 0 | Low RR + Low P | No | 0.287 | 387642 |
| 29089 | Howard County, MO | 0.885 | 0.51 | Low RR + Low P | No | 0.287 | 20280 |
| 38067 | Pembina County, ND | 0.703 | 0.62 | Low RR + Low P | No | 0.287 | 13406 |
| 42019 | Butler County, PA | 0.839 | 0.39 | Low RR + Low P | No | 0.286 | 395781 |
| 51530 | Buena Vista city, VA | 0.934 | 0.48 | Low RR + Low P | No | 0.286 | 13135 |
| 5087 | Madison County, AR | 0.874 | 0.48 | Low RR + Low P | No | 0.286 | 35238 |
| 36091 | Saratoga County, NY | 0.766 | 0.45 | Low RR + Low P | No | 0.285 | 477179 |
| 39089 | Licking County, OH | 1.027 | 0.02 | Low RR + Low P | No | 0.285 | 364966 |
| 36039 | Greene County, NY | 0.84 | 0.45 | Low RR + Low P | No | 0.285 | 94407 |
| 51031 | Campbell County, VA | 1.011 | 0.12 | Low RR + Low P | No | 0.284 | 110335 |
| 31161 | Sheridan County, NE | 0.83 | 0.56 | Low RR + Low P | No | 0.284 | 9934 |
| 37109 | Lincoln County, NC | 0.86 | 0.39 | Low RR + Low P | No | 0.284 | 188783 |
| 34037 | Sussex County, NJ | 0.936 | 0.23 | Low RR + Low P | No | 0.284 | 291707 |
| 26049 | Genesee County, MI | 1.047 | 0 | Low RR + Low P | No | 0.283 | 803445 |
| 25011 | Franklin County, MA | 0.589 | 0.56 | Low RR + Low P | No | 0.283 | 141831 |
| 2020 | Anchorage Borough, AK | 0.817 | 0.4 | Low RR + Low P | No | 0.283 | 573058 |
| 31013 | Box Butte County, NE | 0.737 | 0.58 | Low RR + Low P | No | 0.283 | 21375 |
| 20193 | Thomas County, KS | 0.764 | 0.58 | Low RR + Low P | No | 0.283 | 15773 |
| 39051 | Fulton County, OH | 1.021 | 0.12 | Low RR + Low P | No | 0.282 | 84165 |
| 45091 | York County, SC | 1.048 | 0 | Low RR + Low P | No | 0.282 | 592521 |
| 48453 | Travis County, TX | 0.962 | 0.07 | Low RR + Low P | No | 0.281 | 2662511 |
| 39013 | Belmont County, OH | 0.982 | 0.15 | Low RR + Low P | No | 0.281 | 130413 |
| 55017 | Chippewa County, WI | 0.898 | 0.33 | Low RR + Low P | No | 0.28 | 133723 |
| 35035 | Otero County, NM | 0.959 | 0.2 | Low RR + Low P | No | 0.28 | 137352 |
| 13225 | Peach County, GA | 0.799 | 0.5 | Low RR + Low P | No | 0.28 | 57291 |
| 1019 | Cherokee County, AL | 0.613 | 0.58 | Low RR + Low P | No | 0.28 | 51019 |
| 42053 | Forest County, PA | 0.866 | 0.52 | Low RR + Low P | No | 0.28 | 13052 |
| 6025 | Imperial County, CA | 1.012 | 0.02 | Low RR + Low P | No | 0.279 | 357997 |
| 8019 | Clear Creek County, CO | 0.73 | 0.59 | Low RR + Low P | No | 0.279 | 18514 |
| 39139 | Richland County, OH | 0.994 | 0.06 | Low RR + Low P | No | 0.279 | 250325 |
| 8065 | Lake County, CO | 0.941 | 0.45 | Low RR + Low P | No | 0.279 | 14711 |
| 2170 | Matanuska-Susitna Borough, AK | 0.627 | 0.53 | Low RR + Low P | No | 0.279 | 228711 |
| 29057 | Dade County, MO | 0.632 | 0.63 | Low RR + Low P | No | 0.279 | 15381 |
| 48043 | Brewster County, TX | 0.708 | 0.59 | Low RR + Low P | No | 0.279 | 18857 |
| 20031 | Coffey County, KS | 0.705 | 0.6 | Low RR + Low P | No | 0.279 | 16503 |
| 29149 | Oregon County, MO | 0.55 | 0.65 | Low RR + Low P | No | 0.279 | 17423 |
| 20157 | Republic County, KS | 0.723 | 0.6 | Low RR + Low P | No | 0.279 | 9280 |
| 6049 | Modoc County, CA | 0.749 | 0.58 | Low RR + Low P | No | 0.278 | 17119 |
| 42099 | Perry County, PA | 0.724 | 0.52 | Low RR + Low P | No | 0.278 | 92196 |
| 21177 | Muhlenberg County, KY | 1.084 | 0.02 | Low RR + Low P | No | 0.277 | 61264 |
| 22075 | Plaquemines Parish, LA | 0.452 | 0.62 | Low RR + Low P | No | 0.277 | 45004 |
| 17133 | Monroe County, IL | 0.798 | 0.49 | Low RR + Low P | No | 0.277 | 69958 |
| 16003 | Adams County, ID | 0.905 | 0.5 | Low RR + Low P | No | 0.277 | 9694 |
| 29035 | Carter County, MO | 0.903 | 0.5 | Low RR + Low P | No | 0.277 | 10557 |
| 29141 | Morgan County, MO | 0.754 | 0.53 | Low RR + Low P | No | 0.276 | 43756 |
| 2220 | Sitka Borough, AK | 0.775 | 0.56 | Low RR + Low P | No | 0.276 | 16635 |
| 21097 | Harrison County, KY | 1.03 | 0.19 | Low RR + Low P | No | 0.276 | 38520 |
| 48377 | Presidio County, TX | 0.736 | 0.59 | Low RR + Low P | No | 0.276 | 11749 |
| 36083 | Rensselaer County, NY | 0.896 | 0.27 | Low RR + Low P | No | 0.276 | 318726 |
| 42033 | Clearfield County, PA | 0.994 | 0.07 | Low RR + Low P | No | 0.276 | 154914 |
| 48031 | Blanco County, TX | 0.796 | 0.54 | Low RR + Low P | No | 0.276 | 25459 |
| 31179 | Wayne County, NE | 0.71 | 0.58 | Low RR + Low P | No | 0.276 | 19773 |
| 47119 | Maury County, TN | 0.874 | 0.32 | Low RR + Low P | No | 0.275 | 218756 |
| 28149 | Warren County, MS | 1.05 | 0.03 | Low RR + Low P | No | 0.275 | 84933 |
| 37145 | Person County, NC | 0.978 | 0.18 | Low RR + Low P | No | 0.274 | 79080 |
| 29205 | Shelby County, MO | 0.789 | 0.56 | Low RR + Low P | No | 0.274 | 11927 |
| 5143 | Washington County, AR | 0.64 | 0.5 | Low RR + Low P | No | 0.273 | 518231 |
| 39123 | Ottawa County, OH | 0.989 | 0.16 | Low RR + Low P | No | 0.273 | 79771 |
| 29047 | Clay County, MO | 0.967 | 0.05 | Low RR + Low P | No | 0.273 | 516702 |
| 42009 | Bedford County, PA | 0.775 | 0.48 | Low RR + Low P | No | 0.273 | 94771 |
| 17155 | Putnam County, IL | 0.84 | 0.53 | Low RR + Low P | No | 0.272 | 11114 |
| 48209 | Hays County, TX | 0.831 | 0.34 | Low RR + Low P | No | 0.272 | 549589 |
| 42079 | Luzerne County, PA | 0.994 | 0.01 | Low RR + Low P | No | 0.272 | 653884 |
| 12117 | Seminole County, FL | 0.738 | 0.43 | Low RR + Low P | No | 0.271 | 962894 |
| 20145 | Pawnee County, KS | 0.827 | 0.54 | Low RR + Low P | No | 0.271 | 12314 |
| 2050 | Bethel Census Area, AK | 0.678 | 0.57 | Low RR + Low P | No | 0.271 | 36483 |
| 31101 | Keith County, NE | 0.692 | 0.59 | Low RR + Low P | No | 0.27 | 16292 |
| 33005 | Cheshire County, NH | 0.958 | 0.15 | Low RR + Low P | No | 0.27 | 155166 |
| 46033 | Custer County, SD | 0.595 | 0.62 | Low RR + Low P | No | 0.27 | 18138 |
| 8091 | Ouray County, CO | 0.857 | 0.52 | Low RR + Low P | No | 0.27 | 10279 |
| 8095 | Phillips County, CO | 0.947 | 0.43 | Low RR + Low P | No | 0.27 | 8924 |
| 12077 | Liberty County, FL | 0.852 | 0.52 | Low RR + Low P | No | 0.27 | 15319 |
| 28123 | Scott County, MS | 0.809 | 0.48 | Low RR + Low P | No | 0.27 | 55067 |
| 29081 | Harrison County, MO | 1.038 | 0.25 | Low RR + Low P | No | 0.269 | 16437 |
| 26065 | Ingham County, MI | 0.992 | 0.01 | Low RR + Low P | No | 0.269 | 568114 |
| 17165 | Saline County, IL | 0.892 | 0.4 | Low RR + Low P | No | 0.269 | 45925 |
| 54095 | Tyler County, WV | 0.943 | 0.42 | Low RR + Low P | No | 0.269 | 16009 |
| 18145 | Shelby County, IN | 0.965 | 0.17 | Low RR + Low P | No | 0.268 | 90420 |
| 29091 | Howell County, MO | 0.968 | 0.17 | Low RR + Low P | No | 0.267 | 81358 |
| 12009 | Brevard County, FL | 0.819 | 0.32 | Low RR + Low P | No | 0.267 | 1274686 |
| 18071 | Jackson County, IN | 0.924 | 0.27 | Low RR + Low P | No | 0.267 | 92690 |
| 13269 | Taylor County, GA | 0.796 | 0.54 | Low RR + Low P | No | 0.266 | 15520 |
| 8014 | Broomfield County, CO | 0.705 | 0.48 | Low RR + Low P | No | 0.266 | 153055 |
| 5131 | Sebastian County, AR | 0.909 | 0.19 | Low RR + Low P | No | 0.265 | 258207 |
| 13075 | Cook County, GA | 0.613 | 0.58 | Low RR + Low P | No | 0.265 | 35123 |
| 12073 | Leon County, FL | 0.903 | 0.17 | Low RR + Low P | No | 0.264 | 594195 |
| 19159 | Ringgold County, IA | 0.874 | 0.5 | Low RR + Low P | No | 0.264 | 9330 |
| 56015 | Goshen County, WY | 0.907 | 0.42 | Low RR + Low P | No | 0.264 | 25278 |
| 51021 | Bland County, VA | 0.954 | 0.39 | Low RR + Low P | No | 0.263 | 12317 |
| 53057 | Skagit County, WA | 1.003 | 0 | Low RR + Low P | No | 0.263 | 262739 |
| 42125 | Washington County, PA | 0.94 | 0.1 | Low RR + Low P | No | 0.263 | 420172 |
| 26071 | Iron County, MI | 0.898 | 0.42 | Low RR + Low P | No | 0.262 | 23445 |
| 26141 | Presque Isle County, MI | 1.005 | 0.22 | Low RR + Low P | No | 0.262 | 26628 |
| 35003 | Catron County, NM | 0.831 | 0.53 | Low RR + Low P | No | 0.262 | 7618 |
| 22013 | Bienville Parish, LA | 0.493 | 0.62 | Low RR + Low P | No | 0.262 | 24978 |
| 38089 | Stark County, ND | 0.913 | 0.3 | Low RR + Low P | No | 0.261 | 65719 |
| 23023 | Sagadahoc County, ME | 0.565 | 0.56 | Low RR + Low P | No | 0.261 | 74885 |
| 32027 | Pershing County, NV | 0.923 | 0.43 | Low RR + Low P | No | 0.261 | 12814 |
| 39153 | Summit County, OH | 0.966 | 0.01 | Low RR + Low P | No | 0.26 | 1071502 |
| 2130 | Ketchikan Gateway Borough, AK | 0.702 | 0.56 | Low RR + Low P | No | 0.26 | 27510 |
| 20043 | Doniphan County, KS | 0.775 | 0.54 | Low RR + Low P | No | 0.26 | 14968 |
| 2110 | Juneau Borough, AK | 0.705 | 0.51 | Low RR + Low P | No | 0.26 | 63295 |
| 12065 | Jefferson County, FL | 0.833 | 0.48 | Low RR + Low P | No | 0.26 | 30519 |
| 26155 | Shiawassee County, MI | 0.931 | 0.17 | Low RR + Low P | No | 0.26 | 135992 |
| 48223 | Hopkins County, TX | 0.958 | 0.17 | Low RR + Low P | No | 0.26 | 75965 |
| 6037 | Los Angeles County, CA | 0.979 | 0 | Low RR + Low P | No | 0.26 | 19383110 |
| 51678 | Lexington city, VA | 0.87 | 0.48 | Low RR + Low P | No | 0.259 | 15034 |
| 18077 | Jefferson County, IN | 1.018 | 0.04 | Low RR + Low P | No | 0.259 | 65992 |
| 36111 | Ulster County, NY | 0.757 | 0.4 | Low RR + Low P | No | 0.258 | 364653 |
| 5009 | Boone County, AR | 0.562 | 0.55 | Low RR + Low P | No | 0.258 | 76810 |
| 46015 | Brule County, SD | 0.93 | 0.42 | Low RR + Low P | No | 0.257 | 10612 |
| 5075 | Lawrence County, AR | 0.447 | 0.6 | Low RR + Low P | No | 0.257 | 32512 |
| 5049 | Fulton County, AR | 0.559 | 0.59 | Low RR + Low P | No | 0.257 | 24776 |
| 20001 | Allen County, KS | 0.696 | 0.56 | Low RR + Low P | No | 0.257 | 24901 |
| 29179 | Reynolds County, MO | 0.778 | 0.54 | Low RR + Low P | No | 0.256 | 11946 |
| 49029 | Morgan County, UT | 0.746 | 0.53 | Low RR + Low P | No | 0.255 | 25837 |
| 20131 | Nemaha County, KS | 0.751 | 0.54 | Low RR + Low P | No | 0.255 | 20278 |
| 1073 | Jefferson County, AL | 0.812 | 0.29 | Low RR + Low P | No | 0.255 | 1327979 |
| 29203 | Shannon County, MO | 0.802 | 0.52 | Low RR + Low P | No | 0.255 | 14454 |
| 28127 | Simpson County, MS | 0.839 | 0.42 | Low RR + Low P | No | 0.255 | 51338 |
| 28001 | Adams County, MS | 0.6 | 0.55 | Low RR + Low P | No | 0.254 | 57263 |
| 6053 | Monterey County, CA | 0.848 | 0.24 | Low RR + Low P | No | 0.254 | 863607 |
| 13183 | Long County, GA | 0.617 | 0.56 | Low RR + Low P | No | 0.254 | 37989 |
| 51047 | Culpeper County, VA | 0.587 | 0.52 | Low RR + Low P | No | 0.254 | 109263 |
| 1103 | Morgan County, AL | 0.944 | 0.06 | Low RR + Low P | No | 0.253 | 249364 |
| 15005 | Kalawao County, HI | 0.887 | 0.49 | Low RR + Low P | No | 0.253 | 162 |
| 39155 | Trumbull County, OH | 0.948 | 0.03 | Low RR + Low P | No | 0.253 | 401099 |
| 54051 | Marshall County, WV | 0.856 | 0.37 | Low RR + Low P | No | 0.252 | 59072 |
| 1131 | Wilcox County, AL | 0.559 | 0.59 | Low RR + Low P | No | 0.252 | 20083 |
| 20015 | Butler County, KS | 0.789 | 0.39 | Low RR + Low P | No | 0.252 | 136921 |
| 36051 | Livingston County, NY | 0.61 | 0.51 | Low RR + Low P | No | 0.251 | 122498 |
| 39061 | Hamilton County, OH | 0.946 | 0.01 | Low RR + Low P | No | 0.251 | 1651779 |
| 2150 | Kodiak Island Borough, AK | 0.651 | 0.56 | Low RR + Low P | No | 0.251 | 25204 |
| 36053 | Madison County, NY | 0.871 | 0.26 | Low RR + Low P | No | 0.251 | 133975 |
| 29155 | Pemiscot County, MO | 0.541 | 0.58 | Low RR + Low P | No | 0.25 | 29478 |
| 34041 | Warren County, NJ | 0.93 | 0.09 | Low RR + Low P | No | 0.25 | 222155 |
| 30031 | Gallatin County, MT | 0.54 | 0.5 | Low RR + Low P | No | 0.249 | 251347 |
| 5149 | Yell County, AR | 0.927 | 0.29 | Low RR + Low P | No | 0.249 | 40135 |
| 37189 | Watauga County, NC | 0.719 | 0.45 | Low RR + Low P | No | 0.249 | 109761 |
| 48339 | Montgomery County, TX | 0.896 | 0.08 | Low RR + Low P | No | 0.249 | 1390908 |
| 13081 | Crisp County, GA | 0.433 | 0.58 | Low RR + Low P | No | 0.248 | 39360 |
| 51005 | Alleghany County, VA | 0.928 | 0.32 | Low RR + Low P | No | 0.248 | 29310 |
| 22029 | Concordia Parish, LA | 0.88 | 0.37 | Low RR + Low P | No | 0.248 | 35714 |
| 13093 | Dooly County, GA | 0.513 | 0.6 | Low RR + Low P | No | 0.248 | 21566 |
| 47037 | Davidson County, TN | 0.962 | 0 | Low RR + Low P | No | 0.248 | 1419685 |
| 28021 | Claiborne County, MS | 0.813 | 0.5 | Low RR + Low P | No | 0.247 | 17392 |
| 12083 | Marion County, FL | 0.827 | 0.24 | Low RR + Low P | No | 0.247 | 806396 |
| 18049 | Fulton County, IN | 0.894 | 0.34 | Low RR + Low P | No | 0.247 | 40726 |
| 36067 | Onondaga County, NY | 0.844 | 0.2 | Low RR + Low P | No | 0.247 | 937601 |
| 6087 | Santa Cruz County, CA | 0.879 | 0.15 | Low RR + Low P | No | 0.247 | 525787 |
| 5125 | Saline County, AR | 0.583 | 0.48 | Low RR + Low P | No | 0.247 | 256989 |
| 6097 | Sonoma County, CA | 0.908 | 0.04 | Low RR + Low P | No | 0.246 | 964481 |
| 19187 | Webster County, IA | 0.96 | 0.11 | Low RR + Low P | No | 0.246 | 73137 |
| 29137 | Monroe County, MO | 0.571 | 0.59 | Low RR + Low P | No | 0.246 | 17333 |
| 42013 | Blair County, PA | 0.952 | 0.02 | Low RR + Low P | No | 0.246 | 241007 |
| 51097 | King and Queen County, VA | 0.723 | 0.55 | Low RR + Low P | No | 0.245 | 13434 |
| 56019 | Johnson County, WY | 0.687 | 0.56 | Low RR + Low P | No | 0.245 | 17478 |
| 46115 | Spink County, SD | 0.843 | 0.48 | Low RR + Low P | No | 0.245 | 12394 |
| 29133 | Mississippi County, MO | 0.535 | 0.58 | Low RR + Low P | No | 0.245 | 23487 |
| 29175 | Randolph County, MO | 0.75 | 0.47 | Low RR + Low P | No | 0.244 | 48735 |
| 2122 | Kenai Peninsula Borough, AK | 0.739 | 0.42 | Low RR + Low P | No | 0.244 | 121834 |
| 36029 | Erie County, NY | 0.952 | 0 | Low RR + Low P | No | 0.244 | 1894570 |
| 20011 | Bourbon County, KS | 0.604 | 0.56 | Low RR + Low P | No | 0.243 | 28851 |
| 32021 | Mineral County, NV | 0.929 | 0.38 | Low RR + Low P | No | 0.242 | 9065 |
| 46103 | Pennington County, SD | 0.958 | 0.01 | Low RR + Low P | No | 0.242 | 230209 |
| 19117 | Lucas County, IA | 0.756 | 0.52 | Low RR + Low P | No | 0.242 | 17449 |
| 18073 | Jasper County, IN | 0.899 | 0.24 | Low RR + Low P | No | 0.241 | 66880 |
| 46023 | Charles Mix County, SD | 0.971 | 0.24 | Low RR + Low P | No | 0.241 | 18472 |
| 18091 | La Porte County, IN | 0.962 | 0 | Low RR + Low P | No | 0.241 | 223528 |
| 42123 | Warren County, PA | 0.865 | 0.29 | Low RR + Low P | No | 0.24 | 75390 |
| 34009 | Cape May County, NJ | 0.927 | 0.06 | Low RR + Low P | No | 0.24 | 190015 |
| 37063 | Durham County, NC | 0.675 | 0.39 | Low RR + Low P | No | 0.24 | 670184 |
| 21063 | Elliott County, KY | 0.792 | 0.5 | Low RR + Low P | No | 0.24 | 14537 |
| 17147 | Piatt County, IL | 0.742 | 0.49 | Low RR + Low P | No | 0.24 | 33389 |
| 56035 | Sublette County, WY | 0.745 | 0.52 | Low RR + Low P | No | 0.239 | 17721 |
| 4021 | Pinal County, AZ | 0.714 | 0.35 | Low RR + Low P | No | 0.239 | 949276 |
| 27133 | Rock County, MN | 0.975 | 0.21 | Low RR + Low P | No | 0.239 | 19105 |
| 24041 | Talbot County, MD | 0.95 | 0.09 | Low RR + Low P | No | 0.239 | 75637 |
| 29223 | Wayne County, MO | 0.468 | 0.59 | Low RR + Low P | No | 0.238 | 21628 |
| 18017 | Cass County, IN | 0.894 | 0.22 | Low RR + Low P | No | 0.238 | 75203 |
| 17177 | Stephenson County, IL | 0.707 | 0.44 | Low RR + Low P | No | 0.238 | 86661 |
| 29049 | Clinton County, MO | 0.966 | 0.12 | Low RR + Low P | No | 0.238 | 42851 |
| 8049 | Grand County, CO | 0.474 | 0.57 | Low RR + Low P | No | 0.238 | 31669 |
| 29067 | Douglas County, MO | 0.824 | 0.44 | Low RR + Low P | No | 0.237 | 24209 |
| 6005 | Amador County, CA | 0.837 | 0.31 | Low RR + Low P | No | 0.237 | 83304 |
| 49051 | Wasatch County, UT | 0.475 | 0.54 | Low RR + Low P | No | 0.237 | 73741 |
| 20107 | Linn County, KS | 0.657 | 0.55 | Low RR + Low P | No | 0.237 | 19637 |
| 17139 | Moultrie County, IL | 0.783 | 0.46 | Low RR + Low P | No | 0.236 | 28717 |
| 51510 | Alexandria city, VA | 0.434 | 0.5 | Low RR + Low P | No | 0.236 | 310275 |
| 39169 | Wayne County, OH | 0.965 | 0 | Low RR + Low P | No | 0.235 | 233062 |
| 1015 | Calhoun County, AL | 0.422 | 0.5 | Low RR + Low P | No | 0.235 | 232209 |
| 30095 | Stillwater County, MT | 0.723 | 0.52 | Low RR + Low P | No | 0.235 | 18342 |
| 35045 | San Juan County, NM | 0.685 | 0.4 | Low RR + Low P | No | 0.235 | 241244 |
| 55025 | Dane County, WI | 0.909 | 0.01 | Low RR + Low P | No | 0.235 | 1143105 |
| 26015 | Barry County, MI | 0.864 | 0.2 | Low RR + Low P | No | 0.234 | 127210 |
| 34007 | Camden County, NJ | 0.688 | 0.35 | Low RR + Low P | No | 0.234 | 1051845 |
| 37101 | Johnston County, NC | 0.927 | 0.01 | Low RR + Low P | No | 0.234 | 476857 |
| 49019 | Grand County, UT | 0.665 | 0.54 | Low RR + Low P | No | 0.233 | 19477 |
| 13121 | Fulton County, GA | 0.866 | 0.06 | Low RR + Low P | No | 0.233 | 2152803 |
| 53071 | Walla Walla County, WA | 0.841 | 0.25 | Low RR + Low P | No | 0.233 | 123421 |
| 37081 | Guilford County, NC | 0.904 | 0.02 | Low RR + Low P | No | 0.233 | 1096323 |
| 25001 | Barnstable County, MA | 0.543 | 0.45 | Low RR + Low P | No | 0.233 | 463813 |
| 22047 | Iberville Parish, LA | 0.361 | 0.55 | Low RR + Low P | No | 0.232 | 59230 |
| 29195 | Saline County, MO | 0.982 | 0.04 | Low RR + Low P | No | 0.232 | 46052 |
| 48259 | Kendall County, TX | 0.855 | 0.24 | Low RR + Low P | No | 0.232 | 99556 |
| 4007 | Gila County, AZ | 0.899 | 0.13 | Low RR + Low P | No | 0.231 | 107925 |
| 42061 | Huntingdon County, PA | 0.895 | 0.16 | Low RR + Low P | No | 0.23 | 86811 |
| 8097 | Pitkin County, CO | 0.307 | 0.57 | Low RR + Low P | No | 0.23 | 33487 |
| 48415 | Scurry County, TX | 0.697 | 0.5 | Low RR + Low P | No | 0.23 | 32481 |
| 39151 | Stark County, OH | 0.934 | 0 | Low RR + Low P | No | 0.229 | 745501 |
| 21215 | Spencer County, KY | 0.816 | 0.39 | Low RR + Low P | No | 0.229 | 40743 |
| 16013 | Blaine County, ID | 0.435 | 0.55 | Low RR + Low P | No | 0.229 | 49942 |
| 26125 | Oakland County, MI | 0.881 | 0.02 | Low RR + Low P | No | 0.229 | 2541451 |
| 26157 | Tuscola County, MI | 0.927 | 0.05 | Low RR + Low P | No | 0.228 | 105734 |
| 5129 | Searcy County, AR | 0.712 | 0.52 | Low RR + Low P | No | 0.228 | 15712 |
| 36105 | Sullivan County, NY | 0.784 | 0.3 | Low RR + Low P | No | 0.228 | 159655 |
| 29151 | Osage County, MO | 0.598 | 0.54 | Low RR + Low P | No | 0.228 | 26866 |
| 5077 | Lee County, AR | 0.852 | 0.42 | Low RR + Low P | No | 0.228 | 16568 |
| 42113 | Sullivan County, PA | 0.951 | 0.27 | Low RR + Low P | No | 0.227 | 11679 |
| 47019 | Carter County, TN | 0.901 | 0.1 | Low RR + Low P | No | 0.227 | 113507 |
| 6051 | Mono County, CA | 0.565 | 0.55 | Low RR + Low P | No | 0.226 | 26068 |
| 33017 | Strafford County, NH | 0.813 | 0.21 | Low RR + Low P | No | 0.226 | 265814 |
| 30007 | Broadwater County, MT | 0.732 | 0.51 | Low RR + Low P | No | 0.226 | 15803 |
| 8109 | Saguache County, CO | 0.803 | 0.47 | Low RR + Low P | No | 0.225 | 13315 |
| 30057 | Madison County, MT | 0.565 | 0.56 | Low RR + Low P | No | 0.225 | 18773 |
| 26019 | Benzie County, MI | 0.877 | 0.29 | Low RR + Low P | No | 0.225 | 36768 |
| 24035 | Queen Anne's County, MD | 0.892 | 0.11 | Low RR + Low P | No | 0.224 | 104228 |
| 37185 | Warren County, NC | 0.882 | 0.28 | Low RR + Low P | No | 0.224 | 37513 |
| 37097 | Iredell County, NC | 0.813 | 0.18 | Low RR + Low P | No | 0.224 | 395584 |
| 53037 | Kittitas County, WA | 0.86 | 0.2 | Low RR + Low P | No | 0.224 | 90630 |
| 51036 | Charles City County, VA | 0.679 | 0.53 | Low RR + Low P | No | 0.224 | 13178 |
| 1101 | Montgomery County, AL | 0.723 | 0.3 | Low RR + Low P | No | 0.223 | 451281 |
| 20127 | Morris County, KS | 0.852 | 0.42 | Low RR + Low P | No | 0.223 | 10734 |
| 4025 | Yavapai County, AZ | 0.831 | 0.13 | Low RR + Low P | No | 0.223 | 495311 |
| 18003 | Allen County, IN | 0.887 | 0.02 | Low RR + Low P | No | 0.222 | 786189 |
| 51540 | Charlottesville city, VA | 0.69 | 0.41 | Low RR + Low P | No | 0.222 | 90255 |
| 37067 | Forsyth County, NC | 0.842 | 0.08 | Low RR + Low P | No | 0.222 | 782582 |
| 31037 | Colfax County, NE | 0.887 | 0.32 | Low RR + Low P | No | 0.222 | 21019 |
| 26077 | Kalamazoo County, MI | 0.852 | 0.07 | Low RR + Low P | No | 0.222 | 523107 |
| 23015 | Lincoln County, ME | 0.566 | 0.49 | Low RR + Low P | No | 0.222 | 72792 |
| 8117 | Summit County, CO | 0.369 | 0.53 | Low RR + Low P | No | 0.222 | 61101 |
| 16047 | Gooding County, ID | 0.679 | 0.49 | Low RR + Low P | No | 0.221 | 31940 |
| 47051 | Franklin County, TN | 0.588 | 0.47 | Low RR + Low P | No | 0.221 | 88773 |
| 29029 | Camden County, MO | 0.806 | 0.29 | Low RR + Low P | No | 0.221 | 87804 |
| 13171 | Lamar County, GA | 0.696 | 0.47 | Low RR + Low P | No | 0.221 | 39874 |
| 5089 | Marion County, AR | 0.74 | 0.44 | Low RR + Low P | No | 0.221 | 34803 |
| 23009 | Hancock County, ME | 0.884 | 0.11 | Low RR + Low P | No | 0.221 | 113075 |
| 19195 | Worth County, IA | 0.704 | 0.52 | Low RR + Low P | No | 0.221 | 14608 |
| 17107 | Logan County, IL | 0.885 | 0.19 | Low RR + Low P | No | 0.221 | 55256 |
| 36063 | Niagara County, NY | 0.924 | 0 | Low RR + Low P | No | 0.22 | 419924 |
| 29161 | Phelps County, MO | 0.574 | 0.47 | Low RR + Low P | No | 0.22 | 90557 |
| 18079 | Jennings County, IN | 0.905 | 0.14 | Low RR + Low P | No | 0.22 | 55106 |
| 19147 | Palo Alto County, IA | 0.836 | 0.41 | Low RR + Low P | No | 0.22 | 17600 |
| 19115 | Louisa County, IA | 0.742 | 0.48 | Low RR + Low P | No | 0.22 | 21130 |
| 26159 | Van Buren County, MI | 0.91 | 0.02 | Low RR + Low P | No | 0.219 | 151467 |
| 51610 | Falls Church city, VA | 0.57 | 0.53 | Low RR + Low P | No | 0.219 | 29174 |
| 56039 | Teton County, WY | 0.374 | 0.54 | Low RR + Low P | No | 0.218 | 46529 |
| 18015 | Carroll County, IN | 0.873 | 0.25 | Low RR + Low P | No | 0.218 | 41039 |
| 1085 | Lowndes County, AL | 0.649 | 0.52 | Low RR + Low P | No | 0.218 | 19500 |
| 37029 | Camden County, NC | 0.72 | 0.48 | Low RR + Low P | No | 0.217 | 22227 |
| 26013 | Baraga County, MI | 0.628 | 0.54 | Low RR + Low P | No | 0.217 | 16593 |
| 13079 | Crawford County, GA | 0.524 | 0.55 | Low RR + Low P | No | 0.217 | 24446 |
| 36095 | Schoharie County, NY | 0.86 | 0.22 | Low RR + Low P | No | 0.217 | 60196 |
| 17079 | Jasper County, IL | 0.918 | 0.26 | Low RR + Low P | No | 0.217 | 18316 |
| 1063 | Greene County, AL | 0.543 | 0.56 | Low RR + Low P | No | 0.217 | 14793 |
| 37115 | Madison County, NC | 0.884 | 0.21 | Low RR + Low P | No | 0.217 | 43952 |
| 8007 | Archuleta County, CO | 0.423 | 0.56 | Low RR + Low P | No | 0.217 | 28178 |
| 47061 | Grundy County, TN | 0.864 | 0.31 | Low RR + Low P | No | 0.216 | 27734 |
| 29065 | Dent County, MO | 0.717 | 0.46 | Low RR + Low P | No | 0.216 | 29135 |
| 22091 | St. Helena Parish, LA | 0.583 | 0.53 | Low RR + Low P | No | 0.216 | 21563 |
| 13023 | Bleckley County, GA | 0.569 | 0.53 | Low RR + Low P | No | 0.214 | 24744 |
| 29185 | St. Clair County, MO | 0.496 | 0.56 | Low RR + Low P | No | 0.214 | 19347 |
| 20121 | Miami County, KS | 0.776 | 0.32 | Low RR + Low P | No | 0.214 | 70111 |
| 5025 | Cleveland County, AR | 0.742 | 0.48 | Low RR + Low P | No | 0.214 | 14845 |
| 1099 | Monroe County, AL | 0.838 | 0.3 | Low RR + Low P | No | 0.214 | 38641 |
| 42021 | Cambria County, PA | 0.81 | 0.16 | Low RR + Low P | No | 0.214 | 262097 |
| 39103 | Medina County, OH | 0.771 | 0.19 | Low RR + Low P | No | 0.214 | 367531 |
| 28103 | Noxubee County, MS | 0.859 | 0.34 | Low RR + Low P | No | 0.213 | 19914 |
| 26061 | Houghton County, MI | 0.952 | 0 | Low RR + Low P | No | 0.213 | 74759 |
| 51033 | Caroline County, VA | 0.819 | 0.27 | Low RR + Low P | No | 0.213 | 64580 |
| 42029 | Chester County, PA | 0.577 | 0.36 | Low RR + Low P | No | 0.213 | 1096422 |
| 51125 | Nelson County, VA | 0.788 | 0.4 | Low RR + Low P | No | 0.212 | 29452 |
| 17193 | White County, IL | 0.856 | 0.31 | Low RR + Low P | No | 0.212 | 26996 |
| 16041 | Franklin County, ID | 0.575 | 0.52 | Low RR + Low P | No | 0.212 | 30664 |
| 36065 | Oneida County, NY | 0.901 | 0 | Low RR + Low P | No | 0.211 | 455913 |
| 22081 | Red River Parish, LA | 0.692 | 0.5 | Low RR + Low P | No | 0.211 | 14821 |
| 22059 | LaSalle Parish, LA | 0.5 | 0.53 | Low RR + Low P | No | 0.211 | 29621 |
| 47085 | Humphreys County, TN | 0.869 | 0.24 | Low RR + Low P | No | 0.211 | 38232 |
| 8107 | Routt County, CO | 0.352 | 0.52 | Low RR + Low P | No | 0.209 | 50136 |
| 17185 | Wabash County, IL | 0.849 | 0.33 | Low RR + Low P | No | 0.209 | 21982 |
| 18047 | Franklin County, IN | 0.815 | 0.3 | Low RR + Low P | No | 0.209 | 46179 |
| 49023 | Juab County, UT | 0.764 | 0.42 | Low RR + Low P | No | 0.209 | 25609 |
| 55091 | Pepin County, WI | 0.96 | 0.14 | Low RR + Low P | No | 0.209 | 14819 |
| 51157 | Rappahannock County, VA | 0.582 | 0.54 | Low RR + Low P | No | 0.209 | 14867 |
| 1007 | Bibb County, AL | 0.659 | 0.45 | Low RR + Low P | No | 0.209 | 43854 |
| 17029 | Coles County, IL | 0.905 | 0.02 | Low RR + Low P | No | 0.208 | 92406 |
| 51095 | James City County, VA | 0.516 | 0.43 | Low RR + Low P | No | 0.208 | 164137 |
| 6063 | Plumas County, CA | 0.771 | 0.37 | Low RR + Low P | No | 0.208 | 38574 |
| 17141 | Ogle County, IL | 0.827 | 0.17 | Low RR + Low P | No | 0.207 | 102610 |
| 42129 | Westmoreland County, PA | 0.846 | 0.02 | Low RR + Low P | No | 0.207 | 703161 |
| 42003 | Allegheny County, PA | 0.797 | 0.06 | Low RR + Low P | No | 0.206 | 2457430 |
| 36055 | Monroe County, NY | 0.655 | 0.27 | Low RR + Low P | No | 0.206 | 1499369 |
| 26149 | St. Joseph County, MI | 0.895 | 0.02 | Low RR + Low P | No | 0.206 | 121656 |
| 51830 | Williamsburg city, VA | 0.557 | 0.51 | Low RR + Low P | No | 0.206 | 31532 |
| 37055 | Dare County, NC | 0.675 | 0.39 | Low RR + Low P | No | 0.206 | 76097 |
| 36023 | Cortland County, NY | 0.661 | 0.38 | Low RR + Low P | No | 0.206 | 91833 |
| 35028 | Los Alamos County, NM | 0.361 | 0.53 | Low RR + Low P | No | 0.205 | 38717 |
| 27063 | Jackson County, MN | 0.62 | 0.51 | Low RR + Low P | No | 0.205 | 19817 |
| 33011 | Hillsborough County, NH | 0.793 | 0.08 | Low RR + Low P | No | 0.205 | 854847 |
| 15003 | Honolulu County, HI | 0.83 | 0.02 | Low RR + Low P | No | 0.204 | 1984236 |
| 1067 | Henry County, AL | 0.678 | 0.44 | Low RR + Low P | No | 0.204 | 35569 |
| 51061 | Fauquier County, VA | 0.545 | 0.41 | Low RR + Low P | No | 0.204 | 149842 |
| 13141 | Hancock County, GA | 0.847 | 0.34 | Low RR + Low P | No | 0.204 | 17093 |
| 1133 | Winston County, AL | 0.622 | 0.45 | Low RR + Low P | No | 0.204 | 47352 |
| 55089 | Ozaukee County, WI | 0.819 | 0.11 | Low RR + Low P | No | 0.204 | 186481 |
| 17195 | Whiteside County, IL | 0.749 | 0.27 | Low RR + Low P | No | 0.203 | 109053 |
| 4003 | Cochise County, AZ | 0.772 | 0.17 | Low RR + Low P | No | 0.203 | 250127 |
| 16081 | Teton County, ID | 0.752 | 0.42 | Low RR + Low P | No | 0.203 | 25068 |
| 29041 | Chariton County, MO | 0.654 | 0.5 | Low RR + Low P | No | 0.203 | 14794 |
| 27057 | Hubbard County, MN | 0.849 | 0.22 | Low RR + Low P | No | 0.202 | 44095 |
| 13175 | Laurens County, GA | 0.878 | 0.04 | Low RR + Low P | No | 0.202 | 99651 |
| 6105 | Trinity County, CA | 0.864 | 0.24 | Low RR + Low P | No | 0.202 | 31448 |
| 1021 | Chilton County, AL | 0.458 | 0.46 | Low RR + Low P | No | 0.201 | 92279 |
| 18149 | Starke County, IN | 0.895 | 0.11 | Low RR + Low P | No | 0.201 | 46409 |
| 23013 | Knox County, ME | 0.771 | 0.26 | Low RR + Low P | No | 0.201 | 82156 |
| 27149 | Stevens County, MN | 0.827 | 0.34 | Low RR + Low P | No | 0.2 | 19343 |
| 1121 | Talladega County, AL | 0.411 | 0.43 | Low RR + Low P | No | 0.2 | 161880 |
| 47133 | Overton County, TN | 0.694 | 0.4 | Low RR + Low P | No | 0.199 | 46320 |
| 23021 | Piscataquis County, ME | 0.765 | 0.36 | Low RR + Low P | No | 0.199 | 34889 |
| 20051 | Ellis County, KS | 0.747 | 0.31 | Low RR + Low P | No | 0.199 | 57650 |
| 29143 | New Madrid County, MO | 0.687 | 0.43 | Low RR + Low P | No | 0.198 | 31134 |
| 51199 | York County, VA | 0.665 | 0.31 | Low RR + Low P | No | 0.198 | 142116 |
| 13067 | Cobb County, GA | 0.521 | 0.33 | Low RR + Low P | No | 0.198 | 1548583 |
| 47165 | Sumner County, TN | 0.74 | 0.17 | Low RR + Low P | No | 0.198 | 411715 |
| 30021 | Dawson County, MT | 0.6 | 0.51 | Low RR + Low P | No | 0.198 | 17617 |
| 53033 | King County, WA | 0.802 | 0.02 | Low RR + Low P | No | 0.197 | 4536691 |
| 16015 | Boise County, ID | 0.634 | 0.5 | Low RR + Low P | No | 0.197 | 16877 |
| 39145 | Scioto County, OH | 0.902 | 0 | Low RR + Low P | No | 0.197 | 144158 |
| 1065 | Hale County, AL | 0.483 | 0.51 | Low RR + Low P | No | 0.197 | 29487 |
| 39075 | Holmes County, OH | 0.869 | 0.04 | Low RR + Low P | No | 0.197 | 88761 |
| 29147 | Nodaway County, MO | 0.389 | 0.51 | Low RR + Low P | No | 0.196 | 41410 |
| 23011 | Kennebec County, ME | 0.834 | 0.03 | Low RR + Low P | No | 0.196 | 253794 |
| 26055 | Grand Traverse County, MI | 0.854 | 0.02 | Low RR + Low P | No | 0.196 | 192719 |
| 26099 | Macomb County, MI | 0.788 | 0.04 | Low RR + Low P | No | 0.196 | 1749336 |
| 39041 | Delaware County, OH | 0.56 | 0.32 | Low RR + Low P | No | 0.196 | 458162 |
| 29186 | Ste. Genevieve County, MO | 0.674 | 0.42 | Low RR + Low P | No | 0.196 | 37255 |
| 1011 | Bullock County, AL | 0.621 | 0.49 | Low RR + Low P | No | 0.195 | 20040 |
| 39085 | Lake County, OH | 0.848 | 0 | Low RR + Low P | No | 0.195 | 463376 |
| 36037 | Genesee County, NY | 0.823 | 0.1 | Low RR + Low P | No | 0.195 | 114927 |
| 28011 | Bolivar County, MS | 0.857 | 0.12 | Low RR + Low P | No | 0.194 | 58429 |
| 22017 | Caddo Parish, LA | 0.862 | 0 | Low RR + Low P | No | 0.194 | 455320 |
| 20021 | Cherokee County, KS | 0.651 | 0.43 | Low RR + Low P | No | 0.194 | 38079 |
| 26031 | Cheboygan County, MI | 0.8 | 0.25 | Low RR + Low P | No | 0.194 | 52012 |
| 31039 | Cuming County, NE | 0.571 | 0.51 | Low RR + Low P | No | 0.194 | 17871 |
| 37179 | Union County, NC | 0.663 | 0.24 | Low RR + Low P | No | 0.193 | 505624 |
| 6001 | Alameda County, CA | 0.839 | 0 | Low RR + Low P | No | 0.193 | 3250447 |
| 5117 | Prairie County, AR | 0.594 | 0.5 | Low RR + Low P | No | 0.192 | 16113 |
| 26131 | Ontonagon County, MI | 0.934 | 0.14 | Low RR + Low P | No | 0.192 | 11827 |
| 5011 | Bradley County, AR | 0.796 | 0.35 | Low RR + Low P | No | 0.192 | 20272 |
| 5043 | Drew County, AR | 0.526 | 0.48 | Low RR + Low P | No | 0.192 | 33857 |
| 4019 | Pima County, AZ | 0.717 | 0.11 | Low RR + Low P | No | 0.192 | 2120638 |
| 51117 | Mecklenburg County, VA | 0.837 | 0.14 | Low RR + Low P | No | 0.19 | 61175 |
| 12035 | Flagler County, FL | 0.63 | 0.28 | Low RR + Low P | No | 0.19 | 258197 |
| 6013 | Contra Costa County, CA | 0.831 | 0 | Low RR + Low P | No | 0.19 | 2312044 |
| 41015 | Curry County, OR | 0.889 | 0.05 | Low RR + Low P | No | 0.189 | 46909 |
| 12005 | Bay County, FL | 0.729 | 0.14 | Low RR + Low P | No | 0.189 | 375979 |
| 12127 | Volusia County, FL | 0.804 | 0.01 | Low RR + Low P | No | 0.189 | 1170022 |
| 51133 | Northumberland County, VA | 0.638 | 0.46 | Low RR + Low P | No | 0.188 | 24669 |
| 8051 | Gunnison County, CO | 0.541 | 0.47 | Low RR + Low P | No | 0.188 | 34641 |
| 29003 | Andrew County, MO | 0.455 | 0.49 | Low RR + Low P | No | 0.188 | 36143 |
| 17197 | Will County, IL | 0.654 | 0.18 | Low RR + Low P | No | 0.187 | 1398774 |
| 8027 | Custer County, CO | 0.692 | 0.46 | Low RR + Low P | No | 0.187 | 10871 |
| 25019 | Nantucket County, MA | 0.523 | 0.49 | Low RR + Low P | No | 0.187 | 28887 |
| 16085 | Valley County, ID | 0.489 | 0.5 | Low RR + Low P | No | 0.186 | 25094 |
| 46019 | Butte County, SD | 0.739 | 0.39 | Low RR + Low P | No | 0.186 | 21653 |
| 12089 | Nassau County, FL | 0.55 | 0.33 | Low RR + Low P | No | 0.186 | 199327 |
| 12031 | Duval County, FL | 0.822 | 0 | Low RR + Low P | No | 0.185 | 2047833 |
| 42111 | Somerset County, PA | 0.753 | 0.14 | Low RR + Low P | No | 0.185 | 144826 |
| 18085 | Kosciusko County, IN | 0.808 | 0.05 | Low RR + Low P | No | 0.185 | 160928 |
| 6041 | Marin County, CA | 0.676 | 0.17 | Low RR + Low P | No | 0.185 | 510561 |
| 8119 | Teller County, CO | 0.892 | 0.03 | Low RR + Low P | No | 0.185 | 49477 |
| 34021 | Mercer County, NJ | 0.767 | 0.03 | Low RR + Low P | No | 0.185 | 762450 |
| 25027 | Worcester County, MA | 0.68 | 0.13 | Low RR + Low P | No | 0.184 | 1729739 |
| 37053 | Currituck County, NC | 0.634 | 0.37 | Low RR + Low P | No | 0.184 | 62601 |
| 47179 | Washington County, TN | 0.837 | 0 | Low RR + Low P | No | 0.184 | 274567 |
| 17089 | Kane County, IL | 0.495 | 0.29 | Low RR + Low P | No | 0.184 | 1029121 |
| 8105 | Rio Grande County, CO | 0.824 | 0.27 | Low RR + Low P | No | 0.184 | 22525 |
| 45051 | Horry County, SC | 0.745 | 0.05 | Low RR + Low P | No | 0.184 | 780625 |
| 29033 | Carroll County, MO | 0.658 | 0.45 | Low RR + Low P | No | 0.184 | 16811 |
| 28045 | Hancock County, MS | 0.87 | 0 | Low RR + Low P | No | 0.184 | 92166 |
| 29113 | Lincoln County, MO | 0.787 | 0.1 | Low RR + Low P | No | 0.183 | 127881 |
| 20169 | Saline County, KS | 0.801 | 0.09 | Low RR + Low P | No | 0.183 | 106487 |
| 12061 | Indian River County, FL | 0.519 | 0.31 | Low RR + Low P | No | 0.183 | 337062 |
| 29121 | Macon County, MO | 0.471 | 0.49 | Low RR + Low P | No | 0.182 | 30237 |
| 29131 | Miller County, MO | 0.683 | 0.34 | Low RR + Low P | No | 0.182 | 50993 |
| 5053 | Grant County, AR | 0.431 | 0.48 | Low RR + Low P | No | 0.182 | 36512 |
| 51685 | Manassas Park city, VA | 0.528 | 0.46 | Low RR + Low P | No | 0.182 | 33054 |
| 26143 | Roscommon County, MI | 0.882 | 0.04 | Low RR + Low P | No | 0.181 | 47545 |
| 31137 | Phelps County, NE | 0.711 | 0.41 | Low RR + Low P | No | 0.181 | 18045 |
| 29101 | Johnson County, MO | 0.808 | 0.06 | Low RR + Low P | No | 0.181 | 109411 |
| 13133 | Greene County, GA | 0.762 | 0.27 | Low RR + Low P | No | 0.181 | 40833 |
| 29153 | Ozark County, MO | 0.529 | 0.49 | Low RR + Low P | No | 0.18 | 17924 |
| 48085 | Collin County, TX | 0.698 | 0.07 | Low RR + Low P | No | 0.18 | 2354354 |
| 47007 | Bledsoe County, TN | 0.474 | 0.48 | Low RR + Low P | No | 0.18 | 29884 |
| 36071 | Orange County, NY | 0.819 | 0 | Low RR + Low P | No | 0.18 | 813961 |
| 1051 | Elmore County, AL | 0.483 | 0.35 | Low RR + Low P | No | 0.179 | 180041 |
| 1029 | Cleburne County, AL | 0.408 | 0.49 | Low RR + Low P | No | 0.179 | 31011 |
| 13173 | Lanier County, GA | 0.612 | 0.45 | Low RR + Low P | No | 0.179 | 20629 |
| 47189 | Wilson County, TN | 0.767 | 0.04 | Low RR + Low P | No | 0.179 | 322267 |
| 17035 | Cumberland County, IL | 0.607 | 0.45 | Low RR + Low P | No | 0.179 | 20569 |
| 36011 | Cayuga County, NY | 0.807 | 0.04 | Low RR + Low P | No | 0.179 | 149164 |
| 34005 | Burlington County, NJ | 0.664 | 0.14 | Low RR + Low P | No | 0.178 | 935268 |
| 26147 | St. Clair County, MI | 0.808 | 0.01 | Low RR + Low P | No | 0.178 | 319542 |
| 22027 | Claiborne Parish, LA | 0.611 | 0.43 | Low RR + Low P | No | 0.178 | 27519 |
| 21149 | McLean County, KY | 0.675 | 0.42 | Low RR + Low P | No | 0.178 | 18161 |
| 26037 | Clinton County, MI | 0.739 | 0.12 | Low RR + Low P | No | 0.178 | 159372 |
| 25021 | Norfolk County, MA | 0.498 | 0.25 | Low RR + Low P | No | 0.177 | 1452942 |
| 22033 | East Baton Rouge Parish, LA | 0.243 | 0.31 | Low RR + Low P | No | 0.177 | 898558 |
| 5109 | Pike County, AR | 0.636 | 0.43 | Low RR + Low P | No | 0.176 | 20367 |
| 39017 | Butler County, OH | 0.809 | 0 | Low RR + Low P | No | 0.176 | 782771 |
| 42131 | Wyoming County, PA | 0.883 | 0.01 | Low RR + Low P | No | 0.175 | 51926 |
| 29059 | Dallas County, MO | 0.801 | 0.2 | Low RR + Low P | No | 0.174 | 35355 |
| 47105 | Loudon County, TN | 0.705 | 0.17 | Low RR + Low P | No | 0.174 | 118835 |
| 51127 | New Kent County, VA | 0.403 | 0.44 | Low RR + Low P | No | 0.173 | 51086 |
| 17111 | McHenry County, IL | 0.552 | 0.22 | Low RR + Low P | No | 0.173 | 624358 |
| 37025 | Cabarrus County, NC | 0.761 | 0.02 | Low RR + Low P | No | 0.173 | 475925 |
| 6059 | Orange County, CA | 0.78 | 0 | Low RR + Low P | No | 0.173 | 6286127 |
| 56043 | Washakie County, WY | 0.712 | 0.39 | Low RR + Low P | No | 0.172 | 15434 |
| 39025 | Clermont County, OH | 0.734 | 0.04 | Low RR + Low P | No | 0.172 | 422651 |
| 51147 | Prince Edward County, VA | 0.821 | 0.12 | Low RR + Low P | No | 0.172 | 43948 |
| 20113 | McPherson County, KS | 0.819 | 0.06 | Low RR + Low P | No | 0.171 | 60154 |
| 39159 | Union County, OH | 0.752 | 0.07 | Low RR + Low P | No | 0.17 | 136513 |
| 42045 | Delaware County, PA | 0.714 | 0.03 | Low RR + Low P | No | 0.17 | 1152593 |
| 48371 | Pecos County, TX | 0.733 | 0.3 | Low RR + Low P | No | 0.17 | 29334 |
| 36081 | Queens County, NY | 0.744 | 0 | Low RR + Low P | No | 0.169 | 4530754 |
| 36027 | Dutchess County, NY | 0.741 | 0.02 | Low RR + Low P | No | 0.169 | 594759 |
| 51019 | Bedford County, VA | 0.757 | 0.04 | Low RR + Low P | No | 0.169 | 162266 |
| 35047 | San Miguel County, NM | 0.753 | 0.18 | Low RR + Low P | No | 0.169 | 53570 |
| 33015 | Rockingham County, NH | 0.679 | 0.07 | Low RR + Low P | No | 0.169 | 640322 |
| 30047 | Lake County, MT | 0.649 | 0.29 | Low RR + Low P | No | 0.168 | 66191 |
| 34031 | Passaic County, NJ | 0.731 | 0.02 | Low RR + Low P | No | 0.168 | 1027029 |
| 19109 | Kossuth County, IA | 0.665 | 0.36 | Low RR + Low P | No | 0.168 | 28861 |
| 13317 | Wilkes County, GA | 0.589 | 0.44 | Low RR + Low P | No | 0.168 | 19083 |
| 12017 | Citrus County, FL | 0.64 | 0.16 | Low RR + Low P | No | 0.167 | 329204 |
| 8083 | Montezuma County, CO | 0.816 | 0.07 | Low RR + Low P | No | 0.167 | 52998 |
| 42095 | Northampton County, PA | 0.784 | 0 | Low RR + Low P | No | 0.167 | 636991 |
| 19121 | Madison County, IA | 0.728 | 0.28 | Low RR + Low P | No | 0.167 | 34011 |
| 13237 | Putnam County, GA | 0.762 | 0.18 | Low RR + Low P | No | 0.166 | 46062 |
| 29177 | Ray County, MO | 0.687 | 0.28 | Low RR + Low P | No | 0.165 | 46303 |
| 55043 | Grant County, WI | 0.777 | 0.04 | Low RR + Low P | No | 0.164 | 102724 |
| 26003 | Alger County, MI | 0.769 | 0.28 | Low RR + Low P | No | 0.163 | 17530 |
| 29105 | Laclede County, MO | 0.634 | 0.27 | Low RR + Low P | No | 0.163 | 73025 |
| 34023 | Middlesex County, NJ | 0.628 | 0.09 | Low RR + Low P | No | 0.163 | 1724717 |
| 36059 | Nassau County, NY | 0.6 | 0.09 | Low RR + Low P | No | 0.162 | 2767009 |
| 48215 | Hidalgo County, TX | 0.746 | 0 | Low RR + Low P | No | 0.161 | 1786757 |
| 6017 | El Dorado County, CA | 0.643 | 0.1 | Low RR + Low P | No | 0.16 | 385002 |
| 1079 | Lawrence County, AL | 0.536 | 0.33 | Low RR + Low P | No | 0.16 | 66689 |
| 36121 | Wyoming County, NY | 0.742 | 0.11 | Low RR + Low P | No | 0.16 | 79133 |
| 18127 | Porter County, IN | 0.774 | 0 | Low RR + Low P | No | 0.159 | 350183 |
| 55007 | Bayfield County, WI | 0.672 | 0.31 | Low RR + Low P | No | 0.159 | 33428 |
| 5007 | Benton County, AR | 0.512 | 0.18 | Low RR + Low P | No | 0.158 | 613835 |
| 1027 | Clay County, AL | 0.406 | 0.44 | Low RR + Low P | No | 0.158 | 28294 |
| 36021 | Columbia County, NY | 0.748 | 0.04 | Low RR + Low P | No | 0.158 | 121578 |
| 20057 | Ford County, KS | 0.796 | 0.04 | Low RR + Low P | No | 0.158 | 67904 |
| 29109 | Lawrence County, MO | 0.642 | 0.22 | Low RR + Low P | No | 0.157 | 77561 |
| 34039 | Union County, NJ | 0.645 | 0.04 | Low RR + Low P | No | 0.157 | 1143143 |
| 6057 | Nevada County, CA | 0.671 | 0.09 | Low RR + Low P | No | 0.157 | 204438 |
| 38003 | Barnes County, ND | 0.827 | 0.13 | Low RR + Low P | No | 0.156 | 21487 |
| 18089 | Lake County, IN | 0.739 | 0 | Low RR + Low P | No | 0.155 | 1000255 |
| 6075 | San Francisco County, CA | 0.732 | 0 | Low RR + Low P | No | 0.155 | 1616762 |
| 51145 | Powhatan County, VA | 0.53 | 0.31 | Low RR + Low P | No | 0.155 | 63690 |
| 20091 | Johnson County, KS | 0.481 | 0.16 | Low RR + Low P | No | 0.155 | 1241328 |
| 1033 | Colbert County, AL | 0.431 | 0.3 | Low RR + Low P | No | 0.155 | 116354 |
| 1111 | Randolph County, AL | 0.346 | 0.41 | Low RR + Low P | No | 0.154 | 45246 |
| 12021 | Collier County, FL | 0.287 | 0.21 | Low RR + Low P | No | 0.153 | 801826 |
| 29127 | Marion County, MO | 0.763 | 0.07 | Low RR + Low P | No | 0.153 | 56825 |
| 29097 | Jasper County, MO | 0.699 | 0.03 | Low RR + Low P | No | 0.152 | 249072 |
| 26111 | Midland County, MI | 0.577 | 0.17 | Low RR + Low P | No | 0.152 | 167801 |
| 17031 | Cook County, IL | 0.706 | 0 | Low RR + Low P | No | 0.151 | 10198638 |
| 51735 | Poquoson city, VA | 0.734 | 0.24 | Low RR + Low P | No | 0.151 | 25259 |
| 29119 | McDonald County, MO | 0.471 | 0.36 | Low RR + Low P | No | 0.151 | 47496 |
| 4015 | Mohave County, AZ | 0.686 | 0.01 | Low RR + Low P | No | 0.15 | 444587 |
| 36097 | Schuyler County, NY | 0.731 | 0.19 | Low RR + Low P | No | 0.149 | 35116 |
| 5115 | Pope County, AR | 0.675 | 0.07 | Low RR + Low P | No | 0.148 | 128646 |
| 36079 | Putnam County, NY | 0.452 | 0.22 | Low RR + Low P | No | 0.147 | 196372 |
| 36103 | Suffolk County, NY | 0.647 | 0.01 | Low RR + Low P | No | 0.147 | 3051144 |
| 1037 | Coosa County, AL | 0.447 | 0.42 | Low RR + Low P | No | 0.147 | 20575 |
| 35041 | Roosevelt County, NM | 0.733 | 0.16 | Low RR + Low P | No | 0.144 | 37645 |
| 35049 | Santa Fe County, NM | 0.475 | 0.17 | Low RR + Low P | No | 0.144 | 311717 |
| 6081 | San Mateo County, CA | 0.687 | 0 | Low RR + Low P | No | 0.144 | 1455282 |
| 18057 | Hamilton County, IN | 0.56 | 0.06 | Low RR + Low P | No | 0.143 | 737108 |
| 17131 | Mercer County, IL | 0.779 | 0.09 | Low RR + Low P | No | 0.142 | 31020 |
| 18013 | Brown County, IN | 0.676 | 0.24 | Low RR + Low P | No | 0.142 | 31240 |
| 29169 | Pulaski County, MO | 0.713 | 0.02 | Low RR + Low P | No | 0.141 | 107622 |
| 12109 | St. Johns County, FL | 0.422 | 0.13 | Low RR + Low P | No | 0.14 | 627182 |
| 51007 | Amelia County, VA | 0.623 | 0.3 | Low RR + Low P | No | 0.14 | 26848 |
| 42041 | Cumberland County, PA | 0.647 | 0.01 | Low RR + Low P | No | 0.14 | 539270 |
| 1117 | Shelby County, AL | 0.535 | 0.07 | Low RR + Low P | No | 0.139 | 463033 |
| 36061 | New York County, NY | 0.663 | 0 | Low RR + Low P | No | 0.139 | 3191994 |
| 34027 | Morris County, NJ | 0.59 | 0.02 | Low RR + Low P | No | 0.139 | 1025642 |
| 17199 | Williamson County, IL | 0.59 | 0.11 | Low RR + Low P | No | 0.138 | 133250 |
| 29031 | Cape Girardeau County, MO | 0.327 | 0.22 | Low RR + Low P | No | 0.138 | 165915 |
| 29163 | Pike County, MO | 0.48 | 0.34 | Low RR + Low P | No | 0.138 | 35579 |
| 18107 | Montgomery County, IN | 0.702 | 0.04 | Low RR + Low P | No | 0.137 | 76892 |
| 51049 | Cumberland County, VA | 0.748 | 0.17 | Low RR + Low P | No | 0.137 | 19629 |
| 48091 | Comal County, TX | 0.638 | 0.01 | Low RR + Low P | No | 0.136 | 378677 |
| 34029 | Ocean County, NJ | 0.61 | 0 | Low RR + Low P | No | 0.135 | 1314860 |
| 36001 | Albany County, NY | 0.642 | 0 | Low RR + Low P | No | 0.135 | 632580 |
| 48175 | Goliad County, TX | 0.774 | 0.16 | Low RR + Low P | No | 0.135 | 14264 |
| 34013 | Essex County, NJ | 0.649 | 0 | Low RR + Low P | No | 0.134 | 1700841 |
| 36069 | Ontario County, NY | 0.649 | 0.02 | Low RR + Low P | No | 0.134 | 224975 |
| 39165 | Warren County, OH | 0.597 | 0.01 | Low RR + Low P | No | 0.132 | 501883 |
| 36087 | Rockland County, NY | 0.601 | 0 | Low RR + Low P | No | 0.131 | 681164 |
| 1055 | Etowah County, AL | 0.503 | 0.1 | Low RR + Low P | No | 0.13 | 206144 |
| 12103 | Pinellas County, FL | 0.574 | 0 | Low RR + Low P | No | 0.13 | 1923388 |
| 12087 | Monroe County, FL | 0.423 | 0.15 | Low RR + Low P | No | 0.128 | 162232 |
| 34035 | Somerset County, NJ | 0.501 | 0.03 | Low RR + Low P | No | 0.128 | 695889 |
| 25025 | Suffolk County, MA | 0.623 | 0 | Low RR + Low P | No | 0.128 | 1537237 |
| 51041 | Chesterfield County, VA | 0.417 | 0.05 | Low RR + Low P | No | 0.126 | 762166 |
| 34017 | Hudson County, NJ | 0.599 | 0 | Low RR + Low P | No | 0.125 | 1407853 |
| 6079 | San Luis Obispo County, CA | 0.628 | 0 | Low RR + Low P | No | 0.124 | 563685 |
| 51087 | Henrico County, VA | 0.431 | 0.04 | Low RR + Low P | No | 0.123 | 669313 |
| 29145 | Newton County, MO | 0.453 | 0.14 | Low RR + Low P | No | 0.123 | 120773 |
| 17097 | Lake County, IL | 0.485 | 0.02 | Low RR + Low P | No | 0.123 | 1418659 |
| 18129 | Posey County, IN | 0.542 | 0.19 | Low RR + Low P | No | 0.122 | 50138 |
| 47187 | Williamson County, TN | 0.474 | 0.03 | Low RR + Low P | No | 0.122 | 525198 |
| 17043 | DuPage County, IL | 0.469 | 0.02 | Low RR + Low P | No | 0.121 | 1842877 |
| 25017 | Middlesex County, MA | 0.495 | 0 | Low RR + Low P | No | 0.119 | 3240746 |
| 22053 | Jefferson Davis Parish, LA | 0.415 | 0.21 | Low RR + Low P | No | 0.119 | 63482 |
| 51075 | Goochland County, VA | 0.306 | 0.25 | Low RR + Low P | No | 0.118 | 53359 |
| 34025 | Monmouth County, NJ | 0.565 | 0 | Low RR + Low P | No | 0.118 | 1287027 |
| 1041 | Crenshaw County, AL | 0.663 | 0.17 | Low RR + Low P | No | 0.118 | 26149 |
| 17115 | Macon County, IL | 0.601 | 0 | Low RR + Low P | No | 0.118 | 201924 |
| 37037 | Chatham County, NC | 0.517 | 0.04 | Low RR + Low P | No | 0.117 | 161563 |
| 36119 | Westchester County, NY | 0.538 | 0 | Low RR + Low P | No | 0.116 | 1982553 |
| 51159 | Richmond County, VA | 0.611 | 0.25 | Low RR + Low P | No | 0.116 | 18310 |
| 29001 | Adair County, MO | 0.519 | 0.18 | Low RR + Low P | No | 0.115 | 50273 |
| 34019 | Hunterdon County, NJ | 0.496 | 0.02 | Low RR + Low P | No | 0.115 | 259988 |
| 1091 | Marengo County, AL | 0.369 | 0.28 | Low RR + Low P | No | 0.114 | 37475 |
| 17093 | Kendall County, IL | 0.509 | 0.02 | Low RR + Low P | No | 0.113 | 277470 |
| 22071 | Orleans Parish, LA | 0.536 | 0 | Low RR + Low P | No | 0.111 | 734053 |
| 37183 | Wake County, NC | 0.467 | 0 | Low RR + Low P | No | 0.107 | 2361053 |
| 34003 | Bergen County, NJ | 0.47 | 0 | Low RR + Low P | No | 0.107 | 1911276 |
| 13159 | Jasper County, GA | 0.587 | 0.14 | Low RR + Low P | No | 0.104 | 32434 |
| 37135 | Orange County, NC | 0.414 | 0.01 | Low RR + Low P | No | 0.102 | 300879 |
| 1077 | Lauderdale County, AL | 0.377 | 0.02 | Low RR + Low P | No | 0.101 | 192689 |
| 1003 | Baldwin County, AL | 0.458 | 0 | Low RR + Low P | No | 0.099 | 500038 |
| 29181 | Ripley County, MO | 0.628 | 0.13 | Low RR + Low P | No | 0.099 | 21520 |
| 1001 | Autauga County, AL | 0.354 | 0.04 | Low RR + Low P | No | 0.097 | 120068 |
| 22079 | Rapides Parish, LA | 0.481 | 0 | Low RR + Low P | No | 0.094 | 253288 |
| 1115 | St. Clair County, AL | 0.383 | 0 | Low RR + Low P | No | 0.09 | 189433 |

| Table S5. Full list of Tier 1 priority counties for CKM-involved CVD mortality in 2022–2023 | | | | | | |
| --- | --- | --- | --- | --- | --- | --- |
| County (State) | Population (2022–2023) | P(increasing) | Excess deaths, n | Excess deaths (per 100,000) | Priority score | Smoothed RR in 2022–2023 (95% CI) |
| Pottawatomie County, OK | 147142 | 1 | 116 | 79 | 0.949 | 2.76 (2.42-3.15) |
| Washington County, OK | 106944 | 1 | 94 | 88 | 0.949 | 2.57 (2.23-2.96) |
| Lee County, MS | 165623 | 1 | 109 | 66 | 0.948 | 2.52 (2.21-2.88) |
| Cleveland County, OK | 600677 | 1 | 221 | 37 | 0.947 | 1.89 (1.73-2.06) |
| Spartanburg County, SC | 702646 | 1 | 268 | 38 | 0.946 | 1.87 (1.73-2.02) |
| Baltimore city, MD | 1134346 | 1 | 419 | 37 | 0.945 | 1.87 (1.76-1.99) |
| Comanche County, OK | 243799 | 0.98 | 164 | 67 | 0.944 | 2.73 (2.43-3.06) |
| Pittsburg County, OK | 86955 | 1 | 104 | 119 | 0.941 | 3.18 (2.76-3.67) |
| Natchitoches Parish, LA | 72939 | 1 | 63 | 86 | 0.938 | 2.91 (2.45-3.45) |
| Canadian County, OK | 345083 | 1 | 115 | 33 | 0.937 | 1.88 (1.68-2.12) |
| Delaware County, OK | 83183 | 1 | 81 | 97 | 0.937 | 2.51 (2.16-2.92) |
| Florence County, SC | 273954 | 1 | 110 | 40 | 0.936 | 1.85 (1.64-2.08) |
| Tangipahoa Parish, LA | 274863 | 1 | 93 | 34 | 0.935 | 1.84 (1.63-2.09) |
| Creek County, OK | 146018 | 0.98 | 116 | 79 | 0.935 | 2.57 (2.25-2.92) |
| Sumter County, SC | 208101 | 1 | 88 | 42 | 0.935 | 1.88 (1.65-2.14) |
| Jones County, MS | 132690 | 1 | 62 | 47 | 0.934 | 1.97 (1.69-2.30) |
| Sullivan County, TN | 322997 | 1 | 154 | 48 | 0.933 | 1.81 (1.64-2.00) |
| Ottawa County, OK | 60616 | 1 | 65 | 107 | 0.931 | 3.26 (2.74-3.88) |
| Warren County, KY | 282275 | 1 | 85 | 30 | 0.931 | 1.80 (1.58-2.06) |
| Bryan County, OK | 97005 | 1 | 56 | 58 | 0.931 | 2.23 (1.88-2.64) |
| Taylor County, TX | 291983 | 1 | 102 | 35 | 0.931 | 1.83 (1.62-2.07) |
| Prentiss County, MS | 50026 | 1 | 44 | 88 | 0.93 | 2.79 (2.29-3.39) |
| Okmulgee County, OK | 74046 | 0.99 | 75 | 101 | 0.93 | 3.05 (2.60-3.59) |
| Wayne County, IN | 132343 | 1 | 73 | 55 | 0.927 | 2.03 (1.76-2.35) |
| Tift County, GA | 82889 | 1 | 38 | 46 | 0.926 | 2.04 (1.69-2.47) |
| Garfield County, OK | 123974 | 0.99 | 68 | 55 | 0.925 | 2.19 (1.87-2.55) |
| Union County, MS | 56416 | 1 | 43 | 75 | 0.925 | 2.63 (2.17-3.20) |
| St. Mary Parish, LA | 94874 | 1 | 45 | 48 | 0.925 | 1.93 (1.62-2.29) |
| Campbell County, KY | 186974 | 1 | 74 | 40 | 0.924 | 1.83 (1.59-2.10) |
| Union County, SC | 53368 | 1 | 45 | 85 | 0.921 | 2.49 (2.07-3.01) |
| Livingston County, IL | 70837 | 1 | 40 | 57 | 0.921 | 2.02 (1.68-2.43) |
| Muscogee County, GA | 404309 | 0.96 | 176 | 44 | 0.919 | 2.06 (1.86-2.27) |
| Douglas County, OR | 224725 | 1 | 119 | 53 | 0.919 | 1.79 (1.60-2.00) |
| McCurtain County, OK | 61433 | 0.98 | 55 | 89 | 0.916 | 2.90 (2.42-3.48) |
| Dillon County, SC | 55445 | 1 | 28 | 50 | 0.915 | 2.03 (1.64-2.52) |
| Perry County, KY | 54500 | 0.99 | 40 | 73 | 0.914 | 2.55 (2.09-3.10) |
| Payne County, OK | 166053 | 0.98 | 55 | 33 | 0.913 | 1.90 (1.62-2.22) |
| Linn County, OR | 262118 | 0.99 | 105 | 40 | 0.913 | 1.79 (1.59-2.02) |
| Garvin County, OK | 51636 | 0.99 | 35 | 68 | 0.912 | 2.40 (1.96-2.94) |
| Leflore County, MS | 53042 | 0.98 | 75 | 142 | 0.911 | 4.42 (3.72-5.24) |
| Greene County, PA | 68886 | 0.99 | 36 | 53 | 0.907 | 1.98 (1.64-2.39) |
| Pike County, KY | 112290 | 0.97 | 60 | 54 | 0.905 | 2.02 (1.72-2.36) |
| Carroll County, TN | 57278 | 0.98 | 41 | 72 | 0.904 | 2.36 (1.94-2.85) |
| Knox County, IN | 71946 | 0.97 | 49 | 68 | 0.903 | 2.32 (1.94-2.78) |
| Claiborne County, TN | 65011 | 0.99 | 33 | 50 | 0.902 | 1.96 (1.61-2.39) |
| Butts County, GA | 53632 | 0.99 | 24 | 44 | 0.9 | 2.02 (1.62-2.53) |
| Vernon Parish, LA | 93120 | 0.97 | 38 | 41 | 0.899 | 2.07 (1.71-2.50) |
| Wagoner County, OK | 175977 | 0.94 | 89 | 51 | 0.898 | 2.11 (1.84-2.42) |
| Mayes County, OK | 79459 | 0.94 | 68 | 85 | 0.896 | 2.75 (2.33-3.24) |
| Kay County, OK | 87379 | 0.95 | 56 | 65 | 0.894 | 2.24 (1.90-2.65) |
| Obion County, TN | 60785 | 0.98 | 32 | 53 | 0.893 | 1.96 (1.61-2.39) |
| Marinette County, WI | 84072 | 0.96 | 63 | 74 | 0.891 | 2.13 (1.82-2.49) |
| Avoyelles Parish, LA | 77123 | 0.93 | 75 | 97 | 0.889 | 2.95 (2.51-3.47) |
| Monroe County, MS | 67177 | 0.95 | 51 | 76 | 0.889 | 2.36 (1.98-2.82) |
| Tate County, MS | 56520 | 0.93 | 58 | 103 | 0.886 | 3.32 (2.77-3.98) |
| Robeson County, NC | 233831 | 0.92 | 92 | 39 | 0.883 | 1.94 (1.70-2.21) |
| Stephens County, OK | 87630 | 0.91 | 79 | 90 | 0.881 | 2.63 (2.26-3.07) |
| Boyd County, KY | 95885 | 0.91 | 80 | 83 | 0.88 | 2.53 (2.18-2.94) |
| Osage County, OK | 91982 | 0.93 | 60 | 65 | 0.88 | 2.15 (1.83-2.53) |
| Geary County, KS | 70576 | 0.92 | 34 | 48 | 0.879 | 2.80 (2.26-3.47) |
| Wise County, TX | 152994 | 0.94 | 55 | 36 | 0.879 | 1.84 (1.58-2.16) |
| Clark County, KY | 74296 | 0.97 | 30 | 41 | 0.876 | 1.82 (1.49-2.21) |
| Carter County, OK | 96932 | 0.9 | 70 | 72 | 0.875 | 2.59 (2.21-3.05) |
| Bedford County, TN | 105019 | 0.9 | 61 | 58 | 0.874 | 2.43 (2.06-2.87) |
| St. Martin Parish, LA | 102306 | 0.89 | 62 | 61 | 0.864 | 2.31 (1.97-2.73) |
| Gibson County, TN | 101871 | 0.89 | 58 | 57 | 0.864 | 2.19 (1.86-2.58) |
| Upson County, GA | 56356 | 0.92 | 31 | 55 | 0.86 | 2.11 (1.72-2.59) |
| Lowndes County, MS | 114954 | 0.86 | 64 | 56 | 0.853 | 2.15 (1.84-2.52) |
| Angelina County, TX | 174443 | 0.85 | 88 | 51 | 0.851 | 2.10 (1.83-2.41) |
| Franklin County, VT | 101713 | 0.89 | 46 | 45 | 0.85 | 1.93 (1.63-2.29) |
| Custer County, OK | 56305 | 0.88 | 27 | 48 | 0.84 | 2.14 (1.72-2.65) |
| Cowlitz County, WA | 224928 | 0.87 | 90 | 40 | 0.839 | 1.78 (1.57-2.01) |
| Roane County, TN | 111225 | 0.84 | 63 | 56 | 0.829 | 1.91 (1.64-2.22) |
| Hawkins County, TN | 116503 | 0.84 | 57 | 49 | 0.828 | 1.87 (1.60-2.18) |
| Marshall County, IA | 79835 | 0.82 | 41 | 51 | 0.816 | 2.02 (1.69-2.43) |

| Table S6. State-level distribution of benchmark-based excess deaths for CKM-involved CVD mortality, United States, 2022–2023. | | | | |
| --- | --- | --- | --- | --- |
| State | Counties, n | Population | Excess deaths, n | Excess deaths (per 100,000) |
| Oklahoma | 77 | 8073095 | 5018 | 62.160 |
| Texas | 254 | 60533149 | 4120 | 6.805 |
| Kentucky | 120 | 9037717 | 2052 | 22.702 |
| Mississippi | 82 | 5878618 | 1942 | 33.033 |
| South Carolina | 46 | 10656510 | 1865 | 17.504 |
| Wisconsin | 72 | 11801498 | 1693 | 14.346 |
| Tennessee | 95 | 14175465 | 1341 | 9.457 |
| California | 58 | 78005809 | 1337 | 1.714 |
| Oregon | 36 | 8472737 | 1058 | 12.483 |
| Minnesota | 87 | 11452215 | 964 | 8.418 |
| Maryland | 24 | 12344234 | 955 | 7.737 |
| Iowa | 99 | 6406697 | 868 | 13.547 |
| Ohio | 88 | 23545632 | 747 | 3.174 |
| West Virginia | 55 | 3544106 | 588 | 16.587 |
| Indiana | 92 | 13694473 | 485 | 3.541 |
| Washington | 39 | 15597357 | 431 | 2.766 |
| Nebraska | 93 | 3946439 | 383 | 9.717 |
| Colorado | 64 | 11718649 | 342 | 2.917 |
| Delaware | 3 | 2051349 | 331 | 16.116 |
| Vermont | 14 | 1294574 | 326 | 25.168 |
| Nevada | 17 | 6371597 | 293 | 4.595 |
| Georgia | 159 | 21942377 | 292 | 1.331 |
| Rhode Island | 5 | 2189804 | 278 | 12.714 |
| District of Columbia | 1 | 1349921 | 253 | 18.733 |
| Idaho | 44 | 3903722 | 249 | 6.390 |
| Michigan | 83 | 20070542 | 187 | 0.931 |
| South Dakota | 65 | 1802245 | 144 | 7.969 |
| Wyoming | 23 | 1165686 | 69 | 5.908 |
| Louisiana | 64 | 9161772 | 45 | 0.487 |
| North Dakota | 53 | 1562838 | 28 | 1.781 |
| Montana | 56 | 2255690 | 18 | 0.787 |
| North Carolina | 100 | 21531456 | 7 | 0.033 |
| Arkansas | 75 | 6114136 | -4 | -0.063 |
| Alaska | 27 | 1431516 | -90 | -6.322 |
| Kansas | 105 | 5877262 | -153 | -2.608 |
| Hawaii | 5 | 2874537 | -220 | -7.645 |
| New Hampshire | 10 | 2801057 | -236 | -8.417 |
| New Mexico | 33 | 4227847 | -277 | -6.553 |
| Utah | 29 | 6798970 | -298 | -4.382 |
| Maine | 16 | 2785060 | -341 | -12.246 |
| Missouri | 115 | 12373324 | -641 | -5.181 |
| Pennsylvania | 67 | 25933774 | -1032 | -3.981 |
| Alabama | 67 | 10182371 | -1259 | -12.368 |
| Arizona | 15 | 14797028 | -1352 | -9.136 |
| Virginia | 133 | 17394797 | -1885 | -10.834 |
| Illinois | 102 | 25132204 | -2523 | -10.040 |
| Massachusetts | 14 | 13984139 | -2558 | -18.290 |
| New York | 62 | 39244416 | -3070 | -7.823 |
| New Jersey | 21 | 18551658 | -3254 | -17.542 |
| Florida | 67 | 44856247 | -7795 | -17.377 |

| Table S7. Model fit statistics for negative binomial specifications and test of SVI×time interaction. | | | | | | |
| --- | --- | --- | --- | --- | --- | --- |
| Model specification | Dispersion (θ) | SE(θ) | AIC | Log-likelihood | Degrees of freedom | LRT p-value (interaction) |
| NB: quintile main | 6.563 | 0.095 | 127302.3 | -63644.2 | 15672 |  |
| NB: quintile×time | 6.568 | 0.095 | 127298.6 | -63638.3 | 15668 | 0.0197 |
| NB: svi×time | 6.584 | 0.096 | 127282.3 | -63636.2 | 15674 |  |

| Table S8. Observed versus model-fitted deaths and standardized mortality ratios by biennial period and SVI quintile. | | | | | | | |
| --- | --- | --- | --- | --- | --- | --- | --- |
| Biennial period | SVI quintile | Population | Observed deaths (O) | Expected deaths (E) | Fitted deaths (μ) | SMR (observed) = O/E | SMR (fitted) = μ/E |
| 2014-2015 | Q1 | 77687461 | 24409 | 29231 | 28950 | 0.835 | 0.99 |
| 2014-2015 | Q2 | 1.27E+08 | 42304 | 47509 | 48834 | 0.89 | 1.028 |
| 2014-2015 | Q3 | 1.56E+08 | 56270 | 58406 | 62427 | 0.963 | 1.069 |
| 2014-2015 | Q4 | 1.76E+08 | 66359 | 61783 | 70820 | 1.074 | 1.146 |
| 2014-2015 | Q5 | 1.03E+08 | 43065 | 35462 | 43072 | 1.214 | 1.215 |
| 2016-2017 | Q1 | 83721480 | 27947 | 33467 | 33544 | 0.835 | 1.002 |
| 2016-2017 | Q2 | 1.29E+08 | 46001 | 51375 | 53536 | 0.895 | 1.042 |
| 2016-2017 | Q3 | 1.61E+08 | 62060 | 64949 | 70347 | 0.956 | 1.083 |
| 2016-2017 | Q4 | 1.68E+08 | 66954 | 63155 | 74110 | 1.06 | 1.173 |
| 2016-2017 | Q5 | 1.06E+08 | 49075 | 39087 | 49152 | 1.256 | 1.257 |
| 2018-2019 | Q1 | 83745971 | 30564 | 35402 | 35910 | 0.863 | 1.014 |
| 2018-2019 | Q2 | 1.36E+08 | 50902 | 56620 | 59814 | 0.899 | 1.056 |
| 2018-2019 | Q3 | 1.58E+08 | 62560 | 66631 | 73132 | 0.939 | 1.098 |
| 2018-2019 | Q4 | 1.78E+08 | 75549 | 69603 | 83615 | 1.085 | 1.201 |
| 2018-2019 | Q5 | 99744735 | 47096 | 38467 | 50081 | 1.224 | 1.302 |
| 2020-2021 | Q1 | 65921542 | 29789 | 33548 | 34439 | 0.888 | 1.027 |
| 2020-2021 | Q2 | 1.24E+08 | 52884 | 60383 | 64667 | 0.876 | 1.071 |
| 2020-2021 | Q3 | 1.21E+08 | 57934 | 59757 | 66463 | 0.969 | 1.112 |
| 2020-2021 | Q4 | 1.7E+08 | 83860 | 83345 | 102501 | 1.006 | 1.23 |
| 2020-2021 | Q5 | 1.75E+08 | 90860 | 76715 | 103402 | 1.184 | 1.348 |
| 2022-2023 | Q1 | 70224226 | 31962 | 35441 | 36820 | 0.902 | 1.039 |
| 2022-2023 | Q2 | 1.15E+08 | 49036 | 56773 | 61638 | 0.864 | 1.086 |
| 2022-2023 | Q3 | 1.31E+08 | 63546 | 64216 | 72377 | 0.99 | 1.127 |
| 2022-2023 | Q4 | 1.69E+08 | 81239 | 80693 | 101594 | 1.007 | 1.259 |
| 2022-2023 | Q5 | 1.75E+08 | 90805 | 77690 | 108414 | 1.169 | 1.395 |

| Table S9. Sensitivity of Q5 vs Q1 disparity estimates to region adjustment and state fixed effects | | | |
| --- | --- | --- | --- |
| Period | Base NB model RR (Q5 vs Q1) | Region-adjusted NB model RR (Q5 vs Q1) | State fixed effects NB model RR (Q5 vs Q1) |
| 2014-2015 | 1.23 (1.18–1.28) | 1.25 (1.19–1.30) | 1.38 (1.33–1.43) |
| 2018-2019 | 1.28 (1.25–1.31) | 1.31 (1.28–1.34) | 1.44 (1.40–1.48) |
| 2022-2023 | 1.34 (1.29–1.40) | 1.38 (1.33–1.43) | 1.50 (1.45–1.56) |

| Table S10. Pandemic-structure sensitivity analyses for the Q5 vs Q1 disparity estimate at 2022-2023 | | | |
| --- | --- | --- | --- |
| Specification | AIC | Mid-year (biennial period) | RR (Q5 vs Q1) at 2022-2023 (95% CI) |
| Main | 127298.6 | 2022-2023 | 1.34 (1.29–1.39) |
| Add pandemic indicator | 127300.6 | 2022-2023 | 1.34 (1.29–1.39) |
| Piecewise post-2020 | 127305.1 | 2022-2023 | 1.34 (1.26–1.43) |
| Exclude 20–21 | 101136.2 | 2022-2023 | 1.34 (1.28–1.40) |

| Table S11. INLA Bayesian spatiotemporal model comparison using DIC and WAIC | | | | | | | |
| --- | --- | --- | --- | --- | --- | --- | --- |
| Model | Model components | DIC | WAIC | Effective parameters (pD) | ΔDIC (vs best) | ΔWAIC (vs best) | Rank (by WAIC) |
| M3: M2 + obs_iid | BYM2 (spatial) + RW1 (temporal) + ST_iid (unstructured spatiotemporal) | 106605.5 | 106468.4 | 7928 | 0 | 0 | 1 |
| M2: BYM2+RW1+ST_iid | BYM2 (spatial) + RW1 (temporal) + ST_iid (unstructured spatiotemporal) | 106608.3 | 106471.5 | 7927.5 | 2.8 | 3.1 | 2 |
| M1: BYM2+RW1 | BYM2 (spatial) + RW1 (temporal) | 116093.3 | 124189.5 | 2394.8 | 9487.8 | 17721.1 | 3 |

| Table S12. County-level Getis–Ord Gi* hotspot statistics for smoothed RR of CKM-involved CVD mortality, 2022–2023. | | | | | | |
| --- | --- | --- | --- | --- | --- | --- |
| County FIPS | County (State) | Smoothed RR in 2022–2023 | P(increasing) | Gi* z-score | Gi* p-value (two-sided) | Gi* category |
| 40091 | McIntosh County, OK | 2.4 | 0.552 | 6.26 | <0.001 | Hot spot (p<0.01) |
| 40107 | Okfuskee County, OK | 3.45 | 0.919 | 5.84 | <0.001 | Hot spot (p<0.01) |
| 40131 | Rogers County, OK | 2.41 | 1 | 5.76 | <0.001 | Hot spot (p<0.01) |
| 40143 | Tulsa County, OK | 2.49 | 1 | 5.72 | <0.001 | Hot spot (p<0.01) |
| 40145 | Wagoner County, OK | 2.11 | 0.941 | 5.72 | <0.001 | Hot spot (p<0.01) |
| 40037 | Creek County, OK | 2.57 | 0.985 | 5.64 | <0.001 | Hot spot (p<0.01) |
| 40111 | Okmulgee County, OK | 3.05 | 0.991 | 5.61 | <0.001 | Hot spot (p<0.01) |
| 40061 | Haskell County, OK | 2.66 | 0.896 | 5.52 | <0.001 | Hot spot (p<0.01) |
| 40063 | Hughes County, OK | 2.32 | 0.568 | 5.45 | <0.001 | Hot spot (p<0.01) |
| 40021 | Cherokee County, OK | 2.46 | 0.699 | 5.43 | <0.001 | Hot spot (p<0.01) |
| 40125 | Pottawatomie County, OK | 2.76 | 0.996 | 5.29 | <0.001 | Hot spot (p<0.01) |
| 40123 | Pontotoc County, OK | 2.26 | 0.626 | 5.27 | <0.001 | Hot spot (p<0.01) |
| 40077 | Latimer County, OK | 1.9 | 0.376 | 5.12 | <0.001 | Hot spot (p<0.01) |
| 40101 | Muskogee County, OK | 3.49 | 0.995 | 5.05 | <0.001 | Hot spot (p<0.01) |
| 40019 | Carter County, OK | 2.59 | 0.897 | 5.02 | <0.001 | Hot spot (p<0.01) |
| 40005 | Atoka County, OK | 1.59 | 0.81 | 4.95 | <0.001 | Hot spot (p<0.01) |
| 40137 | Stephens County, OK | 2.63 | 0.914 | 4.93 | <0.001 | Hot spot (p<0.01) |
| 40081 | Lincoln County, OK | 2.39 | 0.741 | 4.92 | <0.001 | Hot spot (p<0.01) |
| 40127 | Pushmataha County, OK | 3.07 | 0.955 | 4.91 | <0.001 | Hot spot (p<0.01) |
| 28071 | Lafayette County, MS | 1.69 | 0.975 | 4.87 | <0.001 | Hot spot (p<0.01) |
| 40121 | Pittsburg County, OK | 3.18 | 0.996 | 4.71 | <0.001 | Hot spot (p<0.01) |
| 40097 | Mayes County, OK | 2.75 | 0.94 | 4.68 | <0.001 | Hot spot (p<0.01) |
| 40031 | Comanche County, OK | 2.73 | 0.985 | 4.63 | <0.001 | Hot spot (p<0.01) |
| 28115 | Pontotoc County, MS | 1.5 | 0.989 | 4.62 | <0.001 | Hot spot (p<0.01) |
| 28017 | Chickasaw County, MS | 2.8 | 1 | 4.61 | <0.001 | Hot spot (p<0.01) |
| 21131 | Leslie County, KY | 2.7 | 0.372 | 4.59 | <0.001 | Hot spot (p<0.01) |
| 40119 | Payne County, OK | 1.9 | 0.985 | 4.52 | <0.001 | Hot spot (p<0.01) |
| 40133 | Seminole County, OK | 2.9 | 0.537 | 4.49 | <0.001 | Hot spot (p<0.01) |
| 40049 | Garvin County, OK | 2.4 | 0.993 | 4.49 | <0.001 | Hot spot (p<0.01) |
| 40051 | Grady County, OK | 2.14 | 0.766 | 4.48 | <0.001 | Hot spot (p<0.01) |
| 48269 | King County, TX | 1.48 | 0.714 | 4.45 | <0.001 | Hot spot (p<0.01) |
| 40083 | Logan County, OK | 1.78 | 0.35 | 4.4 | <0.001 | Hot spot (p<0.01) |
| 40033 | Cotton County, OK | 2.19 | 0.424 | 4.4 | <0.001 | Hot spot (p<0.01) |
| 40103 | Noble County, OK | 2.34 | 0.9 | 4.37 | <0.001 | Hot spot (p<0.01) |
| 40069 | Johnston County, OK | 2.15 | 0.801 | 4.36 | <0.001 | Hot spot (p<0.01) |
| 40075 | Kiowa County, OK | 2.58 | 0.641 | 4.31 | <0.001 | Hot spot (p<0.01) |
| 40087 | McClain County, OK | 1.91 | 0.667 | 4.31 | <0.001 | Hot spot (p<0.01) |
| 40015 | Caddo County, OK | 2.15 | 0.576 | 4.3 | <0.001 | Hot spot (p<0.01) |
| 21193 | Perry County, KY | 2.55 | 0.99 | 4.3 | <0.001 | Hot spot (p<0.01) |
| 40135 | Sequoyah County, OK | 2.27 | 0.684 | 4.3 | <0.001 | Hot spot (p<0.01) |
| 40017 | Canadian County, OK | 1.88 | 0.999 | 4.2 | <0.001 | Hot spot (p<0.01) |
| 40117 | Pawnee County, OK | 3.37 | 0.961 | 4.13 | <0.001 | Hot spot (p<0.01) |
| 40029 | Coal County, OK | 1.94 | 0.795 | 4.04 | <0.001 | Hot spot (p<0.01) |
| 28043 | Grenada County, MS | 1.43 | 0.642 | 4.04 | <0.001 | Hot spot (p<0.01) |
| 40113 | Osage County, OK | 2.15 | 0.93 | 4.02 | <0.001 | Hot spot (p<0.01) |
| 28081 | Lee County, MS | 2.52 | 0.998 | 4.01 | <0.001 | Hot spot (p<0.01) |
| 40011 | Blaine County, OK | 1.8 | 0.854 | 4 | <0.001 | Hot spot (p<0.01) |
| 40079 | Le Flore County, OK | 3.13 | 1 | 3.97 | <0.001 | Hot spot (p<0.01) |
| 40035 | Craig County, OK | 3.14 | 0.846 | 3.91 | <0.001 | Hot spot (p<0.01) |
| 40141 | Tillman County, OK | 2.25 | 0.828 | 3.91 | <0.001 | Hot spot (p<0.01) |
| 28025 | Clay County, MS | 2.44 | 0.971 | 3.87 | <0.001 | Hot spot (p<0.01) |
| 40109 | Oklahoma County, OK | 2.46 | 1 | 3.87 | <0.001 | Hot spot (p<0.01) |
| 40149 | Washita County, OK | 1.61 | 0.941 | 3.87 | <0.001 | Hot spot (p<0.01) |
| 40099 | Murray County, OK | 2.13 | 0.716 | 3.85 | <0.001 | Hot spot (p<0.01) |
| 21013 | Bell County, KY | 2.8 | 0.684 | 3.81 | <0.001 | Hot spot (p<0.01) |
| 40067 | Jefferson County, OK | 2.58 | 0.667 | 3.81 | <0.001 | Hot spot (p<0.01) |
| 40023 | Choctaw County, OK | 2.3 | 0.885 | 3.79 | <0.001 | Hot spot (p<0.01) |
| 40089 | McCurtain County, OK | 2.9 | 0.983 | 3.75 | <0.001 | Hot spot (p<0.01) |
| 40129 | Roger Mills County, OK | 1.32 | 0.593 | 3.73 | <0.001 | Hot spot (p<0.01) |
| 40147 | Washington County, OK | 2.57 | 1 | 3.69 | <0.001 | Hot spot (p<0.01) |
| 40039 | Custer County, OK | 2.14 | 0.884 | 3.68 | <0.001 | Hot spot (p<0.01) |
| 48329 | Midland County, TX | 0.97 | 0.853 | 3.66 | <0.001 | Hot spot (p<0.01) |
| 40093 | Major County, OK | 1.85 | 0.714 | 3.66 | <0.001 | Hot spot (p<0.01) |
| 40073 | Kingfisher County, OK | 1.8 | 0.93 | 3.63 | <0.001 | Hot spot (p<0.01) |
| 40027 | Cleveland County, OK | 1.89 | 1 | 3.57 | <0.001 | Hot spot (p<0.01) |
| 28067 | Jones County, MS | 1.97 | 1 | 3.53 | <0.001 | Hot spot (p<0.01) |
| 47113 | Madison County, TN | 1.49 | 0.598 | 3.52 | <0.001 | Hot spot (p<0.01) |
| 40055 | Greer County, OK | 1.67 | 0.816 | 3.51 | <0.001 | Hot spot (p<0.01) |
| 40095 | Marshall County, OK | 2.16 | 0.669 | 3.49 | <0.001 | Hot spot (p<0.01) |
| 28155 | Webster County, MS | 2.34 | 0.414 | 3.47 | 0.001 | Hot spot (p<0.01) |
| 21121 | Knox County, KY | 1.72 | 0.959 | 3.45 | 0.001 | Hot spot (p<0.01) |
| 28003 | Alcorn County, MS | 0.7 | 0.691 | 3.36 | 0.001 | Hot spot (p<0.01) |
| 40009 | Beckham County, OK | 2.61 | 0.999 | 3.35 | 0.001 | Hot spot (p<0.01) |
| 21235 | Whitley County, KY | 1.65 | 0.376 | 3.34 | 0.001 | Hot spot (p<0.01) |
| 54099 | Wayne County, WV | 1.51 | 0.941 | 3.33 | 0.001 | Hot spot (p<0.01) |
| 48125 | Dickens County, TX | 1.9 | 0.595 | 3.31 | 0.001 | Hot spot (p<0.01) |
| 48337 | Montague County, TX | 1.3 | 0.306 | 3.28 | 0.001 | Hot spot (p<0.01) |
| 28135 | Tallahatchie County, MS | 1.04 | 0.765 | 3.27 | 0.001 | Hot spot (p<0.01) |
| 28127 | Simpson County, MS | 0.84 | 0.419 | 3.24 | 0.001 | Hot spot (p<0.01) |
| 48105 | Crockett County, TX | 1.74 | 0.481 | 3.23 | 0.001 | Hot spot (p<0.01) |
| 40047 | Garfield County, OK | 2.19 | 0.989 | 3.19 | 0.001 | Hot spot (p<0.01) |
| 28073 | Lamar County, MS | 0.9 | 0.81 | 3.19 | 0.001 | Hot spot (p<0.01) |
| 48461 | Upton County, TX | 2.15 | 0.806 | 3.18 | 0.001 | Hot spot (p<0.01) |
| 48383 | Reagan County, TX | 2.49 | 0.557 | 3.18 | 0.001 | Hot spot (p<0.01) |
| 21119 | Knott County, KY | 1.83 | 0.191 | 3.17 | 0.002 | Hot spot (p<0.01) |
| 28013 | Calhoun County, MS | 2.98 | 0.92 | 3.16 | 0.002 | Hot spot (p<0.01) |
| 28145 | Union County, MS | 2.63 | 0.998 | 3.16 | 0.002 | Hot spot (p<0.01) |
| 21051 | Clay County, KY | 3.17 | 0.743 | 3.16 | 0.002 | Hot spot (p<0.01) |
| 28053 | Humphreys County, MS | 1.9 | 0.41 | 3.13 | 0.002 | Hot spot (p<0.01) |
| 48487 | Wilbarger County, TX | 1.45 | 0.565 | 3.12 | 0.002 | Hot spot (p<0.01) |
| 28161 | Yalobusha County, MS | 1.63 | 0.84 | 3.12 | 0.002 | Hot spot (p<0.01) |
| 21189 | Owsley County, KY | 1.22 | 0.419 | 3.11 | 0.002 | Hot spot (p<0.01) |
| 48387 | Red River County, TX | 1.5 | 0.806 | 3.06 | 0.002 | Hot spot (p<0.01) |
| 48483 | Wheeler County, TX | 1.64 | 0.931 | 3.05 | 0.002 | Hot spot (p<0.01) |
| 48077 | Clay County, TX | 1.62 | 0.654 | 3.04 | 0.002 | Hot spot (p<0.01) |
| 40041 | Delaware County, OK | 2.51 | 0.999 | 3.03 | 0.002 | Hot spot (p<0.01) |
| 21133 | Letcher County, KY | 1.54 | 0.36 | 3.03 | 0.002 | Hot spot (p<0.01) |
| 21153 | Magoffin County, KY | 1.16 | 0.452 | 3 | 0.003 | Hot spot (p<0.01) |
| 46069 | Hyde County, SD | 1.73 | 0.391 | 3 | 0.003 | Hot spot (p<0.01) |
| 31089 | Holt County, NE | 0.85 | 0.578 | 2.99 | 0.003 | Hot spot (p<0.01) |
| 48197 | Hardeman County, TX | 1.2 | 0.399 | 2.98 | 0.003 | Hot spot (p<0.01) |
| 40085 | Love County, OK | 2.25 | 0.845 | 2.97 | 0.003 | Hot spot (p<0.01) |
| 48023 | Baylor County, TX | 1.6 | 0.863 | 2.96 | 0.003 | Hot spot (p<0.01) |
| 28137 | Tate County, MS | 3.32 | 0.934 | 2.95 | 0.003 | Hot spot (p<0.01) |
| 40065 | Jackson County, OK | 1.91 | 0.839 | 2.93 | 0.003 | Hot spot (p<0.01) |
| 21045 | Casey County, KY | 1.69 | 0.76 | 2.9 | 0.004 | Hot spot (p<0.01) |
| 40153 | Woodward County, OK | 1.91 | 0.917 | 2.88 | 0.004 | Hot spot (p<0.01) |
| 21095 | Harlan County, KY | 2.38 | 0.78 | 2.88 | 0.004 | Hot spot (p<0.01) |
| 28015 | Carroll County, MS | 1.68 | 0.456 | 2.84 | 0.005 | Hot spot (p<0.01) |
| 48503 | Young County, TX | 1.46 | 0.62 | 2.84 | 0.005 | Hot spot (p<0.01) |
| 40043 | Dewey County, OK | 2.94 | 0.916 | 2.83 | 0.005 | Hot spot (p<0.01) |
| 45009 | Bamberg County, SC | 1.68 | 0.669 | 2.79 | 0.005 | Hot spot (p<0.01) |
| 21149 | McLean County, KY | 0.68 | 0.421 | 2.79 | 0.005 | Hot spot (p<0.01) |
| 47053 | Gibson County, TN | 2.19 | 0.894 | 2.78 | 0.005 | Hot spot (p<0.01) |
| 48211 | Hemphill County, TX | 1.55 | 0.589 | 2.78 | 0.005 | Hot spot (p<0.01) |
| 54059 | Mingo County, WV | 2.27 | 0.165 | 2.78 | 0.005 | Hot spot (p<0.01) |
| 48179 | Gray County, TX | 1.57 | 0.728 | 2.78 | 0.005 | Hot spot (p<0.01) |
| 28039 | George County, MS | 1.05 | 0.959 | 2.77 | 0.006 | Hot spot (p<0.01) |
| 31009 | Blaine County, NE | 1.31 | 0.649 | 2.77 | 0.006 | Hot spot (p<0.01) |
| 48173 | Glasscock County, TX | 2.96 | 0.69 | 2.76 | 0.006 | Hot spot (p<0.01) |
| 48009 | Archer County, TX | 1.38 | 0.536 | 2.75 | 0.006 | Hot spot (p<0.01) |
| 13073 | Columbia County, GA | 1.3 | 1 | 2.73 | 0.006 | Hot spot (p<0.01) |
| 45071 | Newberry County, SC | 1.29 | 0.777 | 2.72 | 0.007 | Hot spot (p<0.01) |
| 48485 | Wichita County, TX | 1.83 | 0.096 | 2.72 | 0.007 | Hot spot (p<0.01) |
| 48227 | Howard County, TX | 1.86 | 0.777 | 2.72 | 0.007 | Hot spot (p<0.01) |
| 40105 | Nowata County, OK | 3.84 | 0.97 | 2.71 | 0.007 | Hot spot (p<0.01) |
| 48037 | Bowie County, TX | 1.75 | 0.105 | 2.71 | 0.007 | Hot spot (p<0.01) |
| 48275 | Knox County, TX | 1.95 | 0.724 | 2.71 | 0.007 | Hot spot (p<0.01) |
| 21069 | Fleming County, KY | 1.3 | 0.136 | 2.71 | 0.007 | Hot spot (p<0.01) |
| 21199 | Pulaski County, KY | 1.52 | 0.821 | 2.71 | 0.007 | Hot spot (p<0.01) |
| 48451 | Tom Green County, TX | 1.16 | 0.956 | 2.7 | 0.007 | Hot spot (p<0.01) |
| 21165 | Menifee County, KY | 1.25 | 0.458 | 2.69 | 0.007 | Hot spot (p<0.01) |
| 30025 | Fallon County, MT | 1.52 | 0.603 | 2.69 | 0.007 | Hot spot (p<0.01) |
| 45027 | Clarendon County, SC | 1.57 | 0.666 | 2.69 | 0.007 | Hot spot (p<0.01) |
| 21021 | Boyle County, KY | 1.51 | 0.881 | 2.68 | 0.007 | Hot spot (p<0.01) |
| 40013 | Bryan County, OK | 2.23 | 0.998 | 2.65 | 0.008 | Hot spot (p<0.01) |
| 28083 | Leflore County, MS | 4.42 | 0.975 | 2.65 | 0.008 | Hot spot (p<0.01) |
| 48433 | Stonewall County, TX | 1.81 | 0.618 | 2.64 | 0.008 | Hot spot (p<0.01) |
| 28131 | Stone County, MS | 0.97 | 0.57 | 2.62 | 0.009 | Hot spot (p<0.01) |
| 40045 | Ellis County, OK | 1.58 | 0.889 | 2.61 | 0.009 | Hot spot (p<0.01) |
| 27109 | Olmsted County, MN | 1.43 | 0.69 | 2.61 | 0.009 | Hot spot (p<0.01) |
| 28117 | Prentiss County, MS | 2.79 | 1 | 2.6 | 0.009 | Hot spot (p<0.01) |
| 48431 | Sterling County, TX | 1.84 | 0.619 | 2.6 | 0.009 | Hot spot (p<0.01) |
| 28113 | Pike County, MS | 1.43 | 0.9 | 2.59 | 0.010 | Hot spot (p<0.01) |
| 30033 | Garfield County, MT | 1.56 | 0.398 | 2.59 | 0.010 | Hot spot (p<0.01) |
| 31149 | Rock County, NE | 1.83 | 0.636 | 2.58 | 0.010 | Hot spot (p<0.01) |
| 21025 | Breathitt County, KY | 2.49 | 0.845 | 2.58 | 0.010 | Hot spot (p<0.01) |
| 48317 | Martin County, TX | 1.47 | 0.625 | 2.58 | 0.010 | Hot spot (p<0.01) |
| 48207 | Haskell County, TX | 1.19 | 0.6 | 2.57 | 0.010 | Hot spot (p<0.05) |
| 47183 | Weakley County, TN | 1.53 | 0.994 | 2.56 | 0.011 | Hot spot (p<0.05) |
| 45059 | Laurens County, SC | 1.64 | 1 | 2.55 | 0.011 | Hot spot (p<0.05) |
| 21159 | Martin County, KY | 1.83 | 0.825 | 2.54 | 0.011 | Hot spot (p<0.05) |
| 21173 | Montgomery County, KY | 1.64 | 0.33 | 2.54 | 0.011 | Hot spot (p<0.05) |
| 48155 | Foard County, TX | 2.53 | 0.672 | 2.51 | 0.012 | Hot spot (p<0.05) |
| 21001 | Adair County, KY | 1.59 | 0.983 | 2.5 | 0.012 | Hot spot (p<0.05) |
| 21135 | Lewis County, KY | 1.83 | 0.468 | 2.5 | 0.012 | Hot spot (p<0.05) |
| 48107 | Crosby County, TX | 1.38 | 0.369 | 2.49 | 0.013 | Hot spot (p<0.05) |
| 54109 | Wyoming County, WV | 1.29 | 0.416 | 2.49 | 0.013 | Hot spot (p<0.05) |
| 51105 | Lee County, VA | 0.93 | 0.525 | 2.48 | 0.013 | Hot spot (p<0.05) |
| 21097 | Harrison County, KY | 1.03 | 0.188 | 2.48 | 0.013 | Hot spot (p<0.05) |
| 28093 | Marshall County, MS | 1.44 | 0.974 | 2.47 | 0.014 | Hot spot (p<0.05) |
| 37147 | Pitt County, NC | 1.6 | 0.052 | 2.47 | 0.014 | Hot spot (p<0.05) |
| 13163 | Jefferson County, GA | 2 | 0.31 | 2.46 | 0.014 | Hot spot (p<0.05) |
| 48415 | Scurry County, TX | 0.7 | 0.499 | 2.46 | 0.014 | Hot spot (p<0.05) |
| 45003 | Aiken County, SC | 0.95 | 0.861 | 2.46 | 0.014 | Hot spot (p<0.05) |
| 30017 | Custer County, MT | 1.35 | 0.829 | 2.44 | 0.015 | Hot spot (p<0.05) |
| 28111 | Perry County, MS | 2.32 | 0.759 | 2.44 | 0.015 | Hot spot (p<0.05) |
| 28077 | Lawrence County, MS | 1.53 | 0.99 | 2.43 | 0.015 | Hot spot (p<0.05) |
| 28107 | Panola County, MS | 3.43 | 0.626 | 2.43 | 0.015 | Hot spot (p<0.05) |
| 45005 | Allendale County, SC | 2.07 | 0.802 | 2.43 | 0.015 | Hot spot (p<0.05) |
| 13033 | Burke County, GA | 1.52 | 0.249 | 2.42 | 0.016 | Hot spot (p<0.05) |
| 13279 | Toombs County, GA | 1.53 | 0.561 | 2.42 | 0.016 | Hot spot (p<0.05) |
| 20125 | Montgomery County, KS | 1.07 | 0.59 | 2.41 | 0.016 | Hot spot (p<0.05) |
| 46073 | Jerauld County, SD | 1.09 | 0.381 | 2.4 | 0.016 | Hot spot (p<0.05) |
| 48345 | Motley County, TX | 1.8 | 0.279 | 2.4 | 0.016 | Hot spot (p<0.05) |
| 48151 | Fisher County, TX | 1.14 | 0.58 | 2.4 | 0.016 | Hot spot (p<0.05) |
| 28163 | Yazoo County, MS | 1.36 | 0.919 | 2.39 | 0.017 | Hot spot (p<0.05) |
| 48225 | Houston County, TX | 1.16 | 0.6 | 2.39 | 0.017 | Hot spot (p<0.05) |
| 31017 | Brown County, NE | 1.47 | 0.379 | 2.39 | 0.017 | Hot spot (p<0.05) |
| 47077 | Henderson County, TN | 1.82 | 0.451 | 2.39 | 0.017 | Hot spot (p<0.05) |
| 21089 | Greenup County, KY | 1.83 | 0.082 | 2.39 | 0.017 | Hot spot (p<0.05) |
| 47017 | Carroll County, TN | 2.36 | 0.979 | 2.38 | 0.017 | Hot spot (p<0.05) |
| 20199 | Wallace County, KS | 2.14 | 0.604 | 2.38 | 0.017 | Hot spot (p<0.05) |
| 37107 | Lenoir County, NC | 1.69 | 0.019 | 2.38 | 0.017 | Hot spot (p<0.05) |
| 46035 | Davison County, SD | 1.33 | 0.649 | 2.37 | 0.018 | Hot spot (p<0.05) |
| 37015 | Bertie County, NC | 1.33 | 0.15 | 2.36 | 0.018 | Hot spot (p<0.05) |
| 38001 | Adams County, ND | 1.05 | 0.484 | 2.35 | 0.019 | Hot spot (p<0.05) |
| 48347 | Nacogdoches County, TX | 1.56 | 0.991 | 2.35 | 0.019 | Hot spot (p<0.05) |
| 28051 | Holmes County, MS | 2.52 | 0.976 | 2.35 | 0.019 | Hot spot (p<0.05) |
| 21171 | Monroe County, KY | 1.28 | 0.295 | 2.34 | 0.019 | Hot spot (p<0.05) |
| 28033 | DeSoto County, MS | 1.27 | 1 | 2.34 | 0.019 | Hot spot (p<0.05) |
| 54043 | Lincoln County, WV | 1.84 | 0.895 | 2.33 | 0.020 | Hot spot (p<0.05) |
| 55083 | Oconto County, WI | 1.73 | 0.848 | 2.33 | 0.020 | Hot spot (p<0.05) |
| 47033 | Crockett County, TN | 1.58 | 0.98 | 2.32 | 0.020 | Hot spot (p<0.05) |
| 54045 | Logan County, WV | 1.77 | 0.435 | 2.32 | 0.020 | Hot spot (p<0.05) |
| 45041 | Florence County, SC | 1.85 | 1 | 2.32 | 0.020 | Hot spot (p<0.05) |
| 45085 | Sumter County, SC | 1.88 | 1 | 2.31 | 0.021 | Hot spot (p<0.05) |
| 48145 | Falls County, TX | 1.75 | 0.445 | 2.31 | 0.021 | Hot spot (p<0.05) |
| 08017 | Cheyenne County, CO | 1.91 | 0.579 | 2.31 | 0.021 | Hot spot (p<0.05) |
| 48293 | Limestone County, TX | 1.72 | 0.776 | 2.29 | 0.022 | Hot spot (p<0.05) |
| 40059 | Harper County, OK | 1.35 | 0.69 | 2.29 | 0.022 | Hot spot (p<0.05) |
| 48235 | Irion County, TX | 2.4 | 0.681 | 2.29 | 0.022 | Hot spot (p<0.05) |
| 05081 | Little River County, AR | 1.5 | 0.76 | 2.29 | 0.022 | Hot spot (p<0.05) |
| 22085 | Sabine Parish, LA | 0.71 | 0.882 | 2.28 | 0.023 | Hot spot (p<0.05) |
| 37117 | Martin County, NC | 1.78 | 0.645 | 2.28 | 0.023 | Hot spot (p<0.05) |
| 47131 | Obion County, TN | 1.96 | 0.983 | 2.28 | 0.023 | Hot spot (p<0.05) |
| 21125 | Laurel County, KY | 1.53 | 0.767 | 2.28 | 0.023 | Hot spot (p<0.05) |
| 30021 | Dawson County, MT | 0.6 | 0.506 | 2.28 | 0.023 | Hot spot (p<0.05) |
| 46041 | Dewey County, SD | 1.64 | 0.565 | 2.28 | 0.023 | Hot spot (p<0.05) |
| 20071 | Greeley County, KS | 2.53 | 0.662 | 2.28 | 0.023 | Hot spot (p<0.05) |
| 28035 | Forrest County, MS | 1.58 | 0.836 | 2.27 | 0.023 | Hot spot (p<0.05) |
| 21019 | Boyd County, KY | 2.53 | 0.911 | 2.27 | 0.023 | Hot spot (p<0.05) |
| 21047 | Christian County, KY | 1.18 | 0.731 | 2.26 | 0.024 | Hot spot (p<0.05) |
| 48135 | Ector County, TX | 1.52 | 0 | 2.26 | 0.024 | Hot spot (p<0.05) |
| 48075 | Childress County, TX | 0.98 | 0.703 | 2.26 | 0.024 | Hot spot (p<0.05) |
| 48129 | Donley County, TX | 1.22 | 0.527 | 2.25 | 0.024 | Hot spot (p<0.05) |
| 28007 | Attala County, MS | 0.83 | 0.598 | 2.25 | 0.024 | Hot spot (p<0.05) |
| 21011 | Bath County, KY | 1.34 | 0.476 | 2.24 | 0.025 | Hot spot (p<0.05) |
| 39087 | Lawrence County, OH | 1.59 | 0.406 | 2.24 | 0.025 | Hot spot (p<0.05) |
| 21127 | Lawrence County, KY | 1.36 | 0.264 | 2.23 | 0.026 | Hot spot (p<0.05) |
| 48237 | Jack County, TX | 1.84 | 0.884 | 2.23 | 0.026 | Hot spot (p<0.05) |
| 30065 | Musselshell County, MT | 0.91 | 0.557 | 2.23 | 0.026 | Hot spot (p<0.05) |
| 48273 | Kleberg County, TX | 1.4 | 0.191 | 2.23 | 0.026 | Hot spot (p<0.05) |
| 45069 | Marlboro County, SC | 0.94 | 0.818 | 2.22 | 0.026 | Hot spot (p<0.05) |
| 21043 | Carter County, KY | 2.06 | 0.258 | 2.22 | 0.026 | Hot spot (p<0.05) |
| 48065 | Carson County, TX | 1.25 | 0.791 | 2.21 | 0.027 | Hot spot (p<0.05) |
| 48101 | Cottle County, TX | 2.59 | 0.627 | 2.21 | 0.027 | Hot spot (p<0.05) |
| 40057 | Harmon County, OK | 1.82 | 0.621 | 2.2 | 0.028 | Hot spot (p<0.05) |
| 55115 | Shawano County, WI | 1.45 | 1 | 2.19 | 0.029 | Hot spot (p<0.05) |
| 28085 | Lincoln County, MS | 1.56 | 0.981 | 2.18 | 0.029 | Hot spot (p<0.05) |
| 48395 | Robertson County, TX | 1.89 | 0.478 | 2.18 | 0.029 | Hot spot (p<0.05) |
| 46015 | Brule County, SD | 0.93 | 0.42 | 2.18 | 0.029 | Hot spot (p<0.05) |
| 48087 | Collingsworth County, TX | 1.58 | 0.833 | 2.17 | 0.030 | Hot spot (p<0.05) |
| 30087 | Rosebud County, MT | 1.74 | 0.968 | 2.17 | 0.030 | Hot spot (p<0.05) |
| 18027 | Daviess County, IN | 1.67 | 0.902 | 2.16 | 0.031 | Hot spot (p<0.05) |
| 48005 | Angelina County, TX | 2.1 | 0.846 | 2.16 | 0.031 | Hot spot (p<0.05) |
| 28095 | Monroe County, MS | 2.36 | 0.948 | 2.16 | 0.031 | Hot spot (p<0.05) |
| 19037 | Chickasaw County, IA | 0.81 | 0.739 | 2.16 | 0.031 | Hot spot (p<0.05) |
| 28009 | Benton County, MS | 1.19 | 0.472 | 2.15 | 0.032 | Hot spot (p<0.05) |
| 47023 | Chester County, TN | 1.57 | 0.804 | 2.15 | 0.032 | Hot spot (p<0.05) |
| 48277 | Lamar County, TX | 1.47 | 0.787 | 2.15 | 0.032 | Hot spot (p<0.05) |
| 48429 | Stephens County, TX | 1.62 | 0.647 | 2.15 | 0.032 | Hot spot (p<0.05) |
| 48195 | Hansford County, TX | 1.17 | 0.561 | 2.14 | 0.032 | Hot spot (p<0.05) |
| 13145 | Harris County, GA | 1.22 | 0.922 | 2.14 | 0.032 | Hot spot (p<0.05) |
| 21237 | Wolfe County, KY | 2.33 | 0.938 | 2.13 | 0.033 | Hot spot (p<0.05) |
| 13245 | Richmond County, GA | 2.34 | 1 | 2.13 | 0.033 | Hot spot (p<0.05) |
| 31041 | Custer County, NE | 1.55 | 0.726 | 2.12 | 0.034 | Hot spot (p<0.05) |
| 28153 | Wayne County, MS | 2.29 | 0.029 | 2.12 | 0.034 | Hot spot (p<0.05) |
| 05091 | Miller County, AR | 1.5 | 0.816 | 2.12 | 0.034 | Hot spot (p<0.05) |
| 21197 | Powell County, KY | 2.01 | 0.252 | 2.12 | 0.034 | Hot spot (p<0.05) |
| 20099 | Labette County, KS | 0.86 | 0.882 | 2.12 | 0.034 | Hot spot (p<0.05) |
| 30071 | Phillips County, MT | 1.03 | 0.555 | 2.11 | 0.035 | Hot spot (p<0.05) |
| 28139 | Tippah County, MS | 1.8 | 0.995 | 2.11 | 0.035 | Hot spot (p<0.05) |
| 22125 | West Feliciana Parish, LA | 0.98 | 0.879 | 2.11 | 0.035 | Hot spot (p<0.05) |
| 21129 | Lee County, KY | 1.45 | 0.289 | 2.11 | 0.035 | Hot spot (p<0.05) |
| 19075 | Grundy County, IA | 1.34 | 0.823 | 2.11 | 0.035 | Hot spot (p<0.05) |
| 48153 | Floyd County, TX | 1.17 | 0.589 | 2.1 | 0.036 | Hot spot (p<0.05) |
| 40003 | Alfalfa County, OK | 1.26 | 0.369 | 2.1 | 0.036 | Hot spot (p<0.05) |
| 48309 | McLennan County, TX | 1.78 | 0.591 | 2.09 | 0.037 | Hot spot (p<0.05) |
| 21057 | Cumberland County, KY | 1.63 | 0.356 | 2.09 | 0.037 | Hot spot (p<0.05) |
| 20075 | Hamilton County, KS | 1.09 | 0.634 | 2.08 | 0.038 | Hot spot (p<0.05) |
| 21071 | Floyd County, KY | 1.69 | 0.795 | 2.08 | 0.038 | Hot spot (p<0.05) |
| 48357 | Ochiltree County, TX | 0.95 | 0.616 | 2.08 | 0.038 | Hot spot (p<0.05) |
| 48253 | Jones County, TX | 1.3 | 0.625 | 2.08 | 0.038 | Hot spot (p<0.05) |
| 48191 | Hall County, TX | 1.36 | 0.596 | 2.08 | 0.038 | Hot spot (p<0.05) |
| 13263 | Talbot County, GA | 1.36 | 0.706 | 2.07 | 0.039 | Hot spot (p<0.05) |
| 22029 | Concordia Parish, LA | 0.88 | 0.374 | 2.06 | 0.039 | Hot spot (p<0.05) |
| 46107 | Potter County, SD | 1.42 | 0.52 | 2.06 | 0.039 | Hot spot (p<0.05) |
| 46031 | Corson County, SD | 1.88 | 0.509 | 2.05 | 0.040 | Hot spot (p<0.05) |
| 48401 | Rusk County, TX | 1.44 | 0.879 | 2.05 | 0.040 | Hot spot (p<0.05) |
| 48447 | Throckmorton County, TX | 1.94 | 0.539 | 2.03 | 0.042 | Hot spot (p<0.05) |
| 46129 | Walworth County, SD | 1.42 | 0.565 | 2.03 | 0.042 | Hot spot (p<0.05) |
| 48367 | Parker County, TX | 1.21 | 0.755 | 2.02 | 0.043 | Hot spot (p<0.05) |
| 48283 | La Salle County, TX | 1.2 | 0.495 | 2.02 | 0.043 | Hot spot (p<0.05) |
| 19013 | Black Hawk County, IA | 1.76 | 0.986 | 2.02 | 0.043 | Hot spot (p<0.05) |
| 45075 | Orangeburg County, SC | 1.55 | 0.927 | 2.01 | 0.044 | Hot spot (p<0.05) |
| 05133 | Sevier County, AR | 1.07 | 0.833 | 2.01 | 0.044 | Hot spot (p<0.05) |
| 22117 | Washington Parish, LA | 1.1 | 0.999 | 2.01 | 0.044 | Hot spot (p<0.05) |
| 46117 | Stanley County, SD | 1.08 | 0.72 | 2.01 | 0.044 | Hot spot (p<0.05) |
| 55041 | Forest County, WI | 1.56 | 0.436 | 2 | 0.046 | Hot spot (p<0.05) |
| 28057 | Itawamba County, MS | 1.19 | 0.579 | 2 | 0.046 | Hot spot (p<0.05) |
| 48233 | Hutchinson County, TX | 1.6 | 0.751 | 2 | 0.046 | Hot spot (p<0.05) |
| 21195 | Pike County, KY | 2.02 | 0.973 | 1.99 | 0.047 | Hot spot (p<0.05) |
| 45061 | Lee County, SC | 1.32 | 0.91 | 1.98 | 0.048 | Hot spot (p<0.05) |
| 19191 | Winneshiek County, IA | 1.36 | 0.873 | 1.98 | 0.048 | Hot spot (p<0.05) |
| 39009 | Athens County, OH | 1.22 | 0.815 | 1.98 | 0.048 | Hot spot (p<0.05) |
| 38095 | Towner County, ND | 1.64 | 0.669 | 1.98 | 0.048 | Hot spot (p<0.05) |
| 31131 | Otoe County, NE | 1.3 | 0.931 | 1.97 | 0.049 | Hot spot (p<0.05) |

# **Supplementary Figures:**


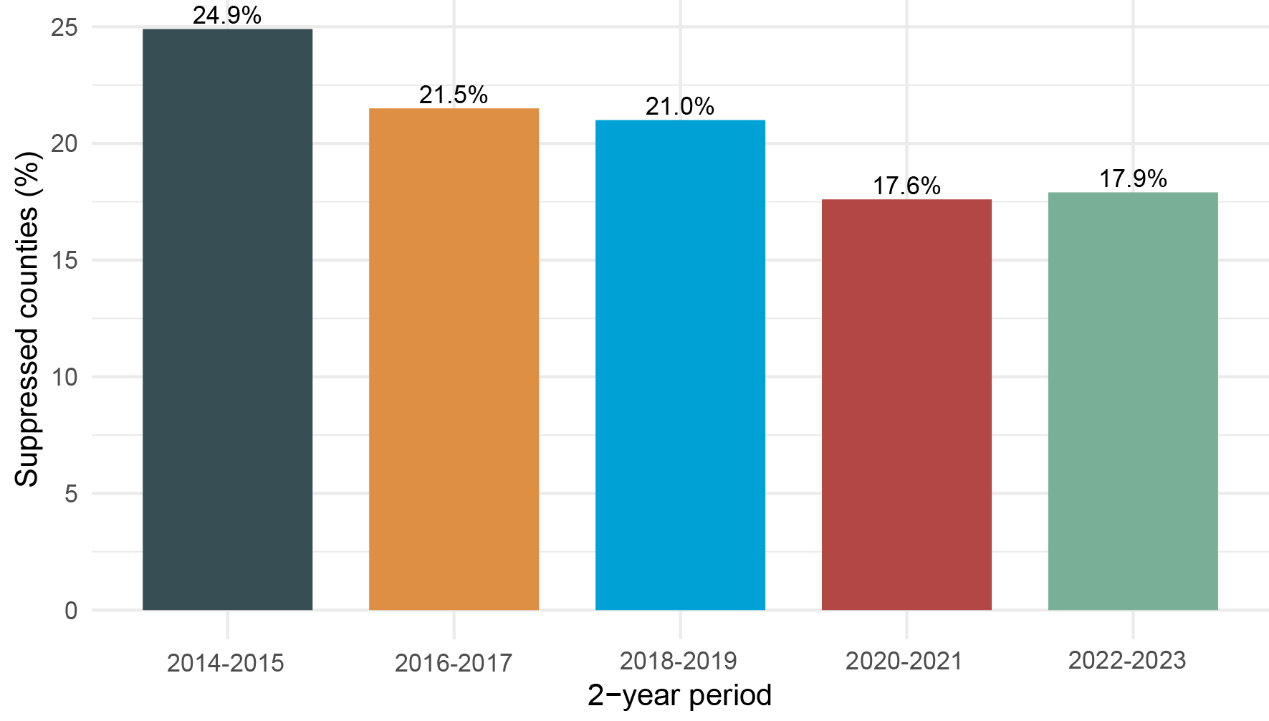


Figure S1. Percentage of counties with suppressed CKM-involved CVD death counts by biennial period, United States, 2014–2023.


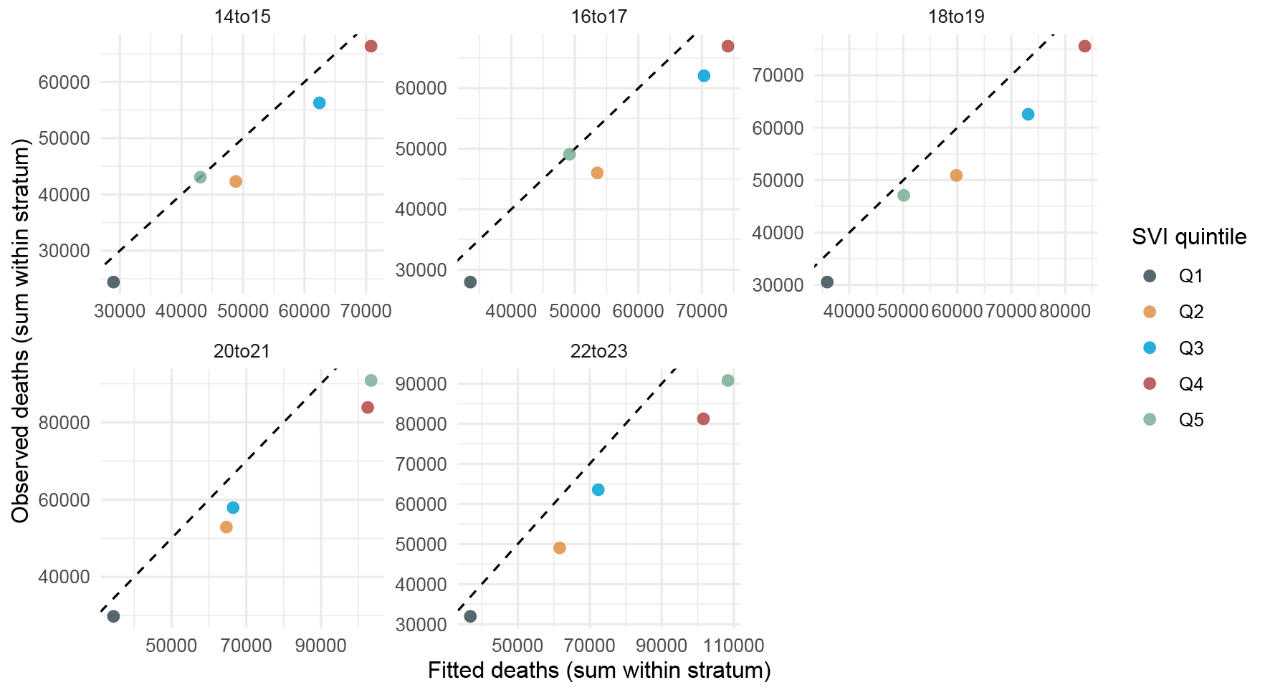


Figure S2. Negative binomial model diagnostics: observed versus fitted deaths by biennial period and SVI quintile


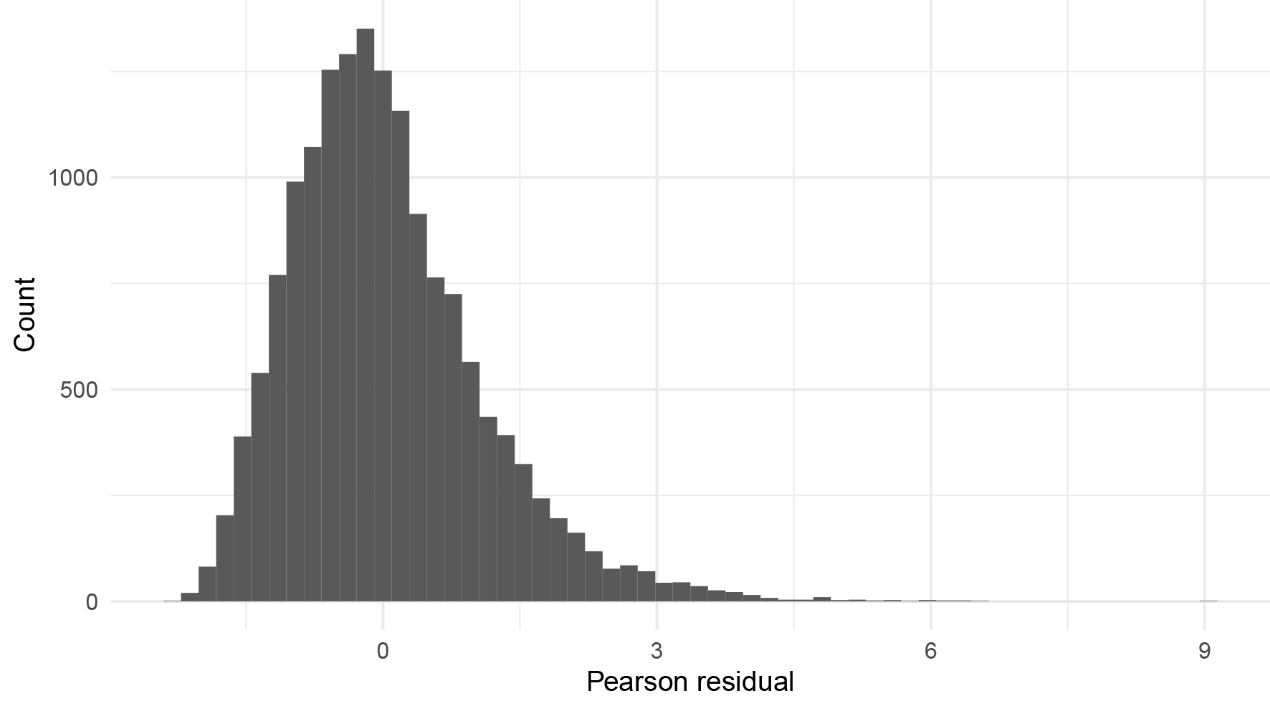


Figure S3. Negative binomial model diagnostics: distribution of Pearson residuals.


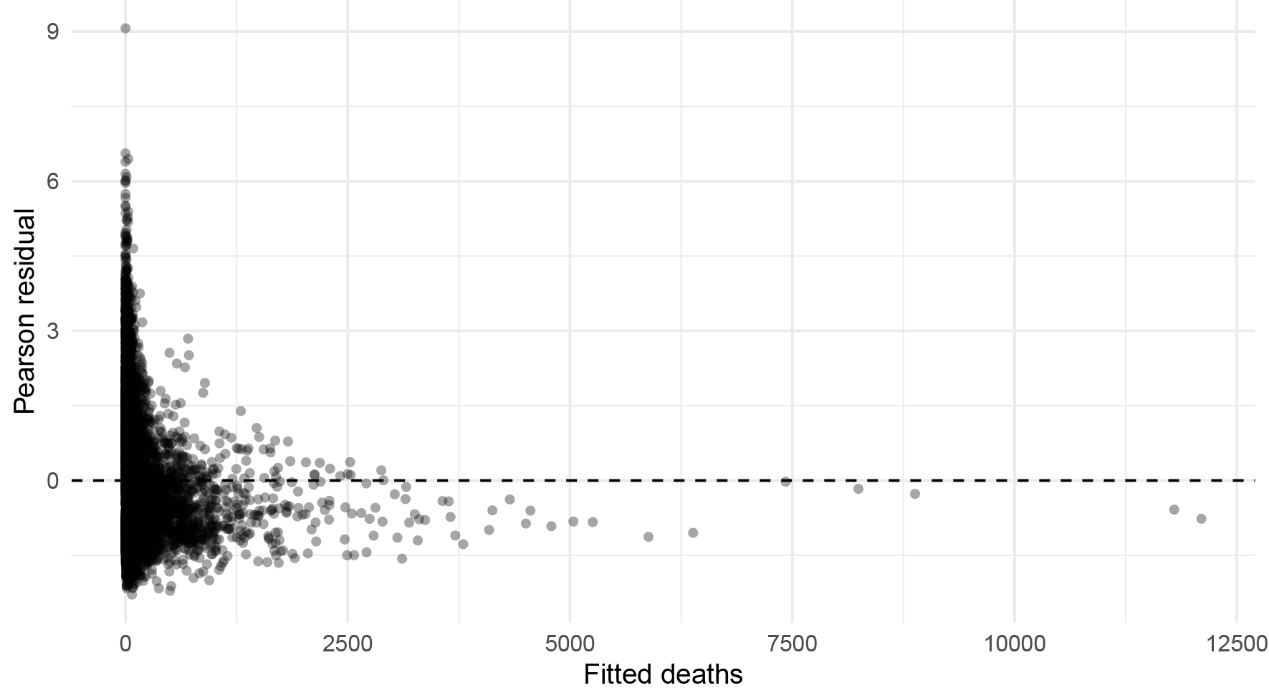


Figure S4. Negative binomial model diagnostics: Pearson residuals versus fitted deaths.


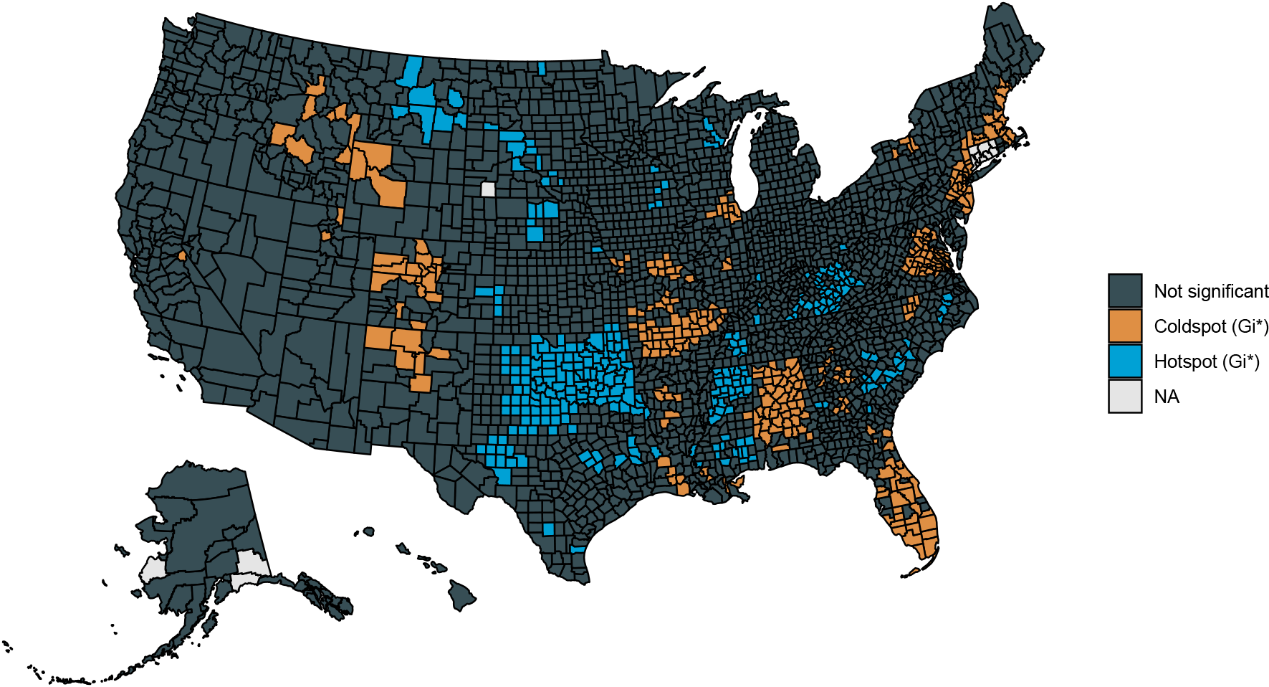


Figure S5. Getis–Ord Gi* hotspot map of smoothed county-level relative risk for CKM-involved CVD mortality, 2022–2023.


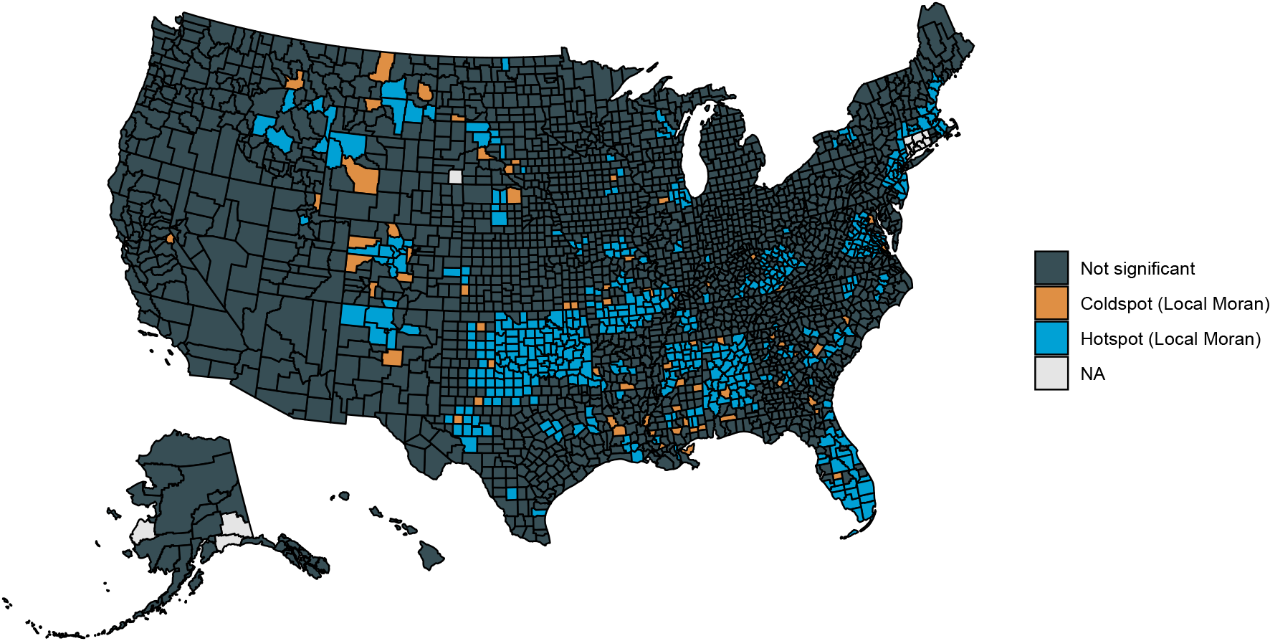


Figure S6. Local Moran’s I cluster/outlier map of smoothed county-level relative risk for CKM-involved CVD mortality, 2022–2023.
